# Use of extracorporeal membrane oxygenation in adult trauma patients with refractory acute cardiopulmonary failure: guideline from the Chinese society of extracorporeal life support 2025

**DOI:** 10.1186/s13054-025-05504-6

**Published:** 2025-07-29

**Authors:** Hua Wang, Zhongran Cen, Xingxing Liu, Zhanguo Liu, Xiaotong Hou, Xiangdong Guan, Jianfeng Wu, Yimin Li, Yonghao Xu, Chengbin Zhou, Zhiyong Peng, Fachun Zhou, Tongwen Sun, Bingyu Qin, Jiandong Lin, Lina Zhang, Jinghui Li, You Shang, Songqiao Liu, Zhenhua Zeng, Xiaowu Wang, Qunqing Chen, Yanwu Guo, Changbiao Peng, Yang Wang, Songjian Li, Chunyao Wang, Shulin Xiang, Zhou Cheng, Peihua Cao, Jie Jiang, Yihao Chen, Duoduo Yu, Wenzhan Liao, Ye Liao, Xiaoqin Cheng, Limei Chen, Yuxuan He, Jie He, Qingling Guo, Zenghui Yue, Ke Deng, Ying Tang, Bo Huang, Cuiping Liu, Sheng Peng, Jing Cai, Yaru Zhu, Kai Wang, Yangyang Wang, Qianwen Wang, Jingjing Yang, Maoyou Shichen, Zhuo Li, Manli Guo, Xueshan Luo, the Chinese Society of Extracorporeal Life Support (CSECLS), the Guangdong Province Restrictive Techniques (ECMO) Clinical Application Training Base

**Affiliations:** 1 Department of Critical Care Medicine, Zhujiang Hospital of Southern Medical University, Guangzhou, China; 2 Department of Critical Care Medicine, Beijing Anzhen Hospital, Capital Medical University, Beijing, China; 3 Department of Critical Care Medicine, The First Affiliated Hospital of Sun Yat-sen University, Guangzhou, China; 4 Department of Critical Care Medicine, The First Affiliated Hospital of Guangzhou Medical University, Guangzhou, China; 5 Department of anesthesiology, Guangdong Provincial People’s Hospital, Guangzhou, China; 6 Department of Critical Care Medicine, Zhongnan Hospital of Wuhan University, Wuhan, China; 7 Department of Critical Care Medicine, The First Affiliated Hospital of Chongqing Medical University, Chongqing, China; 8 Department of Critical Care Medicine, The First Affiliated Hospital of Zhengzhou University, Zhengzhou, China; 9 Department of Critical Care Medicine, Henan Provincial People’s Hospital, Zhengzhou, China; 10 Department of Critical Care Medicine, The First Affiliated Hospital of Fujian Medical University, Fuzhou, China; 11 Department of Critical Care Medicine, Xiangya Hospital of Central South University, Changsha, China; 12 Department of Critical Care Medicine, Haikou People’s Hospital, Haikou, China; 13 Department of Critical Care Medicine, Union Hospital, Tongji Medical College, Huazhong University of Science and Technology, Wuhan, China; 14 Department of Critical Care Medicine, Zhongda Hospital Southeast University, Nanjing, China; 15 Department of Critical Care Medicine, Nanfang Hospital, Southern Medical University, Guangzhou, China; 16 Cardiovascular Surgery, Zhujiang Hospital of Southern Medical University, Guangzhou, China; 17 Thoracic Surgery, Zhujiang Hospital of Southern Medical University, Guangzhou, China; 18 Department of Neurosurgery, Zhujiang Hospital of Southern Medical University, Guangzhou, China; 19 Department of Medical Administration, Zhujiang Hospital of Southern Medical University, Guangzhou, China; 20 Department of Radiology, Zhujiang Hospital of Southern Medical University, Guangzhou, China; 21 Orthopedic Department, Zhujiang Hospital of Southern Medical University, Guangzhou, China; 22 Department of Critical Care Medicine, Peking Union Medical College Hospital, Beijing, China; 23 Department of Critical Care Medicine, The People’s Hospital of Guangxi Zhuang Autonomous Region, Guangxi, China; 24 Department of anesthesiology, Zhongshan City People’s Hospital, Guangdong, China; 25 Department of Biostatistics, School of Public Health, Southern Medical University, Guangzhou, China

**Keywords:** Wounds and injuries, Adult, Extracorporeal membrane oxygenation, Evidence-based medicine

## Abstract

**Background:**

Adult trauma patients with refractory acute cardiopulmonary failure suffer from high morbidity and mortality. In the past decade, a growing body of researches has shown survival benefits of extracorporeal membrane oxygenation (ECMO) in trauma patients who fail to respond to optimal damage control resuscitation (DCR), and there is an opportunity to formulate clinical practice guidelines to guide clinicians in implementing trauma ECMO at the bedside.

**Methods:**

The Chinese Society of Extracorporeal Life Support (CSECLS) convened a domestic panel of interdisciplinary experts to develop this guideline, adhering to the principles of the World Health Organization (WHO) Manual for Guideline Development and the policy of conflict of interest. Clinical key questions pertaining to trauma ECMO use were informed from expert interviews and literature reviews, and formulated as PICO (Population/Intervention/Comparison/Outcome) format for literature retrieval of original studies supporting the question. Then, panelists were assigned to address specific clinical questions, synthesize evidence, formulate recommendations and determine their strength, following the Recommendations Assessment, Development and Evaluation (GRADE) framework. The guideline steering committee and stakeholders approved the final document.

**Results:**

Eleven recommendations regarding trauma ECMO use in adult patients were formulated, focusing on the following topics: (1) indications; (2) patient screening; (3) timing of initiation; (4) multidisciplinary approach; (5) trauma ECMO management; and (6) complication prevention. Supporting evidences are elaborated in detail, and expert opinions on clinical application and future research provided.

**Conclusion:**

Although the quality of the body of evidence is low to very-low, most researches have shown that ECMO improves the survival of adult trauma patients with varied injury mechanisms. However, decision-making should consider the individual characteristics, benefits and potential harms, patients’ values and preferences, and long-term outcomes.

**Supplementary Information:**

The online version contains supplementary material available at10.1186/s13054-025-05504-6.

## Key messages

- Pooled analyses demonstrate comparable survival benefits of ECMO in adult trauma patients with refractory acute cardiopulmonary failure caused by varied injury mechanisms, including polytrauma, traumatic brain injury, moderate to severe burn, and post-traumatic cardiac arrest.
- Success in trauma ECMO treatment includes the implementation of measures in the guidelines, involving appropriate patient selection, early ECMO initiation, multidisciplinary approach, trauma ECMO management, and complication prevention.

**Supplementary Information:**

The online version contains supplementary material available at10.1186/s13054-025-05504-6.

## Background

Severe trauma, defined as an injury severity score (ISS) of > 15, remains the leading cause of death and disability-adjusted life-years among young people in China and worldwide [1, 2]. Of the residents in China, road traffic injuries and high falls rank the 6th and 18th among all causes of death, causing 19 and 10 deaths per 100,000 population each year, respectively, higher than the average of 15.8 and 9.2 deaths per 100,000 population across 195 countries and territories [3, 4]. Hence, improving trauma care has become a priority for the government when formulating public health policies [5].

Traumatic acute respiratory distress syndrome (ARDS) is the primary life-threatening complication leading to late death (> 5 days from injury) among trauma patients, with an overall morbidity and mortality rates of 8.4% and 21.8% in patients with polytrauma [6, 7], 10–25% and 20–80% in those with varied severities of thoracic injury [8], 40.5% and 21.2% in those with traumatic brain injury (TBI) [9], and 43% and 23.4% in those with moderate to severe burn and inhalation injury [10], respectively. Contrarily, traumatic cardiac arrest (TCA) ranks as the leading cause of early death (< 5 days), although it occurs in < 1% of patients with blunt or penetrating chest injury, its pre- and in-hospital mortality are as high as 76–94% and 73% [11–13]. For trauma patients with ARDS or TCA who are refractory to conventional resuscitation, ECMO could provide supplemental cardiopulmonary capacity, maintain gas exchange and hemodynamic stability, thus gaining time for decisive therapies [14]. The mechanism of ECMO is to drain the venous blood from the body to the extracorporeal membrane lung for blood-gas exchange, then return the oxygenated blood via a venous (VV-ECMO) or arterial (VA-ECMO) route, thereby bridging the patient to recovery [15, 16]. Since its first use in 1972 by Dr. Hill for a young male patient with refractory hypoxemia due to road traffic injuries [17], trauma ECMO has expanded on a yearly basis, rendering a substantial survival benefit to the severely injured patients at high-risk of death [18–20].

Currently, the benefits and harms of ECMO to trauma patients remain elusive, and there is a scarcity of published guidelines to guide clinicians at the bedside to implement trauma ECMO [21, 22]. Hence, the Chinese Society of Extracorporeal Life Support (CSECLS) convened a domestic panel of interdisciplinary clinical experts to develop this guideline based on the currently available evidence, focusing on the following topics: (1) indications; (2) patient screening; (3) timing of initiation; (4) multidisciplinary approach; (5) trauma ECMO management; and (6) complication prevention. The guideline is aimed to provide the best practice guidance to clinicians to implement trauma ECMO at the bedside, and the scope is limited to adult trauma patients requiring ECMO. The application settings include medical facilities with ECMO capability, involving emergency department, trauma care center, intensive care unit (ICU), and cardiothoracic surgical unit, etc. The end-users include health professionals in trauma care and ECMO therapy, patients and their proxies, and institutional policymakers.

## Methods

The guideline development working group was established in August 2023 and consisted of a steering group, a domestic expert panel with expertise in critical care medicine, trauma care, and ECMO treatment, an evidence evaluation group, a methodologist, an institutional policymaker and a patient representative. Five experts constituted the external reviewer group. All members disclosed conflicts of interest in accordance with the WHO policies. The guideline was registered with the International Practice Guidelines Registry Platform (Registration No. PREPARE-2023CNN750). Details of the development process and conflict-of-interest forms are accessible online at http://www.guidelines-registry.org.

Based on expert interviews and literature reviews, 34 clinical background questions pertaining trauma ECMO use were identified. Through two rounds of Delphi online expert surveys, eight clinical key questions with outcomes of interest involving six topics were prioritized and formulated as the Population, Intervention, Control, and Outcome (PICO) format. Then, literature retrievals for original studies were conducted as per each question, utilizing databases including PubMed, Embase, Cochrane Library, Medlive.cn, China National Knowledge Internet, Chinese Sci-Tech Journal Database, Sinomed, and Wanfang. The search period was from the inception to February 2024. The literature search strategy and full-text article inclusion criteria are presented in Supplementary material: Tables S1–S2 and Figure S1.

Sixty-seven retrospective studies were included, involving 18 cohort studies [9, 12, 23–38], 16 case-control studies [39–54], and 33 case series [55–87]. The penal was divided into six subgroups to deal with specific clinical problems of relevant domains, performing bias risk assessment, data extraction and evidence synthesis, and consulting with the methodologist. The Newcastle–Ottawa Scale was used for assessing cohort studies [88], the critical appraisal skill program scale for retrospective observational studies [89], and the Joanna Briggs Institute Critical Appraisal Checklist for case series [90]. The characteristics of the included studies and bias risk assessment are shown in Supplementary material: Tables S3–S6. Data reported as median with quartiles were converted into mean and standard deviation using the methods described elsewhere [91, 92]. Single- or two-arm meta-analyses were performed to combine the effect sizes and 95% confidence interval (CI). The maximum likelihood method was used to calculate the variance of study-level heterogeneity, using the Q-test and *I*²value to assess inter-study heterogeneity. Meta-analyses were performed using R software (version 4.3.3, http://www.r-project.org) and the meta package. Statistical analysis plan is accessible online at http://www.guidelines-registry.org.

The Grading of Recommendations Assessment, Development, and Evaluation (GRADE) framework was followed to evaluate the certainty of the body of evidence, formulate recommendations, and determine its strength with the assistance of the online GRADEpro App (https://www.gradepro.org), see Supplementary material: Table S7. In the absence of sufficient data for analyses, evidence supporting the PICO question was expressed narratively and expert opinions were provided. The draft document was discussed through three offline and online meetings and approved if > 75% agreements were obtained through voting by the experts, external reviewers, and patient representatives. The reporting of the guideline follows the Appraisal of Guidelines for Research and Evaluation II and the Reporting Items for Practice Guidelines in Healthcare [93, 94], and will be updated at a 3-year interval as new evidence emerges.

## Results

**Fig. 1:**
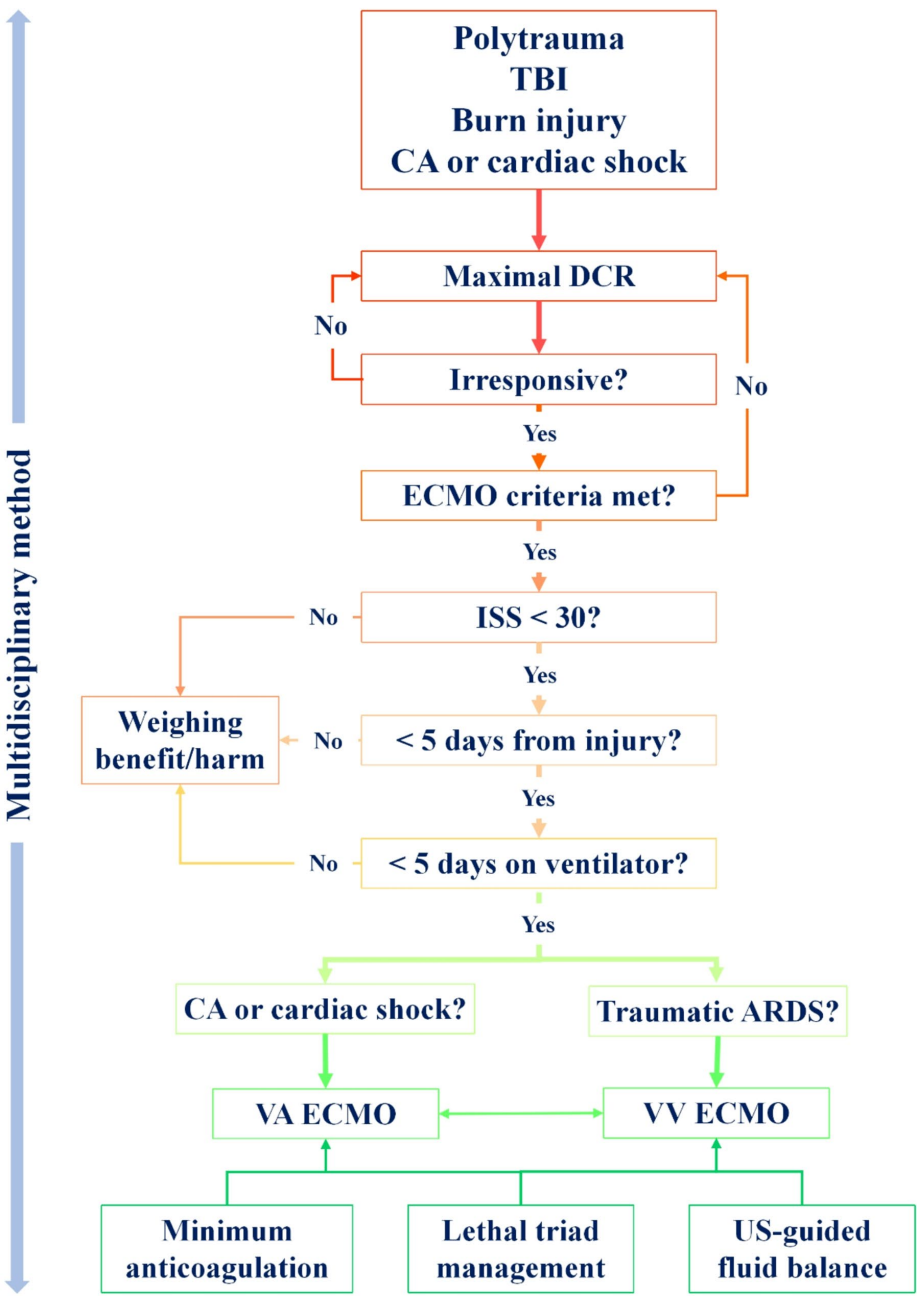
Schematic diagram of the implementation of ECMO in trauma patient. Note: TBI: traumatic brain injury; CA: cardiac arrest; DCR: damage control resuscitation; ECMO: venovenous extracorporeal membrane oxygenation; ISS: injury severity score; ARDS: acute respiratory distress syndrome; VA: venovenous; VA: venoarterial; US: ultrasound

Summary of the clinical key questions and recommendations are shown in Table 1. The schematic diagram of clinical implementation of ECMO in trauma is presented in Fig. 1.

## Domain 1. Indications

*Recommendation 1.* We recommend that VV-ECMO be used for adult patients with polytrauma complicated with ARDS, if conventional lung protective ventilation is ineffective (strong recommendation, very low-quality evidence).

*Recommendation 2.* We recommend that VV-ECMO be used for adult TBI patients with ARDS, if conventional lung protective ventilation is ineffective (strong recommendation, very low-quality evidence).

*Recommendation 3.* We suggest that VV-ECMO be considered for adult trauma patients with ARDS caused by moderate to severe burn and inhalation injury, if optimal fluid resuscitation and lung protective ventilation are ineffective (weak recommendation, very low-quality evidence).

*Recommendation 4.* We suggest that VA-ECMO be considered for selected adult trauma patients with cardiac arrest or cardiogenic shock, if conventional resuscitation is ineffective (weak recommendation, very low-quality evidence).

### Rationale

***Evidence summary.*** Fifty-two retrospective studies involving ECMO-supported adult trauma patients with varied mechanisms of injuries were enrolled in this domain for evidence synthesis, see Supplementary material: Figures S2–S30. Among the studies, 24 were from the United States (1826 patients), 10 from China (110 patients), seven from Europe (251 patients), and five from Korea (61 patients). The mean age of the patients was 36.33 years (95% CI 34.33–38.31) and males accounted for 80.52% (95% CI 77.0–83.62%), with a pooled ISS of 34.17 (95% CI: 31.03–37.3; 1421 patients, 35 studies). The pooled overall survival was 66% (95% CI: 59–72%, *P* = 0.44), which was comparable to that of trauma patients unexposed to ECMO (77,228 patients, 11 studies), with an *OR* of 1.01 (95% CI: 0.43–2.41). No significant differences were found in the survival rates across the geographical regions.

**Table 1:** Summary of the clinical key questions and recommendations

Domains	Clinical questions	Recommendations
Indications	For adult trauma patients with acute cardiopulmonary failure refractory to conventional therapy, should ECMO be used to improve survival?	*Recommendation 1.* We recommend that VV-ECMO be used for adult patients with polytrauma complicated with ARDS, if conventional lung protective ventilation is ineffective (strong recommendation, very low-quality evidence).
		*Recommendation 2*. We recommend that VV-ECMO be used for adult TBI patients with ARDS, if conventional lung protective ventilation is ineffective (strong recommendation, very low-quality evidence).
		*Recommendation 3*. We suggest that VV-ECMO be considered for adult trauma patients with ARDS caused by moderate to severe burn and inhalation injury, if optimal fluid resuscitation and lung protective ventilation are ineffective (weak recommendation, very low-quality evidence).
		*Recommendation 4*. We suggest that VA-ECMO be considered for selected adult trauma patients with cardiac arrest or cardiogenic shock, if conventional resuscitation is ineffective (weak recommendation, very low-quality evidence).
Patient screening	For adult trauma patients with acute cardiopulmonary failure refractory to conventional therapy, should the injury severity score be used as a screening tool for determining the ECMO candidates and treatment success?	*Recommendation 5.* We suggest using the injury severity score as an adjunctive screening tool for determining the ECMO candidate and treatment success in adult trauma patients with acute cardiopulmonary failure refractory to conventional therapy (weak recommendation, very low-quality evidence for both candidate and success).
Timing of initiation	For adult trauma patients with acute cardiopulmonary failure refractory to conventional therapy, should ECMO be initiated earlier to improve survival?	*Recommendation 6.* For adult trauma patients with acute cardiopulmonary failure refractory to conventional therapy, we recommend that ECMO be initiated as early as possible provided that the initiation criteria are met (strong recommendation, very low-quality evidence).
Multidisciplinary approach	In managing adult trauma patients requiring ECMO, should a multidisciplinary approach be used to improve the prognosis?	*Recommendation 7.* We recommend that a multidisciplinary team and quality improvement program be established in the management of adult trauma patients requiring ECMO (strong recommendation, very low-quality evidence).
Trauma ECMO management	In managing adult trauma patients treated with ECMO, should the systemic anticoagulation goal be individualized to reduce anticoagulation-related complications?	*Recommendation 8.* For adult trauma patients treated with ECMO, we suggest minimal anticoagulation strategy be used to achieve individualized systemic anticoagulation goal (weak recommendation, very low-quality evidence).
	In managing severely injured adult trauma patients on ECMO, should coagulopathy, hypothermia, and acidosis be closely managed to improve the prognosis?	*Recommendation 9.* For adult trauma patients with massive bleeding or hemorrhagic shock, we suggest that coagulopathy, hypothermia, and acidosis be closely managed during ECMO treatment (best practice statement).
	In managing adult trauma patients requiring ECMO, should bedside ultrasound be used to optimize fluid management?	*Recommendation 10.* We suggest that bedside ultrasound be used to optimize fluid management in adult trauma patients requiring ECMO (weak recommendation, very low-quality evidence).
Complications	In adult trauma patients with acute cardiopulmonary failure requiring ECMO, should measures be taken to prevent ECMO-related complications?	*Recommendation 11*. We suggest that effective measures be taken to reduce ECMO-related complications in adult trauma patients requiring ECMO (weak recommendation, very low-quality evidence).

Note: VV-ECMO: venovenous extracorporeal membrane oxygenation; TBI: traumatic brain injury; ARDS: acute respiratory distress syndrome

Traumatic ARDS was the most documented indication for VV-ECMO in adult trauma patients, with a pooled overall survival of 68% (95% CI: 64–72%; 549 patients, 28 studies), whereas that of those unexposed to ECMO was 49% (95% CI: 35–62%; 1460 patients, six studies). Likewise, TCA and cardiogenic shock were the most frequent conditions for VA-ECMO. In 18 retrospective studies involving 62 TCA patients on VA-ECMO and 97 patients who shifted from VV to VAV-ECMO due to new-onset cardiac arrest or cardiogenic shock, the pooled overall survival rates were 42% (95% CI: 30–54%) and 55% (95% CI: 26–81%), respectively.

Polytrauma was noted in 38.8% of ECMO-supported adult trauma patients (25 studies, 888 patients), with most of the patients having VV-ECMO due to refractory ARDS and with a pooled survival of 64% (95% CI: 56–71%). When compared with those unexposed to ECMO, the *OR* for survival was 2.38 (95% CI: 0.56–10.1, *P* = 0.24; six studies, 1633 patients). TBI was observed in 14.8% of the ECMO-supported adult trauma patients, with all of them developing ARDS requiring VV-ECMO (14 studies, 340 patients) and with a pooled overall survival rate of 67% (95% CI: 62–72%). When compared with those unexposed to VV-ECMO, the *OR* for survival was 1.41 (95% CI: 0.77–2.58, *P* = 0.26; 12 studies, 216 patients). Burn and inhalation injuries accounted for 4.5% of the patients (nine studies, 103 patients), with pooled total body surface area and survival rate of 42% (95% CI: 26–58%) and 49% (95% CI: 29–70%), respectively.

The pooled duration on ECMO in adult trauma patient was 7.93 (95% CI: 6.76–9.11) days. While the pooled length of stay (LOS) of the patients in the ICU was 21.61 (95% CI: 16.84–26.38) days, and that in hospital was 33.84 (95% CI: 29.62–38.06) days, both were significantly longer than the medians of 9–11.3 days and 5–21.3 days for trauma patients unexposed to ECMO [9, 75].

***Justification and implementation considerations.*** The ELSO guideline recommends that VV-ECMO be used for patients with refractory acute respiratory failure due to thoracic trauma (e.g., traumatic lung injury and severe pulmonary contusion), severe inhalational injury, large bronchopleural fistula, and peri-lung transplant [15]. Our meta-analyses showed that VV-ECMO might confer substantial survival benefits to adult trauma patients with refractory ARDS due to varied injury mechanisms, including polytrauma, TBI, and moderate to severe burn with inhalation injury, which are comparable to that of trauma patients unexposed to ECMO. VV-ECMO facilitates damage control resuscitation in polytraumatized patients, allows neurological protection and lung protective ventilation among TBI patients, and solves the dilemma of aggressive fluid resuscitation while maintaining a “dry lung” in burn patients, thereby gaining time to decisive therapies [14]. Similarly, VA-ECMO increased the survival in patients following traumatic CPR or those with new-onset cardiogenic shock during DCR by nearly 40%. Whereas in settings where ECMO was unavailable, a large retrospective analysis of Trauma Registry involving 814 TCA patients reported that the overall hospital mortality was 73%, and only 7% survived until hospital discharge [13]. Although the quality of evidence is very low due to the non-randomized design, serious inconsistency, imprecision, and heterogeneities across the studies, considering the large survival effects of ECMO in most studies involving trauma patients who did not respond to conventional therapy and are in a life-threatening condition, the panel still recommend ECMO be used in adult trauma patients with refractory acute cardiopulmonary failure caused by varied mechanisms of injury, with confidence that further research is unlikely to change the estimate of the effect. Nonetheless, in deciding to implement trauma ECMO, clinicians should fully consider the reversibility of injury, efficacy of decisive therapy, patient’s preference, and long-term outcomes, not just based on the underlying injury mechanisms. For the TCA patients, the risk of death remains higher even with the return of spontaneous circulation; thus, it is imperative to closely monitor the patients’ hemodynamics, once unstable, timely initiation of VA-ECMO might improve the likelihood of survival. The contraindications to trauma ECMO include poor neurological prognosis, irreversible injury, and uncontrollable bleeding with profound coagulopathy.

## Domain 2. Patient screening

*Recommendation 5*. We suggest using the ISS as an adjunctive screening tool for determining the ECMO candidates and treatment success in adult trauma patients with acute cardiopulmonary failure refractory to conventional therapy (weak recommendation, very low-quality evidence for both candidate and success).

### Rationale

***Evidence summary***. Thirty-five retrospective studies were enrolled in this domain. The subgroup meta-analyses are shown in Supplementary material: Figures S31–S42. ECMO-supported adult trauma patients had a higher ISS at admission than those unexposed to ECMO, with a pooled ISS of 34.17 (95% CI: 31.03–37.3; 35 studies, 1421 patients) and 26.77 (95% CI: 24.14–29.41; 10 studies, 77,183 patients), respectively. The ECMO survivors had a relatively lower ISS than the non-survivors, with a pooled ISS of 30.36 (95% CI: 26.62–34.11) and 37.65 (95% CI: 31.87–43.43), respectively, and the SMD was 0.52 (95% CI: 0.19–0.84, *P* = 0.002; 14 studies, 989 patients). A retrospective analysis of the Premier Healthcare Database (2016–2020) comprising 118 adult trauma patients on ECMO and 59,612 patients unexposed to ECMO revealed that ISS was significantly related to ECMO utilization (*OR* 1.03, 95% CI: 1.01–1.04, *P* < 0.01) [9]. In another retrospective analysis of ECMO-supported trauma patients (453 survivors and 245 non-survivors) from the Trauma Quality Improvement Program database (2010–2019), ISS was independently associated with in-hospital mortality (*OR* 1.02, 95% CI: 1.01–1.03, *P* < 0.01) [23]. Nonetheless, an ISS of > 63 points predicted ECMO unsuitability and failure (*OR* 4.27, 95% CI, 1.37–13.31; *P* < 0.04) [44].

***Justification and implementation considerations***. Although a variety of baseline variables were used to predict ECMO utilization and treatment success in severely injured patients, including age, sex, abbreviated injury scale score, and blood lactate and oxygenation index, our meta-analyses demonstrated that ISS might be independently associated with trauma ECMO use and treatment success. A higher ISS of > 30 points indicates that the patients have more serious and extensive injury and are more likely to develop refractory cardiopulmonary failure, whereas a very high ISS score denotes a poor chance of survival despite ECMO use. Although the ISS prediction validity has not been fully verified, given the simplicity, objectivity, and accessibility of the ISS scoring system [1], the panel issued a weak recommendation on ISS use as an adjunctive screening tool for ECMO candidate and treatment success, thereby facilitating early emergency warning, medical staff assembling, and logistical arrangement. In the case where the ISS exceeds an arbitrary threshold of 30 points, the risks of failure, cost-effectiveness, and the patients’ value and preference should be prudently considered when deciding to cannulate the patient or not.

## Domain 3. Timing of initiation

*Recommendation 6.* For adult trauma patients with acute cardiopulmonary failure refractory to conventional therapy, we recommend that ECMO be initiated as early as possible provided that the initiation criteria are met (strong recommendation, very low-quality evidence).

### Rationale

***Evidence summary***. Twenty-four retrospective studies were enrolled in this domain for meta-analyses, as showed in Supplementary material: Figures S43–S50. For adult trauma patients, early death (≤ 5 days) is largely due to TCA and hemorrhagic shock, whereas ARDS and irreversible brain injury are often the causes of late death [18, 39]. In eight studies (122 patients), the time from injury to cannulation was within 5 days, and the pooled survival rate was 66% (95% CI: 58–74%), whereas that of patients with a time interval of > 5 days (17 patients, 3 studies) was 38% (95% CI: 13–71%). Nine studies (197 patients) reported a time interval of < 5 days from admission to ECMO, with a pooled overall survival of 58% (95% CI: 39–75%), whereas that of patients with a time interval of > 5 days (12 patients, one study) was 42% (95% CI: 15–72%). In a single-center retrospective cohort study involving 36 TBI patients on VV-ECMO, despite a low admission Glasgow Coma Scale score, those with cannulation within 3 h of developing hypoxia had a higher survival rate and showed good neurological outcomes (*OR* 2.08, 95% CI: 1.34–2.35, *P* < 0.001) [40]. Additionally, three studies (88 trauma patients) reported the durations on ventilator prior to ECMO, with 3.71 (95% CI: 3.28–4.15) for survivors and 5.04 (95% CI: 0.05–10.04) days for non-survivors, and the SMD was − 1.70 (95% CI: −5.39 to 1.98, *P* = 0.37).

***Justification and implementation considerations.*** VV-ECMO is recommended by the ELSO guideline for non-trauma patients if the conventional therapy is ineffective and meets the following criteria: (1) PaO_2_/FiO_2_ < 80 mmHg or (2) severe hypercapnia (pH < 7.25 with a PaCO_2_ ≥ 60 mmHg) [15]. For VA-ECMO, the following parameters were considered: (1) systolic blood pressure < 90 mmHg; (2) urine output < 30 ml/h; (3) lactate ≥ 2 mmol/L; (4) S_V_O_2_ < 60%; and (5) altered consciousness ≥ 6 h unresponsive to optimal resuscitation [16]. In our meta-analyses, for adult trauma patients with worsening heart and lung injuries, initiation of ECMO within 5 days following trauma might confer survival benefits, whereas the delays in its initiation as a salvage treatment while attempting conventional management worsen the outcomes. Although the panel failed to determine the exact indicators for ECMO initiation following trauma, e.g., hypoxia duration despite of optimal therapies, most studies followed the ELSO criteria of ECMO initiation. Considering the large effect between the early and delayed ECMO on patient survival, the panel strongly recommends ECMO use as a rescue therapy as early as possible in case of the ELSO criteria are met, especially in those with extensive and worsening injuries. Bedsides, in patients with prolonged ventilator use duration of > 5 days, the pros and cons should be carefully considered before ECMO initiation.

## Domain 4. Multidisciplinary approach

*Recommendation 7.* We recommend that a multidisciplinary team and quality improvement program be established in the management of adult trauma patients requiring ECMO (strong recommendation, very low-quality evidence).

### Rationale

***Evidence summary***. The multidisciplinary approach to ECMO management should include an interdisciplinary team and quality improvement programs. Eighteen retrospective studies involving 357 ECMO-supported adult trauma patients reported the use of a multidisciplinary method in trauma ECMO management, with a pooled overall survival rate of 66% (95% CI: 60–70%), whereas that of patients without the intervention was 62% (95% CI: 54–69%; three studies, 153 patients), see Supplementary material: Figure S51–S52. In a retrospective review of the National Trauma Data Bank (2007–2015) involving 5,781,123 adult trauma patients, those admitted to trauma centers with ECMO capability were more likely to undergo multidisciplinary decisive surgeries and have a significantly decreased *OR* for mortality compared with admission to a non-ECMO facility (adjusted *OR*: 0.96, 95% CI: 0.95–0.97; adjusted *P* < 0.001) [80].

***Justification and implementation considerations***. Although our meta-analyses failed to identify a difference in the survival rates between ECMO-supported adult trauma patients with and without an interdisciplinary intervention, most of the studies were conducted in large-volume ECMO facilities or level I trauma centers, and multidisciplinary approaches might have been in place [76]. Interdisciplinary coordination among the emergency physicians, intensivists, trauma surgeons, and well-trained staff is essential for ECMO success, including a quick response to emergency consultation [73], suitable patient selection [37, 40], rapid cannula insertion, definitive surgery, and safe patient transport [11, 95]. The quality improvement program should include an efficient institutional structure responsible for the operation of the trauma ECMO center, standard procedures for trauma ECMO management, and quality control standards [96]. Meanwhile, a regional trauma network around an ECMO-capable level I trauma center should be encouraged to maximize the access of patients to an ECMO expert center. The ECMO transfer criteria include a PaO_2_/FiO_2_ ratio of ≤ 100 mmHg, without absolute contraindications to ECMO [15].

## Domain 5. Trauma ECMO management

*Recommendation 8.* For adult trauma patients treated with ECMO, we suggest minimal anticoagulation strategy be used to achieve individualized systemic anticoagulation goal (weak recommendation, very low-quality evidence).

### Rationale

***Evidence summary***. Forty-nine retrospective studies involving 990 ECMO-supported adult trauma patients reported anticoagulant strategies, see Supplementary material: Table S8. Narratively, unfractionated heparin was the most used anticoagulant, and Nafamstat was administered as a substitute in patients with heparin intolerance [47, 52]. If bleeding is a serious concern, systemic heparinization could be withheld for 48 h up to 5 days, or a pre-cannula loading dose of 50 IU/kg heparin could be administered without continuous infusion. Additionally, using a heparin-coated circuit with an ECMO blood flow of 3–5 L/min reduced the frequency of anticoagulant use and clotting time, while maintaining the platelet count above 80 × 10^9^/L as well as normal serum levels of clotting factors avoided the occurrence of bleeding [45, 71, 72, 74]. During trauma ECMO, the activated partial thromboplastin time and activated clotting time (ACT) were typically maintained at 45–60 and 150–200 s, respectively, and the antithrombin III activity, when available, was maintained at 80–100% to ensure the efficacy of heparin and prevent bleeding [72, 86]. The thromboelastogram (TEG) reflects the whole profile of blood coagulation, and its early use as a monitoring tool has led to more targeted anticoagulation management [30].

***Justification and implementation considerations***. The ELSO guideline recommends that systemic heparinization with an ACT of 180–220 s be applied to non-trauma patients requiring ECMO [97]. However, for trauma patients on ECMO, the anticoagulant dosage, target, and treatment course remain unclear. In the past decades, the advances in anticoagulant methods and devices, including the centrifugal pump, methylpentene membrane oxygenator, and heparin-coated circuit, have resulted in more choices of anticoagulant strategies and less anticoagulation-related complications in trauma patients undergoing ECMO [14, 73]. Based on clinical experience and expert opinions, the panel conditionally suggested that minimum systemic anticoagulation with individualized target be applied to trauma patients on ECMO. Nonetheless, in deciding to implement trauma ECMO anticoagulation, the severity of injuries and risks of bleeding and thrombosis should be fully considered. In settings where point-of-care coagulation tests or TEG are not readily available, close monitoring of the patients’ vital signs, bleeding at the injured regions, limb ischemia, and circuit clotting is required. However, rapid damage control and surgical hemostasis are prerequisites before performing systemic heparinization.

*Recommendation 9.* For adult trauma patients with massive bleeding or hemorrhagic shock, we suggest that coagulopathy, hypothermia, and acidosis be closely managed during ECMO treatment (best practice statement).

### Rationale

***Evidence summary***. Trauma-induced coagulopathy increased the mortality risk of patients by almost five times [98], and by four or seven times in those with a body core temperature of < 35 °C (*OR* 3.95, 95% CI: 2.90–5.4) or pH < 7.16 (*OR* 7.3, 95% CI: 1.39–38.11) [41, 99]. Over the past decades, the DCR strategy, focusing on rapid bleeding control, maintaining core temperatures between 36 °C and 37 °C, and correcting acidosis, has become a landmark in trauma care and significantly decreased the incidence of traumatic lethal triad and subsequent death [100]. For trauma patients with deteriorated heart and lung dysfunction despite maximal DCR, ECMO support provides additional advantages, including extended damage control for severe injuries, circulatory support, and rewarming in those with hypothermia, thereby interrupting the vicious cycle of the traumatic lethal triad and conferring potential survival benefits without significant complications [59, 62].

Several trauma guidelines have suggested the use of the threshold of prothrombin time (PT)/international standardized ratio (INR) of > 1.5 or clot strength maximum amplitude of TEG of > 66.7 mm to early detect trauma-induced coagulopathy [101, 102]. A large retrospective study has demonstrated that the early use of thromboelastometry (ROTEM) led to less than the expected mortality rate [103]. However, ECMO anticoagulation may affect the reliability of laboratory tests; in this case, the ISS showed potential as an alternative indicator for predicting the occurrence of trauma-induced coagulopathy. An ISS of > 15 denoted that 24.4% of trauma patients had significant coagulopathy, and up to 61.7% if ISS exceeded 45, even 100% when ISS over 60 points [98]. Besides, blood lactate could be used to estimate the extent of bleeding and tissue hypoperfusion during trauma ECMO, with its rapid reduction and normalization indicating a response to damage control resuscitation and survival of the patient [37].

***Justification and implementation considerations***. Massive bleeding or hemorrhagic shock is among the primary causes of early death in trauma patients, which may create the traumatic lethal triad in terms of coagulopathy, hypothermia, and acidosis, significantly increasing the mortality risk of patients with massive but potentially survivable injuries. With the advances in anticoagulant strategies and devices, trauma-induced coagulopathy is no longer an absolute contraindication of ECMO, which in turn may provide survival benefits for trauma patients who failed to respond to maximal DCR. Although insufficient data and inherent defects, such as the retrospective nature, high risk of bias, and heterogeneity across the studies, hindered further analyses, considering the high mortality risk of patients with the traumatic lethal triad and the serious concern about the risk of bleeding by physicians and surgeons who are responsible for ECMO management and decisive surgery, the panel issued a best practice statement on the close monitoring and management of patients with a higher ISS or suspected trauma-induced coagulopathy during ECMO therapy. However, in the context of ECMO therapy, the diagnostic indices for coagulopathy should be treated with caution, and the clinical observation, severity of injury, and physician’s judgment are still of paramount importance in the early detection of the traumatic lethal triad.

*Recommendation 10.* We suggest that bedside ultrasound be used to optimize fluid management in adult trauma patients requiring ECMO (weak recommendation, very low-quality evidence).

### Rationale

***Evidence summary***. Positive fluid balance independently predicted in-hospital and 90-day mortality in ECMO-supported patients, with hazard ratios of 2.21 (95% CI: 1.10–4.41, *P* < 0.05) and 2.61 (95% CI: 1.30–5.23, *P* < 0.05) [104], respectively. However, massive fluid resuscitation following ECMO initiation are needed in trauma patients with hemorrhagic shock for hemodynamic stability and adequate oxygen delivery. In this situation, the principle of early fluid resuscitation guided by hemodynamics and late fluid removal should be followed to improve the patient’s prognosis [102]. However, during ECMO therapy, traditional hemodynamic monitoring, e.g., the transpulmonary thermodilution method, may be ineffective, whereas bedside ultrasound is a preferred visual imaging method for evaluating volume status and fluid responsiveness [105, 106]. A ratio of left and right ventricle end-diastolic area of > 0.6, inferior vena cava (IVC) diameter of > 2.5 cm, and B-lines predominance on lung ultrasound prompt an overloaded volume state that is unlikely to respond to further fluid administration. Although a typical sign of “kissing ventricles,” velocity time integral variability of the left ventricular outflow tract of 12–14%, and IVC variability of > 12% predict fluid responsiveness [107, 108]. Moreover, Extended-Focused Assessment with Sonography for Trauma (E-FAST) is conductive to detect the unidentified bleeding source and etiology of hypovolemia [109]. A large Cochrane review found that the pooled sensitivity and specificity of the bedside ultrasound in detecting hemopneumothorax, hemopericardium, or free abdominal fluid were 0.78 (95% CI: 0.69–0.84) and 0.97 (95% CI: 0.96–0.99) in an adult or mixed population [110].

***Justification and implementation considerations***. Over the last decade, bedside ultrasound has gained wide acceptance within the critical care society due to its non-invasiveness, repeatability, and feasibility. However, few studies have been conducted to evaluate ultrasound-guided fluid management for trauma patients with or without ECMO. Considering the indispensable roles of bedside ultrasound in ECMO management, namely, pre-, during, and post-cannula, especially in management of ECMO blood flow and etiology identification of hypovolemia, as well as the patients’ value and preference, the panel suggest bedside ultrasound be used for fluid management in adult trauma patients requiring ECMO. However, due to the known limitations of ultrasound examination, e.g., considerable operator-dependency, clinicians should not use the bedside ultrasound alone for decision-making instead of clinical evaluation.

## Domain 6. Complications

*Recommendation 11.* We suggest that effective measures be taken to reduce the ECMO-related complications in adult trauma patients requiring ECMO (weak recommendation, very low-quality evidence).

### Rationale

***Evidence summary***. Thirty-four retrospective studies involving 675 ECMO-supported adult trauma patients reported anticoagulation-related complications. Hemorrhage and thrombosis occurred in 19.7% and 18.5% of the patients, of which 4% had hemorrhagic/ischemic stroke, see Supplementary material: Table S9. In eight retrospective studies involving 78 ECMO-supported adult patients with or without TBI, thrombosis occurred in 25.0% and 10.9%, hemorrhage in 15.6% and 23.9%, and stroke in 9.4% and 6.5% of the two cohorts, respectively. Three retrospective cohort studies (1453 patients) have compared the incidence of complications between adult trauma patients with and without ECMO. Acute renal injury was the most frequent complication, occurring in 42.1% and 19.6% of the two cohorts, followed by sepsis in 26.3% and 15.6%, thrombosis in 22.3% and 14.2%, and bleeding in 3.6% and 0.29%, respectively, see Supplementary material: Table S10-S11. Additionally, a retrospective analysis of the ELSO registry (1999–2015) involving 58 ECMO-supported adult patients with burn injury revealed infections (36.2%) and renal failure (34.5%) as the most common complications, with no significant differences observed between the survivors and non-survivors [41]. In two large retrospective analyses of traumatic ARDS patients on VV-ECMO, although the ECMO–related complications were more frequent in these patients as compared to those unexposed to ECMO, systemic heparinization was not associated with the risk of bleeding and death [38, 47].

***Justification and implementation considerations***. ECMO and anticoagulation-related complications are of great concern to clinicians in managing trauma patients receiving ECMO. Although the prevalence seems to be higher in ECMO-supported patients, the rates of clinically important adverse events, e.g., massive intracranial hemorrhage and neurological disability, are comparable to those of trauma patients without ECMO. Given that the survival benefits of ECMO might come at the expense of a potential increase in complications [85], the panel conditionally suggested that effective measures be taken to prevent ECMO-related complications, including immediate damage control, close monitoring of anticoagulation, and implementing ECMO in large-volume and experienced trauma centers, which is expected to further reduce the occurrence of complications [54, 84].

## Discussion

To the best of our knowledge, this is the first clinical practice guideline on the application of ECMO in adult trauma patients with refractory cardiopulmonary failure, primarily applied to medical settings with ECMO capability. The panel strived to collect the available evidence across domestic and abroad to improve the applicability and generality of the guideline, with the aim of improving the prognosis of severely injured adult patients at high risk of death.

Although the panel recommend ECMO use as a rescue therapy rather than as a salvage therapy for adult trauma patients with refractory cardiopulmonary failure, it should be noted that the guideline only addresses questions that the experts are most concerned about. For the other issues regarding trauma ECMO use, e.g., cannulation, lung protective ventilation, nutrition, patient transportation, and staff training, the ELSO serial guidelines for non-trauma ECMO can be used as reference, which are theoretically applicable to the trauma patient population. Moreover, the guideline recommendations are not substitutes for the healthcare provider’s professional judgments, and clinicians should use their expertise to determine the use of trauma ECMO that is consistent with the patient’s value and preference, equity, and human rights.

This guideline is associated with several limitations. First, due to the nature of trauma ECMO therapy, there is a paucity of prospective research in this field, as most studies are retrospective, small sample–sized, and even case series, with moderate to high risk of bias and heterogeneity, resulting in low to very low-quality of evidence. Second, the role of VA-ECMO in TCA and cardiogenic shock needs to be further determined. Most studies involved a small number of cases, even sporadic, without describing the characteristics of the patients and the criteria for VA-ECMO initiation, which precluded the synthesis of evidence supporting its application to those most likely to benefit. Third, owing to insufficient data and wide variation in clinical practice across the trauma ECMO centers, the panel was unable to propose standard schemes for the minimum anticoagulation strategy, traumatic lethal triad prediction, ultrasound-guided fluid management, and preventive measures of ECMO-related complications. The recommendations largely depended on expert experience and opinions, and its reliability and validity need to be further confirmed. Fourth, the long-term outcomes and quality of life of trauma ECMO survivors should be further determined, especially whether the improvement of survival comes at the expense of developing organic and cognitive disabilities among patients, as this has an impact on the generalization of trauma ECMO and patient preference. Therefore, well-designed clinical trials or large-sample prospective studies are needed to provide vigorous evidence supporting ECMO use in adult trauma patients with refractory acute cardiopulmonary failure, in accordance with the individual characteristics of these patients with varied injury mechanisms.

## Electronic supplementary material

Supplementary Material 1. Table S1. Summary of literature retrieval. Table S2. Literature retrieval strategy. Figure S1. Flow diagram of study inclusion. Table S3. Study characteristics. Table S4. Newcastle-Ottawa Quality Assessment Form for Cohort Studies. Table S5. JBI’s tool for assessing case series. Table S6. CASP-case-control-study-checklist. Table S7. Evaluation of the certainty of evidence. Figure S2-30. Subgroup meta-analyses in Domain 1. Figure S31-42. Subgroup meta-analyses in Domain 2. Figure S43-50. Subgroup meta-analyses in Domain 3. Figure S51-52. Subgroup meta-analyses in Domain 4. Table S8. Summary of anticoagulation strategy. Table S9. Statistics of complications. Table S10. Characteristics of the studies on ECMO-related complications. Table S11. Characteristics of the studies on ECMO-related complications in adult trauma patients with or without TBI. Table S12. Evidence to Decision Framework

## Data Availability

The data that support the findings of this guideline are available at http://www.guidelines-registry.org.

## Abbreviations

ACT: Activated clotting time
ARDS: Acute respiratory distress syndrome
CI: Confidence interval
CSECLS: The Chinese Society of Extracorporeal Life Support
DCR: Damage control resuscitation
ECMO: Extracorporeal membrane oxygenation
E-FAST: Extended-Focused Assessment with Sonography for Trauma
ELSO: Extracorporeal life support organization
GRADE: The Grading of Recommendations Assessment, Development and Evaluation
ICU: Intensive care unit
ISS: Injury severity score
INR: International standardized ratio
IVC: Inferior vena cava
LOS: Length of stay
OR: Odds ratio
PaCO_2_: Partial pressure of carbon dioxide in arterial blood
PaO_2_/FiO_2_: Pressure of arterial oxygen to fractional inspired oxygen concentration ratio
PICO: Population, Intervention, Comparison, and Outcome
PT: Prothrombin time
ROTEM: Rotational thromboelastometry
SVO_2_: Mixed venous oxygen saturation
TBI: Traumatic brain injury
TCA: Traumatic cardiac arrest
TEG: Thromboelastography
US: Ultrasound
VV-ECMO: Venovenous extracorporeal membrane oxygenation
VA-ECMO: Venoarterial extracorporeal membrane oxygenation
WHO: World Health Organization
