# Supplementary material for: Use of extracorporeal membrane oxygenation in adult trauma patients with refractory acute cardiopulmonary failure: guideline from the Chinese society of extracorporeal life support 2025
Source: Crit Care. 2025 Jul 29;29:334. doi: 10.1186/s13054-025-05504-6 (PMC12308958; doi:10.1186/s13054-025-05504-6)
Supplement: Supplementary file 1 — Supplementary Material 1. Table S1. Summary of literature retrieval. Table S2. Literature retrieval strategy. Figure S1. Flow diagram of study inclusion. Table S3. Study characteristics. Table S4. Newcastle-Ottawa Quality Assessment Form for Cohort Studies. Table S5. JBI’s tool for assessing case series. Table S6. CASP-case-control-study-checklist. Table S7. Evaluation of the certainty of evidence. Figure S2-30. Subgroup meta-analyses in Domain 1. Figure S31-42. Subgroup meta-analyses in Domain 2. Figure S43-50. Subgroup meta-analyses in Domain 3. Figure S51-52. Subgroup meta-analyses in Domain 4. Table S8. Summary of anticoagulation strategy. Table S9. Statistics of complications. Table S10. Characteristics of the studies on ECMO-related complications. Table S11. Characteristics of the studies on ECMO-related complications in adult trauma patients with or without TBI. Table S12. Evidence to Decision Framework [file 13054_2025_5504_MOESM1_ESM.docx]

**Title**

**Use of Extracorporeal Membrane Oxygenation in Adult Trauma Patients with Refractory Acute Cardiopulmonary Failure – Guideline from the Chinese Society of Extracorporeal Life Support**

**2025**

**Supplementary material**

**Content**

**Table S1.** Summary of literature retrieval.........................................................................................................3

**Table S2.** Literature retrieval strategy...............................................................................................................4

**Figure S1.** Flow diagram of study inclusion...................................................................................................17

**Table S3.** Study characteristics .......................................................................................................................18

**Table S4.** Newcastle-Ottawa Quality Assessment Form for Cohort Studies...................................................32

**Table S5.** JBI’s tool for assessing case series..................................................................................................35

**Table S6.** CASP-case-control-study-checklist.................................................................................................40

**Table S7.** Evaluation of the certainty of evidence...........................................................................................45

**Figure S2-30.** Domain 1 subgroup meta-analyses.......................................................................................... 47

**Figure S31-42.** Domain 2 subgroup meta-analyses ....................................................................................... 62

**Figure S43-50.** Domain 3 subgroup meta-analyses.........................................................................................67

**Figure S51-52.** Domain 4 subgroup meta-analyses ....................................................................................... 71

**Table S8.** Summary of anticoagulation strategy ........................................................................................... 73

**Table S9.** Statistics of complications ............................................................................................................. 98

**Table S10.** Characteristics of studies on ECMO related complications ...................................................... 104

**Table S11.** Characteristics of studies on ECMO-related complications in adult trauma patients with or without TBI .................................................................................................................................................. 105

**Table S12.** Evidence to Decision Framework .............................................................................................. 107

**Table S1. Summary of literature retrieval by the category of questions**

| **Database** | **Questions** | | | | | | | |
| --- | --- | --- | --- | --- | --- | --- | --- | --- |
|  | Indication (Q1) | Patient screening (Q2) | Timing (Q3) | Multidisciplinary (Q4) | Anti-coagulation (Q5) | Lethal triad (Q6) | Ultrasound (Q7) | Complication (Q8) |
| PubMed | 1758 | 177 | 538 | 878 | 304 | 302 | 295 | 645 |
| Embase | 3125 | 321 | 279 | 1859 | 144 | 177 | 209 | 1393 |
| Cochrane | 264 | 89 | 38 | 187 | 7 | 18 | 83 | 64 |
| CNKI | 320 | 82 | 204 | 133 | 67 | 65 | 38 | 110 |
| VIP | 247 | 3 | 107 | 5 | 30 | 31 | 18 | 51 |
| Wangfang | 216 | 217 | 155 | 269 | 36 | 30 | 23 | 63 |
| SinoMed | 646 | 59 | 484 | 144 | 70 | 81 | 160 | 254 |
| Medlive.cn | 135 |  |  |  |  |  |  |  |
| Total | 3937 | 578 | 1766 | 3463 | 658 | 734 | 845 | 2583 |

ISS: injury severity score; CNKI: China National Knowledge Infrastructure; VIP: VIP Information (Weipu database in Chinese).

**Table S2. Literature retrieval strategy by the category of questions.**

| **Search strategy of question 1: Indications** |
| --- |
| **PubMed** |
| (("trauma*"[Title/Abstract] OR "injur*"[Title/Abstract] OR "wound*"[Title/Abstract] OR "burn*"[Title/Abstract] OR "Scald"[Title/Abstract]) AND ("extracorporeal membrane oxygenation*"[Title/Abstract] OR "membrane oxygenation extracorporeal"[Title/Abstract] OR "oxygenation extracorporeal membrane"[Title/Abstract] OR "ECMO"[Title/Abstract] OR "ECLS"[Title/Abstract] OR "extracorporeal life support*"[Title/Abstract] OR "life support extracorporeal"[Title/Abstract]) AND ("thora*"[Title/Abstract] OR ("Chest*OR"[All Fields] AND "airway"[Title/Abstract]) OR "trache*"[Title/Abstract] OR "Intratracheal"[Title/Abstract] OR "pulmon*"[Title/ Abstract] OR "protective ventilation"[Title/Abstract] OR "head*"[Title/Abstract] OR "Craniocerebral" [Title/Abstract] OR "Parietal"[Title/Abstract] OR "skull*"[Title/Abstract] OR "Cranium"[Title/Abstract] OR "Calvaria"[Title/Abstract] OR "Calvarium"[Title/Abstract] OR "cerebr*"[Title/Abstract] OR "Occipital" [Title/Abstract] OR "Temporal"[Title/Abstract] OR "Frontal"[Title/Abstract] OR "Forehead"[Title/Abstract] OR "respiratory distress syndrome"[Title/Abstract] OR "ARDS"[Title/Abstract] OR "Hypoxemia"[Title/Abstract] OR "Anoxia"[Title/Abstract] OR "Anoxemia"[Title/Abstract] OR ("Oxygen"[Title/Abstract] AND "deficienc*" [Title/Abstract]) OR (("heart*"[Title/Abstract] OR "cardi*"[Title/Abstract]) AND ("surg*"[Title/Abstract] OR "operat*"[Title/Abstract])) OR "asystol*"[Title/Abstract] OR "Initiate"[Title/Abstract] OR "Commence" [Title/Abstract] OR "Institute"[Title/Abstract] OR "Induce"[Title/Abstract] OR "Activate"[Title/Abstract] OR "Deploy"[Title/Abstract] OR "Commission"[Title/Abstract] OR "start"[Title/Abstract] OR "begin"[Title/Abstract] OR "assess*"[Title/Abstract] OR "Evaluate"[Title/Abstract] OR "Appraise"[Title/Abstract] OR "Gauge" [Title/Abstract] OR "Measure"[Title/Abstract] OR "trauma severity indices"[Title/Abstract] OR "abbreviated injury scale"[Title/Abstract] OR "glasgow coma scale"[Title/Abstract] OR "glasgow outcome scale" [Title/ Abstract] OR "injury severity score"[Title/Abstract] OR "ISS score"[Title/Abstract] OR "Severity" [Title/Abstract] OR "age"[Title/Abstract] OR "complication"[Title/Abstract] OR "prognos*"[Title/Abstract] OR "medical resourc*"[Title/Abstract] OR "Metaethics"[Title/Abstract] OR "ethic*"[Title/Abstract] OR "law" [Title/Abstract] OR "moral"[Title/Abstract])) NOT ("children"[Title/Abstract] OR "infant"[Title/Abstract] OR "child"[Title/ Abstract] OR "pediatric"[Title/Abstract] OR "neonatal"[Title/Abstract] OR "baby"[Title/Abstract] OR "neonat" [Title/Abstract] OR "newborn"[Title/Abstract] OR "porcine"[Title/Abstract] OR "mouse"[Title/Abstract] OR "mice"[Title/Abstract] OR "rabbit"[Title/Abstract] OR "pigs"[Title/Abstract])  The search was conducted from the inception of the library to February 25, 2024, and **1758** articles were retrieved. |
| **Embase** |
| (trauma*:ti,ab OR injur*:ti,ab OR wound*:ti,ab OR burn*:ti,ab OR scald:ti,ab) AND (extracorporeal:ti,ab AND membrane:ti,ab AND oxygenation*:ti,ab OR (membrane:ti,ab AND oxygenation,:ti,ab AND extracorporeal:ti,ab) OR (oxygenation,:ti,ab AND extracorporeal:ti,ab AND membrane:ti,ab) OR ecmo:ti,ab OR ecls:ti,ab OR (extracorporeal:ti,ab AND life:ti,ab AND support*:ti,ab) OR (life:ti,ab AND support,:ti,ab AND extracorporeal:ti,ab)) AND (((thora*:ti,ab OR chest*or:ti,ab) AND airway:ti,ab OR trache*:ti,ab OR intratracheal:ti,ab OR pulmon*:ti,ab OR (protective:ti,ab AND ventilation:ti,ab) OR head*:ti,ab OR craniocerebral:ti,ab OR parietal:ti,ab OR skull*:ti,ab OR cranium:ti,ab OR calvaria:ti,ab OR calvarium:ti,ab OR cerebr*:ti,ab OR occipital:ti,ab OR temporal:ti,ab OR frontal:ti,ab OR forehead:ti,ab OR respiratory:ti,ab) AND distress:ti,ab AND syndrome:ti,ab OR ards:ti,ab OR hypoxemia:ti,ab OR anoxia:ti,ab OR anoxemia:ti,ab OR (oxygen:ti,ab AND deficienc*:ti,ab) OR ((heart*:ti,ab OR cardi*:ti,ab) AND (surg*:ti,ab OR operat*:ti,ab)) OR asystol*:ti,ab OR initiate:ti,ab OR commence:ti,ab OR institute:ti,ab OR induce:ti,ab OR activate:ti,ab OR deploy:ti,ab OR commission:ti,ab OR start:ti,ab OR begin:ti,ab OR assess*:ti,ab OR evaluate:ti,ab OR appraise:ti,ab OR gauge:ti,ab OR measure:ti,ab OR (trauma:ti,ab AND severity:ti,ab AND indices:ti,ab) OR (abbreviated:ti,ab AND injury:ti,ab AND scale:ti,ab) OR (glasgow:ti,ab AND coma:ti,ab AND scale:ti,ab) OR (glasgow:ti,ab AND outcome:ti,ab AND scale:ti,ab) OR (injury:ti,ab AND severity:ti,ab AND score:ti,ab) OR 'iss score':ti,ab OR severity:ti,ab OR 'age':ti,ab OR complication:ti,ab OR prognos*:ti,ab OR (medical:ti,ab AND resourc*:ti,ab) OR metaethics:ti,ab OR ethic*:ti,ab OR 'law':ti,ab OR moral:ti,ab) NOT (children:ti,ab OR infant:ti,ab OR child:ti,ab OR pediatric:ti,ab OR neonatal:ti,ab OR baby:ti,ab OR neonat:ti,ab OR newborn:ti,ab OR porcine:ti,ab OR mouse:ti,ab OR mice:ti,ab OR rabbit:ti,ab OR pigs:ti,ab)  The search was conducted from the inception of the library to February 25, 2024, and **3125** articles were retrieved. |
| **The Cochrane Library** |
| (Trauma* OR Injur* OR Wound* OR burn* OR Scald) AND ((Extracorporeal Membrane Oxygenation*) OR (Membrane Oxygenation, Extracorporeal) OR (Oxygenation, Extracorporeal Membrane) OR ECMO OR ECLS OR (Extracorporeal Life Support*) OR (Life Support, Extracorporeal)) AND (Thora* OR Chest*OR airway OR Trache* OR Intratracheal OR pulmon* OR (protective ventilation) OR Head* OR Craniocerebral OR Parietal OR Skull* OR Cranium OR Calvaria OR Calvarium OR Cerebr* OR Occipital OR Temporal OR Frontal OR Forehead OR Respiratory Distress Syndrome OR ARDS OR Hypoxemia OR Anoxia OR Anoxemia OR (Oxygen AND Deficienc*) OR Heart* OR cardi* OR Asystol* OR Initiate OR Commence OR Institute OR Induce OR Activate OR Deploy OR Commission OR start OR begin OR Assess* OR Evaluate OR Appraise OR Gauge OR Measure OR (Trauma Severity Indices) OR (Abbreviated Injury Scale) OR (Glasgow Coma Scale) OR (Glasgow Outcome Scale) OR (Injury Severity Score) OR "ISS score" OR severity OR "age" OR complication OR prognos* OR (medical resourc*) OR Metaethics OR Ethic* OR "law" OR moral) NOT (children OR infant OR child OR pediatric OR neonatal OR baby OR neonat OR newborn OR porcine OR mouse OR mice OR rabbit OR pigs)  The search was conducted from the establishment of the library to February 22, 2024, and **264** articles were retrieved. |
| **China National Knowledge Infrastructure** |
| (TKA%=体外模式氧合 + 体外膜式氧合 + 体外膜肺 + 膜肺氧合 + 体外膜氧合 + 体外生命支持 + ECMO + ECLS) AND (TKA%=创伤 + 坠落 + 车祸 + 多发伤 + 烧伤 + 钝挫 + 贯通 + 震爆 + 撞击 + 刺伤 + 切割 + 挫伤 + 烫伤 + 撕裂伤 + 挤压 + 火器伤 + 电击) AND (TKA%=颅 + 脑 + 头 + 胸 + 肺 + 气道 + 气管 + 吸入性损伤 + 低氧血症 + ARDS + 急性呼吸窘迫综合征 + 成人型呼吸窘迫综合征 + 急性呼吸窘迫综合症 + 急性呼吸窘迫症 + 急性呼吸窘迫征 + 成人呼吸窘迫综合征 + 肺保护通气 + 保护性肺通气 + 保护性通气 + 创伤性湿肺 + 心 + 启动 + 开始 + 评估 + 严重程度 + 器官损害 + 年龄 + 合并症 + 预后 + 医疗资源 + 器官捐献 + 伦理)  The search was conducted from the establishment of the library to February 21, 2024, and **320** articles were retrieved. |
| **Wanfang Database** |
| 主题:(ecmo or "体外膜肺" or "体外模式氧合" or "体外膜式氧合" or "膜肺氧合" or "体外膜氧合" or "体外生命支持" or ECLS) and 主题:(创伤 or 坠落 or 车祸 or 多发伤 or 烧伤 or 钝挫 or 贯通 or 震爆 or 撞击 or 刺伤 or 切割 or 挫伤 or 烫伤 or 撕裂伤 or 挤压 or 火器伤 or 电击) and 主题:(颅 or 脑 or 头 or 胸 or 肺 or 气道 or 气管 or 吸入性损伤 or 低氧血症 or ARDS or 急性呼吸窘迫综合征 or 成人型呼吸窘迫综合征 or 急性呼吸窘迫综合症 or 急性呼吸窘迫症 or 急性呼吸窘迫征 or 成人呼吸窘迫综合征 or 肺保护通气 or 保护性肺通气 or 保护性通气 or 创伤性湿肺 or 心 or 启动 or 开始 or 评估 or 严重程度 or 器官损害 or 年龄 or 合并症 or 预后 or 医疗资源 or 器官捐献 or 伦理)  The search was conducted from the inception of the library to February 21, 2024, and **216** articles were retrieved. |
| **VIP Information (Weipu database in Chinese)** |
| (U= ecmo OR 体外膜肺 OR 体外模式氧合 OR 体外膜式氧合 OR 膜肺氧合 OR 体外膜氧合 OR 体外生命支持 OR ECLS) AND (U=创伤 OR 坠落 OR 车祸 OR 多发伤 OR 烧伤 OR 钝挫 OR 贯通 OR 震爆 OR 撞击 OR 刺伤 OR 切割 OR 挫伤 OR 烫伤 OR 撕裂伤 OR 挤压 OR 火器伤 OR 电击)  The search was conducted from the inception of the library to February 22, 2024, and **247** articles were retrieved. |
| **SinoMed** |
| ( "ecmo"[常用字段:智能] OR "体外膜肺"[常用字段:智能] OR "体外模式氧合"[常用字段:智能] OR "体外膜式氧合"[常用字段:智能] OR "膜肺氧合"[常用字段:智能] OR "体外膜氧合"[常用字段:智能] OR "体外生命支持"[常用字段:智能] OR "ECLS"[常用字段:智能]) AND( "创伤"[常用字段:智能] OR "坠落"[常用字段:智能] OR "车祸"[常用字段:智能] OR "多发伤"[常用字段:智能] OR "烧伤"[常用字段:智能] OR "钝挫"[常用字段:智能] OR "贯通"[常用字段:智能] OR "震爆"[常用字段:智能] OR "撞击"[常用字段:智能] OR "刺伤"[常用字段:智能] OR "切割"[常用字段:智能] OR "挫伤"[常用字段:智能] OR "烫伤"[常用字段:智能] OR "撕裂伤"[常用字段:智能] OR "挤压"[常用字段:智能] OR "火器伤"[常用字段:智能] OR "电击"[常用字段:智能]) AND( "颅"[常用字段:智能] OR "脑"[常用字段:智能] OR "头"[常用字段:智能] OR "胸"[常用字段:智能] OR "肺"[常用字段:智能] OR "气道"[常用字段:智能] OR "气管"[常用字段:智能] OR "吸入性损伤"[常用字段:智能] OR "低氧血症"[常用字段:智能] OR "ARDS"[常用字段:智能] OR "急性呼吸窘迫综合征"[常用字段:智能] OR "成人型呼吸窘迫综合征"[常用字段:智能] OR "急性呼吸窘迫综合症"[常用字段:智能] OR "急性呼吸窘迫症"[常用字段:智能] OR "急性呼吸窘迫征"[常用字段:智能] OR "成人呼吸窘迫综合征"[常用字段:智能] OR "肺保护通气"[常用字段:智能] OR "保护性肺通气"[常用字段:智能] OR "保护性通气"[常用字段:智能] OR "创伤性湿肺"[常用字段:智能] OR "心"[常用字段:智能] OR "启动"[常用字段:智能] OR "开始"[常用字段:智能] OR "评估"[常用字段:智能] OR "严重程度"[常用字段:智能] OR "器官损害"[常用字段:智能] OR "年龄"[常用字段:智能] OR "合并症"[常用字段:智能] OR "预后"[常用字段:智能] OR "医疗资源"[常用字段:智能] OR "器官捐献"[常用字段:智能] OR "伦理"[常用字段:智能])  The search was conducted from the inception of the library to February 21, 2024, and **646** articles were retrieved. |

| **Search strategy of Question 2: Patient screening** |
| --- |
| **PubMed** |
| ("Injury Severity Score"[MeSH] OR "ISS Score" OR Injury Severity Score*) AND (("Burns"[MeSH] OR "Wounds and Injuries"[MeSH] OR Trauma* OR Injur* OR Wound* OR burn* OR Scald)) AND ("Extracorporeal Membrane Oxygenation"[MeSH] OR (Extracorporeal Membrane Oxygenation*) OR (Membrane Oxygenation, Extracorporeal) OR (Oxygenation, Extracorporeal Membrane) OR ECMO OR ECLS OR (Extracorporeal Life Support*) OR (Life Support, Extracorporeal)) NOT (children OR infant OR child OR pediatric OR neonatal OR baby OR neonat OR newborn OR porcine OR mouse OR mice OR rabbit OR pigs)  The search was conducted from the inception of the library to February 22, 2024, and **177** articles were retrieved. |
| **Embase** |
| ('injury severity score'/exp OR 'injury severity score' OR 'iss score' OR 'injury'/exp OR injury) AND ('severity'/exp OR severity) AND score* AND ('burns'/exp OR 'burns' OR 'wounds and injuries'/exp OR 'wounds and injuries' OR trauma* OR injur* OR wound* OR burn* OR 'scald'/exp OR scald) AND ('extracorporeal membrane oxygenation'/exp OR 'extracorporeal membrane oxygenation' OR (extracorporeal AND ('membrane'/exp OR membrane) AND oxygenation*) OR (('membrane'/exp OR membrane) AND ('oxygenation,'/exp OR oxygenation,) AND extracorporeal) OR (('oxygenation,'/exp OR oxygenation,) AND extracorporeal AND ('membrane'/exp OR membrane)) OR ecmo OR ecls OR (extracorporeal AND ('life'/exp OR life) AND support*) OR (('life'/exp OR life) AND support, AND extracorporeal)) NOT ('children'/exp OR children OR 'infant'/exp OR infant OR 'child'/exp OR child OR 'pediatric'/exp OR pediatric OR neonatal OR 'baby'/exp OR baby OR neonat OR 'newborn'/exp OR newborn OR 'porcine'/exp OR porcine OR 'mouse'/exp OR mouse OR 'mice'/exp OR mice OR 'rabbit'/exp OR rabbit OR 'pigs'/exp OR pigs)  The search was conducted from the inception of the library to February 22, 2024, and **321** articles were retrieved. |
| **The Cochrane Library** |
| ("Injury Severity Score" OR "ISS Score" OR Injury Severity Score*) AND (("Burns" OR "Wounds and Injuries" OR Trauma* OR Injur* OR Wound* OR burn* OR Scald)) AND ("Extracorporeal Membrane Oxygenation" OR (Extracorporeal Membrane Oxygenation*) OR (Membrane Oxygenation, Extracorporeal) OR (Oxygenation, Extracorporeal Membrane) OR ECMO OR ECLS OR (Extracorporeal Life Support*) OR (Life Support, Extracorporeal)) NOT (children OR infant OR child OR pediatric OR neonatal OR baby OR neonat OR newborn OR porcine OR mouse OR mice OR rabbit OR pigs)  The search was conducted from the establishment of the library to February 22, 2024, and **89** articles were retrieved. |
| **China National Knowledge Infrastructure** |
| (TKA%=损伤严重度评分 + 创伤严重度评分 + 损伤严重程度评分 + 创伤严重程度评分 + ISS) AND (TKA%=体外模式氧合 + 体外膜式氧合 + 体外膜肺 + 膜肺氧合 + 体外膜氧合 + 体外生命支持 + ECMO + ECLS)  The search was conducted from the establishment of the library to February 22, 2024, and **82** articles were retrieved. |
| **Wanfang Database** |
| (ecmo or "体外膜肺" or "体外模式氧合" or "体外膜式氧合" or "膜肺氧合" or "体外膜氧合" or "体外生命支持" or ECLS) AND (损伤严重度评分 OR 创伤严重度评分 OR 损伤严重程度评分 OR 创伤严重程度评分 OR ISS)  The search was conducted from the inception of the library to February 22, 2024, and **217** articles were retrieved. |
| **VIP Information (Weipu database in Chinese)** |
| (U=(ecmo OR 体外膜肺 OR 体外模式氧合 OR 体外膜式氧合 OR 膜肺氧合 OR 体外膜氧合 OR 体外生命支持 OR ECLS)) AND (U=(损伤严重度评分 OR 创伤严重度评分 OR 损伤严重程度评分 OR 创伤严重程度评分 OR ISS))  The search was conducted from the establishment of the library to February 22, 2024, and **3** articles were retrieved. |
| **SinoMed** |
| (ecmo OR 体外膜肺 OR 体外模式氧合 OR 体外膜式氧合 OR 膜肺氧合 OR 体外膜氧合 OR 体外生命支持 OR ECLS) AND (损伤严重度评分 OR 创伤严重度评分 OR 损伤严重程度评分 OR 创伤严重程度评分 OR ISS)  The search was conducted from the establishment of the library to February 22, 2024, and **59** articles were retrieved. |

| **Search strategy of question 3: Timing of initiation** |
| --- |
| **PubMed** |
| (prompt OR quick OR speedy OR timely OR earliness OR early) AND (("Burns"[MeSH] OR "Wounds and Injuries"[MeSH] OR Trauma* OR Injur* OR Wound* OR burn* OR Scald)) AND ("Extracorporeal Membrane Oxygenation"[MeSH] OR (Extracorporeal Membrane Oxygenation*) OR (Membrane Oxygenation, Extracorporeal) OR (Oxygenation, Extracorporeal Membrane) OR ECMO OR ECLS OR (Extracorporeal Life Support*) OR (Life Support, Extracorporeal)) NOT (children OR infant OR child OR pediatric OR neonatal OR baby OR neonat OR newborn OR porcine OR mouse OR mice OR rabbit OR pigs)  The search was conducted from the establishment of the library to February 22, 2024, and **538** articles were retrieved. |
| **Embase** |
| (prompt OR quick OR speedy OR timely OR earliness OR early) AND ((Burns OR Wounds and Injuries OR Trauma OR Injur OR Wound OR burn OR Scald)) AND (Extracorporeal Membrane Oxygenation OR (Extracorporeal Membrane Oxygenation) OR (Membrane Oxygenation, Extracorporeal) OR (Oxygenation, Extracorporeal Membrane) OR ECMO OR ECLS OR (Extracorporeal Life Support) OR (Life Support, Extracorporeal)) NOT (children OR infant OR child OR pediatric OR neonatal OR baby OR neonat OR newborn OR porcine OR mouse OR mice OR rabbit OR pigs)  The search was conducted from the inception of the library to February 22, 2024, and **279** articles were retrieved. |
| **The Cochrane Library** |
| #1 children OR infant OR child OR pediatric OR neonatal OR baby OR neonat OR newborn OR porcine OR mouse OR mice OR rabbit OR pigs  #2 prompt OR quick OR speedy OR timely OR earliness OR early  #3 Trauma* OR Injur* OR Wound* OR burn* OR Scald  #4 Extracorporeal Membrane Oxygenation  #5 (Extracorporeal Membrane Oxygenation*) OR (Membrane Oxygenation, Extracorporeal) OR (Oxygenation, Extracorporeal Membrane) OR ECMO OR ECLS OR (Extracorporeal Life Support*) OR (Life Support, Extracorporeal)  #6 MeSH descriptor: [Burns] explode all trees  #7 MeSH descriptor: [Wounds and Injuries] explode all trees  #8 MeSH descriptor: [Extracorporeal Membrane Oxygenation] explode all trees  #9 #2 AND((#6 OR #7 OR #3) AND(#5 OR #8)) NOT #1  The search was conducted from the establishment of the library to February 22, 2024, and **38** articles were retrieved. |
| **China National Knowledge Infrastructure** |
| (TKA%=体外模式氧合 + 体外膜式氧合 + 体外膜肺 + 膜肺氧合 + 体外膜氧合 + 体外生命支持 + ECMO + ECLS) AND (TKA%=创伤 + 坠落 + 车祸 + 多发伤 + 烧伤 + 钝挫 + 贯通 + 震爆 + 撞击 + 刺伤 + 切割 + 挫伤 + 烫伤 + 撕裂伤 + 挤压 + 火器伤 + 电击) AND (TKA%=早 + 难治 + 衰竭 + 时机 + 时间)  The search was conducted from the establishment of the library to February 22, 2024, and **204** articles were retrieved. |
| **VIP Information (Weipu database in Chinese)** |
| (U=(体外模式氧合 + 体外膜式氧合 + 体外膜肺 + 膜肺氧合 + 体外膜氧合 + 体外生命支持 + ECMO + ECLS) AND (创伤 + 坠落 + 车祸 + 多发伤 + 烧伤 + 钝挫 + 贯通 + 震爆 + 撞击 + 刺伤 + 切割 + 挫伤 + 烫伤 + 撕裂伤 + 挤压 + 火器伤 + 电击) AND (早 + 难治 + 衰竭 + 时机 + 时间))  The search was conducted from the establishment of the library to February 22, 2024, and **107** articles were retrieved. |
| **Wanfang Database** |
| 主题:(ecmo or "体外膜肺" or "体外模式氧合" or "体外膜式氧合" or "膜肺氧合" or "体外膜氧合" or "体外生命支持" or ECLS) and 主题:(创伤 or 坠落 or 车祸 or 多发伤 or 烧伤 or 钝挫 or 贯通 or 震爆 or 撞击 or 刺伤 or 切割 or 挫伤 or 烫伤 or 撕裂伤 or 挤压 or 火器伤 or 电击) and 主题:(早 or 难治 or 衰竭 or 时机 or 时间)  The search was conducted from the establishment of the library to February 22, 2024, and **155** articles were retrieved. |
| **SinoMed** |
| 主题:(ecmo or "体外膜肺" or "体外模式氧合" or "体外膜式氧合" or "膜肺氧合" or "体外膜氧合" or "体外生命支持" or ECLS) and 主题:(创伤 or 坠落 or 车祸 or 多发伤 or 烧伤 or 钝挫 or 贯通 or 震爆 or 撞击 or 刺伤 or 切割 or 挫伤 or 烫伤 or 撕裂伤 or 挤压 or 火器伤 or 电击) and 主题:(早 or 难治 or 衰竭 or 时机 or 时间)  The search was conducted from the establishment of the library to February 22, 2024, and **484** articles were retrieved. |

| **Search strategy of question 4: Multidisciplinary** |
| --- |
| **PubMed** |
| (("trauma*"[Title/Abstract] OR "injur*"[Title/Abstract] OR "wound*"[Title/Abstract] OR "burn*"[Title/Abstract] OR "Scald"[Title/Abstract]) AND ("extracorporeal membrane oxygenation*"[Title/Abstract] OR "membrane oxygenation extracorporeal"[Title/Abstract] OR "oxygenation extracorporeal membrane"[Title/Abstract] OR "ECMO"[Title/Abstract] OR "ECLS"[Title/Abstract] OR "extracorporeal life support*"[Title/Abstract] OR "life support extracorporeal"[Title/Abstract]) AND ((("multidisciplinar*"[Title/Abstract] OR "Interdisciplinary" [Title/Abstract] OR "Cross-disciplinary"[Title/Abstract] OR "Multi-field"[Title/Abstract] OR "Pluralistic" [Title/Abstract] OR "Polydisciplinary"[Title/Abstract] OR "multi department*"[Title/Abstract] OR "inter department*"[Title/Abstract] OR "Cross-departmental"[Title/Abstract] OR "Multi-sectional"[Title/Abstract]) AND ("team*"[Title/Abstract] OR "Group"[Title/Abstract] OR "Squad"[Title/Abstract] OR "Crew"[Title/Abstract] OR "Unit"[Title/Abstract] OR "Collective"[Title/Abstract] OR "Ensemble"[Title/Abstract])) OR ("Procedure" [Title/Abstract] OR "Workflow"[Title/Abstract] OR "Routine"[Title/Abstract] OR "Method"[Title/Abstract] OR "Course"[Title/Abstract] OR "process*"[Title/Abstract] OR "warn*"[Title/Abstract] OR "Alert"[Title/Abstract] OR "Forewarning"[Title/Abstract] OR "Precautionary"[Title/Abstract] OR "Advisory"[Title/Abstract] OR "institution*"[Title/Abstract] OR "Regime"[Title/Abstract] OR "Framework"[Title/Abstract] OR "Structure" [Title/Abstract] OR "Establishment"[Title/Abstract] OR "Organization"[Title/Abstract] OR "Policy" [Title/Abstract] OR "qualit*"[Title/Abstract] OR "standard*"[Title/Abstract] OR "Criterion"[Title/Abstract] OR "Measure"[Title/Abstract] OR "Benchmark"[Title/Abstract] OR "Norm"[Title/Abstract] OR "Gauge" [Title/Abstract] OR "Yardstick"[Title/Abstract] OR "transport*"[Title/Abstract] OR "Transfer"[Title/Abstract] OR "Conveyance"[Title/Abstract] OR "Shipment"[Title/Abstract] OR "Relocation"[Title/Abstract] OR "Move" [Title/Abstract] OR "Convey"[Title/Abstract] OR "Shift"[Title/Abstract]))) NOT ("children"[Title/Abstract] OR "infant"[Title/Abstract] OR "child"[Title/Abstract] OR "pediatric"[Title/Abstract] OR "neonatal"[Title/Abstract] OR "baby"[Title/Abstract] OR "neonat"[Title/Abstract] OR "newborn"[Title/Abstract] OR "porcine" [Title/Abstract] OR "mouse"[Title/Abstract] OR "mice"[Title/Abstract] OR "rabbit"[Title/Abstract] OR "pigs"[Title/Abstract])  The search was conducted from the inception of the library to February 22, 2024, and **878** articles were retrieved. |
| **Embase** |
| #1. 'extracorporeal membrane oxygenation*':ab,ti OR 'membrane oxygenation extracorporeal':ab,ti OR 'oxygenation extracorporeal membrane':ab,ti OR 'ecmo':ab,ti OR 'ecls':ab,ti OR 'extracorporeal life support*':ab,ti OR 'life support extracorporeal':ab,ti  #2. 'trauma*':ab,ti OR 'injur*':ab,ti OR 'wound*':ab,ti OR 'burn*':ab,ti OR 'scald':ab,ti  #3. 'multidisciplinar*':ab,ti OR 'interdisciplinary':ab,ti OR 'cross-disciplinary':ab,ti OR 'multi-field':ab,ti OR 'pluralistic':ab,ti OR 'polydisciplinary':ab,ti OR 'multi department*':ab,ti OR 'inter department*':ab,ti OR 'cross-departmental':ab,ti OR 'multi-sectional':ab,ti  #4. 'team*':ab,ti OR 'group':ab,ti OR 'squad':ab,ti OR 'crew':ab,ti OR 'unit':ab,ti OR 'collective':ab,ti OR 'ensemble':ab,ti  #5. 'procedure':ab,ti OR 'workflow':ab,ti OR 'routine':ab,ti OR 'method':ab,ti OR 'course':ab,ti OR 'process':ab,ti OR 'warn*':ab,ti OR 'alert':ab,ti OR 'forewarning':ab,ti OR 'precautionary':ab,ti OR 'advisory':ab,ti OR 'institution*':ab,ti OR 'regime':ab,ti OR 'framework':ab,ti OR 'structure':ab,ti OR 'establishment':ab,ti OR 'organization':ab,ti OR 'policy':ab,ti OR 'qualit*':ab,ti OR 'standard*':ab,ti OR 'criterion':ab,ti OR 'measure':ab,ti OR 'benchmark':ab,ti OR 'norm':ab,ti OR 'gauge':ab,ti OR 'yardstick':ab,ti OR 'transport':ab,ti OR 'transfer':ab,ti OR 'conveyance':ab,ti OR 'shipment':ab,ti OR 'relocation':ab,ti OR 'move':ab,ti OR 'convey':ab,ti OR 'shift':ab,ti  #6. 'children':ab,ti OR 'infant':ab,ti OR 'child':ab,ti OR 'pediatric':ab,ti OR 'neonatal':ab,ti OR 'baby':ab,ti OR 'neonat':ab,ti OR 'newborn':ab,ti OR 'porcine':ab,ti OR 'mouse':ab,ti OR 'mice':ab,ti OR 'rabbit':ab,ti OR 'pigs':ab,ti  #7. #3 AND #4  #8. #5 OR #7  #9. #1 AND #2 AND #8  #10. #9 NOT #6  The search was conducted from the inception of the library to February 22, 2024, and **1859** articles were retrieved. |
| **The Cochrane Library** |
| #1 trauma* OR injur* OR wound* OR burn* OR Scald  #2 extracorporeal membrane oxygenation* OR membrane oxygenation extracorporeal OR oxygenation extracorporeal membrane OR ECMO OR ECLS OR extracorporeal life support* OR life support extracorporeal  #3 multidisciplinar* OR Interdisciplinary OR Cross-disciplinary OR Multi-field OR Pluralistic OR Polydisciplinary OR multi department* OR inter department* OR Cross-departmental OR Multi-sectional  #4 team* OR Group OR Squad OR Crew OR Unit OR Collective OR Ensemble  #5 Procedure OR Workflow OR Routine OR Method OR Course OR process* OR warn* OR Alert OR Forewarning OR Precautionary OR Advisory OR institution* OR Regime OR Framework OR Structure OR Establishment OR Organization OR Policy OR qualit* OR standard* OR Criterion OR Measure OR Benchmark OR Norm OR Gauge OR Yardstick OR transport* OR Transfer OR Conveyance OR Shipment OR Relocation OR Move OR Convey OR Shift  #6 children OR infant OR child OR pediatric OR neonatal OR baby OR neonat OR newborn OR porcine OR mouse OR mice OR rabbit OR pigs  #7 MeSH descriptor: [Wounds and Injuries] explode all trees  #8 MeSH descriptor: [Extracorporeal Membrane Oxygenation] explode all trees  #9 MeSH descriptor: [Interdisciplinary Research] explode all trees  #10 MeSH descriptor: [Methods] explode all trees  #11 MeSH descriptor: [Child] explode all trees  #12 #1 OR #7  #13 #2 OR #8  #14 #3 OR #9  #15 #5 OR #10  #16 #6 OR #11  #17 #14 AND #4  #18 #17 OR #15  #19 #18 AND #12 AND #13  #20 #19 NOT #16  The search was conducted from the inception of the library to February 22, 2024, and **187** articles were retrieved. |
| **China National Knowledge Infrastructure** |
| (TKA%=体外模式氧合 + 体外膜式氧合 + 体外膜肺 + 膜肺氧合 + 体外膜氧合 + 体外生命支持 + ECMO + ECLS) AND (TKA%=创伤 + 坠落 + 车祸 + 多发伤 + 烧伤 + 钝挫 + 贯通 + 震爆 + 撞击 + 刺伤 + 切割 + 挫伤 + 烫伤 + 撕裂伤 + 挤压 + 火器伤 + 电击) AND (TKA%=多学科 + 跨学科 + 团队 + 流程 + 程序 + 步骤 + 预警 + 制度 + 质控 + 质量控制 + 标准 + 规范 + 准则 + 转运)  The search was conducted from the inception of the library to February 22, 2024, and **133** articles were retrieved. |
| **VIP Information (Weipu database in Chinese)** |
| 题名或关键词=ecmo or 体外膜肺 or 体外模式氧合 or 体外膜式氧合 or 膜肺氧合 or 体外膜氧合 or 体外生命支持 or ECLS AND 题名或关键词=创伤 or 坠落 or 车祸 or 多发伤 or 烧伤 or 钝挫 or 贯通 or 震爆 or 撞击 or 刺伤 or 切割 or 挫伤 or 烫伤 or 撕裂伤 or 挤压 or 火器伤 or 电击 AND 题名或关键词=多学科 + 跨学科 + 团队 + 流程 + 程序 + 步骤 + 预警 + 制度 + 质控 + 质量控制 + 标准 + 规范 + 准则 + 转运  The search was conducted from the inception of the library to February 22, 2024, and **5** articles were retrieved. |
| **Wanfang Database** |
| 主题:(ecmo or "体外膜肺" or "体外模式氧合" or "体外膜式氧合" or "膜肺氧合" or "体外膜氧合" or "体外生命支持" or ECLS) and 主题:(创伤 or 坠落 or 车祸 or 多发伤 or 烧伤 or 钝挫 or 贯通 or 震爆 or 撞击 or 刺伤 or 切割 or 挫伤 or 烫伤 or 撕裂伤 or 挤压 or 火器伤 or 电击) and 主题:(多学科 + 跨学科 + 团队 + 流程 + 程序 + 步骤 + 预警 + 制度 + 质控 + 质量控制 + 标准 + 规范 + 准则 + 转运)  The search was conducted from the inception of the library to February 22, 2024, and **269** articles were retrieved. |
| **SinoMed** |
| ( "ecmo"[全部字段:智能] OR "体外膜肺"[全部字段:智能] OR "体外模式氧合"[全部字段:智能] OR "体外膜式氧合"[全部字段:智能] OR "膜肺氧合"[全部字段:智能] OR "体外膜氧合"[全部字段:智能] OR "体外生命支持"[全部字段:智能] OR "ECLS"[全部字段:智能]) AND ( "创伤"[全部字段:智能] OR "坠落"[全部字段:智能] OR "车祸"[全部字段:智能] OR "多发伤"[全部字段:智能] OR "烧伤"[全部字段:智能] OR "钝挫"[全部字段:智能] OR "贯通"[全部字段:智能] OR "震爆"[全部字段:智能] OR "撞击"[全部字段:智能] OR "刺伤"[全部字段:智能] OR "切割"[全部字段:智能] OR "挫伤"[全部字段:智能] OR "烫伤"[全部字段:智能] OR "撕裂伤"[全部字段:智能] OR "挤压"[全部字段:智能] OR "火器伤"[全部字段:智能] OR "电击"[全部字段:智能]) AND ( "多学科"[全部字段:智能] OR "跨学科"[全部字段:智能] OR "团队"[全部字段:智能] OR "流程"[全部字段:智能] OR "程序"[全部字段:智能] OR "步骤"[全部字段:智能] OR "预警"[全部字段:智能] OR "制度"[全部字段:智能] OR "质控"[全部字段:智能] OR "质量控制"[全部字段:智能] OR "标准"[全部字段:智能] OR "规范"[全部字段:智能] OR "准则"[全部字段:智能] OR "转运"[全部字段:智能])  The search was conducted from the inception of the library to February 22, 2024, and **144** articles were retrieved. |

| **Search strategy of question 5: Anticoagulation** |
| --- |
| **PubMed** |
| ("Blood Coagulation"[MeSH] OR "Anticoagulants"[MeSH] OR "Thrombelastography"[MeSH] OR "Partial Thromboplastin Time"[MeSH] OR Coagulation OR Anticoagulation OR Thromboelastography OR (Activated Partial Thromboplastin Time) OR APTT OR (Activated Clotting Time) OR Clotting OR Congealing OR Solidification OR Thickening OR Anti-clotting OR (Blood thinning) OR Antithrombotic OR (Anticoagulant therapy) OR Anticoagulant OR Anticoagulate OR Thromboelastometry OR (Hemorheological analysis) OR Thromboelastic OR Thromboelasticity OR (Partial thromboplastin time) OR (Clotting time assay) OR Thromboplastin OR Thromboplastic OR (Whole blood clotting time) OR Clot OR "TEG" OR "ACT") AND (("Burns"[MeSH] OR "Wounds and Injuries"[MeSH] OR Trauma* OR Injur* OR Wound* OR burn* OR Scald)) AND ("Extracorporeal Membrane Oxygenation"[MeSH] OR (Extracorporeal Membrane Oxygenation*) OR (Membrane Oxygenation, Extracorporeal) OR (Oxygenation, Extracorporeal Membrane) OR ECMO OR ECLS OR (Extracorporeal Life Support*) OR (Life Support, Extracorporeal)) NOT (children OR infant OR child OR pediatric OR neonatal OR baby OR neonat OR newborn OR porcine OR mouse OR mice OR rabbit OR pigs)  The search was conducted from the establishment of the library to February 21, 2024, and **304** articles were retrieved. |
| **Embase** |
| ("Blood Coagulation" OR "Anticoagulants" OR "Thrombelastography" OR "Partial Thromboplastin Time" OR "Coagulation" OR "Anticoagulation" OR "Activated Partial Thromboplastin Time" OR "APTT" OR "Activated Clotting Time" OR "Clotting" OR "Congealing" OR "Solidification" OR "Thickening" OR "Anti-clotting" OR "Blood thinning" OR "Antithrombotic" OR "Anticoagulant therapy" OR "Anticoagulant" OR "Anticoagulate" OR "Thromboelastometry" OR "Hemorheological analysis" OR "Thromboelastic" OR "Thromboelasticity" OR "Partial thromboplastin time" OR "Clotting time assay" OR "Thromboplastin" OR "Thromboplastic" OR "Whole blood clotting time" OR "Clot" OR "TEG" OR "ACT") AND ("Burns" OR "Wounds" and "Injuries" OR "Wounds" OR "burn" OR "Scald" OR "Injuries") AND ("Extracorporeal Membrane Oxygenation" OR "Extracorporeal Membrane Oxygenation*" OR "Membrane Oxygenation, Extracorporeal" OR "Oxygenation, Extracorporeal Membrane" OR "ECMO" OR "ECLS" OR "Extracorporeal Life Support*" OR "Life Support, Extracorporeal")  The search was conducted from the establishment of the library to February 22, 2024, and **144** articles were retrieved. |
| **The Cochrane Library** |
| (Blood Coagulation OR Anticoagulants OR Thrombelastography OR Partial Thromboplastin Time OR Coagulation OR Anticoagulation OR (Activated Partial Thromboplastin Time) OR APTT OR (Activated Clotting Time) OR Clotting OR Congealing OR Solidification OR Thickening OR Anti-clotting OR (Blood thinning) OR Antithrombotic OR (Anticoagulant therapy) OR Anticoagulant OR Anticoagulate OR Thromboelastometry OR (Hemorheological analysis) OR Thromboelastic OR Thromboelasticity OR Partial thromboplastin time OR Clotting time assay OR Thromboplastin OR Thromboplastic OR Whole blood clotting time OR Clot OR TEG OR ACT) in Title Abstract Keyword AND (Burns OR Wounds and Injuries OR Trauma OR Injur OR Wound OR burn OR Scald) in Title Abstract Keyword AND (Extracorporeal Membrane Oxygenation OR Extracorporeal Membrane Oxygenation OR Membrane Oxygenation, Extracorporeal OR Oxygenation, Extracorporeal Membrane OR ECMO OR ECLS OR Extracorporeal Life Support OR Life Support, Extracorporeal) in Title Abstract Keyword NOT (children OR infant OR child OR pediatric OR neonatal OR baby OR neonat OR newborn OR porcine OR mouse OR mice OR rabbit OR pigs) in Title Abstract Keyword - (Word variations have been searched)  The search was conducted from the establishment of the library to February 21, 2024, and **7** articles were retrieved. |
| **China National Knowledge Infrastructure** |
| (TKA%=体外模式氧合 + 体外膜式氧合 + 体外膜肺 + 膜肺氧合 + 体外膜氧合 + 体外生命支持 + ECMO + ECLS) AND (TKA%=创伤 + 坠落 + 车祸 + 多发伤 + 烧伤 + 钝挫 + 贯通 + 震爆 + 撞击 + 刺伤 + 切割 + 挫伤 + 烫伤 + 撕裂伤 + 挤压 + 火器伤 + 电击) AND (TKA%=凝血 + 抗凝 + 血栓弹力图 + APTT + ACT + 血液凝固 + 血细胞凝集 + 血凝 + 活化部分凝血活酶时间 + 活化部分凝血酶时间 + 活化部分凝血酶原时间 + 活化凝血时间 + 激活全血凝固时间)  The search was conducted from the establishment of the library to February 21, 2024, and **67** articles were retrieved. |
| **Wanfang Database** |
| 主题:(ecmo or "体外膜肺" or "体外模式氧合" or "体外膜式氧合" or "膜肺氧合" or "体外膜氧合" or "体外生命支持" or ECLS) and 主题:(创伤 or 坠落 or 车祸 or 多发伤 or 烧伤 or 钝挫 or 贯通 or 震爆 or 撞击 or 刺伤 or 切割 or 挫伤 or 烫伤 or 撕裂伤 or 挤压 or 火器伤 or 电击) and 主题:(凝血 or 抗凝 or 血栓弹力图 or APTT or ACT or 血液凝固 or 凝血or血细胞凝集or血凝or活化部分凝血活酶时间or活化部分凝血酶时间or活化部分凝血酶原时间or活化凝血时间or激活全血凝固时间)  The search was conducted from the establishment of the library to February 21, 2024, and **36** articles were retrieved. |
| **VIP Information (Weipu database in Chinese)** |
| (U=体外模式氧合 + 体外膜式氧合 + 体外膜肺 + 膜肺氧合 + 体外膜氧合 + 体外生命支持 + ECMO + ECLS) AND (U=创伤 + 坠落 + 车祸 + 多发伤 + 烧伤 + 钝挫 + 贯通 + 震爆 + 撞击 + 刺伤 + 切割 + 挫伤 + 烫伤 + 撕裂伤 + 挤压 + 火器伤 + 电击) AND (U=凝血 + 抗凝 + 血栓弹力图 + APTT + ACT + 血液凝固 + 血细胞凝集 + 血凝 + 活化部分凝血活酶时间 + 活化部分凝血酶时间 + 活化部分凝血酶原时间 + 活化凝血时间 + 激活全血凝固时间)  The search was conducted from the establishment of the library to February 21, 2024, and **30** articles were retrieved. |
| **SinoMed** |
| ("ECMO"[常用字段:智能] OR "ECLS"[常用字段:智能] OR "体外膜肺"[常用字段:智能] OR "体外模式氧合"[常用字段:智能] OR "体外膜式氧合"[常用字段:智能] OR "膜肺氧合"[常用字段:智能] OR "体外膜氧合"[常用字段:智能] OR "体外生命支持"[常用字段:智能]) AND ("创伤"[常用字段:智能] OR "坠落"[常用字段:智能] OR "车祸"[常用字段:智能] OR "多发伤"[常用字段:智能] OR "烧伤"[常用字段:智能] OR "钝挫"[常用字段:智能] OR "贯通"[常用字段:智能] OR "震爆"[常用字段:智能] OR "撞击"[常用字段:智能] OR "刺伤"[常用字段:智能] OR "切割"[常用字段:智能] OR "挫伤"[常用字段:智能] OR "烫伤"[常用字段:智能] OR "撕裂伤"[常用字段:智能] OR "挤压"[常用字段:智能] OR "火器伤"[常用字段:智能] OR "电击"[常用字段:智能]) AND ("凝血"[常用字段:智能] OR "抗凝"[常用字段:智能] OR "血栓弹力图"[常用字段:智能] OR "APTT"[常用字段:智能] OR "ACT"[常用字段:智能] OR "血液凝固"[常用字段:智能] OR "凝血"[常用字段:智能] OR "血细胞凝集"[常用字段:智能] OR "血凝"[常用字段:智能] OR "活化部分凝血活酶时间"[常用字段:智能] OR "活化部分凝血酶时间"[常用字段:智能] OR "活化部分凝血酶原时间"[常用字段:智能] OR "活化凝血时间"[常用字段:智能] OR "激活全血凝固时间"[常用字段:智能])  The search was conducted from the establishment of the library to February 21, 2024, and **70** articles were retrieved. |

| **Search strategy of question 6: Traumatic lethal triad** |
| --- |
| **PubMed** |
| ("Blood Coagulation Disorders"[MeSH] OR "Hypothermia"[MeSH] OR "Acidosis"[MeSH] OR Acidos* OR Bleeding disorder OR Blood clotting disorder OR Coagulation disorder OR Hemorrhagic condition OR Low body temperature OR Subnormal temperature OR Chilliness OR Cold exposure OR Freezing OR Undercooling OR Acidic condition OR Acid-base imbalance) AND (("Burns"[MeSH] OR "Wounds and Injuries"[MeSH] OR Trauma* OR Injur* OR Wound* OR burn* OR Scald)) AND ("Extracorporeal Membrane Oxygenation"[MeSH] OR (Extracorporeal Membrane Oxygenation*) OR (Membrane Oxygenation, Extracorporeal) OR (Oxygenation, Extracorporeal Membrane) OR ECMO OR ECLS OR (Extracorporeal Life Support*) OR (Life Support, Extracorporeal)) NOT (children OR infant OR child OR pediatric OR neonatal OR baby OR neonat OR newborn OR porcine OR mouse OR mice OR rabbit OR pigs)  The search was conducted from the establishment of the library to February 21, 2024, and **302** articles were retrieved. |
| **Embase** |
| ('acidos*':ab,ti OR 'bleeding disorder*':ab,ti OR 'blood clotting disorder*':ab,ti OR 'coagulation disorder*':ab,ti OR 'hemorrhagic condition*':ab,ti OR 'low body temperature':ab,ti OR 'subnormal temperature':ab,ti OR 'chilliness':ab,ti OR 'cold exposure':ab,ti OR 'freezing':ab,ti OR 'undercooling':ab,ti OR 'acidic condition*':ab,ti OR 'acid-base imbalance*':ab,ti) AND (trauma*:ab,ti OR injur*:ab,ti OR wound*:ab,ti OR burn*:ab,ti OR scald*:ab,ti) AND ('extracorporeal membrane oxygenation*':ab,ti OR ecmo:ab,ti OR ecls:ab,ti OR 'extracorporeal life support*':ab,ti) NOT (children:ab,ti OR infant*:ab,ti OR pediatric:ab,ti OR neonatal:ab,ti OR baby:ab,ti OR neonat*:ab,ti OR newborn:ab,ti OR porcine:ab,ti OR mouse:ab,ti OR mice:ab,ti OR rabbit:ab,ti OR pigs:ab,ti)  The search was conducted from the establishment of the library to February 25, 2024, and **177** articles were retrieved. |
| **The Cochrane Library** |
| (Acidos* OR Bleeding disorder OR Blood clotting disorder OR Coagulation disorder OR Hemorrhagic condition OR Low body temperature OR Subnormal temperature OR Chilliness OR Cold exposure OR Freezing OR Undercooling OR Acidic condition OR Acid-base imbalance) AND ((Trauma* OR Injur* OR Wound* OR burn* OR Scald)) AND ((Extracorporeal Membrane Oxygenation*) OR (Membrane Oxygenation, Extracorporeal) OR (Oxygenation, Extracorporeal Membrane) OR ECMO OR ECLS OR (Extracorporeal Life Support*) OR (Life Support, Extracorporeal)) NOT (children OR infant OR child OR pediatric OR neonatal OR baby OR neonat OR newborn OR porcine OR mouse OR mice OR rabbit OR pigs)  The search was conducted from the establishment of the library to February 25, 2024, and **18** articles were retrieved. |
| **China National Knowledge Infrastructure** |
| (TKA%=体外模式氧合 + 体外膜式氧合 + 体外膜肺 + 膜肺氧合 + 体外膜氧合 + 体外生命支持 + ECMO + ECLS) AND (TKA%=创伤 + 坠落 + 车祸 + 多发伤 + 烧伤 + 钝挫 + 贯通 + 震爆 + 撞击 + 刺伤 + 切割 + 挫伤 + 烫伤 + 撕裂伤 + 挤压 + 火器伤 + 电击) AND (TKA%=凝血 + 体温 + 温度 + 酸中毒)  The search was conducted from the establishment of the library to February 25, 2024, and **65** articles were retrieved. |
| **Wanfang Database** |
| 主题:(ecmo or "体外膜肺" or "体外模式氧合" or "体外膜式氧合" or "膜肺氧合" or "体外膜氧合" or "体外生命支持" or ECLS) and 主题:(创伤 or 坠落 or 车祸 or 多发伤 or 烧伤 or 钝挫 or 贯通 or 震爆 or 撞击 or 刺伤 or 切割 or 挫伤 or 烫伤 or 撕裂伤 or 挤压 or 火器伤 or 电击) and 主题:(凝血 or 体温 or 温度 or 酸中毒)  The search was conducted from the establishment of the library to February 25, 2024, and **30** articles were retrieved. |
| **VIP Information (Weipu database in Chinese)** |
| (((((((((任意字段=体外模式氧合 OR 任意字段=体外膜式氧合) OR 任意字段=体外膜肺) OR 任意字段=膜肺氧合) OR 任意字段=体外膜氧合) OR 任意字段=体外生命支持) OR 任意字段=ECMO) OR 任意字段=ECLS) AND ((((((((((((((((任意字段=创伤 OR 任意字段=坠落) OR 任意字段=车祸) OR 任意字段=多发伤) OR 任意字段=烧伤) OR 任意字段=钝挫) OR 任意字段=贯通) OR 任意字段=震爆) OR 任意字段=撞击) OR 任意字段=刺伤) OR 任意字段=切割) OR 任意字段=挫伤) OR 任意字段=烫伤) OR 任意字段=撕裂伤) OR 任意字段=挤压) OR 任意字段=火器伤) OR 任意字段=电击)) AND (((任意字段=凝血 OR 任意字段=体温) OR 任意字段=温度) OR 任意字段=酸中毒))  The search was conducted from the inception of the library to February 26, 2024, and **31** articles were retrieved. |
| **SinoMed** |
| ( "ecmo"[全部字段:智能] OR "体外膜肺"[全部字段:智能] OR "体外模式氧合"[全部字段:智能] OR "体外膜式氧合"[全部字段:智能] OR "膜肺氧合"[全部字段:智能] OR "体外膜氧合"[全部字段:智能] OR "体外生命支持"[全部字段:智能] OR "ECLS"[全部字段:智能]) AND( "创伤"[全部字段:智能] OR "坠落"[全部字段:智能] OR "车祸"[全部字段:智能] OR "多发伤"[全部字段:智能] OR "烧伤"[全部字段:智能] OR "钝挫"[全部字段:智能] OR "贯通"[全部字段:智能] OR "震爆"[全部字段:智能] OR "撞击"[全部字段:智能] OR "刺伤"[全部字段:智能] OR "切割"[全部字段:智能] OR "挫伤"[全部字段:智能] OR "烫伤"[全部字段:智能] OR "撕裂伤"[全部字段:智能] OR "挤压"[全部字段:智能] OR "火器伤"[全部字段:智能] OR "电击"[全部字段:智能]) AND( "凝血"[全部字段:智能] OR "体温"[全部字段:智能] OR "温度"[全部字段:智能] OR "酸中毒"[全部字段:智能])  The search was conducted from the establishment of the library to February 26, 2024, and **81** articles were retrieved. |

| **Search strategy of question 7: Bedside ultrasound** |
| --- |
| **PubMed** |
| (Ultrasound OR Ultrason* OR "Volume Administration" OR "Capacity Administration" OR "Quantity Management" OR "Volume Control" OR "Volume Regulation" OR "Volume Handling" OR "Volume Oversight" OR "Volume Stewardship" OR "Volume Governance" OR "Fluid Replacement Management" OR "Fluid Resuscitation Management" OR "Rehydration Management" OR "Fluid Replenishment Management" OR "Replacement Fluid Management") AND (("Burns"[MeSH] OR "Wounds and Injuries"[MeSH] OR Trauma* OR Injur* OR Wound* OR burn* OR Scald)) AND ("Extracorporeal Membrane Oxygenation"[MeSH] OR (Extracorporeal Membrane Oxygenation*) OR (Membrane Oxygenation, Extracorporeal) OR (Oxygenation, Extracorporeal Membrane) OR ECMO OR ECLS OR (Extracorporeal Life Support*) OR (Life Support, Extracorporeal)) NOT (children OR infant OR child OR pediatric OR neonatal OR baby OR neonat OR newborn OR porcine OR mouse OR mice OR rabbit OR pigs)  The search was conducted from the establishment of the library to February 21, 2024, and **295** articles were retrieved. |
| **Embase** |
| ('ultrasound'/exp OR ultrasound OR ultrason* OR 'volume administration' OR 'capacity administration' OR 'quantity management' OR 'volume control' OR 'volume regulation' OR 'volume handling' OR 'volume oversight' OR 'volume stewardship' OR 'volume governance' OR 'fluid replacement management' OR 'fluid resuscitation management' OR 'rehydration management' OR 'fluid replenishment management' OR 'replacement fluid management') AND (((((((((((((('burns'/exp OR burns OR 'wounds'/exp OR wounds) AND ('injuries'/exp OR injuries) OR 'wounds'/exp OR wounds OR physical) AND ('trauma'/exp OR trauma) OR physical) AND traumas OR 'trauma,'/exp OR trauma,) AND physical OR traumas OR 'injury'/exp OR injury OR 'injuries'/exp OR injuries OR 'injuries,'/exp OR injuries,) AND ('wounds'/exp OR wounds) OR 'wounds'/exp OR wounds) AND ('injury'/exp OR injury) OR 'injury'/exp OR injury) AND ('wounds'/exp OR wounds) OR 'injuries'/exp OR injuries) AND ('wounds'/exp OR wounds) OR wounds,) AND ('injury'/exp OR injury) OR 'research related') AND ('injury'/exp OR injury) OR 'injury,'/exp OR injury,) AND 'research related' OR 'research'/exp OR research) AND related AND ('injuries'/exp OR injuries) OR 'research related') AND ('injuries'/exp OR injuries) OR trauma* OR injur* OR wound* OR burn* OR 'scald'/exp OR scald) AND (((('extracorporeal membrane oxygenation'/exp OR 'extracorporeal membrane oxygenation' OR 'membrane'/exp OR membrane) AND ('oxygenators'/exp OR oxygenators) OR 'membrane'/exp OR membrane) AND ('oxygenator'/exp OR oxygenator) OR 'oxygenator,'/exp OR oxygenator,) AND ('membrane'/exp OR membrane) OR (extracorporeal AND ('membrane'/exp OR membrane) AND oxygenation*) OR (('membrane'/exp OR membrane) AND ('oxygenation,'/exp OR oxygenation,) AND extracorporeal) OR (('oxygenation,'/exp OR oxygenation,) AND extracorporeal AND ('membrane'/exp OR membrane)) OR ecmo OR ecls OR (extracorporeal AND ('life'/exp OR life) AND support*) OR (('life'/exp OR life) AND support, AND extracorporeal)) NOT ('children'/exp OR children OR 'infant'/exp OR infant OR 'child'/exp OR child OR 'pediatric'/exp OR pediatric OR neonatal OR 'baby'/exp OR baby OR neonat OR 'newborn'/exp OR newborn OR 'porcine'/exp OR porcine OR 'mouse'/exp OR mouse OR 'mice'/exp OR mice OR 'rabbit'/exp OR rabbit OR 'pigs'/exp OR pigs)  The search was conducted from the establishment of the library to February 22, 2024, and **209** articles were retrieved. |
| **The Cochrane Library** |
| (Ultrasound OR Ultrason* OR "Volume Administration" OR "Capacity Administration" OR "Quantity Management" OR "Volume Control" OR "Volume Regulation" OR "Volume Handling" OR "Volume Oversight" OR "Volume Stewardship" OR "Volume Governance" OR "Fluid Replacement Management" OR "Fluid Resuscitation Management" OR "Rehydration Management" OR "Fluid Replenishment Management" OR "Replacement Fluid Management") AND (Burns OR Wounds and Injuries OR Wounds OR Physical Trauma OR Physical Traumas OR Trauma, Physical OR Traumas OR Injury OR Injuries OR Injuries, Wounds OR Wounds and Injury OR Injury and Wounds OR Injuries and Wounds OR Wounds, Injury OR Research-Related Injury OR Injury, Research-Related OR Research Related Injuries OR Research-Related Injuries OR Trauma* OR Injur* OR Wound* OR burn* OR Scald) AND ("Extracorporeal Membrane Oxygenation" OR Membrane Oxygenators OR Membrane Oxygenator OR Oxygenator, Membrane OR (Extracorporeal Membrane Oxygenation*) OR (Membrane Oxygenation, Extracorporeal) OR (Oxygenation, Extracorporeal Membrane) OR ECMO OR ECLS OR (Extracorporeal Life Support*) OR (Life Support, Extracorporeal)) NOT (children OR infant OR child OR pediatric OR neonatal OR baby OR neonat OR newborn OR porcine OR mouse OR mice OR rabbit OR pigs)  The search was conducted from the establishment of the library to February 22, 2024, and **83** articles were retrieved. |
| **China National Knowledge Infrastructure** |
| (TKA%=体外模式氧合 + 体外膜式氧合 + 体外膜肺 + 膜肺氧合 + 体外膜氧合 + 体外生命支持 + ECMO + ECLS) AND (TKA%=创伤 + 坠落 + 车祸 + 多发伤 + 烧伤 + 钝挫 + 贯通 + 震爆 + 撞击 + 刺伤 + 切割 + 挫伤 + 烫伤 + 撕裂伤 + 挤压 + 火器伤 + 电击) AND (TKA%=超声 + 容量管理 + 容量控制 + 容量调节 + 液体置换 + 液体复苏 + 补液)  The search was conducted from the inception of the library to February 26, 2024, and **38** articles were retrieved. |
| **Wanfang Database** |
| 主题:(ecmo OR "体外膜肺" OR "体外模式氧合" OR "体外膜式氧合" OR "膜肺氧合" OR "体外膜氧合" OR "体外生命支持" OR ECLS) AND 主题:(创伤 OR 坠落 OR 车祸 OR 多发伤 OR 烧伤 OR 钝挫 OR 贯通 OR 震爆 OR 撞击 OR 刺伤 OR 切割 OR 挫伤 OR 烫伤 OR 撕裂伤 OR 挤压 OR 火器伤 OR 电击) AND 主题:(超声 OR 容量管理 OR 容量控制 OR 容量调节 OR 液体置换 OR 液体复苏 OR 补液)  The search was conducted from the inception of the library to February 26 2024, and **23** articles were retrieved. |
| **VIP Information (Weipu database in Chinese)** |
| (U=ecmo OR "体外膜肺" OR "体外模式氧合" OR "体外膜式氧合" OR "膜肺氧合" OR "体外膜氧合" OR "体外生命支持" OR ECLS) AND (U=创伤 OR 坠落 OR 车祸 OR 多发伤 OR 烧伤 OR 钝挫 OR 贯通 OR 震爆 OR 撞击 OR 刺伤 OR 切割 OR 挫伤 OR 烫伤 OR 撕裂伤 OR 挤压 OR 火器伤 OR 电击) AND (U=超声 OR 容量管理 OR 容量控制 OR 容量调节 OR 液体置换 OR 液体复苏 OR 补液)  The search was conducted from the establishment of the library to February 22, 2024, and **18** articles were retrieved. |
| **SinoMed** |
| ( "ecmo"[全部字段:智能] OR ""体外膜肺""[全部字段:智能] OR ""体外模式氧合""[全部字段:智能] OR ""体外膜式氧合""[全部字段:智能] OR ""膜肺氧合""[全部字段:智能] OR ""体外膜氧合""[全部字段:智能] OR ""体外生命支持""[全部字段:智能] OR "ECLS"[全部字段:智能]) AND( "创伤"[全部字段:智能] OR "坠落"[全部字段:智能] OR "车祸"[全部字段:智能] OR "多发伤"[全部字段:智能] OR "烧伤"[全部字段:智能] OR "钝挫"[全部字段:智能] OR "贯通"[全部字段:智能] OR "震爆"[全部字段:智能] OR "撞击"[全部字段:智能] OR "刺伤"[全部字段:智能] OR "切割"[全部字段:智能] OR "挫伤"[全部字段:智能] OR "烫伤"[全部字段:智能] OR "撕裂伤"[全部字段:智能] OR "挤压"[全部字段:智能] OR "火器伤"[全部字段:智能] OR "电击"[全部字段:智能]) AND( "超声"[全部字段:智能] OR "容量管理"[全部字段:智能] OR "容量控制"[全部字段:智能] OR "容量调节"[全部字段:智能] OR "液体置换"[全部字段:智能] OR "液体复苏"[全部字段:智能] OR "补液"[全部字段:智能])  The search was conducted from the establishment of the library to February 22, 2024, and **160** articles were retrieved. |

| **Search strategy of question 8: Complications** |
| --- |
| **PubMed** |
| (Complication*[Title/Abstract] OR "side effect*"[Title/Abstract]) AND ((Trauma*[Title/Abstract] OR Injur*[Title/Abstract] OR Wound*[Title/Abstract] OR burn*[Title/Abstract] OR Scald[Title/Abstract])) AND ((Extracorporeal Membrane Oxygenation*[Title/Abstract]) OR (Membrane Oxygenation, Extracorporeal[Title/Abstract]) OR (Oxygenation, Extracorporeal Membrane[Title/Abstract]) OR ECMO[Title/Abstract] OR ECLS[Title/Abstract] OR (Extracorporeal Life Support*[Title/Abstract]) OR (Life Support, Extracorporeal[Title/Abstract])) NOT (children[Title/Abstract] OR infant[Title/Abstract] OR child[Title/Abstract] OR pediatric[Title/Abstract] OR neonatal[Title/Abstract] OR baby[Title/Abstract] OR neonat[Title/Abstract] OR newborn[Title/Abstract] OR porcine[Title/Abstract] OR mouse[Title/Abstract] OR mice[Title/Abstract] OR rabbit[Title/Abstract] OR pigs[Title/Abstract])  The search was conducted from the establishment of the library to February 21, 2024, and **645** articles were retrieved. |
| **Embase** |
| #1:("side effect*"):ab,ti OR ((Complication*):ab,ti)  #2:Trauma*:ab,ti OR Injur*:ab,ti OR Wound*:ab,ti OR burn*:ab,ti OR Scald:ab,ti  #3('Extracorporeal Membrane Oxygenation*'):ab,ti OR (('Membrane Oxygenation, Extracorporeal'):ab,ti) OR (('Oxygenation, Extracorporeal Membrane'):ab,ti) OR ((ECMO):ab,ti) OR ((ECLS):ab,ti) OR (('Extracorporeal Life Support*'):ab,ti) OR (('Life Support, Extracorporeal'):ab,ti)  #4(child):ab,ti OR ((infant):ab,ti) OR ((child):ab,ti) OR ((pediatric):ab,ti) OR ((neonatal):ab,ti) OR ((baby):ab,ti) OR ((neonat):ab,ti) OR ((newborn):ab,ti) OR ((pig):ab,ti) OR ((mouse):ab,ti) OR ((mice):ab,ti) OR ((rabbit):ab,ti) OR ((pigs):ab,ti)  #1 AND #2 AND #3 NOT #4  The search was conducted from the inception of the library to February 21, 2024, and **1393** articles were retrieved. |
| **The Cochrane Library** |
| #1(Complication*):ti,ab,kw OR ("side effect*"):ti,ab,kw  #2(Trauma*):ti,ab,kw OR (Injur*):ti,ab,kw OR (Wound*):ti,ab,kw OR (burn*):ti,ab,kw OR (Scald):ti,ab,kw  #3(Extracorporeal Membrane Oxygenation*):ti,ab,kw OR (Membrane Oxygenation, Extracorporeal):ti,ab,kw OR (Oxygenation, Extracorporeal Membrane) OR (ECMO ):ti,ab,kw OR (ECLS):ti,ab,kw OR (Extracorporeal Life Support*):ti,ab,kw OR (Life Support, Extracorporeal):ti,ab,kw  #4(children):ti,ab,kw OR (infant):ti,ab,kw OR (child):ti,ab,kw OR (pediatric):ti,ab,kw OR (neonatal):ti,ab,kw OR (baby):ti,ab,kw OR (neonat):ti,ab,kw OR (newborn):ti,ab,kw OR (porcine):ti,ab,kw OR (mouse):ti,ab,kw OR (mice):ti,ab,kw OR (rabbit):ti,ab,kw OR (pigs):ti,ab,kw  #1 AND #2 AND #3 NOT #4  The search was conducted from the establishment of the library to February 21, 2024, and **64** articles were retrieved. |
| **China National Knowledge Infrastructure** |
| (TKA%=并发症 + 副作用 + 不良反应) AND (TKA%=体外模式氧合 + 体外膜式氧合 + 体外膜肺 + 膜肺氧合 + 体外膜氧合 + 体外生命支持 + ECMO + ECLS) AND (TKA%=创伤 + 坠落 + 车祸 + 多发伤 + 烧伤 + 钝挫 + 贯通 + 震爆 + 撞击 + 刺伤 + 切割 + 挫伤 + 烫伤 + 撕裂伤 + 挤压 + 火器伤 + 电击)  The search was conducted from the establishment of the library to February 21, 2024, and **110** articles were retrieved. |
| **Wanfang Database** |
| 主题:(ecmo or "体外膜肺" or "体外模式氧合" or "体外膜式氧合" or "膜肺氧合" or "体外膜氧合" or "体外生命支持" or ECLS) and 主题:(创伤 or 坠落 or 车祸 or 多发伤 or 烧伤 or 钝挫 or 贯通 or 震爆 or 撞击 or 刺伤 or 切割 or 挫伤 or 烫伤 or 撕裂伤 or 挤压 or 火器伤 or 电击) and 主题:(并发症 or 不良反应 or 副作用)  The search was conducted from the establishment of the library to February 21, 2024, and **63** articles were retrieved. |
| **VIP Information (Weipu database in Chinese)** |
| (U= ecmo+体外模式氧合+体外膜式氧合+体外膜肺氧合+体外膜肺+膜肺氧合+体外膜氧合+体外生命支持+ECLS) AND (U=创伤+坠落+车祸+多发伤+烧伤+钝挫+贯通+震爆+撞伤+刺伤+切割+挫伤+烫伤+撕裂伤+挤压+火器伤+电击) AND (U=并发症+不良反应+副作用)  The search was conducted from the establishment of the library to May 15, 2024, and **54** articles were retrieved. |
| **SinoMed** |
| ("并发症"[常用字段:智能] OR "副作用"[常用字段:智能] OR "不良反应"[常用字段:智能]) AND ("体外模式氧合"[常用字段:智能] OR "膜肺氧合"[常用字段:智能] OR "体外膜氧合"[常用字段:智能] OR "体外膜肺"[常用字段:智能] OR "体外膜氧合"[常用字段:智能] OR "ECMO"[常用字段:智能] OR "体外生命支持"[常用字段:智能] OR "ECLS"[常用字段:智能]) AND ("创伤"[常用字段:智能] OR "坠落"[常用字段:智能] OR "车祸"[常用字段:智能] OR "多发伤"[常用字段:智能] OR "烧伤"[常用字段:智能] OR "钝挫"[常用字段:智能] OR "贯通"[常用字段:智能] OR "震爆"[常用字段:智能] OR "撞击"[常用字段:智能] OR "刺伤"[常用字段:智能] OR "切割"[常用字段:智能] OR "挫伤"[常用字段:智能] OR "烫伤"[常用字段:智能] OR "撕裂伤"[常用字段:智能] OR "挤压"[常用字段:智能] OR "火器伤"[常用字段:智能] OR "电击"[常用字段:智能])  The search was conducted from the establishment of the library to February 21, 2024, and **254** articles were retrieved. |

| **Search strategy of Medlive.cn** |
| --- |
| 体外模式氧合（发布年限不限，语言种类不限）（48篇）  体外生命支持（发布年限不限，语言种类不限）（53篇）  膜肺氧合（发布年限不限，语言种类不限）（40篇）  体外膜式氧合（发布年限不限，语言种类不限）（16篇）  体外膜肺（发布年限不限，语言种类不限）（11篇）  体外膜氧合（发布年限不限，语言种类不限）（27篇）  ECMO（发布年限不限，语言种类不限）（35篇）  ECLS（发布年限不限，语言种类不限）（9篇） |

**Figure S1. Flow diagram of study inclusion**

Abstract screening for eligibility (n=7039)

**Database search：**

Question 1 (3937), Question 2 (578) Question 3 (1766), Question 4 (3463) Question 5 (658), Question 6 (734) Question 7 (845), Question 8 (2583)

Hand search (52)

Medlive.cn (135)

Total literature (n=14751)

Remove duplicate records: 7712

**Excluded**:

Not trauma patients: 3310

Not adults: 264

Inappropriate study design: 765

Studies less than five patients: 775

Studies unrelated to ECMO: 43

Subjects were not humans: 255

Irrelevant literature: 1488

Full-text inaccessible: 35

Finally included literature: 67

Full-text assessed for eligibility (n=104)

**Excluded:**

Not trauma patients: 2

Not adults: 2

Studies with fewer than five patients: 5

Studies unrelated to ECMO: 2

Irrelevant literature: 4

Over-lapped and duplicates: 22

**Identification**

**Screening**

**Eligibility**

**Included**

**Figure S1.** Flow diagram of study inclusion

**Table S3. Study characteristics**

| **Study_ID** | **1st author** | **Year of publication** | **Study period** | **Country/district(hospital)** | **Level I trauma center** | **Data bank** | **Study design** | **Grouping** | **Group** | **Sample size** | **Age (yrs)** | **Male (n)%** | **Injury mechanism** | **ECMO mode (n)** | **ISS** | **survival to hospital discharge (n)** |
| --- | --- | --- | --- | --- | --- | --- | --- | --- | --- | --- | --- | --- | --- | --- | --- | --- |
| 2 | Abate | 2022 | 2010.1–2016.12 | America | NA | TQIP | case-control study | Survivors vs Nonsurvivors | Survivors | 226 | 28(median) (IQR 20) | 81.90% | NA | NA | 26(median) (IQR 17) | 226 |
| 2 | Abate | 2022 | 2010.1–2016.12 | America | NA | TQIP | case-control study | Survivors vs Nonsurvivors | Nonsurvivors | 136 | 34(median) (IQR 30) | 89.70% | NA | NA | 29(median) (IQR 21) | 0 |
| 7 | Austin | 2024 | 2014.1-2022.8 | America | NA | NA | case-control study | Survivors vs Nonsurvivors | Survivors | 26 | 28.5 (21, 40) | 51.00% | Blunt 100%, Penetrating 0% | VV ECMO 26 | 27 (17, 34) | 26 |
| 7 | Austin | 2024 | 2014.1-2022.8 | America | NA | NA | case-control study | Survivors vs Nonsurvivors | Nonsurvivors | 10 | 30.5 (24, 37) | 80.00% | Blunt 90%, Penetrating 10% | VV ECMO 10 | 34.5 (29, 50) | 0 |
| 7 | Austin | 2024 | 2014.1-2022.8 | America | NA | NA | case-control study | TBI VS nonTBI | TBI | 36 | 29 (23.5, 39) | 81.00% | Blunt 97%, Penetrating 3% | VV ECMO 36 | 29.5 (20, 38) | 26 |
| 7 | Austin | 2024 | 2014.1-2022.8 | America | NA | NA | case-control study | TBI VS nonTBI | nonTBI | 39 | NA | NA | NA | VV ECMO 39 | NA | 25 |
| 11 | Christopher | 2017 | 1999.1-2015.12 | NA | NA | ELSO international registry | case-control study | Survivors vs Nonsurvivors | Survivors | 25 | 30 (24, 46) | 72.00% | Burn 100% | VV ECMO 22, VA ECMO 3 | NA | 25 |
| 11 | Christopher | 2017 | 1999.1-2015.12 | NA | NA | ELSO international registry | case-control study | Survivors vs Nonsurvivors | Nonsurvivors | 33 | 39 (28, 52) | 78.10% | Burn 100% | VV ECMO 22, VA ECMO 11 | NA | 0 |
| 14 | Mehran Dadras | 2019 | 2017.1-2019.1 | Germany | NA | NA | case series | Single arm study | NA | 8 | 48(34,58) | 75% | burn（100%） | VV ECMO （8） | NA | 5 |
| 16 | Fouché | 2023 | 2017.4-2022.6 | America | NA | NA | case series | Single arm study | NA | 12 | 39.5(25.75, 50.75) | 75% | Burn (100%) | VV （9） VA （1） VV-to-VA（2） | NA | 8 |
| 20 | Hsu | 2016 | NA | Taiwan, China | NA | NA | case series | Single arm study | NA | 6 | 43.3 ± 11.6 | 83% | Burn (100%) | VV-ECMO （2） VA-ECMO (4) | NA | 1 |
| 23 | Yao-Kuang Huang | 2009 | 2004.3-2007.10 | Taiwan, China | NA | NA | case series | Single arm study | NA | 9 | 37(26.5, 44) | NA | Blunt (88.9%) Penetrating(11.1%) | VV-ECMO (7) VA-ECMO (2) | 44.56 ± 4.93 | 7 |
| 28 | Eunji Kim | 2024 | 2011.11~2022.01 | Pusan National University School of Medicine, Korea. | Yes | NA | Cohort study | ECLS or no ECLS | No ECLS | 18 | 47.50(34.25,58.50) | 72.20% | Blunt 61.1%, Penetrating injury 38.9% | NA | 30.00 (13.25,40.25) | 15 |
| 28 | Eunji Kim | 2024 | 2011.11~2022.01 | Pusan National University School of Medicine, Korea. | Yes | NA | Cohort study | ECLS or no ECLS | ECLS | 10 | 53.00(38.25, 73.50) | 80% | Blunt 80%, Penetrating injury 20% | NA | 27.50 (16.25, 35.00) | 6 |
| 28 | Eunji Kim | 2024 | 2011.11~2022.01 | Pusan National University School of Medicine, Korea. | Yes | NA | Cohort study | survival or death | Survivors | 21 | 45.00 (34.00, 55.00) | 76.20% | Blunt 61.9%, Penetrating injury 38.1% | NA | 25.00 (13.00, 34.00) | 21 |
| 28 | Eunji Kim | 2024 | 2011.11~2022.01 | Pusan National University School of Medicine, Korea. | Yes | NA | Cohort study | survival or death | death | 7 | 67.00(60.50, 81.50) | 71.40% | Blunt 85.7%, Penetrating injury 14.3% | NA | 35.00 (30.00, 59.00) | 7 |
| 29 | Daniel Lammers | 2023 | 2017~2019 | America | Yes(The vast majority of centers performing ECMO were ACS level I trauma centers (91.7%)) | American College of Surgeons (ACS) Trauma Quality Improvement Program (TQIP) database | Cohort study | ECMO within 24 hours VS after 24 hours | Within 24 Hours | 234 | 32.6(mean) | 86.40% | Penetrating injury 9% | NA | 30.7(mean) | 135 |
| 29 | Daniel Lammers | 2023 | 2017~2019 | America | Yes(The vast majority of centers performing ECMO were ACS level I trauma centers (91.7%)) | American College of Surgeons (ACS) Trauma Quality Improvement Program (TQIP) database | Cohort study | ECMO within 24 hours VS after 24 hours | After 24 Hours | 464 | 36.2(mean) | 80.20% | Penetrating injury 3.4% | NA | 27.6(mean) | 316 |
| 30 | Alex Lee | 2023 | 2014.01~2021.02 | British | Yes | British Columbia Trauma Registry (BCTR) | case series | single arm study | NA | 25 | 38.8(mean) (range, 19–67) | 76% | Blunt 80%, Penetrating injury 20% | VV-ECMO (18), VA-ECMO (7) | 40(mean) (range, 22–57) | 15 |
| 31 | Joseph E. Marcus | 2019 | 2012.09~2018.05 | Brooke Army Medical Center | NA | NA | case series | Single-arm study | NA | 15 | 31 (21, 54) | 73.33% | Burn 100% | VV-ECMO 15 | NA | 7 |
| 35 | Natthida Owattanapanich | 2021 | 2007-2017 | NA | No | National Trauma Data Bank | cohort study | Resuscitative thoracotomy(RT) alone VS RT with immediate ECMO | RT alone | 4034 | 31 （23,46） | 85.70% | Gunshot wound 44.5%, Traffic accident 33.2%, Penetrating injury 16.8%, Fall 3.4%, Other 2.1% | NA | 25 (17,38) | 1472 |
| 35 | Natthida Owattanapanich | 2021 | 2007-2017 | NA | No | National Trauma Data Bank | cohort study | Resuscitative thoracotomy(RT) alone VS RT with immediate ECMO | RT with immediate ECMO | 23 | 23（17,33） | 87% | Gunshot wound 47.8%, Traffic accident 8.7%, Penetrating injury 43.5%, Fall 0%, Other 0% | NA | 25(25,43) | 11 |
| 36 | Steven Neubauer | 2023 | 2018.03~2020.03 | St Elizabeth Healthcare Center | Yes | NA | case series | Single arm study | NA | 5 | 27 (22.5,51) | 100% | Traffic accident 80%, Gunshot wound 20% | VV-ECMO 5 | 29 (26.5,33.5) | 5 |
| 37 | Joep J.J. Ouwerkerk | 2023 | 2010~2019 | US/ Massachusetts | NA | Trauma Quality Improvement Program database | case-control study | Survivors vs Nonsurvivors | Survivors | 337 | 29.0 (22.0, 45.0) | 82.80% | Blunt 72.7%, Penetrating injury 16.9%, Mixed injury 6.2%, Other 4.2% | NA | 27.0 (17.0, 35.0) | 337 |
| 37 | Joep J.J. Ouwerkerk | 2023 | 2010~2019 | US/ Massachusetts | NA | Trauma Quality Improvement Program database | case-control study | Survivors vs Nonsurvivors | Nonsurvivors | 205 | 32.0 (23.0, 54.0) | 88.30% | Blunt 69.8%, Penetrating injury 17.1%, Mixed injury 7.8%, Other 5.4% | NA | 29.0 (22.0, 43.0) | 0 |
| 38 | Michael J. Perchinsky, | 1995 | NA | Emanuel Hospital and Health Center | NA | NA | case series | Single arm study | NA | 6 | NA | NA | Traffic accident (3), Fall (1), Gunshot wound (2) | NA | mean53 (range, 26-75) | 3 |
| 41 | Soussi | 2016 | 2013.1~2016.1 | Paris, France | NA | NA | cohort study | ECMO VS non-ECMO | ECMO | 11 | 51(40，60) | 90.90% | Burn 100% | VA-ECMO (2) VV-ECMO (8) VA-ECMO＋VV-ECMO (1) | NA | 1 |
| 41 | Soussi | 2016 | 2013.1~2016.1 | Paris, France | NA | NA | cohort study | ECMO VS non-ECMO | non-ECMO | 71 | 56(41,68) | NA | Burn 100% | 0 | NA | 35 |
| 45 | Tecos | 2023 | 2017.1~2022.4 | Omaha, US | Yes | ELSO | case series | Single arm study | NA | 12 | 34.25（13.8-64.0） | 66.70% | NA | VA-ECMO (3) VV-ECMO (9) | NA | 5 |
| 50 | Chen | 2022 | 2000.1~2021.12 | China | NA | NA | cohort study | ECMO VS Ventilator | Ventilator | 21 | 36. 3±10. 2 | 61.90% | blast lung injury 100% | 0 | NA | 15 |
| 50 | Chen | 2022 | 2000.1~2021.12 | China | NA | NA | cohort study | ECMO VS Ventilator | ECMO | 16 | 39. 2±11. 1 | 62.50% | blast lung injury 100% | VV-ECMO (16) | NA | 14 |
| 51 | Liu | 2023 | 2019.7~2022.6 | China | NA | NA | case series | Single arm study | NA | 13 | 43.8±17.6 | 92.30% | NA | VV-ECMO (13) | 33.9±7.1 | 10 |
| 52 | Fei | 2023 | 2019.8~2022.8 | China | NA | NA | case series | Single arm study | NA | 8 | 42.857±21.318 | 87.50% | Traffic accident 62.5%，Crush injury 37.5% | VA-ECMO (4) VV-ECMO (4) | 31.286±20.508 | 7 |
| 53 | Jiang | 2005 | 1997.3~2005.3 | China | NA | NA | cohort study | ECMO VS non-ECMO | ECMO | 12 | 49.04±12.09 | 83.30% | Multiple injuries 83.3% Multiple fractures 16.7% | VA-ECMO 12 | 33.08±3.90 | NA |
| 53 | Jiang | 2005 | 1997.3~2005.3 | China | NA | NA | cohort study | ECMO VS non-ECMO | non-ECMO | 14 | NA | NA | Multiple injuries 100% | 0 | 31.57±4.20 | NA |
| 54 | Li | 2021 | 2014.3-2020.7 | China/the First Affiliated Hospital of Army  Medical University | NA | NA | case series | Single-arm study | NA | 5 | 48.2±7.5736 | 100% | Burn(100%) | VV-ECMO 5 | NA | 1 |
| 55 | Xie | 2005 | 2002.6-2004.8 | China/Zhongshan People's Hospital | NA | NA | case series | Single-arm study | NA | 17 | 23.3±0.7 | 64.70% | traffic injuries （76.5%） , Fall injuries（ 23.5%） | VV-ECMO | 24.3±7.5 | 9 |
| 61 | Anderson | 1994 | 1988.5-1993.8 | the University of Michigan Medical center | NA | NA | case series | suvivors VS. nonsurvivors | survivors | 15 | NA | NA | Traffic accident （93.3%), Gunshot wound (6.7%) | VA-ECMO 3, VV-ECMO 8, VA-ECMO-to-VV-ECMO 3、VV-ECMO-to-VA-ECMO 3 | NA | 15 |
| 61 | Anderson | 1994 | 1988.5-1993.8 | the University of Michigan Medical center | NA | NA | case series | suvivors VS. nonsurvivors | Nonsurvivors | 9 | NA | NA | Traffic accident(66.7%）, Gunshot wound（ 22.2%）, Penetrating （ 11.1%） | VA-ECMO 1, VV-ECMO 5, VV-ECMO-to-VA-ECMO 3 | NA | 9 |
| 62 | Senunas | 1997 | 1988-1994 | the University of Michigan Medical Center | NA | NA | case series | Single-arm study | NA | 14 | 19.07±9.34 | 28.60% | Traffic accident (100%) | NA | 19.2857±7.3914 | 8 |
| 64 | Cordell-Smith | 2006 | 1992.5-2000.11 | England | yes | NA | case-control study | suvivors VS. nonsurvivors | survivors | 20 | 27(mean) | NA | NA(总体：Traffic accident 85.7%, Sports accidents 7.1%, Crush injury7.1%) | VV-ECMO 20 | 19 | 20 |
| 64 | Cordell-Smith | 2006 | 1992.5-2000.11 | England | yes | NA | case-control study | suvivors VS. nonsurvivors | Nonsurvivors | 8 | 28(mean) | NA | NA(总体：Traffic accident 85.7%, Sports accidents 7.1%, Crush injury7.1%) | VV-ECMO 4, VV-ECMO-to-VA-ECMO 4 | 14 | 0 |
| 67 | Bein | 2012 | 2005.6-2011.8 | America | yes | NA | case series | PECLA VS VV ECMO | VV-ECMO | 5 | 23.4 ± 3.647 | NA | traffic accident(1); bombing with traumatic brain injury(1);gunshot wound(3) | VV ECMO 5 | 24.8± 12.28 | 5 |
| 68 | Biderman | 2013 | NA | Israel | NA | Rabin Medical Center | case series | ECMO VS iLA | ECMO | 5 | 28.4±4.39 | 60.00% | fall(20%); Traffic accident(60%),bombing(20%） | NA | 52.4±10.26 | 3 |
| 69 | Bonacchi | 2012 | 2008.12-2012.5 | Careggi Teaching Hospital, Florence, Italy | NA | NA | case-control study | ECLS success VS ECLS failure | ECLS success | 14 | 47±17.6 | 71.00% | Blunt 100% | VA-ECMO(10)，VV-ECMO(4) | 46.5± 16.3 | 5 |
| 69 | Bonacchi | 2012 | 2008.12-2012.5 | Careggi Teaching Hospital, Florence, Italy | NA | NA | case-control study | ECLS success VS ECLS failure | ECLS failure | 4 | 43±19.7 | 50.00% | Blunt 100% | VV-ECMO(4) | 65±9.6 | 0 |
| 70 | Ried | 2013 | 2002.04-2012.04 | Regensburg, Germany | yes | Regensburg ECMO Registry database | cohort study | PECLA VS VV ECMO | VV-ECMO | 26 | 29.3 ± 13.2 | 92.00% | traffic accident (81%)  Blast injury/gunshot wound(11%)  fall (4%) Blunt (4%) | vv-ECMO(26) | 59.4 ± 11.2 | 21 |
| 71 | Guirand | 2014 | 2001.01-2009.12 | America | yes | Wake Forest School of Medicine ECLS Registry. Los Angeles County + University of Southern California (LAC + USC) Medical Center trauma and SICU database. | cohort study | VV ECLS vs CONV | VV-ECMO | 17 | 30.9 ± 11.4 | 71% | blunt (88%) | **VV-ECMO(17)** | 30.6 ±14.4 | 11 |
| 71 | Guirand | 2014 | 2001.01-2009.12 | America | yes |  | cohort study | VV ECLS vs CONV | CONV | 17 | 34.1 ± 10.7 | 88% | blunt(65%) | 0 | 29.4± 13.1 | 4 |
| 72 | Tseng | 2014 | 2003.11-2012.10 | Taiwan, China | NA | NA | case series | single arm study | NA | 9 | 37（26.5,46） | 88.90% | high-voltage electrocution (11.1%), penetrating chest trauma (11.1%), and blunt chest or poly-trauma (77.8%) | VA-ECLS(9) | 34（15.5,41） | 3 |
| 73 | Wu | 2014 | 2004.1-2013.6 | Taiwan, China，Chang Gung Memorial Hospital | yes | NA | case-control study | Survivors vs Nonsurvivors | Survivors | 14 | 41 (29,57) | NA | Accidental fall(14.3%),traffic accident(78.6%),crush injury(7.1%) | VV ECMO 14 | 29 (19–43) | 14 |
| 73 | Wu | 2014 | 2004.1-2013.6 | Taiwan, China，Chang Gung Memorial Hospital | yes | NA | case-control study | Survivors vs Nonsurvivors | Nonsurvivors | 6 | 30 (22,61) | NA | Accidental fall(16.7%),traffic accident(83.3%) | VV ECMO 6 | 63 (26–75) | 0 |
| 73 | Wu | 2014 | 2004.1-2013.6 | Taiwan, China，Chang Gung Memorial Hospital | yes | NA | case-control study | Patient with hemorrhagic complication VS Patient without hemorrhage complications | Patient with hemorrhagic complication | 7 | 33 (23,53) | NA | NA | VV ECMO 7 | 29 (18–75) | 4 |
| 73 | Wu | 2014 | 2004.1-2013.6 | Taiwan, China，Chang Gung Memorial Hospital | yes | NA | case-control study | Patient with hemorrhagic complication VS Patient without hemorrhage complications | Patient without hemorrhage complications | 13 | 43 (28,58) | NA | NA | VV ECMO 13 | 41 (24–47) | 10 |
| 74 | Bosarge | 2016 | 2012.03-2014.11 | Birmingham, Alabama, University of Alabama at Birmingham (UAB) | YES | NA | cohort study | ECMO VS CONV | ECMO | 15 | 36.0 (25.0 ,47.0) | 100% | NA | Venovenous (V/V) in 10 patients, venoarterial (V/A) in 3, venoarterial venous (V/A/V) in 2 | 26.0 (17.0, 34.0) | 13 |
| 74 | Bosarge | 2016 | 2012.03-2013.2 | Birmingham, Alabama, University of Alabama at Birmingham (UAB) | YES | NA | cohort study | ECMO VS CONV | CONV | 14 | 40.0 (23.0, 47.0) | 92.90% | NA | 0 | 25.0 (25.0 , 33.0) | 5 |
| 75 | Wu | 2015 | 2008.1~2014.1 | Taichung,China | NA | NA | case-control study | Survivors VS Nonsurvivors | Survivors | 13 | 33.8±16.4 | 100% | traffic accident(84.6%),Fall(15.4%) | VV-ECMO(8),VA-ECMO(5) | 29.0 (25, 29) | 13 |
| 75 | Wu | 2015 | 2008.1~2014.1 | Taichung,China | NA | NA | case-control study | Survivors VS Nonsurvivors | Nonsurvivors | 6 | 55.8±14.7 | 66.70% | traffic accident (100%) | VV-ECMO(1),VA-ECMO(5) | 40.5 (25, 50) | 0 |
| 76 | Chen | 2016 | 2009.9~2012.9 | Taipei, China | NA | NA | case series | Single arm study | NA | 7 | 31 (21,49) | 85.70% | traffic accident ( 71.4%) , fall (28.6%) | VV-ECMO(7) | 36(27,57) | 4 |
| 77 | Kim | 2016 | 2007.1~2015.3 | Anyang,Korea | NA | NA | case series | trauma VS nontrauma | trauma | 9 | 48.0 (20.5,62.0) | 88.90% | traffic accident（44.4%）; Gunshot wound（11.1%）; Crush injury（ 22.2%）; Fall(22.2%) | VV-ECMO(9) | NA | 8 |
| 78 | Ull | 2017 | 2008.1~2014.2 | Bochum,Germany | YES | retrospective review of medical records;ECLS value databases;TraumaRegister of the German Trauma Society | case series | trauma VS nontrauma | trauma | 49 | 49.9 (16.6–86.2) | 89.80% | NA | VV-ECMO(34),VA-ECMO(4),PECLA(14) | 32 ( 4–66) | 32 |
| 79 | Ahmad | 2017 | 2006.1~2015.11 | Baltimore,Maryland | YES | NA | case-control study | Survivors vs Nonsurvivors | survivors | 17 | 35 (25, 45) | 62% | Blunt, 65%; Penetrating injury, 29%; Drown, 6% | VV-ECMO(17) | 25(18, 32) | 17 |
| 79 | Ahmad | 2017 | 2006.1~2015.11 | Baltimore,Maryland | YES | NA | case-control study | Survivors vs Nonsurvivors | Nonsurvivors | 22 | 27 (22, 42) | 82% | Blunt, 82%; Penetrating injury, 14%; Crush injury, 5% | VV-ECMO(22),VA-ECMO(7) | 41 (26, 50) | 0 |
| 80 | Burke | 2017 | 2012.1 ~ 2014.12 | Seattle,WA, United States | YES | the National Trauma Data Bank | case-control study | Survivors vs Nonsurvivors | survivors | 51 | 24 (19, 36) | 86% | Penetrating injury 14% Blunt 80% Other mechanism 6%) | NA | 24 (10, 30) | 51 |
| 80 | Burke | 2017 | 2012.1~ 2014.12 | Seattle,WA,United States | YES | the National Trauma Data Bank | case-control study | Survivors vs Nonsurvivors | Nonsurvivors | 29 | 34 (19, 50) | 83% | Penetrating injury 7% Blunt 86% Other mechanism 7% | NA | 29 (21, 38) | 0 |
| 81 | Huh | 2017 | 2015.4 ~2016.5 | Busan, Korea | YES | NA | case series | Single arm study | NA | 5 | 39.4±8.53 | 80% | Traffic accident 60%, Penetrating injury 20%, Fall 20% | NA | 33.8±12.26 | 5 |
| 82 | Lin | 2017 | 2006.3 ~2016.7 | Taiwan, China | NA | NA | case-control study | Survivors VS.Mortality | Survivors | 22 | 40.5±14.9 | 86.40% | Traffic accident (%) 77.3  Fall (%) 13.6 Burn (%) 4.5 Violence (%)4.5 | VV-ECMO(22) | 23.4±9.2 | 22 |
| 82 | Lin | 2017 | 2006.3 ~2016.7 | Taiwan, China | NA | NA | case-control study | Survivors VS.Mortality | Mortality | 21 | 34.1±15.1 | 81% | Traffic accident (%) 61.9 Fall (%) 23.8 Burn (%)14.3 Violence (%) 10.1 | VA-ECMO(17)、VV-ECMO(4) | 35.3±11.4 | 0 |
| 83 | Nosanov | 2017 | 2002-2011 | America | No | National Burn Repository (version 8.0) | Cohort study | ECMO VS. No ECMO | ECMO | 30 | 38.9 ± 20.3 | 80.00% | Burn (100%) | NA | NA | 14 |
| 83 | Nosanov | 2017 | NA | America | No | National Burn Repository (version 8.0) | Cohort study | ECMO VS. No ECMO | No ECMO | 30 | 39.2 ± 20.3 | 80.00% | Burn (100%） | NA | NA | 26 |
| 85 | Grant | 2018 | 2016.6-2017.9 | America | Yes | NA | Cohort study | pre-Advanced ECMO Program vs. post-Advanced ECMO Program | post-Advanced ECMO Program | 12 | 36.5 | 83% | Fall 25%, Burn 8.33%, Traffic accident 33.33%, Crush injury 8.33%, Penetrating injury 16.67%, Gunshot wound 8.33% | VV-ECMO 11, VA-ECMO 1 | 34(median) | 6 |
| 85 | Grant | 2018 | 2014.1-2016.5 | America | Yes | NA | Cohort study | pre-Advanced ECMO Program vs. post-Advanced ECMO Program | pre-Advanced ECMO Program | 7 | 30 | 86% | Blunt 14.29%, Burn 14.29%, Traffic accident 42.86%, Gunshot wound 28.57% | VV-ECMO 5, VA-ECMO 2 | 30(median) | 3 |
| 86 | Ainsworth | 2018 | 2012.9-2017.9 | America | No | NA | case series | Single arm study | NA | 11 | 37.45±15.58 | 81.20% | Burn 100% | VV-ECMO 11 | NA | 5 |
| 89 | Strumwasser | 2018 | 2016.12~2017.12 | America | No | NA | case series | Single arm study | NA | 7 | 41±15.03 | 100% | Traffic accident 71.43%, Gunshot wound 28.57% | VV-ECMO 5, VA-ECMO 2 | 32.57±14.07 | 2 |
| 90 | Szentgyorgyi | 2018 | 2011.12-2017.6 | Wythenshawe Hospital | NA | NA | case series | Single arm study | NA | 5 | 33(29,35.5) | 60% | burns100% | VV-ECMO 5 | NA | 4 |
| 92 | Kruit | 2019 | 2011.12-2017.5 | the United Kingdom | No | the five National Respiratory ECMO centers in the United Kingdom | case-control study | Hemorrhagic Complication VS No Hemorrhagic Complication | Hemorrhagic Complication | 26 | 30 (24, 37) | NA | NA | NA | 39 (28, 57) | NA |
| 92 | Kruit | 2019 | 2011.12-2017.5 | the United Kingdom | No | the five National Respiratory ECMO centers in the United Kingdom | case-control study | Hemorrhagic Complication VS No Hemorrhagic Complication | No Hemorrhagic Complication | 26 | 36 (27, 50) | NA | NA | NA | 27 (21, 51) | NA |
| 92 | Kruit | 2019 | 2011.12-2017.5 | the United Kingdom | No | the five National Respiratory ECMO centers in the United Kingdom | case-control study | Survivors VS Deaths | Survivors | 44 | 33 (23, 45) | 84% | Traffic accident 75% ; Fall 16% ; Assault 9% | NA | 36(24,57) | NA |
| 92 | Kruit | 2019 | 2011.12-2017.5 | the United Kingdom | No | the five National Respiratory ECMO centers in the United Kingdom | case-control study | Survivors VS Deaths | Deaths | 8 | 32 (26, 37) | 63% | Traffic accident 75% ; Fall 12.5% ; Mising data 12.5% | NA | 29(26,33) | NA |
| 93 | Lang | 2019 | 2002-2016 | Medical University of Vienna | Yes | NA | case-control study | Survivors vs Nonsurvivors | Survivors | 4 | 26.25±8.88 | 0.00% | Traffic accident 50%, Penetrating injury 25%, Blunt 25% | VA-ECMO 4 | 29.75±16.338 | NA |
| 93 | Lang | 2019 | 2002-2016 | Medical University of Vienna | Yes | NA | case-control study | Survivors vs Nonsurvivors | Nonsurvivors | 12 | 37.583±15.957 | 41.70% | Traffic accident 50%, Fall 33.3%, Gunshot wound 8.3%, Penetrating injury 8.3% | VV-ECMO 1, VA-ECMO 11 | 35.917±11.139 | NA |
| 94 | Lee | 2020 | 2007.01-2018.12 | Hallym University Sacred Heart Hospital | NA | NA | case-control study | Survivors vs Nonsurvivors | NA | 42 | 41 (18.75,52.75) | 88.10% | traffic accident 21.4%, Near drowning 42.9%, Gunshot wound 2.4%, Intoxication 4.8%, Crushing injury 11.9%, Fall 9.5%, Hanging 2.4%, penetrating injury4.8% | VV-ECMO 29, Other types 13 | NA | 34 |
| 94 | Lee | 2020 | 2007.01-2018.12 | Hallym University Sacred Heart Hospital | NA | NA | case-control study | Survivors vs Nonsurvivors | Survivors | 34 | 39(18.75, 48.0) | 85.30% | traffic accident 23.5%, Near drowning 44.1%, Gunshot wound 2.9%, Intoxication 2.9%, Crushing injury 8.8%, Fall 11.8%, Hanging 2.9%, penetrating injury2.9% | VV-ECMO 28, Other types 6 | NA | 34 |
| 94 | Lee | 2020 | 2007.01-2018.12 | Hallym University Sacred Heart Hospital | NA | NA | case-control study | Survivors vs Nonsurvivors | Nonsurvivors | 8 | 49.5(24.5, 59.0) | 100% | traffic accident 12.5%, Near drowning 37.5%, Intoxication 12.5%, Crushing injury 25%, penetrating injury 12.5% | VV-ECMO 1, Other types7 | NA | 0 |
| 95 | Akhmerov | 2020 | 2007-2015 | America | level I or II trauma centers | National Trauma Data Bank | case series | Single arm study | NA | 522 | 32.0 ± 17.4 | 80.80% | Blunt 69.6%, Penetrating injury 15%, Burn 8.1%, Other 8.1% | NA | 26.8 ± 15.1 | 209 |
| 97 | Amos | 2021 | 2010.01-2020.06 | The Alfred | NA | NA | cohort study | Early initiation of ECMO ( < 72 hours) VS Late ( > 72 hours) | NA | 11 | 39±17.8 | 91% | traffic accident 73%；fall18%；Pulmonary blast injury9%。 | VV 7, VA 3, VV to VAV to VV ECMO 1 | 50(34,54) | 5 |
| 97 | Amos | 2021 | 2010.01-2020.06 | The Alfred | NA | NA | cohort study | Early initiation of ECMO ( < 72 hours) VS Late ( > 72 hours) | Early initiation of ECMO ( < 72 hours) | 6 | NA | NA | traffic accident 50%；fall 33%；Pulmonary blast injury17%。 | VV ECMO 3, VA/VAV ECMO 3 | 42.5 | 4 |
| 97 | Amos | 2021 | 2010.01-2020.06 | The Alfred | NA | NA | cohort study | Early initiation of ECMO ( < 72 hours) VS Late ( > 72 hours) | Late ( > 72 hours) | 5 | NA | NA | traffic accident 100%； | VV ECMO 4, VA/VAV ECMO 1 | 50 | 1 |
| 99 | Parker | 2021 | NA | USA | NA | NA | case series | Survivors vs Nonsurvivors | NA | 13 | 28（25,37.5） | 85% | traumatic brain injury （100%） | VV-ECMO (13) | 48(33.5,66) | 5 |
| 99 | Parker | 2021 | NA | USA | NA | NA | case series | Survivors vs Nonsurvivors | Survivors | 5 | NA | NA | traumatic brain injury （100%） | VV-ECMO (5) | NA | 5 |
| 99 | Parker | 2021 | NA | USA | NA | NA | case series | Survivors vs Nonsurvivors | Nonsurvivors | 8 | NA | NA | traumatic brain injury （100%） | VV-ECMO (8) | NA | 0 |
| 102 | Thani | 2022 | 2014.01-2020.01 | Qatar | Yes | NA | Cohort study | ECMO VS non-ECMO | ECMO | 22 | 29.6 ± 13.8 | 86.40% | Traffic accident （81.8%），fall （13.6%）， Struck by a heavy Object(4.5%) | VV-ECMO (21), VA-ECMO (1) | 30.6±12.3 | NA |
| 102 | Thani | 2022 | 2014.01-2020.01 | Qatar | Yes | NA | Cohort study | ECMO VS non-ECMO | non-ECMO | 63 | 35.9 ± 15.1 | 93.70% | Traffic accident （63.5%） Fall (20.6%) Struck by a heavy Object(3.2%) Assault(6.3%) Others(6.3%) | 0 | 30.3±14.1 | NA |
| 103 | Brewer | 2022 | 2013.10-2020.2 | USA/Connecticut | Yes | NA | case series | emergency general surgery VS trauma patients | Trauma ECMO patients | 12 | 33.6 ± 4.0 | NA | NA | VV-ECMO 11, VA-ECMO 1 | 27.6 ± 6.0 | 10 |
| 104 | Eisenga | 2022 | 2012.7-2021.6 | USA | Yes | NA | case series | single arm study | NA | 10 | 33±12.99 | NA | Penetrating injury（100%） | VA-3, VV 5, VV-to-VA 2 | NA | 7 |
| 105 | Lee | 2022 | 2013.1-2017.12 | Korea | NA | NA | Cohort study | VV-ECMO VS CMV groups | VV-ECMO | 16 | 47.5 (34.3, 71.3) | 81.30% | Traffic accidents（74.9%) ;Falls (12.5%);Crushing (6.3%) ;Others (6.3%) | VV ECMO16 | 23.5 (10.8, 29.0) | 9 |
| 105 | Lee | 2022 | 2013.1-2017.12 | Korea | NA | NA | Cohort study | VV-ECMO VS CMV groups | Conventional mechanical ventilation | 32 | 58.0（49.0,70.5） | 65.60% | Traffic accidents（75.0%) ;Falls (15.6%);Others (9.4%) | 0 | 17.5（9.0，25.0） | 15 |
| 106 | Ismael A Salas De Armas | 2022 | 2015.6-2018.8 | US/TX | Yes | NA | case series | single arm study | NA | 15 | 30.6±12.6 | 93% | Traffic accident（ 93%）, Fall （6.7%,） | VV-ECMO （15） | 34.1±11.5 | 13 |
| 107 | Jaimin R. TRivedi | 2022 | 2016-2019 | US/Kentucky | Yes | NA | case series | single arm study | NA | 7 | 32.1±8.7 | 100% | Blunt 100% | VA-ECMO （2） 、VV-ECMO （5） | NA | 5 |
| 108 | Weidemann | 2022 | 2011.4-2019.4 | German/Hannover | Yes | NA | case series | Survivors vs Nonsurvivors | NA | 19 | 28±11 | 78.90% | Traffic accident （73.68%）, Fall （15.79%）, Penetrating injury （5.26%）, Blunt （5.26%） | VV-ECMO (13), VA-to-VV (5),VAV-to-VV (1) | 45±13 | 10 |
| 108 | Weidemann | 2022 | 2011.4-2019.4 | German/Hannover | Yes | NA | case series | Survivors vs Nonsurvivors | Survivors | 10 | 28.42±11.39 | 80% | Traffic accident 80%, Fall 10%, Blunt 10% | VV(6), VA-to-VV (3),VAV-to-VV (1) | 44.4±13.7 | 10 |
| 108 | Weidemann | 2022 | 2011.4-2019.4 | German/Hannover | Yes | NA | case series | Survivors vs Nonsurvivors | Nonsurvivors | 9 | 29.25±12.34 | 77.80% | Traffic accident （66.67%） ，Fall （22.22%）， Penetrating injury（ 11.11%） | VV (7), VA -to-VV (2) | 45.9±12.9 | 0 |
| 110 | Jordan Hatfield, BS | 2023 | 2016.1-2020.6 | US/NC | No | Premier Healthcare Database (PH | cohort study | ECMO VS. No ECMO | No ECMO | 59612 | 56 (35,72) | 71.10% | traumatic brain injury 100% | 0 | 17 (16,29) | 42485 |
| 110 | Jordan Hatfield, BS | 2023 | 2016.1-2020.6 | US/NC | No | Premier Healthcare Database (PH | cohort study | ECMO VS. No ECMO | ECMO | 118 | 30 (22,46) | 78.00% | traumatic brain injury （100%） | NA | 29 (17,41) | 78 |
| 112 | Marius Marc-Daniel Mader | 2023 | 2015-2019 | German | NO | TraumaRegister DGU | cohort study | ECMO VS. No ECMO | ECMO | 134 | 49.8±20.1 | 80% | Penetrating injury (0.8%) Severe head injury (AIS 4+) (65%) Isolated head injury (13%) Chest trauma (AIS 3+) (64%) Abdominal trauma (AIS 3+) (19%) Extremity/pelvic trauma (AIS 3+) (34%) Polytrauma (Berlin definition) (58%) | NA | 35.9±14.6 | 83 |
| 112 | Marius Marc-Daniel Mader | 2023 | 2015-2019 | German | NO | TraumaRegister DGU | cohort study | ECMO VS. No ECMO | No ECMO | 12113 | 56.6±21.2 | 70% | Penetrating injury 1.6% Severe head injury (AIS 4+) 62% Isolated head injury 35% Chest trauma (AIS 3+) 30% Abdominal trauma (AIS 3+) 5% Extremity/pelvic trauma (AIS 3+) 14% Polytrauma (Berlin definition) 27% | 0 | 24.6±11.2 | 10590 |
| 113 | Elizabeth K. Powell | 2023 | 2014.1.1-2022.8.1 | US/MD | No | NA | cohort study | Early VV ECMO VS.Non Early VV ECMO | Non Early VV ECMO | 18 | NA | NA | NA | VV (18) | NA | 11 |
| 113 | Elizabeth K. Powell | 2023 | 2014.1.1-2022.8.1 | US/MD | No | NA | cohort study | Early VV ECMO VS.Non Early VV ECMO | Early VV ECMO | 57 | 29 (22,40) | 81% | Penetrating injury （21%） Blunt （79%） | VV (57) | 33.4 ±17 | 40 |
| 113 | Elizabeth K. Powell | 2023 | 2014.1.1-2022.8.1 | US/MD | No | NA | cohort study | Early ECMO suvivors VS. nonsurvivors | Early ECMO suvivors | 40 | 29 (23.5,42） | 80% | Penetrating injury （25%） Blunt （75%） | VV (40) | 34 (21,45) | 40 |
| 113 | Elizabeth K. Powell | 2023 | 2014.1.1-2022.8.1 | US/MD | No | NA | cohort study | Early ECMO suvivors VS. nonsurvivors | Early ECMO nonsurvivors | 17 | 29 (22,36) | 82% | Penetrating injury （12%） Blunt （88%） | VV (17) | 29 (22,41) | 0 |
| 111 | Seon Hee Kim | 2023 | 2017.3-2019.2 | Korea/Busan | Yes | NA | case-control study | Survivors vs Nonsurvivors | NA | 21 | 45.0 ± 17.8 | 85.70% | Traffic accident （76.2%） Fall （9.5%） Crush injury （9.5%） Penetrating injury （4.8%） | VV (21) | 28.9 ± 11.0 | 16 |
| 111 | Seon Hee Kim | 2023 | 2017.3-2019.2 | Korea/Busan | Yes | NA | case-control study | Weaning success VS. Weaning failure | Weaning success | 19 | 44.5 ± 16.8 | NA | NA | VV (19) | 28.3 ± 10.9 | 16 |
| 111 | Seon Hee Kim | 2023 | 2017.3-2019.2 | Korea/Busan | Yes | NA | case-control study | Weaning success VS. Weaning failure | Weaning failure | 2 | 49.5 ± 34.7 | NA | NA | VV (2) | 34.5 ± 14.9 | 0 |
| 111 | Seon Hee Kim | 2023 | 2017.3-2019.2 | Korea/Busan | Yes | NA | case-control study | Survivors vs Nonsurvivors | Survivors | 16 | 42.9 ± 17.9 | NA | NA | VV (16) | 26.6 ± 9.1 | 16 |
| 111 | Seon Hee Kim | 2023 | 2017.3-2019.2 | Korea/Busan | Yes | NA | case-control study | Survivors vs Nonsurvivors | Nonsurvivors | 5 | 51.6 ± 17.6 | NA | NA | VV (5) | 36.4 ± 14.1 | 0 |
| new1 | Henry | 2021 | 2013-2016 | USA | Partial Yes | TQIP datebase | Cohort study | ecmo VS no ecmo | no ecmo | 1266 | 56(39,69) | 79% | NA | 0 | 27(20,38) | 633 |
| new1 | Henry | 2021 | 2013-2016 | USA | Partial Yes | TQIP datebase | Cohort study | ecmo VS no ecmo | ecmo | 97 | 35 (22,51) | 81% | NA | NA | 27 (17,34) | 75 |
| new1 | Henry | 2021 | 2013-2016 | USA | Partial Yes | TQIP datebase | Cohort study | Early ≤ 7Days VS Late > 7 Days (n = 26) | Early ≤ 7Days | 71 | 39±17 | 82% | NA | NA | 27 (17,36) | 53 |
| new1 | Henry | 2021 | 2013-2016 | USA | Partial Yes | TQIP datebase | Cohort study | Early ≤ 7Days VS Late > 7 Days (n = 26) | Late > 7 Days | 26 | 34 ±16 | 81% | NA | NA | 26 (19,34) | 21 |
| new1 | Henry | 2021 | 2013-2016 | USA | Partial Yes | TQIP datebase | Cohort study | Anticoagulated VSno Anticoagulated | Anticoagulated | 85 | 36 ±17 | 86% | NA | NA | 27 (17,34) | 66 |
| new1 | Henry | 2021 | 2013-2016 | USA | Partial Yes | TQIP datebase | Cohort study | Anticoagulated VSno Anticoagulated | no Anticoagulated | 12 | 46±17 | 58% | NA | NA | 23 (12,35) | 9 |
| new2 | James E. Huang | 2020 | 2012.1-2018.8 | US | Yes | NA | case series | NA | NA | 12 | 31（27.3,38.0） | 91.70% | Blunt （66.7%） Penetrating injury （25%） Mixed injury （8.3%） | VV (12) | 28.0（18.8,39.0） | 9 |
| new3 | Menaker | 2018 | 2015.1-2016.11 | The R Adams Cowley Shock Trauma Center | Yes | NA | case-control study | Survivors vs Nonsurvivors | NA | 18 | 28.5 ( 24,43) | NA | blunt 67% | VV 18 | 27 ( 21,41) | 14 |
| new3 | Menaker | 2018 | 2015.1-2016.11 | The R Adams Cowley Shock Trauma Center | Yes | NA | case-control study | Survivors vs Nonsurvivors | Survivors | 14 | 30 (27,43) | NA | NA | VV 14 | 33 (22,44) | 14 |
| new3 | Menaker | 2018 | 2015.1-2016.11 |  | Yes | NA | case-control study | Survivors vs Nonsurvivors | Nonsurvivors | 4 | 23（22，31） | NA | NA | VV 4 | 22 (19，24) | 0 |

**Table S4. Newcastle-Ottawa Quality Assessment Form for Cohort Studies**

| **Study_ID** | **Title** | **Country/district** | **1st author** | **Year of publication** | **Selection（0-4）** | | | | **Comparability（0-2）** | **Outcome（0-3）** | | | **Scores (0-9)** |
| --- | --- | --- | --- | --- | --- | --- | --- | --- | --- | --- | --- | --- | --- |
|  |  |  |  |  | **Representativeness of the exposed cohort** | **Selection of the non-exposed cohort** | **Ascertainment of exposure** | **Demonstration that outcome of interest was not present at start of study** | **Comparability of cohorts based on the design or analysis controlled for confounders** | **Assessment of outcome** | **Was follow-up long enough for outcomes to occur** | **Adequacy of follow-up of cohorts** |  |
| 28 | Role of extracorporeal life support for traumatic hemopericardium: A single level I trauma center review | Busan | Eunji Kim | 2024 | ⭐ | ⭐ | ⭐ | ⭐ | ⭐ | ⭐ | ⭐ | ⭐ | 8 |
| 29 | Early Use of Extracorporeal Membrane Oxygenation for Traumatically Injured Patients: A National Trauma Database Analysis | US/ Birmingham, Alabama | Daniel Lammers | 2023 | ⭐ | ⭐ | ⭐ | ⭐ | 0 | ⭐ | ⭐ | ⭐ | 7 |
| 35 | Extracorporeal Membrane Oxygenation May Improve Outcomes After Resuscitative Thoracotomy: A National Trauma Data Bank Analysis | US/NTDB | Natthida Owattanapanich | 2021 | ⭐ | ⭐ | ⭐ | ⭐ | 0 | ⭐ | ⭐ | ⭐ | 7 |
| 41 | Extracorporeal membrane oxygenation in burn patients with refractory acute respiratory distress syndrome leads to 28% of 90-day survival | France/Paris | Sabri Soussi | 2016 | ⭐ | ⭐ | ⭐ | ⭐ | 0 | ⭐ | ⭐ | ⭐ | 7 |
| 50 | 体外膜肺氧合与呼吸机治疗重度肺爆震伤的疗效比较 | China/Zhangzhou | Jianming Chen | 2022 | ⭐ | ⭐ | ⭐ | ⭐ | ⭐ | ⭐ | ⭐ | ⭐ | 8 |
| 53 | 急诊床边体外膜肺治疗重度创伤后ARDS疗效观察 | China/Zhejiang | Guoping Jiang | 2005 | ⭐ | 0 | ⭐ | ⭐ | ⭐ | ⭐ | ⭐ | ⭐ | 7 |
| 70 | Extracorporeal lung support in trauma patients with severe chest injury and acute lung failure: a 10-year institutional experience | Germany/Regensburg | Michael Ried | 2013 | ⭐ | ⭐ | ⭐ | ⭐ | ⭐ | ⭐ | ⭐ | ⭐ | 8 |
| 71 | Venovenous extracorporeal life support improves survival in adult trauma patients with acute hypoxemic respiratory failure: A multicenter retrospective cohort study | US/the Wake Forest School of Medicine and University of Southern California (LAC + USC) Medical Center | Derek M. Guirand | 2014 | ⭐ | ⭐ | ⭐ | ⭐ | ⭐⭐ | ⭐ | ⭐ | ⭐ | 9 |
| 74 | Early initiation of extracorporeal membrane oxygenation improves survival in adult trauma patients with severe adult respiratory distress syndrome | US/ Birmingham, Alabama | Patrick L. Bosarge | 2015 | ⭐ | ⭐ | ⭐ | ⭐ | ⭐⭐ | ⭐ | ⭐ | ⭐ | 9 |
| 83 | A National Perspective on ECMO Utilization in Patients with Burn Injury | US/Washington | Lauren B. Nosanov | 2017 | ⭐ | ⭐ | ⭐ | ⭐ | ⭐⭐ | ⭐ | ⭐ | ⭐ | 9 |
| 85 | The Impact of an Advanced ECMO Program on Traumatically Injured Patients | USA/ University of Miami | April A. Grant | 2018 | ⭐ | ⭐ | ⭐ | ⭐ | ⭐ | ⭐ | ⭐ | ⭐ | 8 |
| 97 | ECMO (extra corporeal membrane oxygenation) in major trauma: A 10 year single centre experience | Australia/The Alfred | Amos | 2021 | 0 | ⭐ | ⭐ | ⭐ | 0 | ⭐ | ⭐ | ⭐ | 6 |
| 102 | Outcome of post‑traumatic acute respiratory distress syndrome in young patients requiring extracorporeal membrane oxygenation (ECMO) | Qatar/level I center | Al‑Thani | 2022 | ⭐ | ⭐ | ⭐ | ⭐ | ⭐⭐ | ⭐ | ⭐ | ⭐ | 9 |
| 105 | Use of venovenous extracorporeal membrane oxygenation in trauma patients with severe adult respiratory distress syndrome: A retrospective study | Korea/Seoul | Lee | 2022 | ⭐ | ⭐ | ⭐ | ⭐ | ⭐⭐ | ⭐ | ⭐ | ⭐ | 9 |
| 110 | Utilization and Outcomes of Extracorporeal Membrane Oxygenation Following Traumatic Brain Injury in the United States | US/Durham | Hatfield | 2023 | ⭐ | ⭐ | ⭐ | ⭐ | ⭐⭐ | ⭐ | ⭐ | ⭐ | 9 |
| 112 | Extracorporeal membrane oxygenation in traumatic brain injury – A retrospective, multicenter cohort study | German, Austrian,  Swiss | Mader | 2023 | ⭐ | ⭐ | ⭐ | ⭐ | ⭐⭐ | ⭐ | ⭐ | ⭐ | 9 |
| 113 | Early veno-venous extracorporeal membrane oxygenation is an effective strategy for traumatically injured patients presenting with refractory respiratory failure | US  Maryland | Powell | 2023 | 0 | ⭐ | ⭐ | ⭐ | 0 | ⭐ | ⭐ | ⭐ | 6 |
| new1 | Extracorporeal support for trauma: A trauma quality improvement project (TQIP) analysis in patients with acute respiratory distress syndrome | US/dallas | henry | 2021 | ⭐ | ⭐ | ⭐ | ⭐ | ⭐⭐ | ⭐ | ⭐ | ⭐ | 9 |

**Table S5. JBI’s tool for assessing case series**

| **Study_ID** | **Title** | **Country/district** | **1st author** | **Year of publication** | **Question** | | | | | | | | | |
| --- | --- | --- | --- | --- | --- | --- | --- | --- | --- | --- | --- | --- | --- | --- |
|  |  |  |  |  | 1.Were there clear criteria for inclusion in the case series? | 2. Was the condition measured in a standard, reliable way for all participants  included in the case series? | 3. Were valid methods used for identification of the condition for all  participants included in the case series? | 4. Did the case series have consecutive inclusion of participants? | 5. Did the case series have complete inclusion of participants? | 6. Was there clear reporting of the demographics of the participants in the study? | 7. Was there clear reporting of clinical information of the participants? | 8. Were the outcomes or follow-up results of cases clearly reported? | 9. Was there clear reporting of the presenting sites’/clinics’ demographic  information? | 10. Was statistical analysis appropriate |
| 14 | Extracorporeal membrane oxygenation for acute respiratory distress syndrome in burn patients: a case series and literature update | Germany/ Bochum | Dadras, M. | 2019 | Yes | Yes | Yes | Yes | Yes | Yes | Yes | Yes | Yes | Yes |
| 16 | Extracorporeal membrane oxygenation utilization in burn patients with severe acute respiratory distress syndrome | US/Chicago | Fouché, T. W. | 2023 | Yes | Yes | Yes | Yes | Yes | Yes | Yes | Yes | Yes | Yes |
| 20 | Benefit of extracorporeal membrane oxygenation in major burns after stun grenade explosion: Experience from a single military medical center | Taiwan/Taipei | Hsu, P. S. | 2016 | Yes | Yes | Yes | Yes | Yes | Yes | Yes | Yes | Yes | Yes |
| 23 | Extracorporeal life support in post-traumatic respiratory distress patients | Taiwan/Taipei | Yao-Kuang Huang | 2009 | Yes | Yes | Yes | Yes | Yes | Yes | Yes | Yes | Yes | Yes |
| 30 | Extracorporeal life support in trauma: Indications and technique | Canada/Vancouver, | Alex Lee | 2023 | Yes | Yes | Yes | Yes | Yes | Yes | Yes | Yes | Yes | Yes |
| 31 | Infections in patients with burn injuries receiving extracorporeal membrane oxygenation | US/Brooke Army Medical Center | Joseph E. Marcus | 2019 | Yes | Yes | Yes | Yes | Yes | Yes | Yes | Yes | Yes | Yes |
| 36 | Venovenous extracorporeal membrane oxygenation experience in a community level I trauma center | US/Ohio | Neubauer | 2022 | Yes | Yes | Yes | Yes | Yes | Yes | Yes | Yes | Yes | Yes |
| 38 | Extracorporeal cardiopulmonary life support with heparin-bonded circuitry in the resuscitation of massively injured trauma patients | US | Michael J. Perchinsky, | 1995 | Yes | Yes | Yes | No | No | Yes | Yes | Yes | No | Yes |
| 45 | EXTRACORPOREAL MEMBRANE OXYGENATION FOR TRAUMA PATIENTS: A SINGLE-CENTER 5-YEAR RETROSPECTIVE REVIEW | US/Nebraska | Tecos, M. | 2023 | Yes | Yes | Yes | Yes | Yes | Yes | Yes | Yes | Yes | Yes |
| 51 | 体外膜肺氧合在严重创伤后重度急性呼吸窘迫综合征中的疗效观察 | China/Zhengzhou | Ying Liu | 2023 | Yes | Yes | Yes | Yes | Yes | Yes | Yes | Yes | Yes | Yes |
| 52 | 体外膜肺氧合救治严重创伤患者8例临床分析及文献复习 | China/Zhejiang | Danting Fei | 2023 | Yes | Yes | Yes | Yes | Yes | Yes | Yes | Yes | Yes | Yes |
| 54 | 回顾性分析和系统综述体外膜肺氧合在烧伤合并急性呼吸窘迫综合征救治中的临床效果 | China/Chongqing | Haisheng Li | 2021 | Yes | Yes | Yes | Unclear | Yes | Yes | Yes | Yes | Yes | Yes |
| 55 | 体外膜肺氧合在严重肺挫伤中的应用 | China/Guangdong | Gang Xie | 2005 | Yes | Yes | Yes | Unclear | Yes | Yes | Yes | Yes | Yes | Yes |
| 61 | Extracorporeal life support for respiratory failure after multiple trauma | US/Michigan | Anderson | 1994 | Yes | Yes | Yes | Yes | Yes | Yes | Yes | Yes | Yes | Yes |
| 62 | Extracorporeal life support for patients with significant orthopaedic trauma | US/Michigan | Senunas, L. E. | 1997 | Yes | Yes | Yes | Yes | Yes | Yes | Yes | Yes | Yes | Yes |
| 67 | Transportable extracorporeal lung support for rescue of severe respiratory failure in combat casualties | Germany/Regensburg | Thomas Bein | 2012 | Yes | Yes | Yes | Yes | Yes | Yes | Yes | Yes | Yes | Yes |
| 68 | Extracorporeal life support in patients with multiple injuries and severe respiratory failure:A single-center experience? | Israel/Jerusalem, | Philippe Biderman | 2013 | Yes | Yes | Yes | Yes | Yes | Yes | Yes | Yes | Yes | Yes |
| 72 | Venoarterial extracorporeal life support in post-traumatic shock and cardiac arrest:lessons learned | Taiwan/Taoyuan | Yuan-His Tseng | 2014 | Yes | Yes | Yes | Yes | Yes | Yes | Yes | Yes | Yes | Yes |
| 76 | Early Percutaneous Heparin-Free Veno-Venous Extra Corporeal Life Support (ECLS)is a Safe and Effective Means of Salvaging Hypoxemic Patients with Complicated Chest Trauma | Taiwan/Cathay General Hospital | Thay-Hsiung Chen | 2016 | Yes | Yes | Yes | Yes | Yes | Yes | Yes | Yes | Yes | Yes |
| 77 | Extracorporeal Membrane Oxygenation Support in Trauma Versus Nontrauma Patients with Noninfectious Acute Respiratory Failure | Korea/Anyang | Hyoung Soo Kim, | 2016 | Yes | Yes | Yes | Yes | Yes | Yes | Yes | Yes | Yes | Yes |
| 78 | Outcome measures of extracorporeal life support (ECLS) in trauma patients versus patients without trauma:a 7-year single-center retrospective cohort study | Germany/Bochum | Christopher Ull | 2016 | Yes | Yes | Yes | Yes | Yes | Yes | Yes | Yes | Yes | Yes |
| 81 | Is extracorporeal cardiopulmonary resuscitation practical in severe chest trauma? A systematic review in single center of developing country | Busan, Korea | Huh | 2017 | Yes | Yes | Yes | Yes | Yes | Yes | Yes | Yes | Yes | Yes |
| 86 | Revisiting extracorporeal membrane oxygenation for ARDS in burns: A case series and review of the literature | US Army Institute of Surgical Research | Craig R. Ainsworth | 2018 | Yes | Yes | Yes | Yes | Yes | Yes | Yes | Yes | Yes | Yes |
| 89 | Extracorporeal membrane oxygenation in trauma: A single institution experience and review of the literature | USA/Los Angeles | Aaron Strumwasser | 2018 | Yes | Yes | Yes | Yes | Yes | Yes | Yes | Yes | Yes | Yes |
| 90 | Extracorporeal membrane oxygenation in patients with severe respiratory failure following burns and smoke inhalation injury | UK/ Manchester | Lajos Szentgyorgyi | 2018 | Yes | Yes | Yes | Yes | Yes | Yes | Yes | Yes | Yes | Yes |
| 95 | Access to extracorporeal life support as a quality metric: Lessons from trauma | USA california/ level I or II trauma centers. | Akhmerov | 2020 | Yes | Yes | Yes | Yes | Yes | Yes | Yes | Yes | Yes | Yes |
| 99 | Single Center Experience With Veno-Venous Extracorporeal Membrane Oxygenation in Patients With Traumatic Brain Injury | US/San Francisco | Parker | 2021 | Yes | Yes | Yes | Unclear | Yes | Yes | Yes | Yes | Yes | Yes |
| 103 | Application and outcomes of extracorporeal life support in emergency general surgery and trauma | USA/Hartford | Brewer | 2022 | Yes | Yes | Yes | Yes | Yes | Yes | Yes | Yes | Yes | Yes |
| 104 | Extracorporeal membrane oxygenation support in the setting of penetrating traumatic injuries | USA/Dallas | Eisenga | 2022 | Yes | Yes | Yes | Yes | Yes | Yes | No | Yes | No | Yes |
| 106 | Traumatic respiratory failure and veno-venous extracorporeal membrane oxygenation support | US/Houston | Salas | 2022 | Yes | Yes | Yes | Yes | Yes | Yes | Yes | Yes | Yes | Yes |
| 107 | Use of Extracorporeal Membrane Oxygenation in Blunt Traumatic Injury Patients with Acute Respiratory Distress Syndrome | US/Kentucky | Trivedi, | 2022 | Yes | Yes | Yes | Unclear | Yes | Yes | Yes | Yes | Yes | Yes |
| 108 | Analysis of extracorporeal membrane oxygenation in trauma patients with acute respiratory distress syndrome: A case series | Germany/Hannover | Weidemann | 2022 | Yes | Yes | Yes | Yes | Yes | Yes | No | Yes | Yes | Yes |
| new2 | Predictive survival factors of the traumatically injured on venovenous extracorporeal membrane oxygenation: A Bayesian model | US Hawaii /level 1 trauma center | Huang JE | 2020 | Yes | Yes | Yes | Yes | Yes | Yes | Yes | Yes | Yes | Yes |

**Table S6. CASP-case-control-study-checklist**

| **Study_ID** | **Title** | **Country/district** | **1st author** | **Year of publication** | **Section A: Are the results of the trial valid?** | | | | | | **Section B: What are the results?** | | | **Section C: Will the results help locally?** | |
| --- | --- | --- | --- | --- | --- | --- | --- | --- | --- | --- | --- | --- | --- | --- | --- |
|  |  |  |  |  | **1. Did the study address a clearly focused issue?** | **2. Did the authors use an appropriate method to answer their question?** | **3. Were the cases recruited in an acceptable way?** | **4. Were the controls selected in an acceptable way?** | **5. Was the exposure accurately measured to minimise bias?** | **6. (a) Aside from the experimental intervention, were the groups treated equally? (b) Have the authors taken account of the potential confounding factors in the design and/or in their analysis?** | **7. How large was the treatment effect?** | **8. How precise was the estimate of the treatment effect?** | **9. Do you believe the results?** | **10. Can the results be applied to the local population?** | **11. Do the results of this study fit with other available evidence?** |
| 2 | Predictors of Mortality in Trauma Patients with ARDS on Extracorporeal Membrane Oxygenation | US/CA | Miseker Abate | 2022 | Yes | Yes | Yes | Yes | Yes | (a)Yes（b）Yes | The study found that severe head and thorax injuries were significant predictors of mortality, with odds ratios of 2.66 and 3.52, respectively. | The estimates were provided with 95% confidence intervals, indicating the precision of the results. | Yes | Yes | Yes |
| 7 | Venovenous extracorporeal membrane oxygenation in patients with traumatic brain injuries and severe respiratory failure: A single-center retrospective analysis | US/ Birmingham, Alabama | Austin | 2023 | Yes | Yes | Yes | Yes | Yes | (a)Yes（b）Yes | The study found no significant difference in survival rates between TBI  and non-TBI patients treated with VV ECMO. Specific survival rates and  the odds ratios were provided, indicating the relative similarity in  outcomes. | Confidence intervals and p-values were reported, showing the precision  of the estimates. For instance, the survival rate difference had a  p-value of 0.45, indicating no statistically significant difference. | Yes | Yes | Yes |
| 11 | Extracorporeal life support use in adult Burn patients | US/Washington | Christopher R. Burke | 2016 | Yes | Yes | Yes | Yes | Yes | (a)Yes（b）Yes | The study found a 43% survival rate to hospital discharge for adult burn patients treated with ECLS. It identified acidosis and pre-ECLS use of inotropes/vasopressors as significant risk factors for increased mortality. | Confidence intervals and p-values were provided for key outcomes. For  example, acidosis had an odds ratio (OR) of 7.3 (95% CI: 1.39–38.11),  indicating a significant impact on mortality. | Yes | Yes | Yes |
| 37 | Predictors of Mortality in Extracorporeal Membrane Oxygenation Support Patients Following Major Trauma | US/ Massachusetts | Joep J.J. Ouwerkerk | 2023 | Yes | Yes | Yes | Yes | Yes | (a)Yes（b）Yes | Among 542 patients, 205 died (37.8%). Independent predictors of mortality included female gender, ECMO within 4 hours after presentation, decreased Glasgow Coma Scale (GCS), increased age, units of blood transfused in the first 4 hours, and abbreviated injury score (AIS) for external injuries. An external AIS of ≥3 had the strongest predictive value for mortality. | The results include specific numerical values and statistical analyses,  such as odds ratios, confidence intervals, and p-values for key outcomes  and predictors. | Yes | Yes | Yes |
| 64 | Traumatic lung injury treated by extracorporealmembrane oxygenation (ECMO) | UK/ Sheffield | J.A. Cordell-Smith | 2006 | Yes | Yes | Yes | Yes | Yes | (a)Unclear（b）No | The study found: Of the 28 patients treated with ECMO, 20 (approximately 71.4%) survived and were discharged from the tertiary unit. | The results include specific numerical values and proportions for survivors and non-survivors, such as the Murray scores and PaO2/FiO2 ratios. | Yes | Yes | Yes |
| 69 | Extracorporeal life support in patients with severe trauma: An advanced treatment strategy for refractory clinical settings | Italy/, Careggi Teaching Hospital, Largo Brambilla, Florence | Massimo Bonacchi | 2013 | Yes | Yes | Yes | Yes | Yes | (a)Yes（b）Yes | Among the 18 patients treated with ECLS, 14 were successfully supported, showing significant improvements in hemodynamic and respiratory parameters. The survival rate was 71.4% among the successfully treated patients. | The results include specific numerical values and statistical analyses, such as means, standard deviations, and confidence intervals for key outcomes and predictors. | Yes | Yes | Yes |
| 73 | Venovenous extracorporeal life support for posttraumatic respiratory distress syndrome in adults:the risk of major hemorrhages | Taiwan/Taoyuan | Meng-Yu Wu | 2014 | Yes | Yes | Yes | Yes | Yes | (a)Yes（b）Yes | Out of 20 patients treated with VV-ECLS, 14 survived. The median  PaO2/FiO2 ratio significantly improved from 56 to 106 mmHg soon after  VV-ECLS initiation. However, seven major hemorrhages occurred, with  three being lethal. | The results include specific numerical values and statistical analyses,  such as means, standard deviations, and confidence intervals for key  outcomes and predictors. | Yes | Yes | Yes |
| 75 | Use of extracorporeal membrane oxygenation in severe traumatic lung injury with respiratory failure | Taiwan/ Chang Gung | Shih-Chi Wu, | 2015 | Yes | Yes | Yes | Yes | Yes | (a)Yes（b）Yes | The study found: Out of 19 patients treated with ECMO, 13 survived, resulting in a survival rate of 68.4%. Significant differences were noted in age and ICU stay between survivors and non-survivors. | The results include specific numerical values and statistical analyses, such as means, standard deviations, and confidence intervals for key outcomes and predictors. | Yes | Yes | Yes |
| 79 | Extracorporeal membrane oxygenation after traumatic injury | US/Maryland School | Sarwat B. Ahmad | 2017 | Yes | Yes | Yes | Yes | Yes | (a)Yes（b）Yes | The study found: Of the 39 patients treated with venovenous ECMO, 17 survived, resulting in a survival rate of 44%. Significant differences were noted in BMI, arterial pH, PaCO2, and ISS between survivors and non-survivors. | The results include specific numerical values and statistical analyses, such as means, standard deviations, and confidence intervals for key outcomes and predictors. | Yes | Yes | Yes |
| 80 | Extracorporeal life support is safe in trauma patients | US/ | Burke CR 2017 | 2017 | Yes | Yes | Yes | Yes | Yes | (a)Yes（b）Yes | The study found: Overall survival to hospital discharge  was 64%. Non-survivors had a higher median ISS (29 vs. 24, p = 0.018)  and a shorter median total hospital length of stay (8 days vs. 32 days, p  < 0.001). | The results include specific numerical values and statistical analyses,  such as means, interquartile ranges (IQR), and confidence intervals for  key outcomes and predictors. | Yes | Yes | Yes |
| 82 | Extracorporeal membrane oxygenation support in post-traumatic cardiopulmonary failure A 10-year single institutional experience |  | Lin | 2017 | Yes | Yes | Yes | Yes | Yes | (a)Yes（b）Yes | The study identified significant prognostic factors for in-hospital  mortality, including ISS >30 (OR, 9.48; 95% CI: 1.04–18.47; P=0.042)  and the requirement of RRT (OR, 8.64; 95% CI: 1.73–26.09; P=0.020). | The confidence intervals and p-values provided for key prognostic factors indicate a high degree of precision in the estimates. | Yes | Yes | Yes |
| 92 | Assessment of safety and bleeding risk in the use of extracorporeal membrane oxygenation for multitrauma patients: A multicenter review | AUS/New South Wales | Natalie Kruit | 2019 | Yes | Yes | Yes | Yes | Yes | (a)Yes（b）Yes | The study found: Out of 52 patients treated with ECMO,  the overall hospital mortality was 15%. The incidence of bleeding  complications was 50%, with most not requiring intervention. The 30-day  and 6-month survival rates were 88% and 85%, respectively. | The results include specific numerical values and statistical analyses, such as means, standard deviations, confidence intervals, and p-values for key outcomes and predictors. | Yes | Yes | Yes |
| 93 | Survival rate and outcome of extracorporeal life support (ecLS) for treatment of acute cardiorespiratory failure in trauma patients | Austria/Vienna | nikolaus W. Lang | 2019 | Yes | Yes | Yes | Yes | Yes | (a)Yes（b）Yes | The study found: Of the 18 patients treated with ECLS, 6 survived (33.3%) and had a satisfying neurological outcome with a mean Glasgow Outcome Scale (GOS) of 5. Survivors were significantly younger than non-survivors and had a lower ISS. | The results include specific numerical values and statistical analyses, such as means, interquartile ranges (IQR), and confidence intervals for key outcomes and predictors. | Yes | Yes | Yes |
| 94 | Clinical outcomes of extracorporeal membrane oxygenation in acute traumatic lung injury: a retrospective study | Korea/ Gwanpyeong-ro | Lee HK | 2020 | Yes | Yes | Yes | Yes | Yes | (a)Yes（b）Yes | The study found: Thirty-four out of 42 patients (81%) survived and were discharged after a median hospital stay of 23 days. Multivariate analysis identified pre-ECMO lactate level and type of ECMO (VV ECMO) as significant predictors of survival. | The results include specific numerical values and statistical analyses, such as odds ratios, confidence intervals, and p-values for key outcomes and predictors. | Yes | Yes | Yes |
| 111 | Outcomes in trauma patients undergoing veno-venous extracorporeal membrane oxygenation for acute respiratory distress syndrome | Korea/Pusan | Kim SH | 2023 | Yes | Yes | Yes | Yes | Yes | (a)Yes（b）Yes | The study found: Of the 21 trauma patients treated with VV ECMO, 19 patients (90.5%) were successfully weaned off ECMO, and 16 patients (76.2%) survived to discharge. Univariate analysis showed significant differences in survival between groups with different TRISS scores. | The results include specific numerical values and statistical analyses, such as means, standard deviations, confidence intervals, and p-values for key outcomes and predictors. | Yes | Yes | Yes |
| new3 | Veno-Venous Extracorporeal Membrane Oxygenation (VV ECMO) for Acute Respiratory Failure Following Injury: Outcomes in a High-Volume Adult Trauma Center with a Dedicated Unit for VV ECMO | USA/Baltimore | Jay Menaker | 2018 | Yes | Yes | Yes | Yes | Yes | (a)Yes（b）Yes | Eighteen patients required VV ECMO during the study period. The median age was 28.5 years, and the survival to discharge was 78%. Survivors had a significantly higher ISS, longer ICU and hospital length of stay, and more ventilator days compared to non-survivors. The median time from injury to cannulation was longer in survivors. | The results include specific numerical values and statistical analyses, such as means, interquartile ranges (IQR), and p-values for key outcomes and predictors. | Yes | Yes | Yes |

**Table S7. Evaluation of the certainty of evidence.**

| **Certainty assessment** | | | | | | | **Certainty** | **Importance** |  |
| --- | --- | --- | --- | --- | --- | --- | --- | --- | --- |
| **№ of studies** | **Study design** | **Risk of bias** | **Inconsistency** | **Indirectness** | **Imprecision** | **Other considerations** |  |  |  |
| **Survival of traumatic ARDS patients with and without ECMO** | | | | | | | | |  |
| 6 | non-randomised studies | not serious | serious^a^ | not serious | serious^b^ | none | ⨁◯◯◯ Very low | CRITICAL |  |
| **Survival (traumatic cardiac arrest)** | | | | | | | | |  |
| 14 | non-randomised studies | not serious | not serious | not serious | serious^c^ | none | ⨁◯◯◯ Very low | CRITICAL |  |
| **Survival (TBI vs. non-TBI)** | | | | | | | | |  |
| 12 | non-randomised studies | not serious | not serious | not serious | serious^c^ | none | ⨁◯◯◯ Very low | CRITICAL |  |
| **Survival (burn injury)** | | | | | | | | |  |
| 9 | non-randomised studies | not serious | serious^a^ | not serious | serious^c^ | none | ⨁◯◯◯ Very low | CRITICAL |  |
| **ISS (ECMO vs. non-ECMO)** | | | | | | | | | |
| 7 | | non-randomised studies | not serious | very serious^g^ | serious^e^ | serious^c^ | none | ⨁◯◯◯ Very low | CRITICAL |
| **ISS (survivors vs. non-survivors)** | | | | | | | | | |
| 14 | | non-randomised studies | not serious | not serious | serious^e^ | serious^c^ | none | ⨁◯◯◯ Very low | CRITICAL |

| **Certainty assessment** | | | | | | | | | | | | | | **Certainty** | | **Importance** | |
| --- | --- | --- | --- | --- | --- | --- | --- | --- | --- | --- | --- | --- | --- | --- | --- | --- | --- |
| **№ of studies** | | **Study design** | | **Risk of bias** | | **Inconsistency** | | **Indirectness** | | **Imprecision** | | **Other considerations** | |  |  |  |  |
| **Survival (time from injury to ECMO < 5 days *vs.* > 5 days)** | | | | | | | | | | | | | | | | |  |
| 8 | | non-randomised studies | | not serious | | not serious | | serious^d^ | | serious^c^ | | none | | ⨁◯◯◯ Very low | | CRITICAL |  |
| **Survival (time from admission to ECMO < 5 days *vs.* > 5 days)** | | | | | | | | | | | | | | | | |  |
| 9 | | non-randomised studies | | not serious | | serious^a^ | | serious^d^ | | serious^c^ | | none | | ⨁◯◯◯ Very low | | CRITICAL |  |
| **Survival (with vs. without interdisciplinary approach during ECMO treatment)** | | | | | | | | | | | | | | | | |  |
| 18 | | non-randomised studies | | serious | | not serious | | not serious | | serious^c^ | | none | | ⨁◯◯◯ Very low | | CRITICAL |  |

#### ECMO: Extracorporeal Membrane Oxygenation; TBI: traumatic brain injuries; CA: cardiac arrest; ISS: injury severity score.

#### Explanations

a. Substantial heterogeneity, of unequivocal importance.

b. The optimal information size criterion is met, but the 95% CI overlaps no effect and fails to exclude important benefit.

c. The sample size is insufficient.

d. There are time differences in outcomes.

e. The interventions used in the studies differed.

f. The optimal information size criterion is not met. The 95% CI overlaps no effect and fails to exclude important benefit.

g. Considerable heterogeneity, of unequivocal importance.

**Figure S2-30. Domain 1 subgroup meta-analyses.**

**Domain 1. Indications**


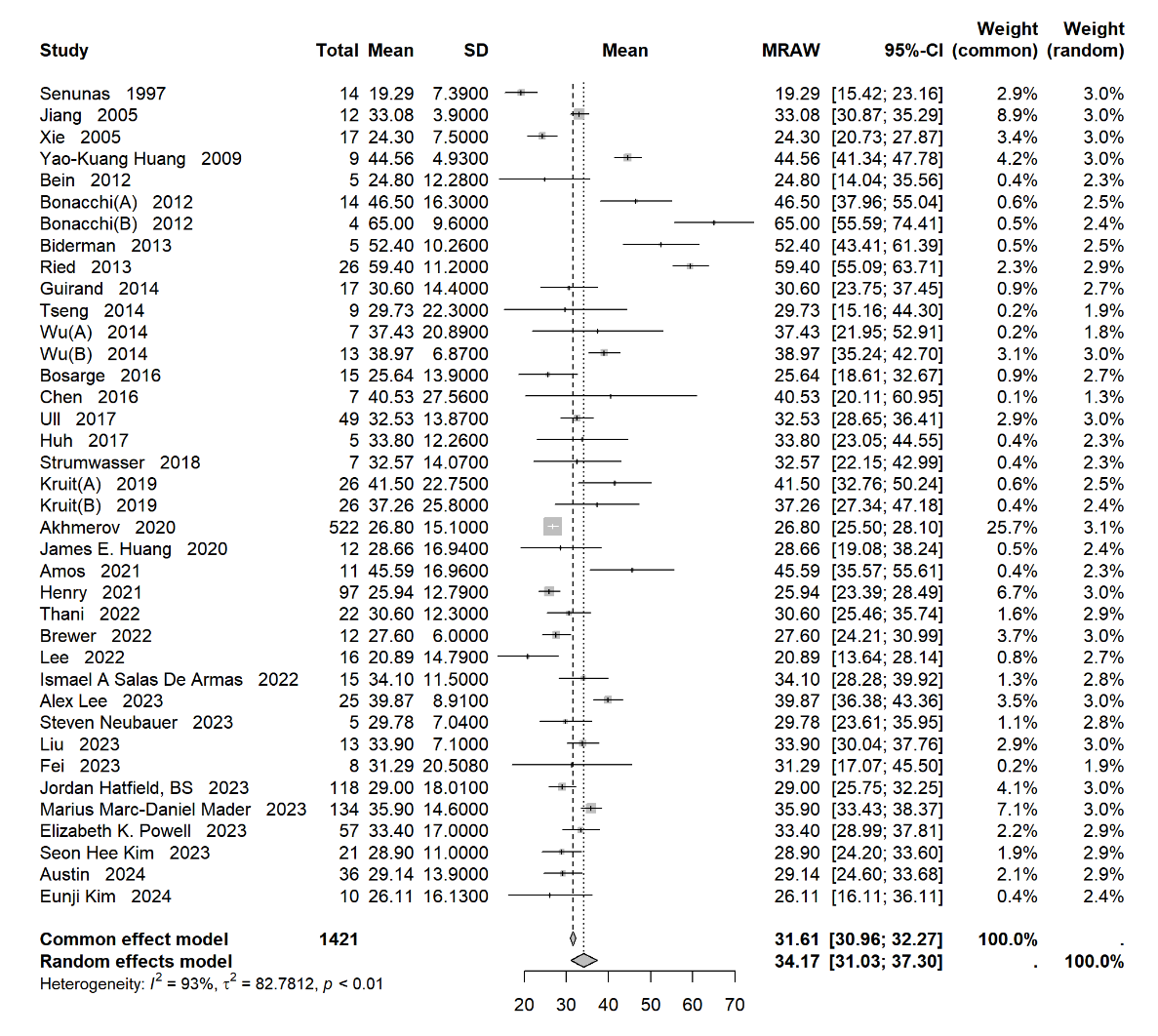


**Figure S2.** Forest plot of ISS of the ECMO-supported adult trauma patients.

CI: confidence interval; SD: Standard Deviation; ISS: Injury Severity Score; MRAW: Mean of Raw Values; ECMO: Extracorporeal Membrane Oxygenation.


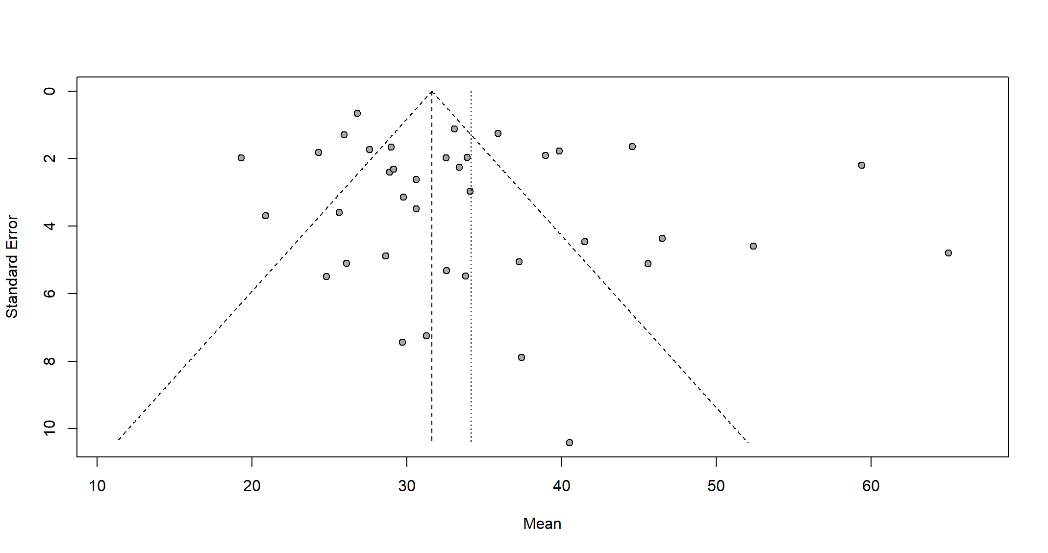


**Figure S3.** Funnel plot of ISS of the ECMO-supported adult trauma patients.


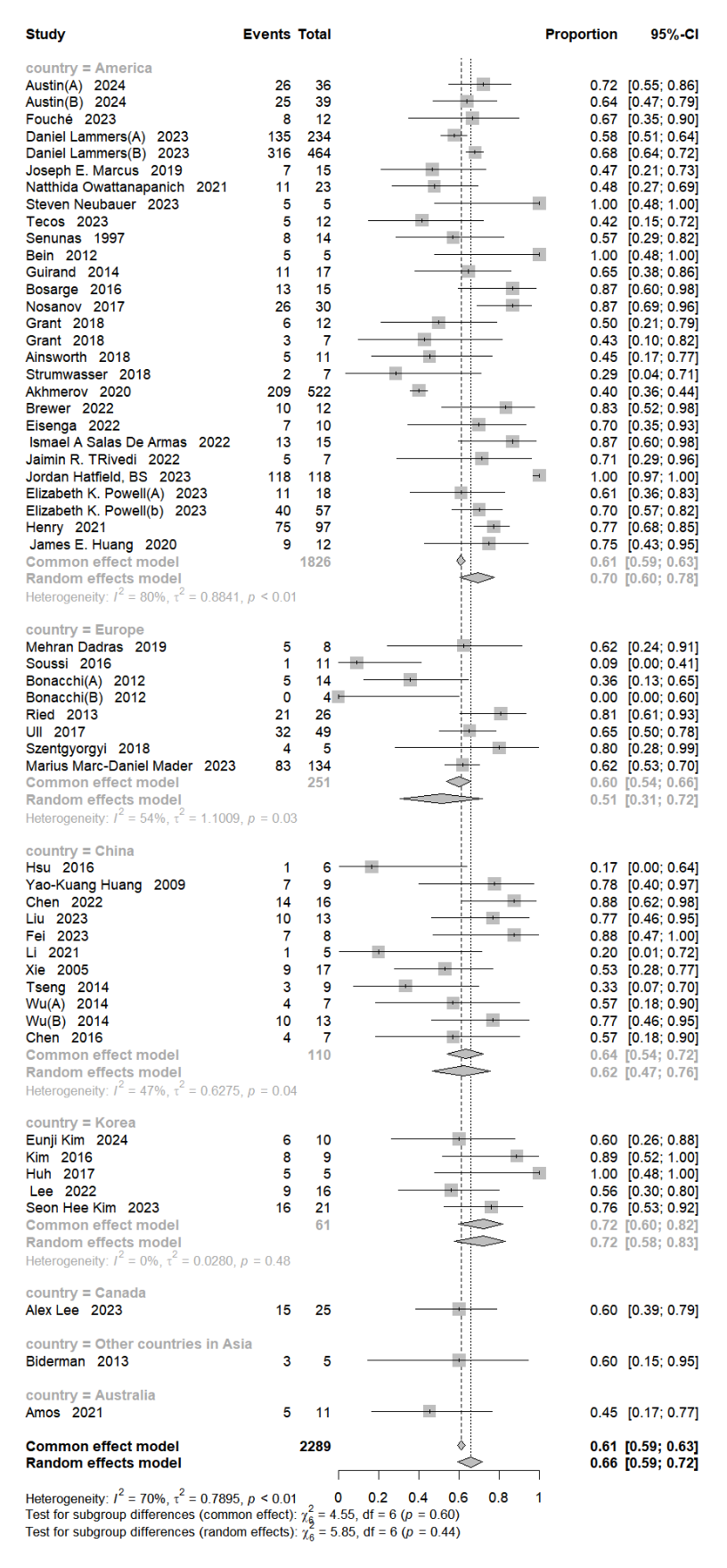


**Figure S4.** Forest plot of overall survival rate of the ECMO-supported adult trauma patients across geographical regions.

CI: confidence interval; ECMO: Extracorporeal Membrane Oxygenation.


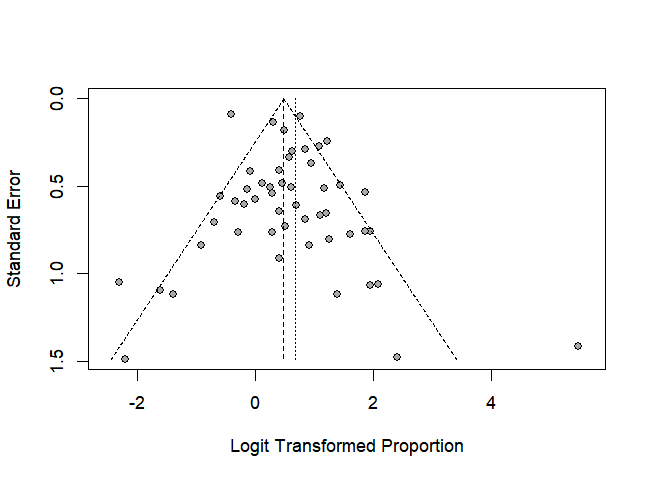


**Figure S5.** Funnel plot of overall survival rate of the ECMO-supported adult trauma patients across geographical regions.


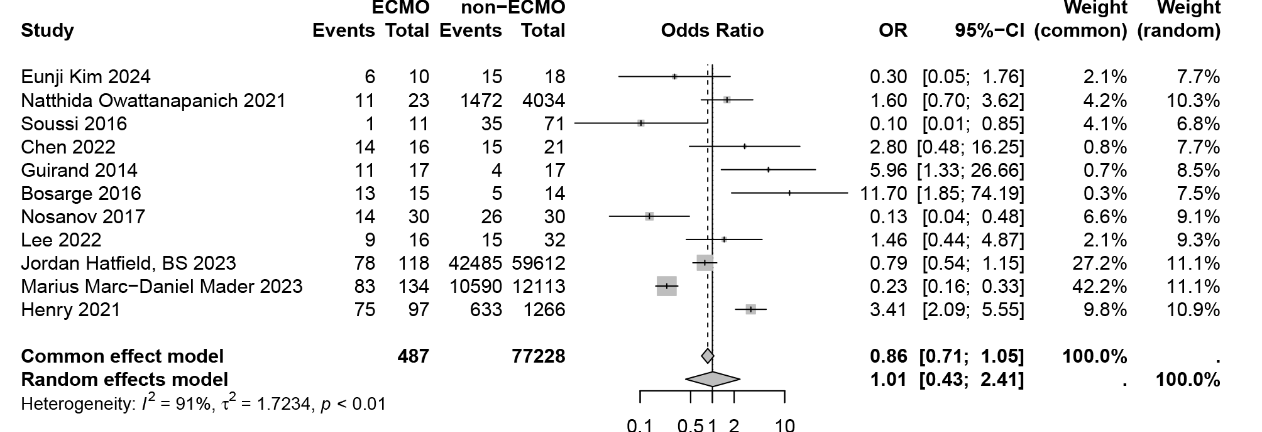


**Figure S6.** Forest plot of Odds Ratio of survival in trauma patients with *vs*. without ECMO.

CI: confidence interval; ECMO: Extracorporeal Membrane Oxygenation.


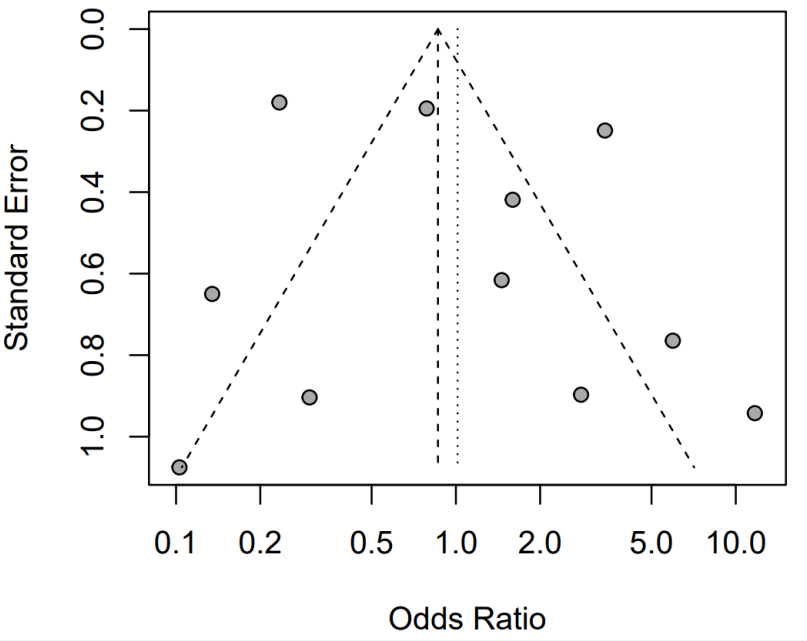


**Figure S7.** Funnel plot of Odds Ratio of survival in trauma patients with *vs*. without ECMO.


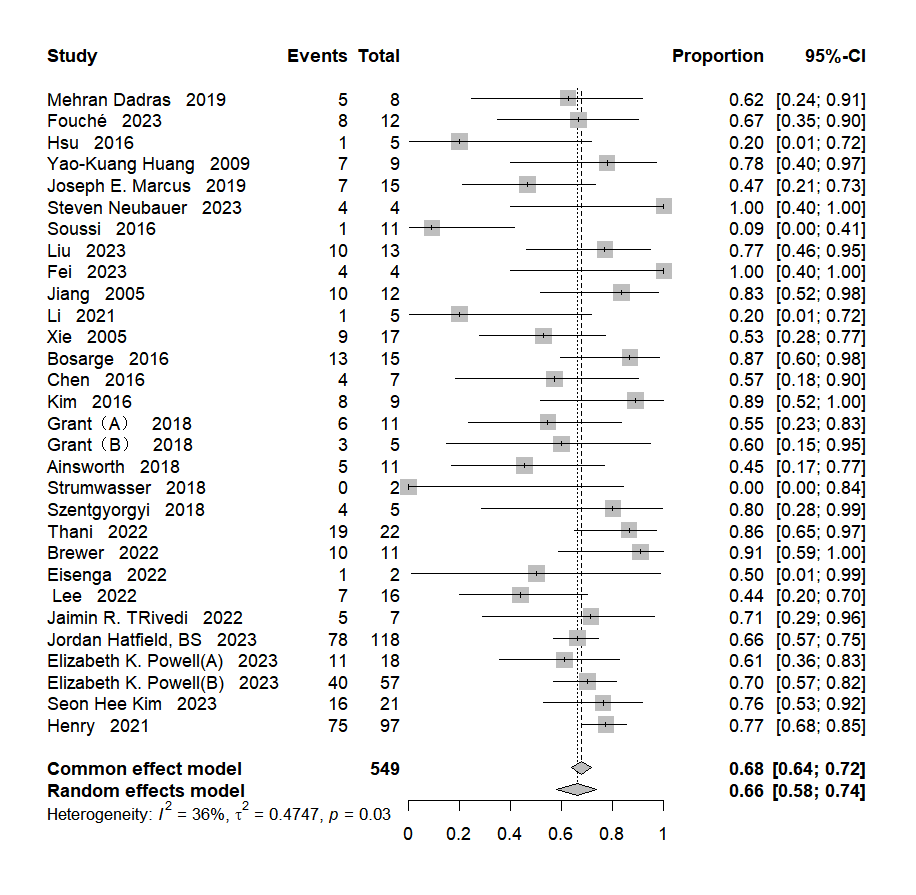


**Figure S8.** Forest plot of overall survival rate of the traumatic ARDS patients exposed to ECMO.

CI: confidence interval; ARDS: acute respiratory distress syndrome; ECMO: Extracorporeal Membrane Oxygenation.


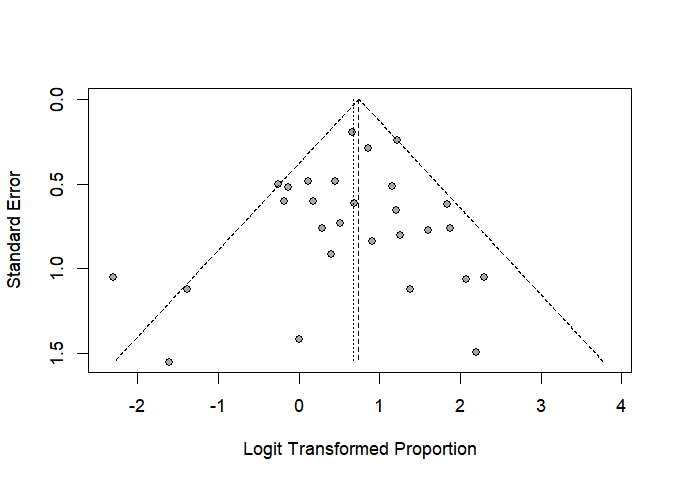


**Figure S9.** Funnel plot of overall survival rate of the traumatic ARDS patients exposed to ECMO.


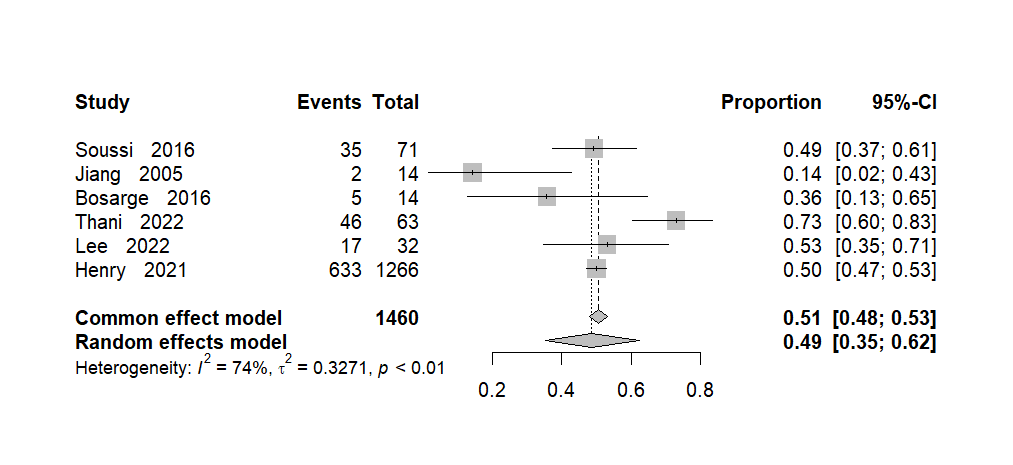


**Figure S10.** Forest plot of survival rate of traumatic ARDS patients unexposed to ECMO.

CI: confidence interval; ECMO: Extracorporeal Membrane Oxygenation; ARDS: Acute Respiratory Distress Syndrome.


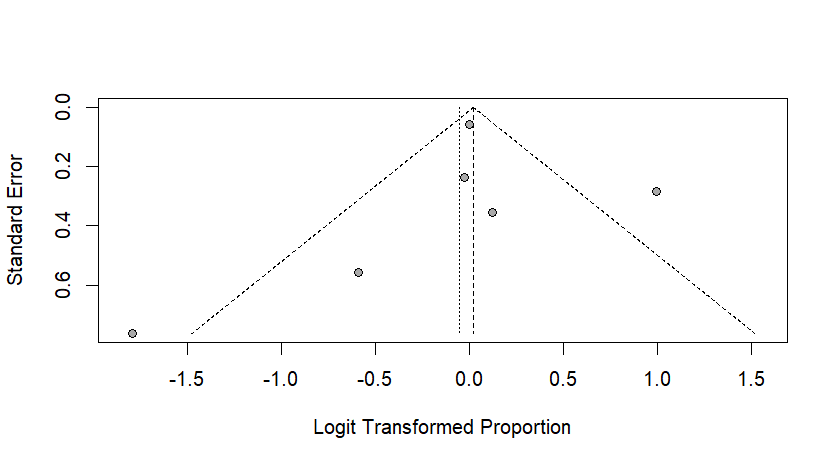


**Figure S11.** Funnel plot of survival rate of traumatic ARDS patients unexposed to ECMO.


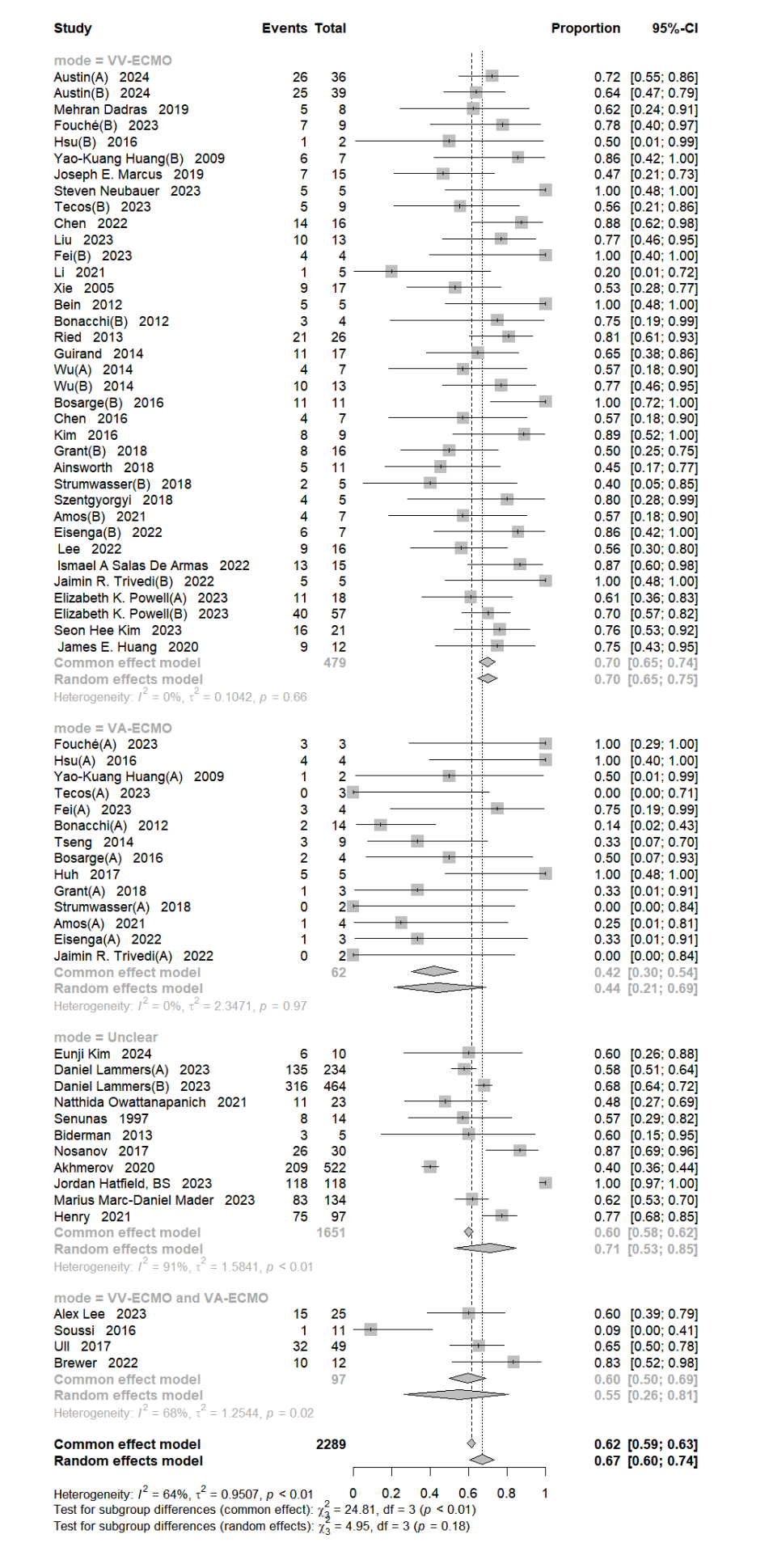


**Figure S12.** Forest plot of overall survival rate of patients on VA- and VAV-ECMO with traumatic cardiac arrest and cardiogenic shock.

CI: confidence interval; ECMO: Extracorporeal Membrane Oxygenation.


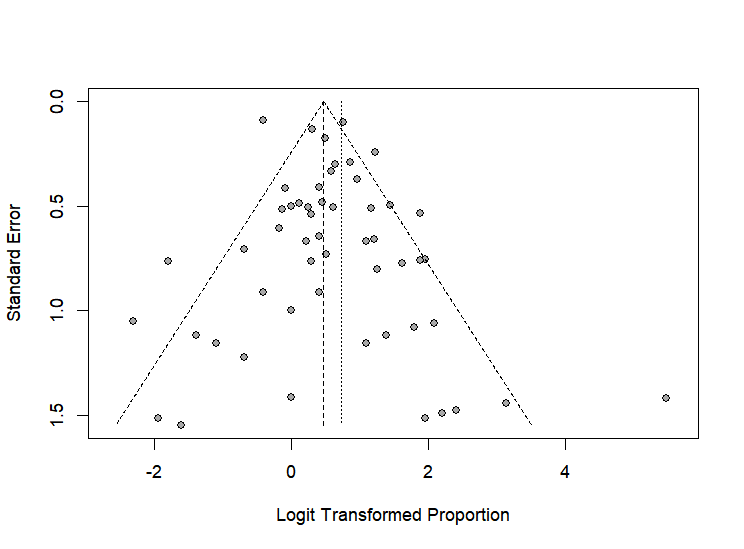


**Figure S13.** Funnel plot of overall survival rate of patients on VA- and VAV-ECMO with traumatic cardiac arrest and cardiogenic shock.


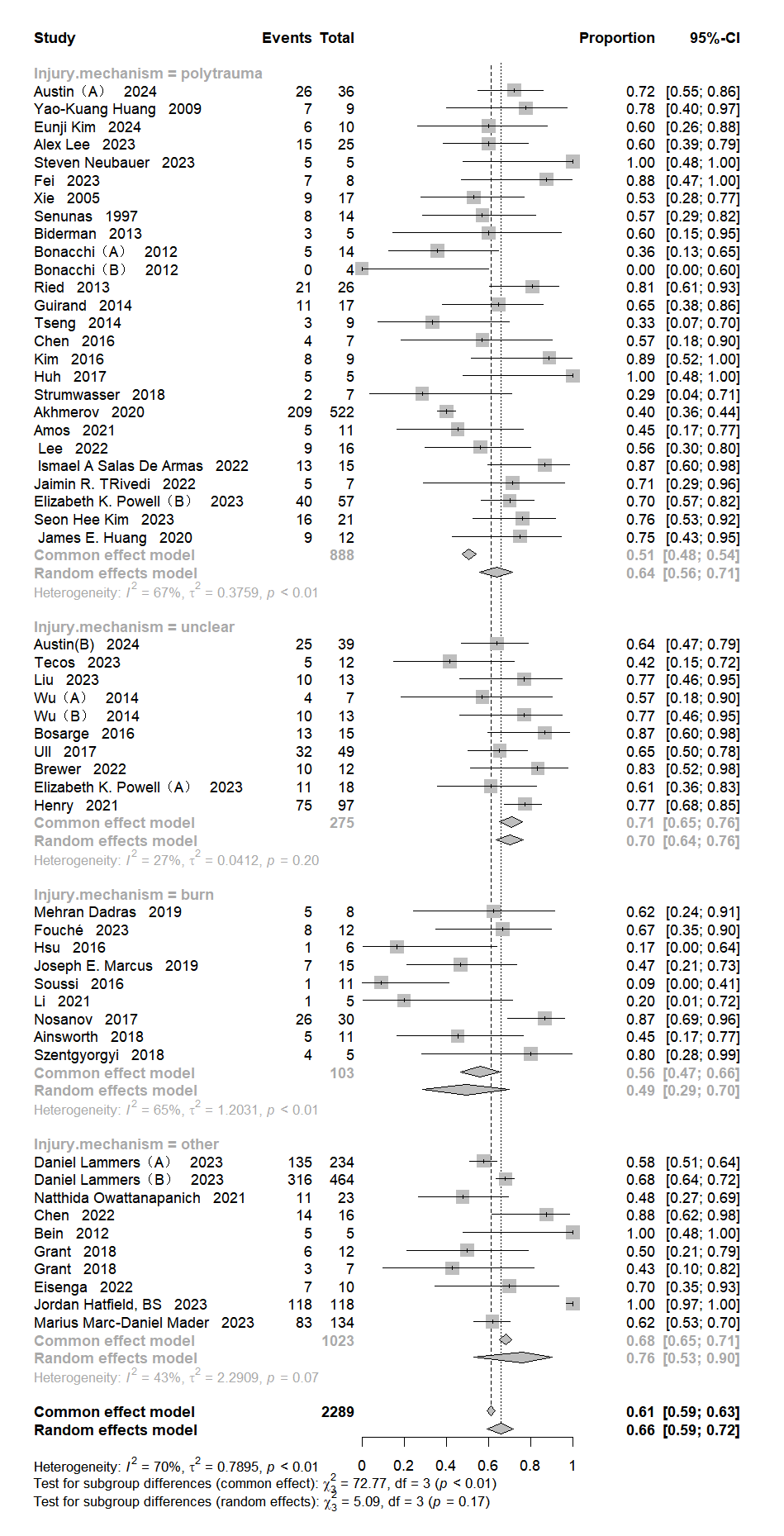


**Figure S14.** Subgroup analysis of overall survival rate of the polytraumatized patients on ECMO.

CI: confidence interval.


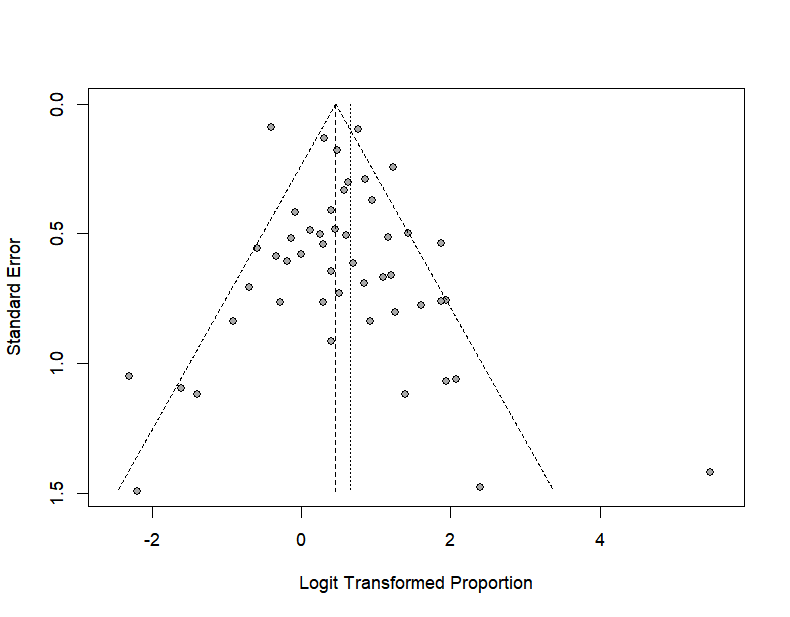


**Figure S15**. Funnel plot of overall survival rate of the polytraumatized patients on ECMO





**Figure S16.** Forest plot of Odds Ratio of survival of traumatic ARDS patients with and without ECMO.

CI: confidence interval; ARDS: acute respiratory distress syndrome.





**Figure S17.** Funnel plot of survival rate of Odds Ratio of survival of traumatic ARDS patients with and without ECMO.


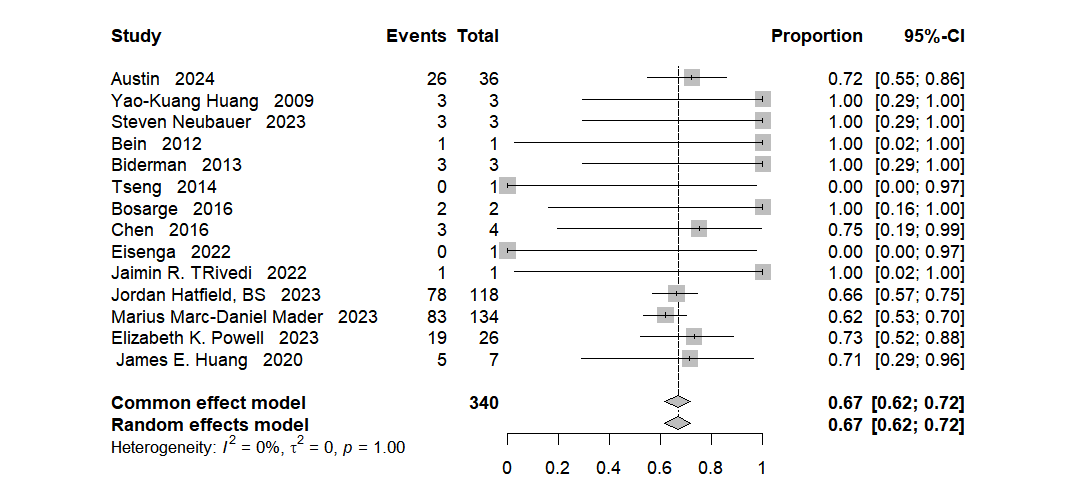


**Figure S18.** Forest plot of overall survival rate of TBI patients with ECMO.

CI: confidence interval; TBI: traumatic brain injury.


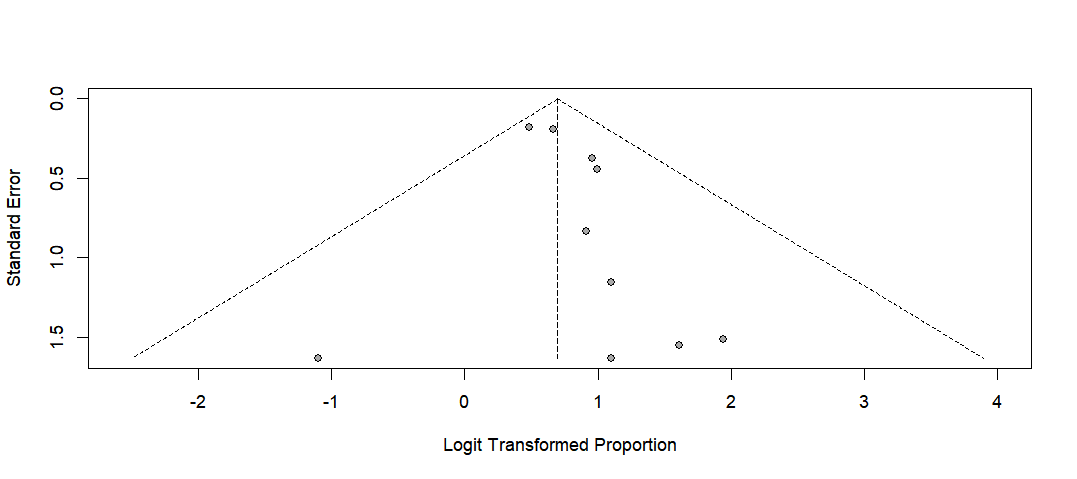


**Figure S19.** Funnel plot of overall survival rate of TBI patients with ECMO.


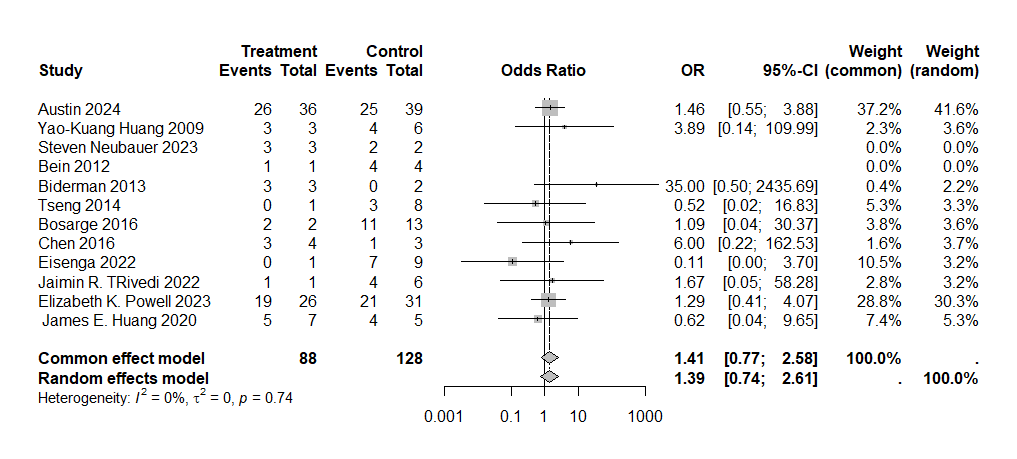


**Figure S20.** Forest plot of Odds Ratio of survival in TBI patients with *vs.* without ECMO.

CI: confidence interval; ECMO, Extracorporeal Membrane Oxygenation; TBI, Traumatic Brain Injury.



**Figure S21.** Forest plot of the total body surface area of patients with burns and inhalation injuries.

CI: confidence interval; SD: Standard Deviation; MRAW: Mean of Raw Values.





**Figure S22.** Funnel plot of the total body surface area of patients with burns and inhalation injuries.


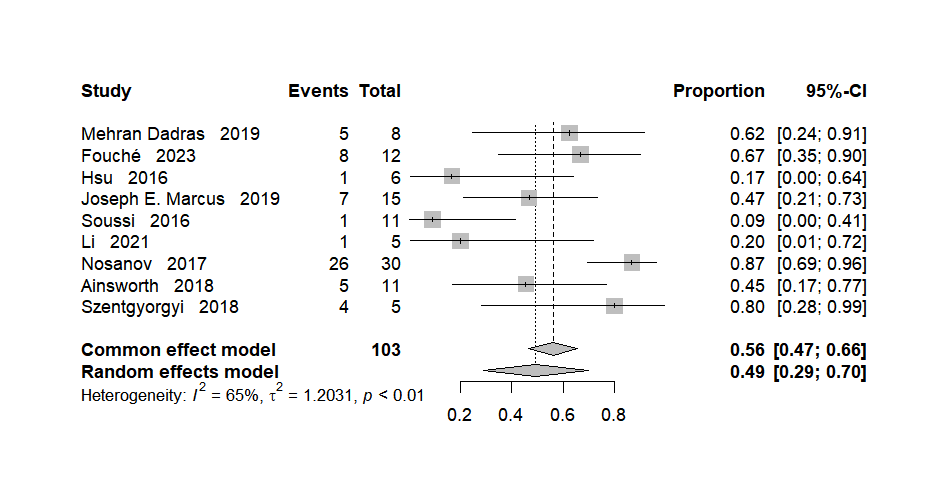


**Figure S23.** Forest plot of the survival of patients with burns and inhalation injuries.

CI: confidence interval.


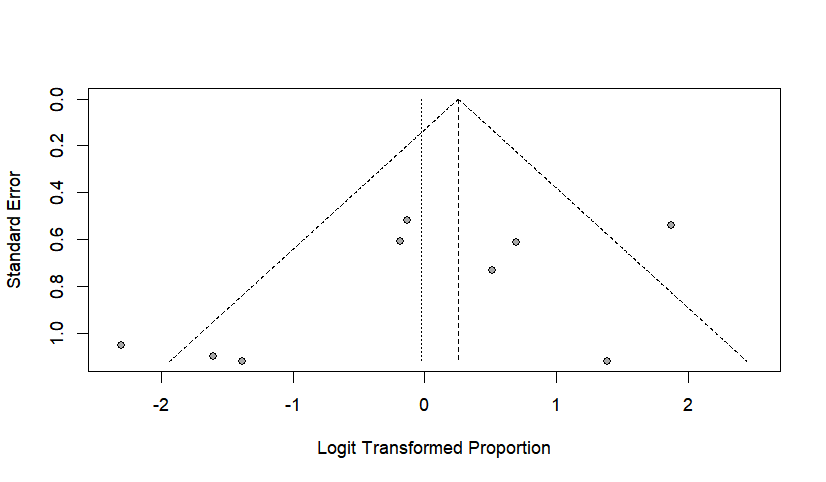


**Figure S24.** Funnel plot of the survival of patients with burns and inhalation injuries.





**Figure 25.** Forest plot of length of stay of the ECMO-supported adult trauma patients in the intensive care unit.

CI: confidence interval; SD: Standard Deviation; MRAW: Mean of Raw Values; ECMO: Extracorporeal Membrane Oxygenation.





**Figure 26.** Funnel plot of length of stay of the ECMO-supported adult trauma patients in the intensive care unit.





**Figure 27.** Forest plot of length of stay of the ECMO-supported adult trauma patients in hospital.

CI: confidence interval; SD: Standard Deviation; MRAW: Mean of Raw Values; ECMO: Extracorporeal Membrane Oxygenation.





**Figure 28.** Funnel plot of length of stay of the ECMO-supported adult trauma patients in hospital.





**Figure S29.** Forest plot of duration on ECMO of the ECMO-supported adult trauma patients

CI: confidence interval; SD: Standard Deviation; MRAW: Mean of Raw Values; ECMO: Extracorporeal Membrane Oxygenation.





**Figure S30.** Funnel plot of duration on ECMO of the ECMO-supported adult trauma patients

**Figure S31-42. Domain 2 subgroup meta-analyses.**

**Domain 2. Patient Screening**


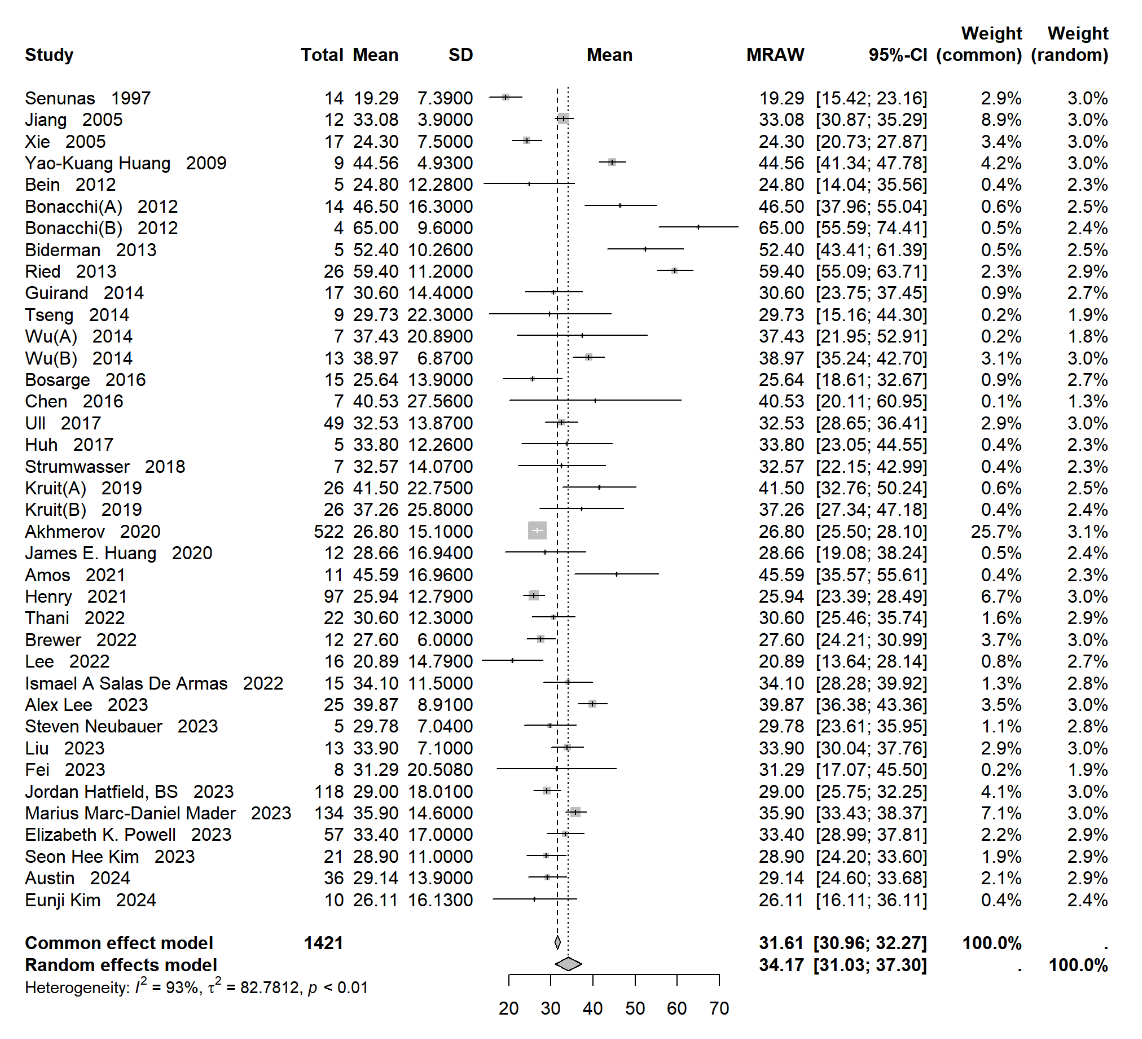


**Figure S31.** Forest plot of ISS of trauma patients with ECMO.

CI: confidence interval; SD: Standard Deviation; MRAW: Mean of Raw Values; ISS: Injury Severity Score; ECMO: Extracorporeal Membrane Oxygenation.


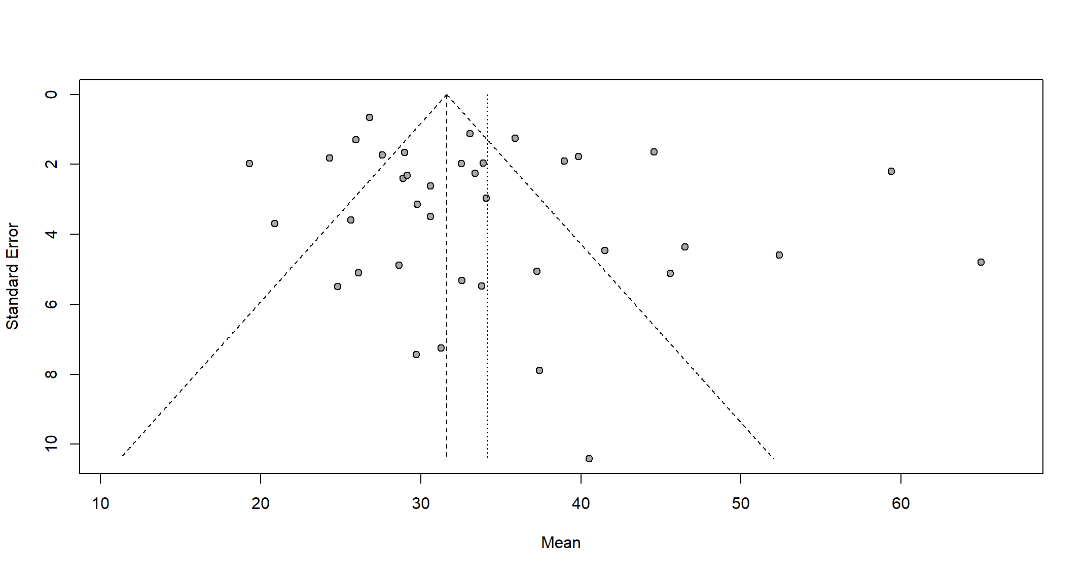


**Figure S32.** Funnel plot of ISS of trauma patients with ECMO.


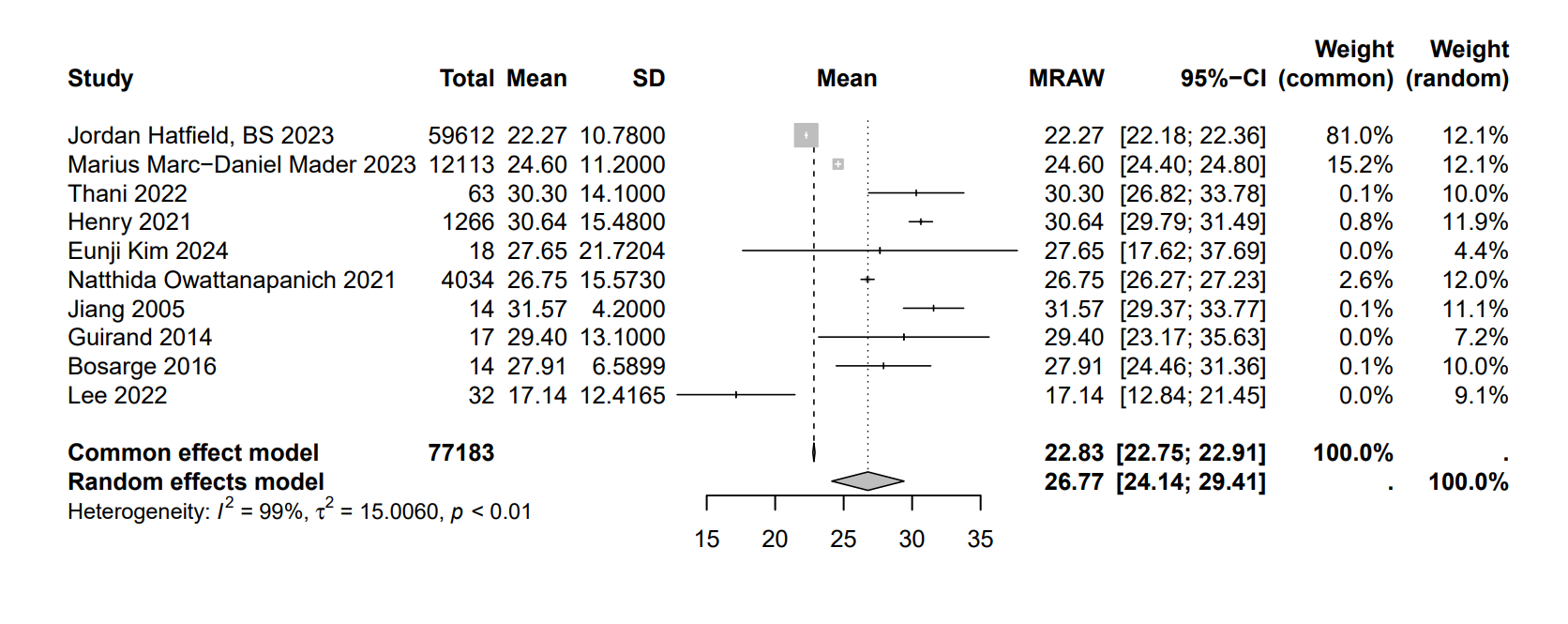


**Figure S33.** Forest plot of ISS of trauma patients without ECMO.

CI: confidence interval; SD: Standard Deviation; MRAW: Mean of Raw Values; ISS: Injury Severity Score; ECMO: Extracorporeal Membrane Oxygenation.


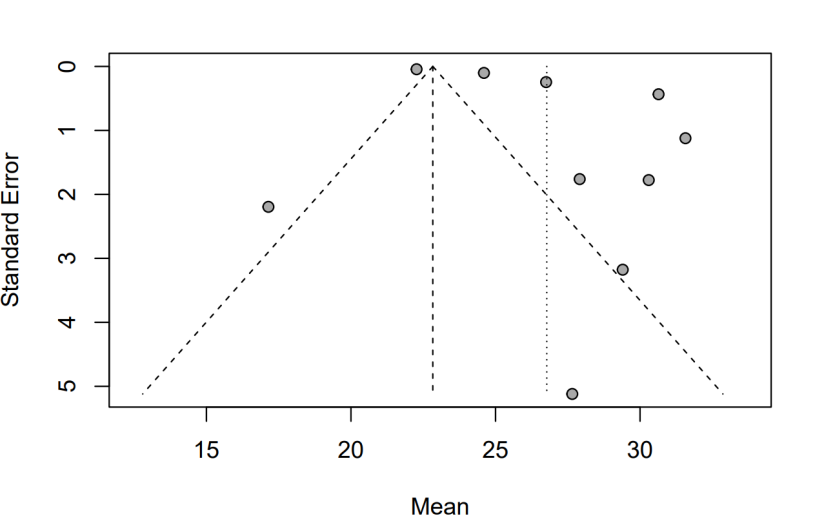


**Figure S34.** Funnel plot of ISS of trauma patients without ECMO.





**Figure S35.** Forest plot of SMD of ISS in trauma patients with *vs.* without ECMO.

CI: confidence interval; SD: Standard Deviation; SMD: Standardized Mean Difference; ISS: Injury Severity Score; ECMO: Extracorporeal Membrane Oxygenation.





**Figure S36.** Funnel plot of SMD of ISS in trauma patients with *vs*. without ECMO.


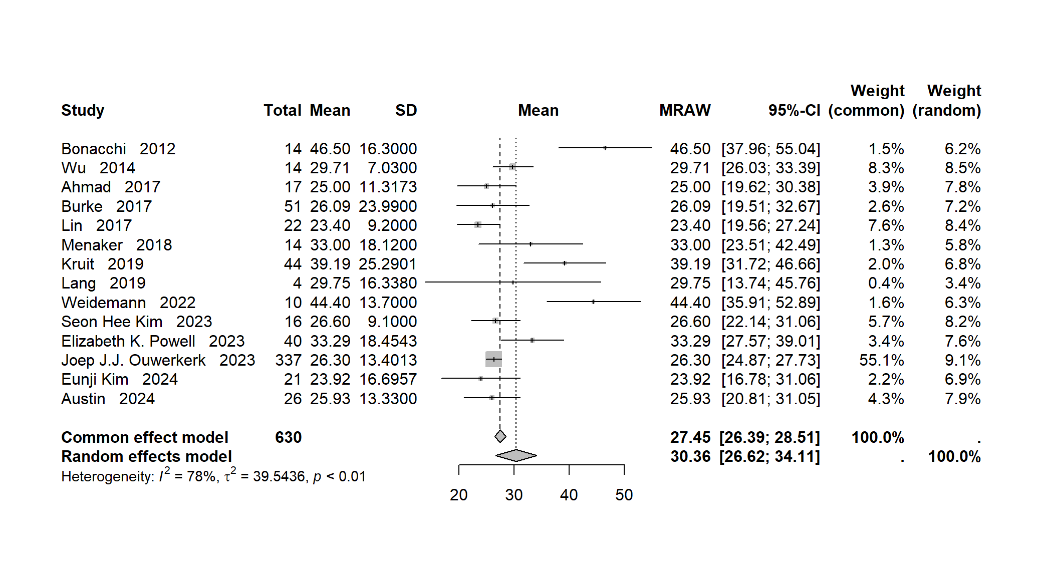


**Figure S37**. Forest plot of ISS of the survivors of adult trauma patients on ECMO.

CI: confidence interval; SD: Standard Deviation; MRAW: Mean of Raw Values; ISS: Injury Severity Score; ECMO: Extracorporeal Membrane Oxygenation.


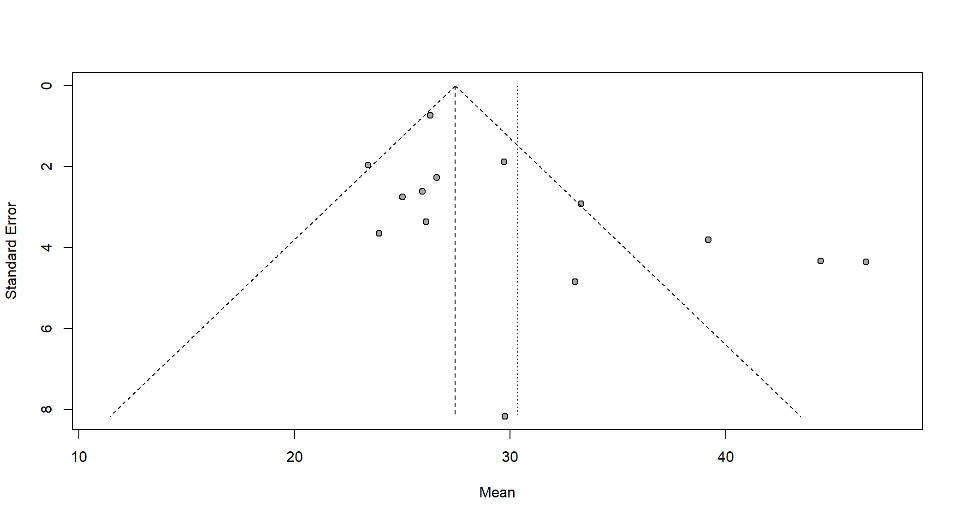
**Figure S38.** Funnel plot of ISS of the survivors of adult trauma patients on ECMO.


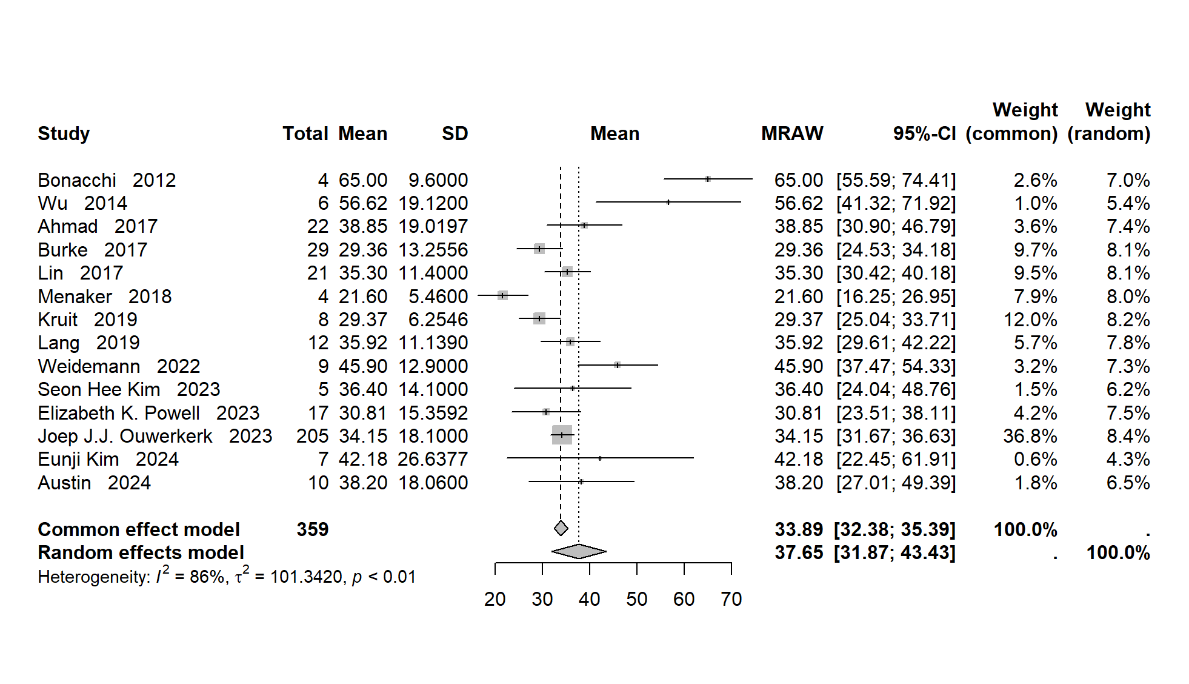
 **Figure S39.** Forest plot of ISS of the non-survivors of adult trauma patients on ECMO.

CI: confidence interval; SD: Standard Deviation; MRAW: Mean of Raw Values; ISS: Injury Severity Score; ECMO: Extracorporeal Membrane Oxygenation.


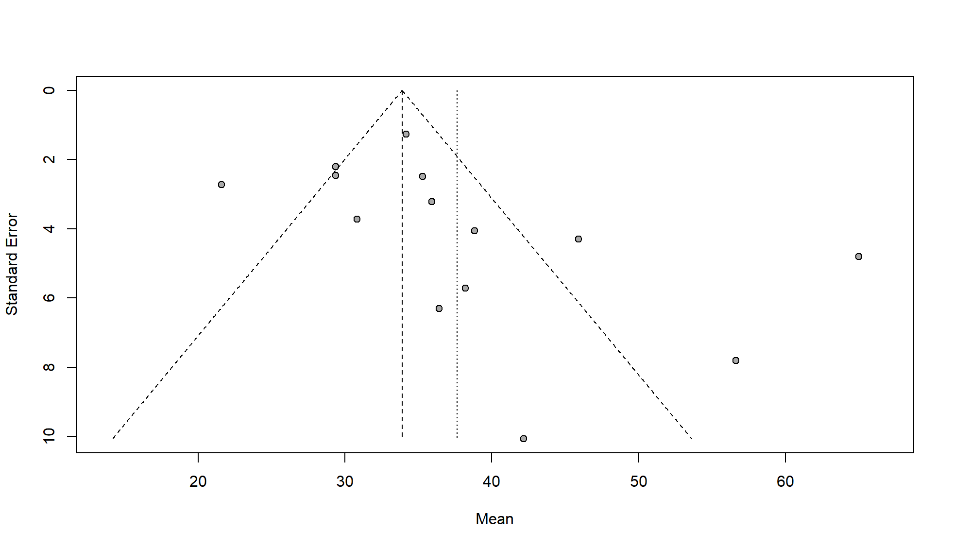


**Figure S40.** Funnel plot of ISS of the non-survivors of adult trauma patients on ECMO.


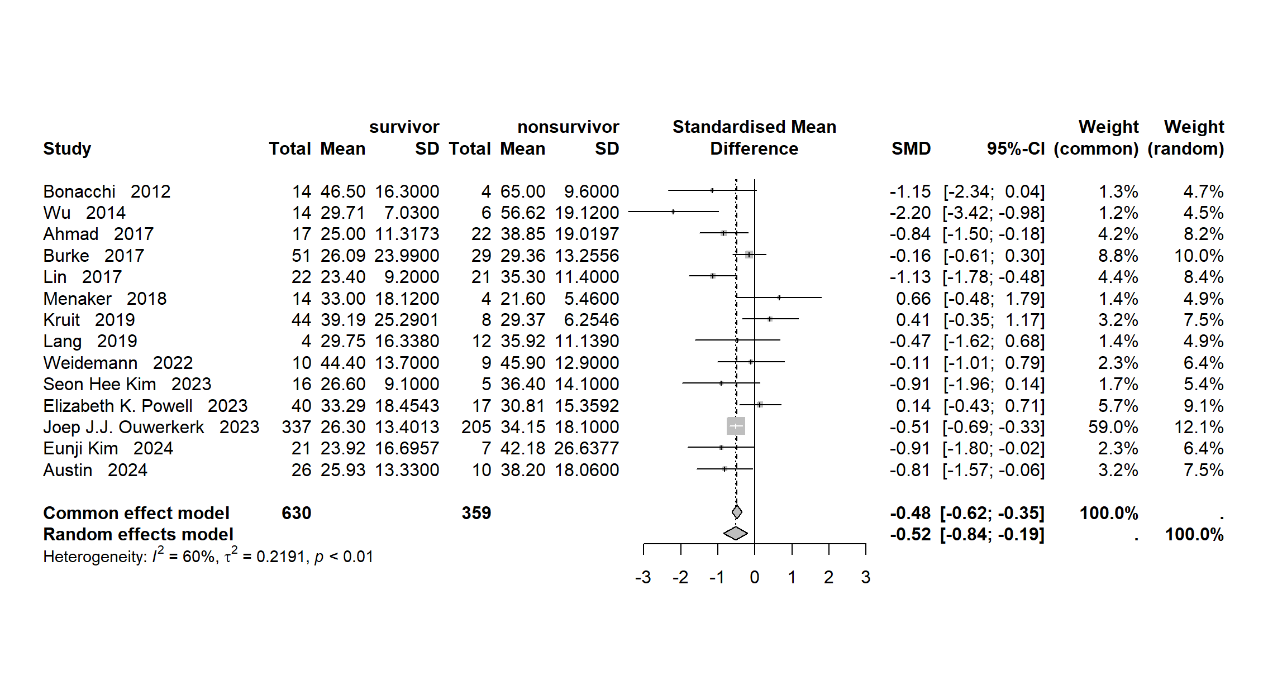
**Figure S41.** Forest plot of ISS of the survivors *vs*. non-survivors of adult trauma patients on ECMO.

CI: confidence interval; SD: Standard Deviation; SMD: Standardized Mean Difference; ISS: Injury Severity Score; ECMO: Extracorporeal Membrane Oxygenation.


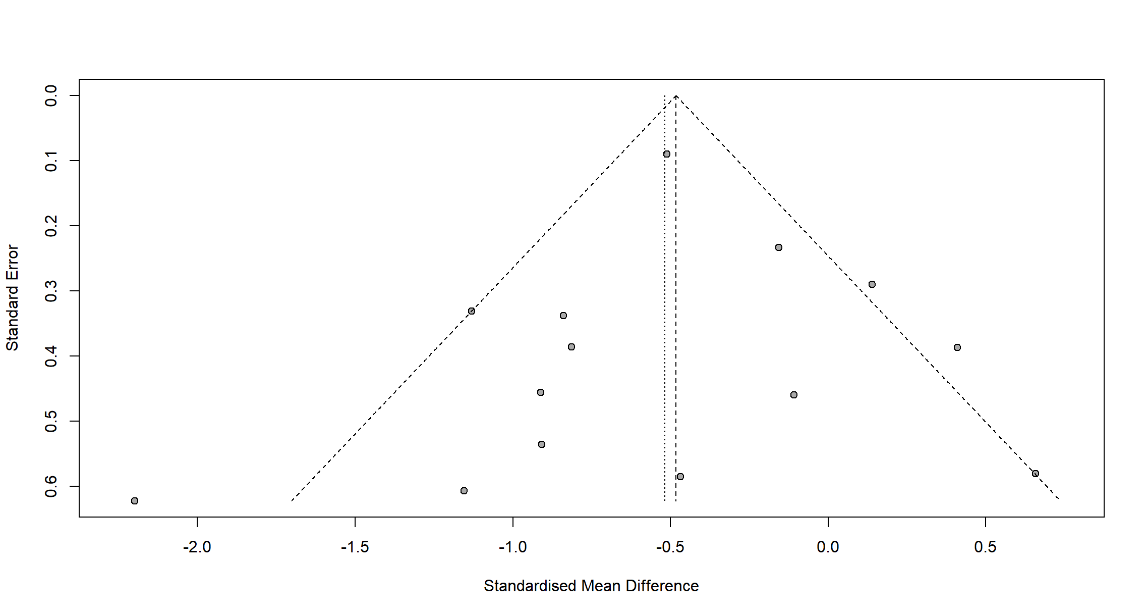


**Figure S42.** Funnel plot of ISS of the survivors *vs*. non-survivors of adult trauma patients on ECMO.

**Figure S43-50. Domain 3 subgroup meta-analyses.**

**Domain 3. Timing of initiation**


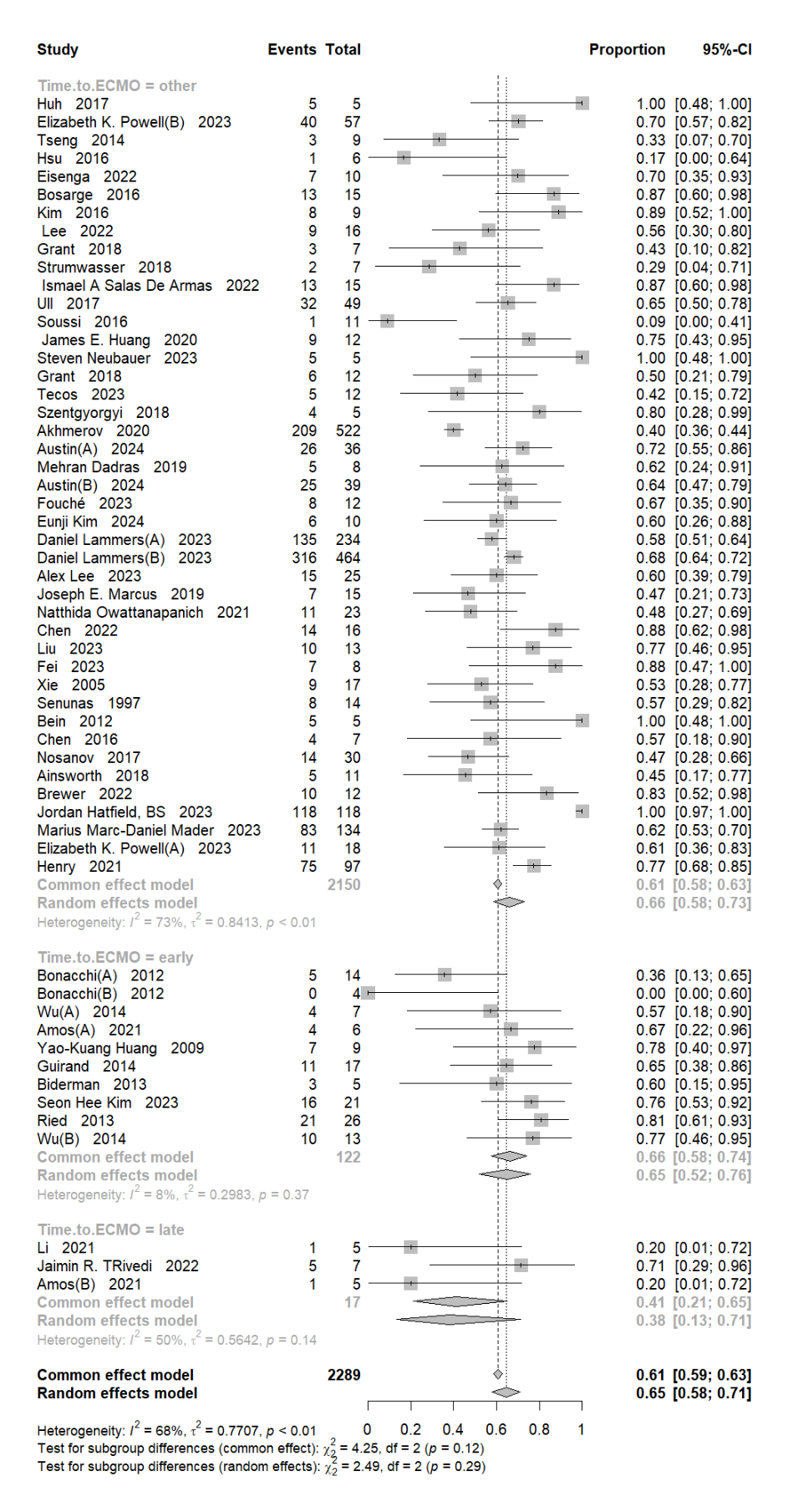


**Figure S43.** Forest plot of overall survival rate of adult trauma patients placed on ECMO within 5 days from injury to ECMO initiation.

CI: confidence interval; ECMO: Extracorporeal Membrane Oxygenation.


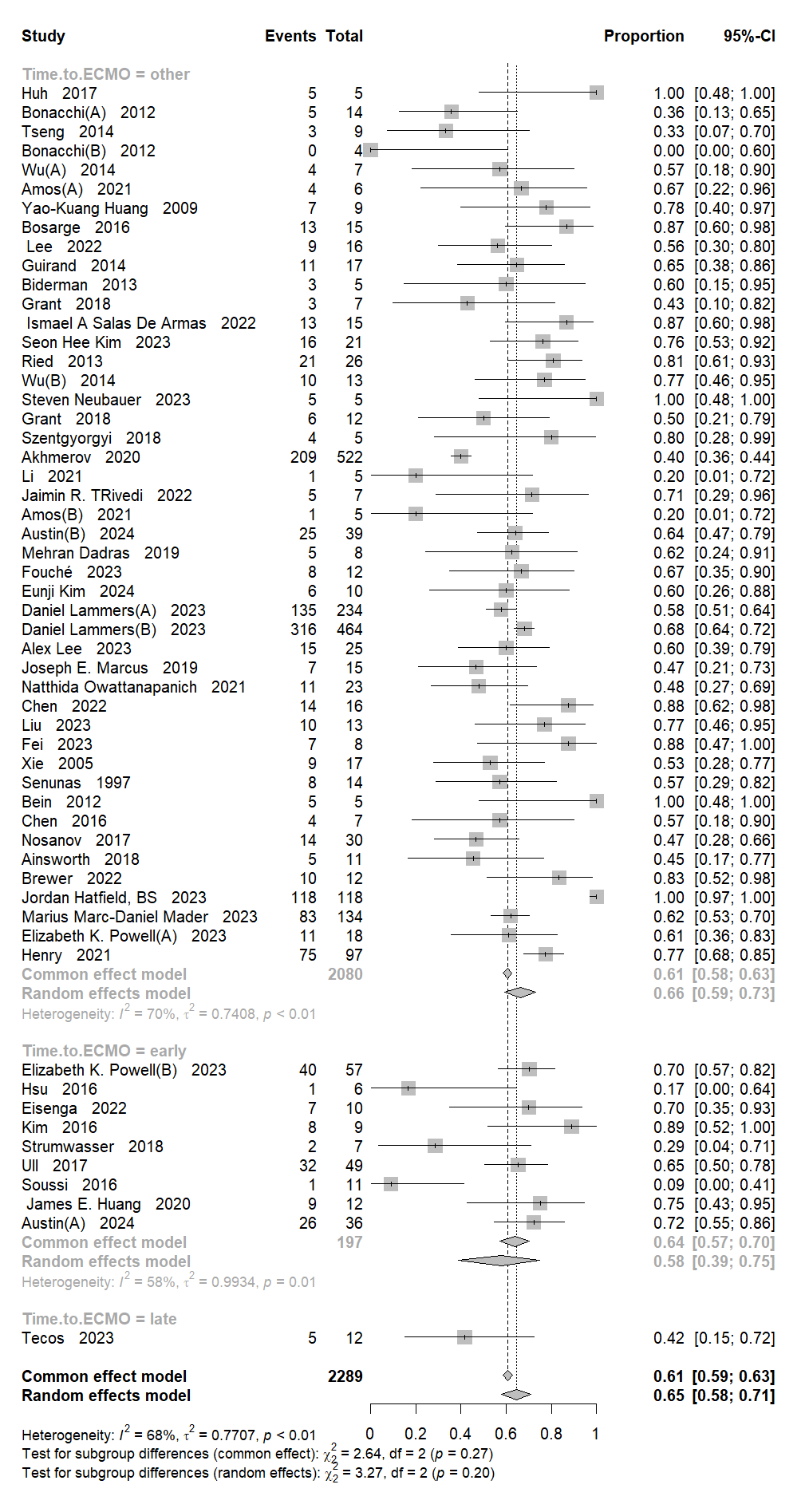


**Figure S44.** Forest plot of overall survival rate of adult trauma patients placed on ECMO within 5 days from admission to ECMO initiation.

CI: confidence interval; ECMO: Extracorporeal Membrane Oxygenation.


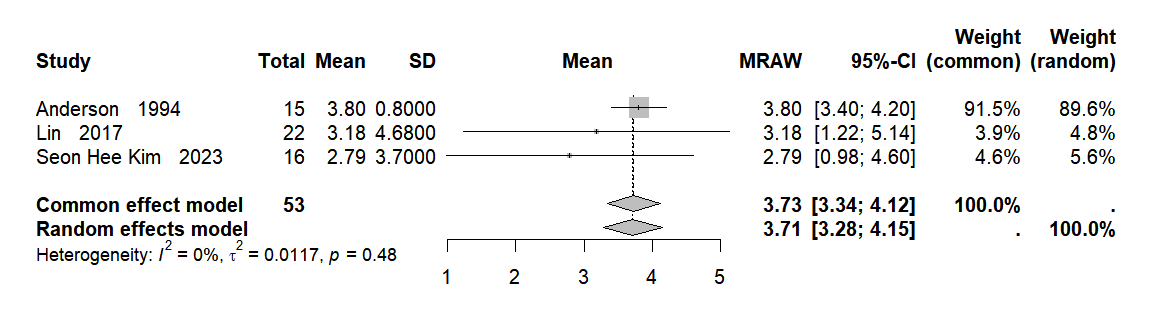


**Figure S45.** Forest plot of duration of time on ventilator prior to ECMO initiation for survivors.

CI: confidence interval; SD: Standard Deviation; MRAW: Mean of Raw Values; ECMO: Extracorporeal Membrane Oxygenation.


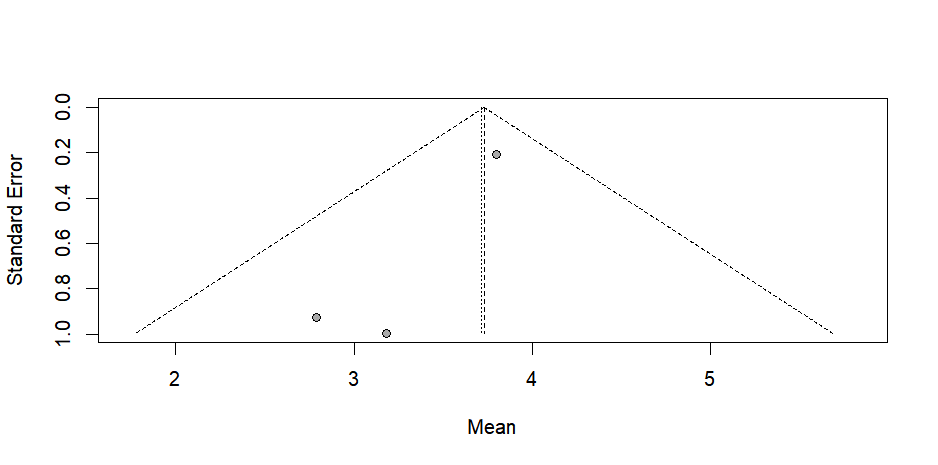


**Figure S46.** Funnel plot of duration of time on ventilator prior to ECMO initiation for survivors.


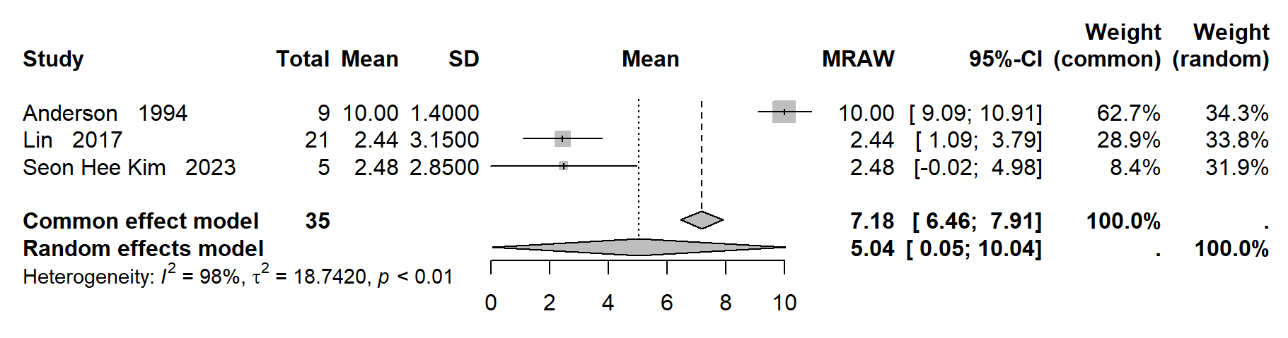


**Figure S47.** Forest plot of duration of time on ventilator prior to ECMO initiation for non-survivors.

CI: confidence interval; SD: Standard Deviation; MRAW: Mean of Raw Values; ECMO: Extracorporeal Membrane Oxygenation.


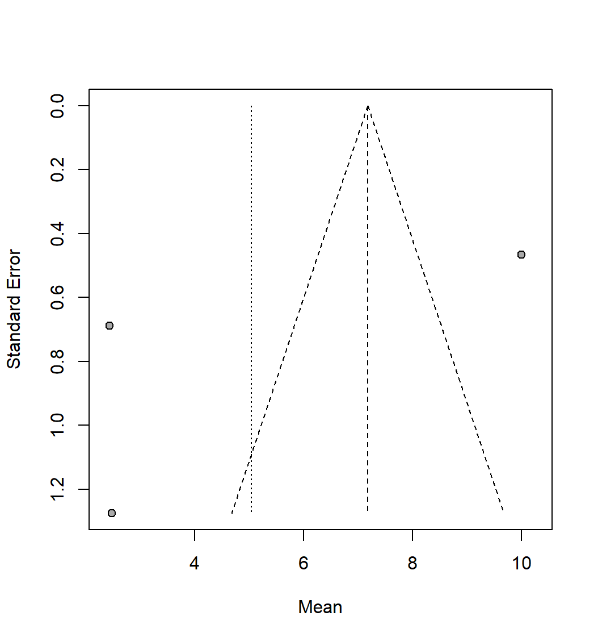


**Figure S48.** Funnel plot of duration of time on ventilator prior to ECMO initiation for non-survivors.


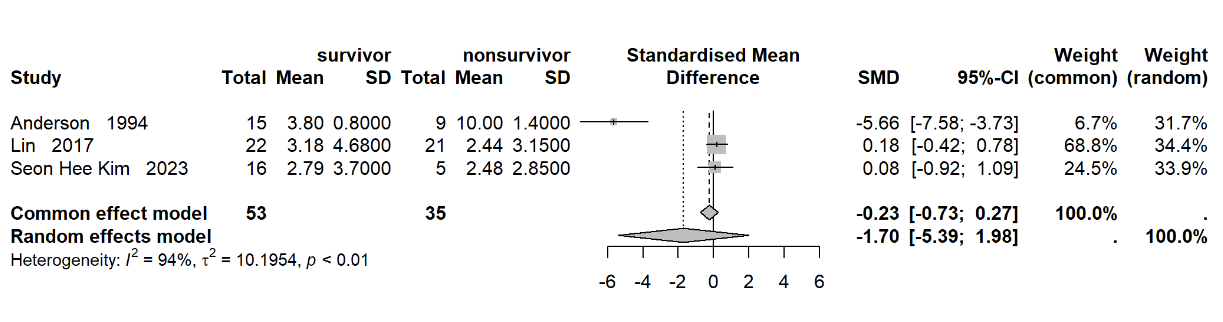


**Figure S49.** Forest plot of the difference in duration of time on ventilator prior to ECMO initiation for survivors and non-survivors.

CI: confidence interval; SD: Standard Deviation; ECMO: Extracorporeal Membrane Oxygenation.


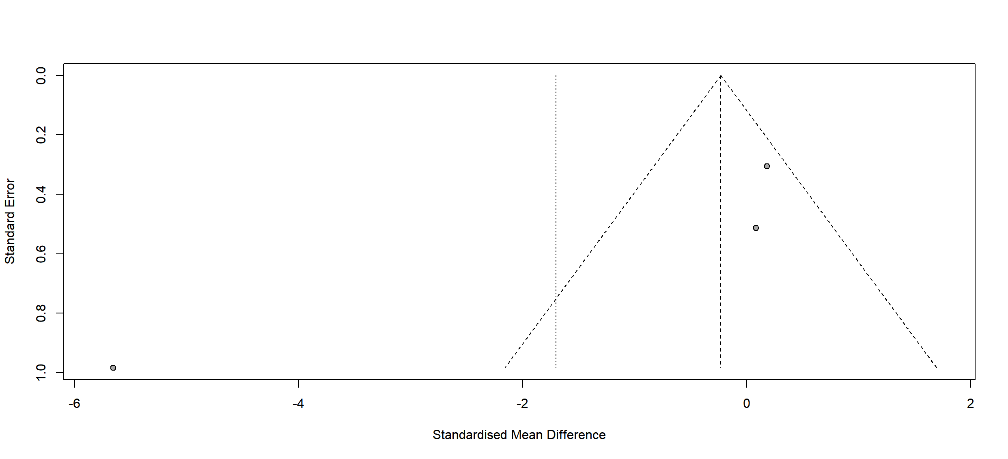


**Figure S50.** Funnel plot of the difference in duration of time on ventilator prior to ECMO initiation for survivors and non-survivors.

**Figure S51-52. Domain 4 subgroup meta-analyses.**

**Domain 4. Multidisciplinary approach**

##
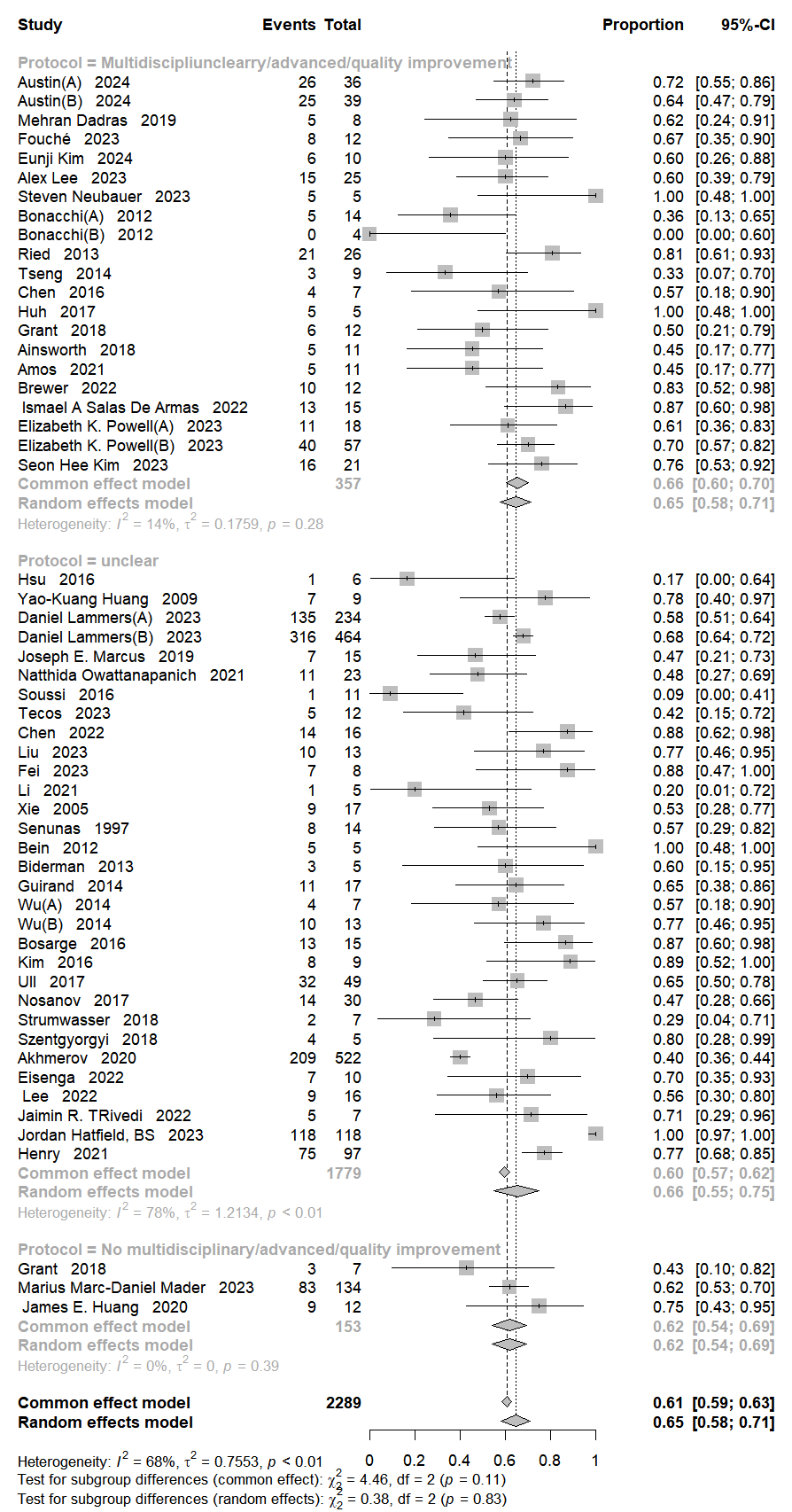


**Figure S51.** Subgroup analysis of overall survival rate based on whether an interdisciplinary approach is employed.

CI: confidence interval.


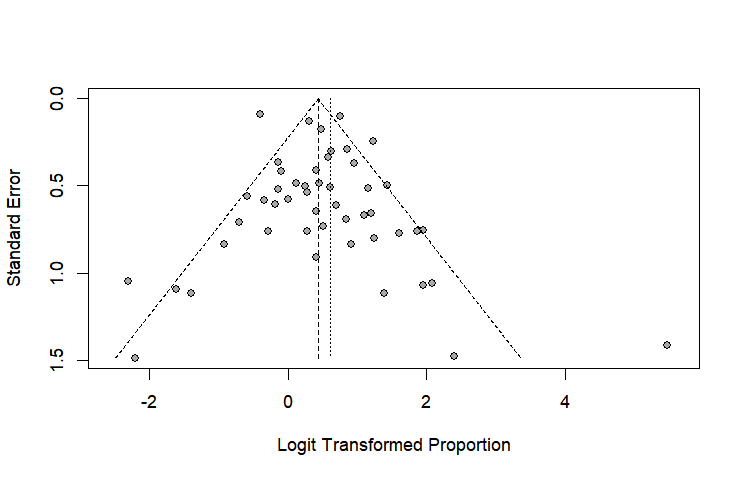


**Figure S52.** Funnel plot of subgroup analysis of overall survival rate based on whether an interdisciplinary team is employed.

**Table S8. Summary of anticoagulation strategy**

| **Study_ID** | **1st author** | **Year of publication** | **Study period** | **Country/district(hospital)** | **Data bank** | **Study design** | **Group** | **Sample size** | **ECMO pts size** | **Age (yrs)** | **Male (n)%** | **ECMO mode (n)** | **anticoagulation strategy** | **Anticoagulant monitoring index** | **Range of anticoagulation target (s)** | **Anticoagulation related complications** | **ECMO related complications** | **Death of cause** |
| --- | --- | --- | --- | --- | --- | --- | --- | --- | --- | --- | --- | --- | --- | --- | --- | --- | --- | --- |
| 7 | Austin | 2024 | 2014.1-2022.8 | America | NA | case-control study | TBI | 36 | 36 | 29 (23.5, 39) | 81.00% | VV ECMO 36 | Heparin | PTT | 45-55 | Bleeding 17, clotting 10 | Bleeding 17, clotting 10, Neurologic decline 5 | poor neurologic prognosis 3, multi-organ failure with escalating vasopressor requirements 3 |
| 7 | Austin | 2024 | 2014.1-2022.8 | America | NA | case-control study | nonTBI | 39 | 39 | NA | NA | VV ECMO 39 | Heparin | PTT | 45-55 | NA | NA | NA |
| 14 | Mehran Dadras | 2019 | 2017.1-2019.1 | Germany | NA | case series | NA | 8 | 8 | 48(34,58) | 75% | VV ECMO （8） | Heparin anticoagulation | partial thrombo-plastin time、ATIII | 40-50s、80-100% | thoracic hemorrhage 1 | thoracic hemorrhage(1)  acute kidney failure (4) | Sepsis (3) |
| 23 | Yao-Kuang Huang | 2009 | 2004.3-2007.10 | Taiwan, China | NA | case series | NA | 9 | 9 | 37(26.5, 44) | NA | VV-ECMO (7) VA-ECMO (2) | Heparin was prescribed at 10–15 units/(kg h) after ECLS and titrated for an activated coagulation time (ACT) of 180–250 s and an activated partial thromboplastin time (aPTT) of 55–60 s to prevent thrombo-embolic events.If hemorrhage was a concern after ECLS was deployed,we did not use any anti-fibronlytics. Instead, the heparin washeld for 12–24 h, the ACT and APTT levels were closely monitoredand the ECLS circuit was changed if circus thrombus developed | ACT、APTT | 180-250s、55-60s | NA | NA | prolonged shock and irreversible liver failure（1）  occult colon rupture and overwhelming peritonitis and sepsis （1） |
| 28 | Eunji Kim | 2024 | 2011.11~2022.01 | Pusan National University School of Medicine, Korea. | NA | Cohort study | ECLS | 10 | 10 | 53.00(38.25, 73.50) | 80% | NA | heparin | ACT | 150–200 | NA | NA | MOF (2), refractory hypoxia despite postoperative veno-venous ECMO (1), septic shock (1). |
| 28 | Eunji Kim | 2024 | 2011.11~2022.01 | Pusan National University School of Medicine, Korea. | NA | Cohort study | No ECLS | 18 | 0 | 47.50(34.25,58.50) | 72.20% | NA | heparin | ACT | 150–200 | NA | NA | NA |
| 30 | Alex Lee | 2023 | 2014.01~2021.02 | British | British Columbia Trauma Registry (BCTR) | case series | NA | 25 | 25 | 38.8(mean) (range, 19–67) | 76% | VV-ECMO (18), VA-ECMO (7) | Indeed, our standard practice for most GE patients has been a heparin bolus of50 units/kg IV. | NA | NA | NA | cannula related vascular injuries (4), Circuit clots requiring exchange(3), Circuit clot-failure of system(1). | Ongoing hemorrhagic shock （2）, Delayed hepatic failure （1）, Ongoing cardiogenic shock or arrest (2), Refractory status epilepticus (1), Severe anoxic brain injury (1), NA (3) |
| 36 | Steven Neubauer | 2023 | 2018.03~2020.03 | St Elizabeth Healthcare Center | NA | case series | NA | 5 | 5 | 27 (22.5,51) | 100% | VV-ECMO 5 | continuous infusion of heparin | ACT、PTT | ACT 170-220s，PTT 70-100s | Hemorrhage (2) | Hemorrhage (2), pneumothorax (1), asystolic event (1), and stroke (1). | NA |
| 38 | Michael J. Perchinsky, | 1995 | NA | Emanuel Hospital and Health Center | NA | case series | NA | 6 | 6 | NA | NA | NA | A simplified extracorporeal cardiopulmonary life support (ECLS) system was assembled consisting of a centrifugal pump head, heat exchanger, membranous oxygenator, percutaneous cammlas, and heparin-bonded circuitry. The entire system has heparin-bonded surfaces. | PLT, PT, PTT, FIB | NA | NA | surgical bleeding and coagulopathies 6 | NA |
| 50 | Chen | 2022 | 2000.1~2021.12 | China | NA | cohort study | ECMO | 16 | 16 | 39. 2±11. 1 | 62.50% | VV-ECMO (16) | heparin | ACT | 140–200s | NA | NA | Multi organ failure （1），Massive gastrointestinal bleeding （1） |
| 50 | Chen | 2022 | 2000.1~2021.12 | China | NA | cohort study | Ventilator | 21 | 0 | 36. 3±10. 2 | 61.90% | 0 | NA | NA | NA | NA | NA | Respiratory failure caused by bilateral severe pulmonary infection （3），liver failure （2），kidney failure （1) |
| 51 | Liu | 2023 | 2019.7~2022.6 | China | NA | case series | NA | 13 | 13 | 43.8±17.6 | 92.30% | VV-ECMO (13) | unfractionated heparin | ACT、APTT | 160-200s、50-70s | cerebral hemorrhage （1） | cerebral hemorrhage 1 kidney failure 1 bloodstream infection 2  Septic shock 2 lung infection 5 | Septic shock （2），cerebral hemorrhage （1） |
| 52 | Fei | 2023 | 2019.8~2022.8 | China | NA | case series | NA | 8 | 8 | 42.857±21.318 | 87.50% | VA-ECMO (4) VV-ECMO (4) | low-dose heparin anticoagulation，no heparin anticoagulation | ACT | 110-200s | limb intermuscular vein thrombosis （3）， peroneal vein thrombosis （2），femoral artery thrombosis （1） | limb intermuscular vein thrombosis 3， peroneal vein thrombosis 2，femoral artery thrombosis 1 | NA |
| 53 | Jiang | 2005 | 1997.3~2005.3 | China | NA | cohort study | ECMO | 12 | 12 | 49.04±12.09 | 83.30% | VA-ECMO 12 | unfractionated heparin | ACT | 150-230s | 0 | NA | hypoxic-ischemic encephalopathy （1），Aspergillus infection （1） |
| 53 | Jiang | 2005 | 1997.3~2005.3 | China | NA | cohort study | non-ECMO | 14 | 0 | NA | NA | 0 | NA | NA | NA | NA | NA | NA |
| 54 | Li | 2021 | 2014.3-2020.7 | China/the First Affiliated Hospital of Army  Medical University (the Third Military Medical University) | NA | case series | NA | 5 | 5 | 48.2±7.5736 | 100% | VV-ECMO 5 | Heparin | APTT | 60-80 | NA | NA | MODS、Septic shock 4 |
| 55 | Xie | 2005 | 2002.6-2004.8 | China/Zhongshan People's Hospital | NA | case series | NA | 17 | 17 | 23.3±0.7 | 64.70% | VV-ECMO | Small doses of heparin, 20 to 30 mg each time | APTT | 160 | 0 | NA | secondary brainstem injury 3, massive pulmonary hemorrhage 1, MODS 4 |
| 61 | Anderson | 1994 | 1988.5-1993.8 | the University of Michigan Medical center | NA | case series | survivors | 15 | 15 | NA | NA | VA-ECMO 3, VV-ECMO 8, VA-ECMO-to-VV-ECMO 3、VV-ECMO-to-VA-ECMO 3 | Continuous systemic heparinization | ACT | 170-190 | Bleeding 9 | Bleeding 9, Renal failure 2, Seizure 1, Cerebral infarcthemorrhage 3, Pneumothorax 2, Oxygenator failure 1, ECLS circuit change 5 | 0 |
| 61 | Anderson | 1994 | 1988.5-1993.8 | the University of Michigan Medical center | NA | case series | Nonsurvivors | 9 | 9 | NA | NA | VA-ECMO 1, VV-ECMO 5, VV-ECMO-to-VA-ECMO 3 | Continuous systemic heparinization | ACT | 170-190 | Bleeding 9 | Bleeding 9, Renal failure 3, Arrhythmiacardiac arrest 3, Cerebral infarcthemorrhage 2, Oxygenator failure 1, Racewaytubing rupture 2, Pump failure 1, ECLS circuit change 1 | NA |
| 62 | Senunas | 1997 | 1988-1994 | the University of Michigan Medical Center | NA | case series | NA | 14 | 14 | 19.07±9.34 | 28.60% | NA | Heparin anticoagulation | ACT | 170-190 | Bleeding 7 | Bleeding 7, Cardiac arrest 1, Seizure 2, pneumothorax 1, acute renal failure 4, ECLS circuit change required 2 | NA |
| 64 | Cordell-Smith | 2006 | 1992.5-2000.11 | England | NA | case-control study | survivors | 20 | 20 | 27(mean) | NA | VV-ECMO 20 | Heparin anticoagulation | ACT | 180-220 | NA | NA | 0 |
| 64 | Cordell-Smith | 2006 | 1992.5-2000.11 | England | NA | case-control study | Nonsurvivors | 8 | 8 | 28(mean) | NA | VV-ECMO 4, VV-ECMO-to-VA-ECMO 4 | Heparin anticoagulation | ACT | 180-220 | NA | NA | irreversible cardiogenic failure 4, sepsis 4 |
| 67 | Bein | 2012 | 2005.6-2011.8 | America | NA | case series | VV-ECMO | 5 | 5 | 23.4 ± 3.647 | NA | VV ECMO 5 | Heparin-bonded surfaces, decreased blood trauma through the circuit, and complete membrane separation of the blood and gas flows reduced the systemic anticoagulation requirements, allowing a target partial thromboplastin time (aPTT) of 1.5 times the normal | APTT | aPTT 1.5 X normal | heparin-induced thrombocytopenia(1) | heparin-induced thrombocytopenia（1） | 0 |
| 68 | Biderman | 2013 | NA | Israel | Rabin Medical Center | case series | ECMO | 5 | 5 | 28.4±4.39 | 60.00% | NA | In patients with substantial risk of bleeding, no anticoagulation was given until the risk of bleeding decreased. In those patients, we kept high ﬂow on the ECMO to prevent clot formation.In those patients who could not receive heparin(TBI), high blood flow (4Y5 L/min) ;was maintained to prevent clotting. | Afull blood count and coagulation proﬁle are drawn during initial cannulation. Coagulation is titrated with activated clotting time | NA | NA | NA | NA |
| 69 | Bonacchi | 2012 | 2008.12-2012.5 | Careggi Teaching Hospital, Florence, Italy | NA | case-control study | ECLS success | 14 | 14 | 47±17.6 | 71.00% | VA-ECMO(10)，VV-ECMO(4) | In all cases, because of actual or potential bleeding risk, we initially performed heparin-free ECLS until bleeding stopped and normalization of patient coagulative status was achieved | activated partial thromboplastin time range | 50 to 60 seconds | NA | leg ischemia due to femoral artery cannulation 1  oxygenator failure due to clot formation 1 | Cerebral death and（7）；septic multiple organ failure（2） |
| 69 | Bonacchi | 2012 | 2008.12-2012.5 | Careggi Teaching Hospital, Florence, Italy | NA | case-control study | ECLS failure | 4 | 4 | 43±19.7 | 50.00% | VV-ECMO(4) | In all cases, because of actual or potential bleeding risk, we initially performed heparin-free ECLS until bleeding stopped and normalization of patient coagulative status was achieved | activated partial thromboplastin time range | 50 to 60 seconds | NA | NA | NA |
| 70 | Ried | 2013 | 2002.04-2012.04 | Regensburg, Germany | Regensburg ECMO Registry database | cohort study | VV-ECMO | 26 | 26 | 29.3 ± 13.2 | 92.00% | vv-ECMO(26) | In all ELS devices, heparin-coated cannulas and circuits were used to decrease the systemic heparin dosage and to reduce post-traumatic bleeding complications；Our anticoagula- tion protocol used for vv-ECMO is also based on contin- uous heparin infusion, beginning with 600 IU/hour。 | PTT | 40-50s | NA | Cannula-related complications (3) | Leading causes of death were multiorgan failure (n =9), fulminant bleeding (n =1) and cerebral hypoxia and bleeding with entrapment (n =1) |
| 71 | Guirand | 2014 | 2001.01-2009.12 | America | Wake Forest School of Medicine ECLS Registry. Los Angeles County + University of Southern California (LAC + USC) Medical Center trauma and SICU database. | cohort study | VV-ECMO | 17 | 17 | 30.9 ± 11.4 | 71% | **VV-ECMO(17)** | Full anticoagulation was achieved by titrating a continuous infusion of unfractionated heparin | ACT | 180-220S | ECLS patients received more blood transfusions and had more bleeding complications | Hemorrhagic 3;AKI 16 | one patient death attributable to hemorrhage |
| 71 | Guirand | 2014 | 2001.01-2009.12 | America | Wake Forest School of Medicine ECLS Registry. Los Angeles County + University of Southern California (LAC + USC) Medical Center trauma and SICU database. | cohort study | CONV | 17 | 0 | 34.1 ± 10.7 | 88% | 0 | Full anticoagulation was achieved by titrating a continuous infusion of unfractionated heparin | ACT | 180-220S | ECLS patients received more blood transfusions and had more bleeding complications | Pneumonia3 ; AKI 16 | NA |
| 72 | Tseng | 2014 | 2003.11-2012.10 | Taiwan, China | NA | case series | NA | 9 | 9 | 37（26.5,46） | 88.90% | VA-ECLS(9) | 1、The ECLS device used was the Capiox emergent bypass system (EBS, Terumo Inc., Tokyo, Japan) with heparin-coated inner surface. 2、Patients with a low risk of bleeding would accept a load- ing dose of heparin (5000 unit) just before cannulation；3、In patients with a high risk of bleeding, a “heparin-mini- mized” strategy (no loading or maintaining dose of heparin) would be adopted. The length of the “heparin- free” strategy was not exceeded 48 h in our practice and ahigh-flow ECLS (blood flow > 2.5 L/min) must be maintained to reduce the risk of thrombosis. | ACT、aPTT | 160-180 and 40-55 seconds | uncontrolled retroperitoneal hemorrhage (1) 、hemothorax (1). | uncontrolled retroperitoneal hemorrhage（1） and hemothorax （1）； | NA |
| 73 | Wu | 2014 | 2004.1-2013.6 | Taiwan, China，Chang Gung Memorial Hospital | NA | case-control study | Survivors | 14 | 14 | 41 (29,57) | NA | VV ECMO 14 | ACT and aPTT were checked at least every 6 hour in the first day of VV. In heparin-titrated ECLS, ACT/aPTT value was 160–180/40–55 seconds. In heparin-minimized ECLS received no exogenous heparinization. The heparin-minimized ECLS maintained a high blood flow rate (>3 L/min) and was switched to the heparin-titrated mode after 48 hours to reduce the possibility of thromboembolism. To achieve acceptable oxygenation and to reduce the bleeding risk, hemaglobin and platelet count were also checked daily to maintain a level more than 10 g/dL and 80 billion/L, espectively. Coagulation factor transfusion was seldom needed in patients without hemorrhage. In patients with hemorrhages, withholding heparin plus blood transfusion (RBC: plasma: platelet about 1:1:3) was the first step to achieve hemostasis on ECLS." | ACT/aPTT/aPTT value | 1、High Risk of Hemorrhagic Complications on ECLS:160–180/40–55s ; 2\plications on ECLS:A prolonged ACT/aPTT (160-180/40-55 sec) : | NA | NA | 0 |
| 73 | Wu | 2014 | 2004.1-2013.6 | Taiwan, China，Chang Gung Memorial Hospital | NA | case-control study | Nonsurvivors | 6 | 6 | 30 (22,61) | NA | VV ECMO 6 | 1、High Risk of Hemorrhagic Complications on ECLS:Heparin-minimized VV-ECLS Circuitry priming dose : 2500 u/1L Loading dose before cannulation : Not used A prolonged ACT/aPTT :not maintained; 2、No high Risk of Hemorrhagic Complications on ECLS:Heparin-titrated VV-ECLS Circuitry priming dose : 2500 u/1L . Loading dose before cannulation : 5000 u · A prolonged ACT/aPTT (160-180/40-55 sec) : maintained by heparin infusion (from 500u/h). | ACT/aPTT/aPTT value | 1、High Risk of Hemorrhagic Complications on ECLS:160–180/40–55s ; 2\plications on ECLS:A prolonged ACT/aPTT (160-180/40-55 sec) : | NA | NA | NA |
| 74 | Bosarge | 2016 | 2012.03-2014.11 | Birmingham, Alabama, University of Alabama at Birmingham (UAB) | NA | cohort study | ECMO | 15 | 15 | 36.0 (25.0 ,47.0) | 100% | Venovenous (V/V) in 10 patients, venoarterial (V/A) in 3, venoarterial venous (V/A/V) in 2 | The anticoagulation protocol at UAB for all ECMO patients is to maintain a low dosing of heparin, which is monitored with thromboelastogram (TEG). | TEG time | two times the nonheparinized baseline | Of 15 patients (40%), 6 had hemorrhagic complications, which included persistent epistaxis requiring nasal packing, gastrointestinal bleeding, surgical incision or cannula insertion site bleeding, and expanding hematomas | Bleeding(6); RIJ thrombus(3); SVC occlusion(1); RUE thrombus(1) | Multiorgan failure（1）；Hypoxia（1） |
| 74 | Bosarge | 2016 | 2012.03-2013.2 | Birmingham, Alabama, University of Alabama at Birmingham (UAB) | NA | cohort study | CONV | 14 | 0 | 40.0 (23.0, 47.0) | 92.90% | 0 | NA | NA | NA | NA | NA | Multiorgan failure（4）；Hypoxia（4）；Septic shock（1） |
| 75 | Wu | 2015 | 2008.1~2014.1 | Taichung,China | NA | case-control study | Survivors | 13 | 13 | 33.8±16.4 | 100% | VV-ECMO(8),VA-ECMO(5) | Use of heparin,Use of CVVH | ACT | ACT maintained 180 - 200s. and 140-160s in patients with coagulopathy and bleeding. | NA | NA | 0 |
| 75 | Wu | 2015 | 2008.1~2014.1 | Taichung,China | NA | case-control study | Nonsurvivors | 6 | 6 | 55.8±14.7 | 66.70% | VV-ECMO(1),VA-ECMO(5) | Use of heparin,Use of CVVH | ACT | ACT maintained 180 - 200s. and 140-160s in patients with coagulopathy and bleeding. | coagulopathy(2) | persistent hemorrhage and coagulopathy （2） | pneumonia(3),  coagulopathy(2), cardiac rupture with cardiac tamponade(1) |
| 76 | Chen | 2016 | 2009.9~2012.9 | Taipei, China | NA | case series | NA | 7 | 7 | 31 (21,49) | 85.70% | VV-ECMO(7) | All trauma patients are maintained heparin-free until clinical signs of bleeding can be controlled. Afterwards, minimal (or none) systemic heparinization is used to meet the target activated coagulation time (ACT) at 170 seconds. | ACT | 170s | NA | ARF,4;Acute pancreatitis,1;Brain swelling,2;Sepsis,1;Transient memory deficit,1; Nil,2;Acute lung edema,1;ACS,1. | Irreversible brain damagewith vasodilatory shock,1; Septic shock with MOF,1; Irreversible brain damagewith vasodilatory shock,1 |
| 77 | Kim | 2016 | 2007.1~2015.3 | Anyang,Korea | NA | case series | trauma | 9 | 9 | 48.0 (20.5,62.0) | 88.90% | VV-ECMO(9) | heparin or nafamostat mesilate | APTT | 60-80 s | NA | NA | NA |
| 79 | Ahmad | 2017 | 2006.1~2015.11 | Baltimore,Maryland | NA | case-control study | survivors | 17 | 17 | 35 (25, 45) | 62% | VV-ECMO(17) | Ninety-four percent of the survivors were anticoagulated with heparin versus 55% of nonsurvivors (p = 0.01) | ACT,PTT | ACT(160-180),PTT(60-80,or45-55) | NA | NA | 0 |
| 79 | Ahmad | 2017 | 2006.1~2015.11 | Baltimore,Maryland | NA | case-control study | Nonsurvivors | 22 | 22 | 27 (22, 42) | 82% | VV-ECMO(22),VA-ECMO(7) | Ninety-four percent of the survivors were anticoagulated with heparin versus 55% of nonsurvivors (p = 0.01) | ACT,PTT | ACT(160-180),PTT(60-80,or45-55) | NA | NA | cardiorespiratory ,10 ; brain death, 1;died after withdrawal of care,11. |
| 81 | Huh | 2017 | 2015.4 ~2016.5 | Busan, Korea | NA | case series | NA | 5 | 5 | 39.4±8.53 | 80% | NA | heparin | ACT | 150-200 | NA | Cerebral infarction 2 | NA |
| 82 | Lin | 2017 | 2006.3 ~2016.7 | Taiwan, China | NA | case-control study | Survivors | 22 | 22 | 40.5±14.9 | 86.40% | VV-ECMO(22) | Heparin-coated cannula (Medtronic,Bio-Medicus,USA)ensured superior biocompatibility and could be heparinfree in the first 12hours of deployment if bleeding was a concern. heparin was held for 12–24 hours, ACT and APTT levels were closely monitored, and the ECMO circuit was changed early, once thrombus was detected. | ACT and APTT | NA | Bleeding 8, Cerebral infarction 2, ICH 1 | Bleeding 8, Cerebral infarction 2, ICH 1, Limb ischemia 2, Sepsis 5 | 0 |
| 82 | Lin | 2017 | 2006.3 ~2016.7 | Taiwan, China | NA | case-control study | Mortality | 21 | 21 | 34.1±15.1 | 81% | VA-ECMO(17)、VV-ECMO(4) | Heparin-coated cannula (Medtronic,Bio-Medicus,USA)ensured superior biocompatibility and could be heparinfree in the first 12hours of deployment if bleeding was a concern. heparin was held for 12–24 hours, ACT and APTT levels were closely monitored, and the ECMO circuit was changed early, once thrombus was detected. | ACT and APTT | NA | Bleeding 9, Cerebral infarction 4, ICH 2 | Bleeding 9, Cerebral infarction 4, ICH 2, Limb ischemia 4, Sepsis 12 | NA |
| 85 | Grant | 2018 | 2016.6-2017.9 | America | NA | Cohort study | post-Advanced ECMO Program | 12 | 12 | 36.5 | 83% | VV-ECMO 11, VA-ECMO 1 | 58% Patients Received Heparin | PTT | 40-50S | Thrombosis/ischemia 2, Bleeding event 5 | Renal failure requiring dialysis 6, Liver failure 4, Brain death 1 , Thrombosis/ischemia 2, Bleeding event 5 | NA |
| 85 | Grant | 2018 | 2014.1-2016.5 | America | NA | Cohort study | pre-Advanced ECMO Program | 7 | 7 | 30 | 86% | VV-ECMO 5, VA-ECMO 2 | 86% Patients Received Heparin | NA | NA | Thrombosis/ischemia 3, Bleeding event 3 | Renal failure requiring dialysis 5, Liver failure 0, Brain death 1 , Thrombosis/ischemia 3, Bleeding event 3 | NA |
| 86 | Ainsworth | 2018 | 2012.9-2017.9 | America | NA | case series | NA | 11 | 11 | 37.45±15.58 | 81.20% | VV-ECMO 11 | heparin | ACT/anti Xa/aPTT | ACT: patients therapeutic with times of 160–180s if they were having issues with bleeding or 180–200s if they were not having issues with coagulopathy;/anti Xa: 0.2–0.4IU/ml for patients without bleeding complications. If patients are having a bleeding complication, we usually lower our goal level to 0.1–0.3 IU/ml. /aPTT : 45–60s | It was not possible to determine whether the burn patient had complications | NA | Multi-organ failure 5, Cerebral Hemorrhage 1 |
| 89 | Strumwasser | 2018 | 2016.12~2017.12 | America | NA | case series | NA | 7 | 7 | 41±15.03 | 100% | VV-ECMO 5, VA-ECMO 2 | unfractionated heparin | ACT/anti Xa/aPTT | ACT goal at 150–180 s or a PTT of 65–90 s. In the presence of hyperbilirubinemia or hemolysis, anticoagulation was to a targeted anti-Xa level of 0.4–0.8 IU/mL. | clotting of the ECMO circuit 3 | clotting of the ECMO circuit 3, hospital-acquired pneumonia 1 | Multisystem organ failure 2, Cardiopulmonary arrest 1, NA 2 |
| 90 | Szentgyorgyi | 2018 | 2011.12-2017.6 | Wythenshawe Hospital | NA | case series | NA | 5 | 5 | 33(29,35.5) | 60% | VV-ECMO 5 | All patients were anti-coagulated with heparin according to standard departmental protocol aiming to keep the ACT between 160 and 180s | ACT | 160-180 | NA | AKI 4; right internal jugular vein thrombus 1;neutropenic sepsis 1;gastrointestinal bleeding 1; | sepsis and multi organ failure 1 |
| 92 | Kruit | 2019 | 2011.12-2017.5 | the United Kingdom | the five National Respiratory ECMO centers in the United Kingdom | case-control study | Survivors | 44 | 44 | 33 (23, 45) | 84% | NA | Anticoagulation was commenced in 43 patients (84%); for 36 patients (65%), this was in the first 72 hours after ECMO commencement. | NA | NA | 总体：Bleeding 20(cerebral hemorrhage 3)，Thrombotic complications 16, | 总体：Bleeding 26, Thrombotic complications 21 (oxygenator failure (8), deep vein thrombosis (8), followed by pulmonary embolism (3)) | 0 |
| 92 | Kruit | 2019 | 2011.12-2017.5 | the United Kingdom | the five National Respiratory ECMO centers in the United Kingdom | case-control study | Deaths | 8 | 8 | 32 (26, 37) | 63% | NA | Anticoagulation was commenced in 43 patients (84%); for 36 patients (65%), this was in the first 72 hours after ECMO commencement. | NA | NA | 总体：Bleeding 20(cerebral hemorrhage 3)，Thrombotic complications 16, | 总体：Bleeding 26, Thrombotic complications 21 (oxygenator failure (8), deep vein thrombosis (8), followed by pulmonary embolism (3)) | Cerebral herniation 2, Massive hemorrhage 1, Mesenteric ischemia 1, Cardiac arrest 1, Unknown 3 |
| 93 | Lang | 2019 | 2002-2016 | Medical University of Vienna | NA | case-control study | Survivors | 4 | 4 | 26.25±8.88 | 0.00% | VA-ECMO 4 | Depending on blood coagulation and severity of injuries, heparin-free ECLS, delaying heparin administration for up to 48 hours, or an initial bolus of 500IE/h heparin was started soon after transfer to ICU. A therapeutic level of the aPTT of 60–65 seconds was aimed. LDH was checked as a surrogate parameter for hemolysis to reduce heparin dose if necessary. | APTT | 60-65 | NA | 总体（存活组+非存活组）：Local bleeding at the puncture site 1, Malpositioning of the Y-cannula 1, MOF 3, Diffuse intravascular coagulopathy 1, Hypoxic cerebral edema 1, Vascular injury 1 | 0 |
| 93 | Lang | 2019 | 2002-2016 | Medical University of Vienna | NA | case-control study | Nonsurvivors | 12 | 12 | 37.583±15.957 | 41.70% | VV-ECMO 1, VA-ECMO 11 | Depending on blood coagulation and severity of injuries, heparin-free ECLS, delaying heparin administration for up to 48 hours, or an initial bolus of 500IE/h heparin was started soon after transfer to ICU. A therapeutic level of the aPTT of 60–65 seconds was aimed. LDH was checked as a surrogate parameter for hemolysis to reduce heparin dose if necessary. | APTT | 60-65 | Intravascular diffuse coagulopathy 1 | 总体（存活组+非存活组）：Local bleeding at the puncture site 1, Malpositioning of the Y-cannula 1, MOF 3, Diffuse intravascular coagulopathy 1, Hypoxic cerebral edema 1, Vascular injury 1 | MOF 3, Intravascular diffuse coagulopathy 1, Hypoxic cerebral edema 1 |
| 94 | Lee | 2020 | 2007.01-2018.12 | Hallym University Sacred Heart Hospital | NA | case-control study | NA | 42 | 42 | 41 (18.75,52.75) | 88.10% | VV-ECMO 29, Other types 13 | During ECMO support, heparin or nafamostat mesilate was used for anticoagulation, with a target activated partial thromboplastin time (aPTT) of 60–80 s. Anticoagulation was usually initiated after confirmed bleeding control was achieved for 48 h in patients with a high risk of bleeding or those who underwent surgery. | APTT | 60-80 | Leg ischemia 1;Ulcer bleeding 1 | Leg ischemia 1;Bed sore 2 ;ARF (creatinine > 2 mg/dL) 11 ; Cholecystitis 2; Ulcer bleeding 1;CNS injury 1 ;Multiorgan failure 2 | NA |
| 97 | Amos | 2021 | 2010.01-2020.06 | The Alfred | NA | cohort study | NA | 11 | 11 | 39±17.8 | 91% | VV ECMO 7, VA ECMO 3, VV ECMO to VAV ECMO to VV ECMO 1 | Heparin free (3/11), delayed (3/11) or low dose heparin (2/11) ；Heparin（3/11）. | NA | NA | Haemorrhage 4 | Haemorrhage 4, Venous thrombosis 3, Limb ischaemia 2, Ischaemic stroke 1, Circuit change due to thrombosis 2, Pump failure 1 | Multi-organ failure 4, Uncontrollable haemorrhage 1, Traumatic brain injury 1 |
| 99 | Parker | 2021 | NA | USA | NA | case series | NA | 13 | 13 | 28（25,37.5） | 85% | VV-ECMO (13) | heparin | APTT | 45-55s | Circuit thrombosis(4),bleeding from a thoracic excision （1）epistaxis（1） | Circuit thrombosis(4),bleeding from a thoracic excision （1）epistaxis（1） | multisystem organ failure(4),removal of life sustaining therapy( 3), death by neurologic criteria(1) |
| 102 | Thani | 2022 | 2014.01-2020.01 | Qatar | NA | Cohort study | ECMO | 22 | 22 | 29.6 ± 13.8 | 86.40% | VV-ECMO (21), VA-ECMO (1) | In case of traumatic brain injury (TBI), no heparin was given for 48–72 h post-trauma | NA | NA | NA | NA | Head injury（3）;Multiorgan failure（3 ）;Septic shock,（1）;Multiorgan failure（3 ）;Septic shock(1);Cardiac arrest (1 ) |
| 102 | Thani | 2022 | 2014.01-2020.01 | Qatar | NA | Cohort study | non-ECMO | 63 | 0 | 35.9 ± 15.1 | 93.70% | 0 | NA | NA | NA | NA | NA | Head injury(17);Multiorgan failure(6 );Septic shock(4);Multiorgan failure(6);Septic shock(4 ) |
| 103 | Brewer | 2022 | 2013.10-2020.2 | USA/Connecticut | NA | case series | Trauma ECMO patients | 12 | 12 | 33.6 ± 4.0 | NA | VV-ECMO 11, VA-ECMO 1 | Systemic anticoagulation | ACT、anti-Xa | >150s、0.3–0.7 | NA | NA | NA |
| 105 | Lee | 2022 | 2013.1-2017.12 | Korea | NA | Cohort study | VV-ECMO | 16 | 16 | 47.5 (34.3, 71.3) | 81.30% | VV ECMO16 | Systemic anticoagulation with unfractionated heparin was administered as an initial bolus of 50–100units/kg at the time of cannulation, followed by a continuous infusion of 20–50units/kg/h to achieve an activated clotting time of 160–220s.  Nafamostat mesilate (Futhan®, SK chemicals, Seoul, Korea) was used as an alternative anticoagulant to heparin for trauma patients with coagulopathy or high bleeding risk | ACT | 160–220s | NA | 0 | Septic shock （2）;MOF （2 ） ,Brain death （2 ） ;Cardiac arrest （1） |
| 105 | Lee | 2022 | 2013.1-2017.12 | Korea | NA | Cohort study | Conventional mechanical ventilation | 32 | 0 | 58.0（49.0,70.5） | 65.60% | 0 | NA | NA | NA | NA | NA | Septic shock （8）;MOF （6） ,Brain death （2） ;Cardiac arrest （1） |
| 106 | Ismael A Salas De Armas | 2022 | 2015.6-2018.8 | US/TX | NA | case series | NA | 15 | 15 | 30.6±12.6 | 93% | VV-ECMO （15） | Heparin(80%)、lirudin（20%） | PTT | 40-60s | NA | DVT 2 falcine, subdural hemorrhage and diffused cerebral edema 1 frontal infarct with hemorrhagic conversion and midline shift 1 | NA |
| 107 | Jaimin R. TRivedi | 2022 | 2016-2019 | US/Kentucky | NA | case series | NA | 7 | 7 | 32.1±8.7 | 100% | VA-ECMO （2） 、VV-ECMO （5） | All the ECMO patients receive heparin with adjustment based on their coagulation profile and ensuing complications. | NA | NA | bleeding or ECMO circuit issues 3 | pulmonary infections 4  shock liver 1 acute renal failure 4 hypofibrinogenemia 1 thrombocytopenia 1 continued blood loss 1 hip disarticulation 1 limb ischemia 1  rhabdomyolysis 1 pulmonary embolus 1 stroke 1 anoxic brain injury 1 | NA |
| 108 | Weidemann | 2022 | 2011.4-2019.4 | German/Hannover | NA | case series | NA | 19 | 19 | 28±11 | 78.90% | VV-ECMO (13), VA-ECMO-to-VV-ECMO (5),VAV-ECMO-to-VV-ECMO (1) | Heparin、ATⅢ | ACT、ATⅢ | 160-180、80%-100% | massive bleeding from surgical wounds 1 | massive bleeding from surgical wounds 1  another patient developed right heart failure 1 | traumatic brain injury 3  ARDS 3 multiple organ failure including ARDS 3 |
| 113 | Elizabeth K. Powell | 2023 | 2014.1.1-2022.8.1 | US/MD | NA | cohort study | Non Early VV ECMO | 18 | 18 | NA | NA | VV-ECMO (18) | heparin | PTT | 45-55 | NA | NA | NA |
| 113 | Elizabeth K. Powell | 2023 | 2014.1.1-2022.8.1 | US/MD | NA | cohort study | Early VV ECMO | 57 | 57 | 29 (22,40) | 81% | VV-ECMO (57) | heparin | PTT | 45-55 | NA | NA | NA |
| 111 | Seon Hee Kim | 2023 | 2017.3-2019.2 | Korea/Busan | NA | case-control study | NA | 21 | 21 | 45.0 ± 17.8 | 85.70% | VV-ECMO (21) | Anticoagulation was based on the ELSO Guidelines for Adult Respiratory Failure 2017.15 Activated clotting time (ACT) was determined in all patients before commencing ECMO. The heparin dose was adjusted to a target ACT of between 150 and 200 s considering the bleeding risk.13 In patients with a substantial risk of bleeding, no anticoagulation was administered until the bleeding risk decreased, and the ECMO was maintained at a high flow rate to prevent clot formation.The platelet count was maintained at >80,000. | 1、ACT；2、ECMO flow rate + platelet | 1、150-200；2、high flow rate + ＞80000 | 0 | NA | multi-organ failure 5 |
| new1 | Henry | 2021 | 2013-2016 | USA | TQIP datebase | Cohort study | ecmo | 97 | 97 | 35 (22,51) | 81% | NA | unfractionated heparin、low molecular weight heparin、No Anticoagulation、others | NA | NA | Deep Vein Thrombosis 17 Pulmonary Embolus 16 | Acute Kidney Injury 28 Myocardial Infarction 3 Stroke 4 Deep Vein Thrombosis 17 Pulmonary Embolus 16 Central Line Infection 1 Ventilator Associated Pneumonia 7 Sepsis 17 Superficial SSI 4 Deep SSI 1 Compartment syndrome 2 | NA |
| new3 | Menaker | 2018 | 2015.1-2016.11 | The R Adams Cowley Shock Trauma Center | NA | case-control study | NA | 18 | 18 | 28.5 ( 24,43) | NA | VV ECMO 18 | Fourteen (78%) patients received low-dose anticoagulation (PTT 45–55) while on ECMO. Four patients did not receive any anticoagulation while on ECMO; two due to profound coagulopathy, one due to a spinal cord injury and one patient who died prior to initiating heparin. | PTT | 45-55 | Bleeding 6, DVT 10 | Bleeding 6, DVT 10 | profound shock and coagulopathy 3 primary neurologic injury 1 |

**Table S9. Statistics of complications**

| **Study_ID** | **1st author** | **Year of publication** | **Group** | **Sample size** | **Anticoagulation related complications** | **ECMO related complications** | **BLEEDING TOTAL** | **unknown** | **thoracic hemorrhage** | **cerebral hemorrhage** | **retroperitoneal hemorrhage** | **bleeding at the puncture site** | **gastrointestinal bleeding** | **epistaxis** | **surgical site bleeding** | **bleeding from surgical wounds** | **CLOTTING TOTAL** | **unknown** | **Circuit clots** | **DVT** | **Cerebral infarction** | **pulmonary embolism** | **artery thrombosis** | **limb ischemia** | **stroke** |
| --- | --- | --- | --- | --- | --- | --- | --- | --- | --- | --- | --- | --- | --- | --- | --- | --- | --- | --- | --- | --- | --- | --- | --- | --- | --- |
| 7 | Austin | 2024 | Survivors | 26 | Bleeding 10, clotting 9 | Bleeding 10, clotting 9, Neurologic decline 4 | 10 | 10 |  |  |  |  |  |  |  |  | 9 | 9 |  |  |  |  |  |  |  |
| 7 | Austin | 2024 | Nonsurvivors | 10 | Bleeding 7, clotting 1 | Bleeding 7, clotting 1, Neurologic decline 1 | 7 | 7 |  |  |  |  |  |  |  |  | 1 | 1 |  |  |  |  |  |  |  |
| 14 | Mehran Dadras | 2019 | NA | 8 | thoracic hemorrhage 1 | thoracic hemorrhage(1)  acute kidney failure (4) | 1 |  | 1 |  |  |  |  |  |  |  | 0 |  |  |  |  |  |  |  |  |
| 30 | Alex Lee | 2023 | NA | 25 | Circuit clots requiring exchange(3), Circuit clot-failure of system(1). | cannula related vascular injuries (4), Circuit clots requiring exchange(3), Circuit clot-failure of system(1). | 0 |  |  |  |  |  |  |  |  |  | 4 |  | 4 |  |  |  |  |  |  |
| 36 | Steven Neubauer | 2023 | NA | 5 | Hemorrhage (2) | Hemorrhage (2), pneumothorax (1), asystolic event (1), and stroke (1). | 2 | 2 |  |  |  |  |  |  |  |  | 0 |  |  |  |  |  |  |  | 1 |
| 38 | Michael J. Perchinsky, | 1995 | NA | 6 | surgical bleeding and coagulopathies 6 | surgical bleeding and coagulopathies 6 | 0 |  |  |  |  |  |  |  | 6 |  | 0 |  |  |  |  |  |  |  |  |
| 51 | Liu | 2023 | NA | 13 | cerebral hemorrhage （1） | cerebral hemorrhage 1, kidney failure 1, bloodstream infection 2, Septic shock 2, lung infection 5 | 1 |  |  | 1 |  |  |  |  |  |  | 0 |  |  |  |  |  |  |  |  |
| 52 | Fei | 2023 | NA | 8 | limb intermuscular vein thrombosis 3， peritoneal vein thrombosis 2，femoral artery thrombosis 1 | limb intermuscular vein thrombosis 3， peritoneal vein thrombosis 2，femoral artery thrombosis 1 | 0 |  |  |  |  |  |  |  |  |  | 6 |  |  | 5 |  |  | 1 |  |  |
| 53 | Jiang | 2005 | ECMO | 12 | 0 | NA | 0 |  |  |  |  |  |  |  |  |  | 0 |  |  |  |  |  |  |  |  |
| 55 | Xie | 2005 | NA | 17 | 0 | NA | 0 |  |  |  |  |  |  |  |  |  | 0 |  |  |  |  |  |  |  |  |
| 61 | Anderson | 1994 | survivors | 15 | Bleeding 9, Cerebral infarct/hemorrhage 3 | Bleeding 9, Renal failure 2, Seizure 1, Cerebral infarct/hemorrhage 3, Pneumothorax 2, Oxygenator failure 1, ECLS circuit change 5 | 9 | 9 |  |  |  |  |  |  |  |  | 6 | 6 |  |  |  |  |  |  | 3 |
| 61 | Anderson | 1994 | Nonsurvivors | 9 | Bleeding 9, Cerebral infarct/hemorrhage 2 | Bleeding 9, Renal failure 3, Arrhythmiacardiac arrest 3, Cerebral infarct/hemorrhage 2, Oxygenator failure 1, Raceway/tubing rupture 2, Pump failure 1, ECLS circuit change 1 | 9 | 9 |  |  |  |  |  |  |  |  | 3 | 3 |  |  |  |  |  |  | 2 |
| 62 | Senunas | 1997 | NA | 14 | Bleeding 7 | Bleeding 7, Cardiac arrest 1, Seizure 2, pneumothorax 1, acute renal failure 4, ECLS circuit change required 2 | 7 |  |  |  |  |  |  |  |  |  | 2 | 2 |  |  |  |  |  |  |  |
| 67 | Bein | 2012 | VV-ECMO | 5 | heparin-induced thrombocytopenia(1) | heparin-induced thrombocytopenia（1） | 0 |  |  |  |  |  |  |  |  |  | 0 |  |  |  |  |  |  |  |  |
| 69 | Bonacchi | 2012 | ECLS success | 14 | oxygenator failure due to clot formation 1 | leg ischemia due to femoral artery cannulation 1  oxygenator failure due to clot formation 1 | 0 |  |  |  |  |  |  |  |  |  | 1 |  | 1 |  |  |  |  | 1 |  |
| 70 | Ried | 2013 | VV-ECMO | 26 | NA | Cannula-related complications (3) | 0 |  |  |  |  |  |  |  |  |  | 0 |  |  |  |  |  |  |  |  |
| 71 | Guirand | 2014 | VV-ECMO | 17 | Hemorrhagic 3; ECLS patients received more blood transfusions and had more bleeding complications | Hemorrhagic 3;AKI 16 | 3 | 3 |  |  |  |  |  |  |  |  | 0 |  |  |  |  |  |  |  |  |
| 72 | Tseng | 2014 | NA | 9 | uncontrolled retroperitoneal hemorrhage (1) 、hemothorax (1). | uncontrolled retroperitoneal hemorrhage（1） and hemothorax （1）； | 2 |  | 1 |  | 1 |  |  |  |  |  | 0 |  |  |  |  |  |  |  |  |
| 73 | Wu | 2014 | Patient with hemorrhagic complication | 7 | 7 | NA | 7 | 7 |  |  |  |  |  |  |  |  | 0 |  |  |  |  |  |  |  |  |
| 73 | Wu | 2014 | Patient without hemorrhage complications | 13 | heparin-induced thrombocytopenia（1） | NA | 0 |  |  |  |  |  |  |  |  |  | 0 |  |  |  |  |  |  |  |  |
| 74 | Bosarge | 2016 | ECMO | 15 | 6 had hemorrhagic complications, including persistent epistaxis requiring nasal packing, gastrointestinal bleeding, surgical incision or cannula insertion site bleeding, and expanding hematomas | Bleeding(6); RIJ thrombus右颈内静脉(3); SVC occlusion上腔静脉(1); RUE thrombus右上肢(1) | 6 | 1 |  |  | 1 | 1 | 1 | 1 | 1 |  | 4 |  |  | 4 |  |  |  |  |  |
| 82 | Lin | 2017 | Survivors | 22 | Bleeding 8, Cerebral infarction 2, ICH 1 | Bleeding 8, Cerebral infarction 2, ICH 1, Limb ischemia 2, Sepsis 5 | 9 | 8 |  | 1 |  |  |  |  |  |  | 2 |  |  |  | 2 |  |  | 2 |  |
| 82 | Lin | 2017 | Mortality | 21 | Bleeding 9, Cerebral infarction 4, ICH 2 | Bleeding 9, Cerebral infarction 4, ICH 2, Limb ischemia 4, Sepsis 12 | 11 | 9 |  | 2 |  |  |  |  |  |  | 4 |  |  |  | 4 |  |  | 4 |  |
| 85 | Grant | 2018 | post-Advanced ECMO Program | 12 | Thrombosis/ischemia 2, Bleeding event 5 | Renal failure requiring dialysis 6, Liver failure 4, Brain death 1 , Thrombosis/ischemia 2, Bleeding event 5 | 5 | 5 |  |  |  |  |  |  |  |  | 2 | 2 |  |  |  |  |  |  |  |
| 85 | Grant | 2018 | pre-Advanced ECMO Program | 7 | Thrombosis/ischemia 3, Bleeding event 3 | Renal failure requiring dialysis 5, Liver failure 0, Brain death 1 , Thrombosis/ischemia 3, Bleeding event 3 | 3 | 3 |  |  |  |  |  |  |  |  | 3 | 3 |  |  |  |  |  |  |  |
| 89 | Strumwasser | 2018 | NA | 7 | clotting of the ECMO circuit 3 | clotting of the ECMO circuit 3, hospital-acquired pneumonia 1 | 0 |  |  |  |  |  |  |  |  |  | 3 |  | 3 |  |  |  |  |  |  |
| 90 | Szentgyorgyi | 2018 | NA | 5 | right internal jugular vein thrombus 1; gastrointestinal bleeding 1; | AKI 4; right internal jugular vein thrombus 1;neutropenic sepsis 1;gastrointestinal bleeding 1; | 1 |  |  |  |  |  | 1 |  |  |  | 1 |  |  | 1 |  |  |  |  |  |
| 92 | Kruit | 2019 | Survivors+ Deaths | 52 | 总体：Bleeding 20(cerebral hemorrhage 3)，Thrombotic complications 16 | 总体：Bleeding 26, Thrombotic complications 21 (oxygenator failure (8), deep vein thrombosis (8), followed by pulmonary embolism (3)) | 20 | 17 |  | 3 |  |  |  |  |  |  | 16 | 5 |  | 8 |  | 3 |  |  |  |
| 93 | Lang | 2019 | Survivors+Nonsurvivors | 16 | Local bleeding at the puncture site 1, | 总体（survivors + non-survivors）：Local bleeding at the puncture site 1, Malpositioning of the Y-cannula 1, MOF 3, Diffuse intravascular coagulopathy 1, Hypoxic cerebral edema 1, Vascular injury 1 | 1 |  |  |  |  | 1 |  |  |  |  | 0 |  |  |  |  |  |  |  |  |
| 94 | Lee | 2020 | NA | 42 | Leg ischemia 1;Ulcer bleeding 1 | Leg ischemia 1;Bed sore 2 ;ARF (creatinine > 2 mg/dL) 11 ; Cholecystitis 2; Ulcer bleeding 1;CNS injury 1 ;Multiorgan failure 2 | 1 |  |  |  |  |  | 1 |  |  |  | 1 |  |  |  |  |  |  | 1 |  |
| 97 | Amos | 2021 | NA | 11 | Haemorrhage 4 | Haemorrhage 4, Venous thrombosis 3, Limb ischaemia 2, Ischaemic stroke 1, Circuit change due to thrombosis 2, Pump failure 1 | 4 | 4 |  |  |  |  |  |  |  |  | 6 |  | 2 | 3 |  |  |  | 1 | 1 |
| 99 | Parker | 2021 | NA | 13 | Circuit thrombosis(4),bleeding from a thoracic excision （1）epistaxis（1） | Circuit thrombosis(4),bleeding from a thoracic excision （1）epistaxis（1） | 2 |  |  |  |  |  |  | 1 | 1 |  | 4 |  | 4 |  |  |  |  |  |  |
| 105 | Lee | 2022 | VV-ECMO | 16 | NA | 0 | 0 |  |  |  |  |  |  |  |  |  | 0 |  |  |  |  |  |  |  |  |
| 106 | Ismael A Salas De Armas | 2022 | NA | 15 | DVT 2 falcine, subdural hemorrhage and diffused cerebral edema 1 frontal infarct with hemorrhagic conversion and midline shift 1 | DVT 2 falcine, subdural hemorrhage and diffused cerebral edema 1 frontal infarct with hemorrhagic conversion and midline shift 1 | 2 |  |  | 2 |  |  |  |  |  |  | 2 |  |  | 2 |  |  |  |  |  |
| 107 | Jaimin R. TRivedi | 2022 | NA | 7 | bleeding or ECMO circuit issues 3 | pulmonary infections 4  shock liver 1 acute renal failure 4 hypofibrinogenemia 1 thrombocytopenia 1 continued blood loss 1 hip disarticulation 1 limb ischemia 1  rhabdomyolysis 1 pulmonary embolus 1 stroke 1 anoxic brain injury 1 | 3 | 3 |  |  |  |  |  |  |  |  | 2 |  |  |  |  | 1 |  | 1 | 1 |
| 108 | Weidemann | 2022 | NA | 19 | massive bleeding from surgical wounds 1 | massive bleeding from surgical wounds 1  another patient developed right heart failure 1 | 1 |  |  |  |  |  |  |  |  | 1 | 0 |  |  |  |  |  |  |  |  |
| 111(单组拆分) | Seon Hee Kim | 2023 | NA | 21 | 0 | NA | 0 |  |  |  |  |  |  |  |  |  | 0 |  |  |  |  |  |  |  |  |
| new1 | Henry | 2021 | ecmo | 97 | Deep Vein Thrombosis 17 Pulmonary Embolus 16 | Acute Kidney Injury 28 Myocardial Infarction 3 Stroke 4 Deep Vein Thrombosis 17 Pulmonary Embolus 16 Central Line Infection 1 Ventilator Associated Pneumonia 7 Sepsis 17 Superficial SSI 4 Deep SSI 1 Compartment syndrome 2 | 0 |  |  |  |  |  |  |  |  |  | 33 |  |  | 17 |  | 16 |  |  | 4 |
| new3 | Menaker | 2018 | NA | 18 | Bleeding 6, DVT 10 | Bleeding 6, DVT 10 | 6 | 6 |  |  |  |  |  |  |  |  | 10 |  |  | 10 |  |  |  |  |  |
| total | 34 articles |  |  | 675 |  |  | 133 | 103 | 2 | 9 | 2 | 2 | 3 | 2 | 8 | 1 | 125 | 31 | 14 | 50 | 6 | 20 | 1 | 10 | 12 |
|  |  |  |  |  |  |  | 19.70% |  |  |  |  |  |  |  |  |  | 18.50% |  |  |  |  |  |  |  |  |

**Table S10. Domain 6** **Complications.**

**Table S10.** **Characteristics of studies on ECMO related complications**

| **Group** | **author** | **Year** | **Sample size** | **Hemorrhage -1** | **Thromboembolism/Ischemia -1** | **Infection-1** | **AKI-1** | **Hemorrhage-2** | **Thromboembolism/**  **Ischemia-2** | **Infection-2** | **AKI-2** | **ECMO related complications** |
| --- | --- | --- | --- | --- | --- | --- | --- | --- | --- | --- | --- | --- |
| ECMO | Nosanov | 2017 | 30 | 0 | 0 | Pneumonia 9, Sepsis 6 | Renal failure 6 | 0 | 0 | 15 | 6 | Pneumonia 9, Renal failure 6, Sepsis 6 |
| nonECMO | Nosanov | 2017 | 30 | 0 | 0 | 0 | 0 | 0 | 0 | 0 | 0 | Pneumonia 0, Renal failure 0, Sepsis 0 |
| nonECMO | Henry | 2021 | 1266 | 0 | MI 32, Stroke 51, DVT 139, PE 51 | CLI 35, Sepsis 203, SSI 36 | AKI 241 | 0 | 273 | 274 | 241 | Acute Kidney Injury 241, Myocardial Infarction 32, Stroke 51, Deep Vein Thrombosis 139, Pulmonary Embolus 51, Central Line Infection 35, Ventilator Associated Pneumonia 0, Sepsis 203, Superficial SSI 13, Deep SSI 23, Compartment syndrome 13 |
| ECMO | Henry | 2021 | 97 | 0 | MI 3, Stroke 4, DVT 17, PE 16 | CLI 1, VAP 7, Sepsis 17, SSI 5 | AKI 28 | 0 | 40 | 30 | 28 | Acute Kidney Injury 28, Myocardial Infarction 3, Stroke 4, Deep Vein Thrombosis 17, Pulmonary Embolus 16, Central Line Infection 1, Ventilator Associated Pneumonia 7, Sepsis 17, Superficial SSI 4, Deep SSI 1, Compartment syndrome 2 |
| ECMO | Guirand | 2014 | 17 | 3 | 0 | 0 | AKI 16 | 3 | 0 | 0 | 16 | Hemorrhagic 3, AKI 16 |
| nonECMO | Guirand | 2014 | 17 | 0 | 0 | Pneumonia 3 | AKI 16 | 0 | 0 | 3 | 16 | Pneumonia 3, AKI 16 |

**Table S11.** Characteristics of studies on ECMO-related complications in adult trauma patients with or without TBI.

| **Author,year** | Study design | Grouping | Group | Sample size | ECMO mode (n) | TBI cases | TBI survival (n) | **ECMO related complications** | **TBI ECMO-related complications** | **non-TBI ECMO-related complications** |
| --- | --- | --- | --- | --- | --- | --- | --- | --- | --- | --- |
| Steven Neubauer，2023 | case series | Single arm study | NA | 5 | VV-ECMO 5 | 3 | 3 | Hemorrhage (2), pneumothorax (1), asystolic event (1), and stroke (1). | Hemorrhage (1), pneumothorax (1), asystolic event (1), and stroke (1). | Hemorrhage (1) |
| Bein，2012 | case series | PECLA VS VV ECMO | VV-ECMO | 5 | VV ECMO 5 | 1 | 1 | heparin-induced thrombocytopenia（1） | 0 | heparin-induced thrombocytopenia（1） |
| Tseng，2014 | case series | single arm study | NA | 9 | VA-ECLS (9) | 1 | 0 | uncontrolled retroperitoneal hemorrhage（1） and hemothorax （1）； | 0 | uncontrolled retroperitoneal hemorrhage（1） and hemothorax （1）； |
| Bosarge，2016 | cohort study | ECMO VS CONV | ECMO | 15 | Venovenous (V/V) in 10 patients, venoarterial (V/A) in 3, venoarterial venous (V/A/V) in 2 | 2 | 2 | Bleeding (6); RIJ thrombus (3); SVC occlusion (1); RUE thrombus (1) | Bleeding (1); RUE thrombus (1) | Bleeding (5); RIJ thrombus (3); SVC occlusion (1) |
| Huh，2017 | case series | Single arm study | NA | 5 | VA-ECMO (5) | 0 | 0 | Cerebral infarction 2 | 0 | Cerebral infarction (2) |
| Amos，2021 | cohort study | Early initiation of ECMO (< 72 hours) VS Late (> 72 hours) | NA | 11 | VV ECMO 7, VA ECMO 3, VV ECMO to VAV ECMO to VV ECMO 1 | 7 | NA | Haemorrhage（4）, Venous thrombosis（3）, Limb ischaemia（2）, Ischaemic stroke（1）, Circuit change due to thrombosis（2）, Pump failure（1） | Uncontrollable haemorrhage(pre-existing)（1）、Circuit change due to thrombosis （1）、Ischaemic stroke （1）、Venous thrombosis （2）, Limb ischaemia （1） | Hemorrhage （3）, Venous thrombosis （1）, Limb ischaemia （1）, Circuit change due to thrombosis （1）, Pump failure (1) |
| Parker，2021 | case series | Survivors vs Nonsurvivors | NA | 13 | VV-ECMO (13) | 13 | 5 | Circuit thrombosis(4),bleeding from a thoracic excision （1）epistaxis（1） | Circuit thrombosis(4),bleeding from a thoracic excision （1）epistaxis（1） | 0 |
| Ismael A Salas De Armas，2022 | case series | single arm study | NA | 15 | VV-ECMO （15） | 5 | NA | DVT 2 falcine, subdural hemorrhage and diffused cerebral edema 1 frontal infarct with hemorrhagic conversion and midline shift 1 | falcine, subdural hemorrhage and diffused cerebral edema (1) | frontal infarct with hemorrhagic conversion and midline shift (1) |
| Total | NA | NA | NA | **78** | VV-ECMO（55） VA-ECMO（20） VAV-ECMO（2） VV-ECMO to VAV-ECMO to VV-ECMO（1） | **32** | NA | Hemorrhage 16、thrombosis 13、stroke 6、limb ischaemia 2、DVT 2、pneumothorax 1、asystolic event 1、superior vena cava occlusion 1、Pump failure 1、thrombocytopenia 1 | Hemorrhage 5（15.6%）、thrombosis 8（25.0%）、stroke 3(9.4%)、limb ischaemia 1、pneumothorax 1、asystolic event 1 | Hemorrhage 11(23.9%)、thrombosis 5(10.9%)、stroke 3(6.5%)、limb ischaemia 1、superior vena cava occlusion 1、Pump failure 1、thrombocytopenia 1 |

**Table S12. Evidence to Decision Framework**

**Domain 1. Indications**

| **Question 1:** | **For adult trauma patients with acute cardiopulmonary failure refractory to conventional therapy, should ECMO be used to improve survival?** |
| --- | --- |
| Population | Adult trauma patients with acute cardiopulmonary failure refractory to conventional therapy |
| Intervention | ECMO |
| Comparator | Conventional therapy |
| Main outcomes | Critical: survival rate |
| Background | The benefits and harms of ECMO to trauma patients with refractory cardiopulmonary failure due to varied injury mechanisms remain elusive, including polytrauma, TBI, moderate to severe burn with inhalation injury, and traumatic cardiac arrest. |
| Conflict of interests | None |

**Assessment**

| **Problem**  Is the problem a priority? | | |
| --- | --- | --- |
| **Judgement** | **Research evidence** | **Additional considerations** |
| ○ No  ○ Probably no  ○ Probably yes  ● Yes  ○ Varies  ○ Don't know | Among the residents in China, road traffic injuries and high falls ranked the 6th and 18th among all causes of death, causing 19 and 10 deaths per 100,000 population each year, respectively, which are higher than the average of 15.8 and 9.2 deaths per 100,000 population across 195 countries and territories. Therefore, improving trauma care has become a priority for the government when formulating public health policies.  Through question formulating and outcome selecting and Delphi expert voting, more than 75% of the experts ranked the question and outcome as critical and agreed to be included in the consensus. |  |
| **Desirable Effects**  How substantial are the desirable anticipated effects? | | |
| **Judgement** | **Research evidence** | **Additional considerations** |
| ○ Trivial  ○ Small  ○ Moderate  ● Large  ○ Varies  ○ Don't know | One-arm meta-analysis by the Evidence Group shows that traumatic ARDS was the most documented indication for VV-ECMO in adult trauma patients with varied injury mechanisms, with a pooled overall survival of 68% (95% CI: 64%–72%; 549 patients, 28 studies), whereas that of those unexposed to ECMO was 49% (95% CI: 35%–62%; 1460 patients, six studies). Traumatic cardiac arrest (TCA) and cardiogenic shock were the most frequent conditions for VA-ECMO, the pooled overall survival rates were 42% (95% CI: 30%–54%) and 55% (95% CI: 26%–81%), respectively (18 retrospective studies, 159 patients). In settings where ECMO was unavailable, the overall hospital mortality of TCA patients was 73%, and only 7% survived until hospital discharge.  When compared with those unexposed to ECMO, the *OR* for survival was 2.38 (95% CI: 0.56–10.1, *P* = 0.24; six studies, 1633 patients) in ECMO-supported adult polytraumatized patients, and 1.41 (95% CI: 0.77–2.58, *P* = 0.26; 12 studies, 216 patients) in the ECMO-supported adult TBI patients. The pooled overall survival rate was 49% (95% CI: 29%–70%) in the ECMO-supported patients with burn and inhalation injury (nine studies, 103 patients). |  |
| **Undesirable Effects**  How substantial are the undesirable anticipated effects? | | |
| **Judgement** | **Research evidence** | **Additional considerations** |
| ○ Trivial  ● Small  ○ Moderate  ○ Large  ○ Varies  ○ Don't know | Retrospective studies show that the prevalence of ECMO-related complications seems to be higher in ECMO-supported trauma patients. However, the rates of clinically important adverse events, e.g., massive intracranial hemorrhage and neurological disability, are comparable to those of trauma patients without ECMO.  No data are available on the impact of traumatic ECMO on survivors' long-term quality of life and organ disability. |  |
| **Certainty of evidence**  **What is the overall certainty of the evidence of effects?** | | |
| **Judgement** | **Research evidence** | **Additional considerations** |
| ○ Very low  ● Low  ○ Moderate  ○ High  ○ No included studies | The certainty of evidence is very low due to the non-randomized design, serious inconsistency (in population, intervention and outcome), imprecision (wide OR 95% CI), and heterogeneities across the studies (*I*^2^=0%–70%, τ^2^=0.3–0.9, all *P* < 0.01). The optimal information size criterion is met, but the 95% CI overlaps no effect and fails to exclude important benefit. Nonetheless, the survival benefit is large in most of included studies, and the panel has high confidence that the further research is unlikely to change the estimate of the effect. |  |
| **Values and Preferences**  Is there important uncertainty about or variability in how much people value the main outcomes? | | |
| **Judgement** | **Research evidence** | **Additional considerations** |
| ○ Important uncertainty or variability  ○ Possibly important uncertainty or variability  ● Probably no important uncertainty or variability  ○ No important uncertainty or variability | Under the condition that the indications and the initiation criteria are met, and the injury is reversible, the patient representative, stakeholders and experts are unanimously inclined to accept ECMO intervention. |  |
| **Balance of effects**  **Does the balance between desirable and undesirable effects favor the intervention or the comparison?** | | |
| **Judgement** | **Research evidence** | **Additional considerations** |
| ○ Favors the comparison  ○ Probably favors the comparison  ○ Does not favor either the intervention or the comparison  ○ Probably favors the intervention  ● Favors the intervention  ○ Varies  ○ Don't know | Most of included studies showed that trauma ECMO rendered a substantial survival benefit to the severely injured patients who are refractory to conventional therapy and at high-risk of death, with comparable survival rates to those unexposed to ECMO. The undesirable effects are small. The rates of clinically important ECMO-related adverse events, e.g., massive intracranial hemorrhage and neurological disability, are comparable to those of trauma patients without ECMO.  The consensus expert panel have moderate to high confidence that ECMO could improve the prognosis of patients with severe trauma, and future researches are unlikely to change this estimate of the effect. It is strongly recommended for clinical use. |  |
| **Cost effectiveness**  Does the cost-effectiveness of the intervention favor the intervention or the comparison? | | |
| **Judgement** | **Research evidence** | **Additional considerations** |
| ○ Favors the comparison  ○ Probably favors the comparison  ○ Does not favor either the intervention or the comparison  ○ Probably favors the intervention  ● Favors the intervention  ○ Varies  ○ No included studies | There is a lack of cost-effect analysis. Though ECMO treatment is resource-consuming, but it improves the survival of severely injured patients with refractory cardiopulmonary failure, and the benefits outweigh the costs. |  |
| **Equity**  What would be the impact on health equity? | | |
| **Judgement** | **Research evidence** | **Additional considerations** |
| ○ Reduced  ○ Probably reduced  ○ Probably no impact  ○ Probably increased  ○ Increased  ● Varies  ○ Don't know | Lack of data for quantitative analysis. Though ECMO treatment may lead to resource occupation and in resource allocation inequality, but it may promote the right to life and health of trauma patients. |  |
| **Acceptability**  Is the intervention acceptable to key stakeholders? | | |
| **Judgement** | **Research evidence** | **Additional considerations** |
| ○ No  ○ Probably no  ● Probably yes  ○ Yes  ○ Varies  ○ Don't know | There is a lack of extensive patient survey and analysis. The patient representative, stakeholders and experts are unanimously inclined to accept ECMO use for trauma patients with refractory cardiopulmonary failure, if the criteria of ethics, laws and policies are met. |  |
| **Feasibility**  Is the intervention feasible to implement? | | |
| **Judgement** | **Research evidence** | **Additional considerations** |
| ○ No  ○ Probably no  ○ Probably yes  ● Yes  ○ Varies  ○ Don't know | Retrospective analysis shows that with the advances in ECMO technology, its use in trauma has increased exponentially in recent years. There are more than 800 registered ECMO centers in China, which are distributed in tertiary hospitals or level I trauma centers with trauma ECMO capacity, making it feasible to implement trauma ECMO. | The reversibility of injury, the effectiveness of decisive treatment and the long-term outcomes of patients should be prudently considered. |

**TYPE OF RECOMMENDATION**

| Strong recommendation against the intervention  **○** | Conditional recommendation against the intervention  **○** | Conditional recommendation for either the intervention or the comparison  ○ | Weak recommendation on the intervention for patients with burn or traumatic cardiac arrest  ● | Strong recommendation on the intervention for patients with poly-trauma and TBI  ● |
| --- | --- | --- | --- | --- |

**Conclusion**

| Recommendation |
| --- |
| **Recommendation 1.** We recommend that VV-ECMO be used for adult patients with polytrauma complicated with ARDS, if conventional lung protective ventilation is ineffective (strong recommendation, very low-quality evidence).  **Recommendation 2.** We recommend that VV-ECMO be used for adult TBI patients with ARDS, if conventional lung protective ventilation is ineffective (strong recommendation, very low-quality evidence).  R**ecommendation 3.** We suggest that VV-ECMO be considered for adult trauma patients with ARDS caused by moderate to severe burn and inhalation injury, if optimal fluid resuscitation and lung protective ventilation are ineffective (weak recommendation, very low-quality evidence).  **Recommendation 4.** We suggest that VA-ECMO be considered for selected adult trauma patients with cardiac arrest or cardiogenic shock, if conventional resuscitation is ineffective (weak recommendation, very low-quality evidence). |
| Justification |
| Our meta-analyses showed that VV-ECMO conferred survival benefits to adult trauma patients with refractory ARDS due to varied injury mechanisms, including polytrauma, TBI, and moderate to severe burn with inhalation injury, with comparable survival rates to those unexposed to ECMO. VV-ECMO facilitates damage control surgery in polytraumatized patients, allows both neurological and lung protective ventilation among TBI patients, and solves the dilemma of aggressive fluid resuscitation while maintaining a “dry lung” in burn patients, thereby gaining time to damage control and bridging the patients to recovery. VA-ECMO increased the survival of patients following traumatic CPR or new-onset cardiogenic shock by 40%. |
| Implementation considerations |
| In deciding to implement trauma ECMO, clinicians should fully consider the reversibility of injury, efficacy of decisive therapy, patient’s preference, and long-term outcomes, not just based on the underlying injury mechanisms. For the TCA patients, it is imperative to closely monitor the patients’ hemodynamics, and once the hemodynamics is unstable, timely initiation of VA-ECMO could improve the likelihood of survival. The contraindications to trauma ECMO include poor neurological prognosis, irreversible injury, and uncontrollable bleeding with profound coagulopathy. |

**References**

1. Zhou M, Wang H, Zeng X, Yin P, Zhu J, Chen W, et al. Mortality, morbidity, and risk factors in China and its provinces, 1990-2017: a systematic analysis for the Global Burden of Disease Study 2017. lancet. 2019, 394 (10204): 1145-1158. doi: 10.1016/ S0140-6736(19)30427-1.
2. GBD 2017 Causes of Death Collaborators. Global, regional, and national age-sex-specific mortality for 282 causes of death in 195 countries and territories, 1980-2017: a systematic analysis for the Global Burden of Disease Study 2017. lancet. 2018, 392 (10159): 1736-1788. doi: 10.1016/ S0140-6736(18)32203-7.
3. Dadras M, Wagner JM, Wallner C, Huber J, Buchwald D, Strauch J, et al. Extracorporeal membrane oxygenation for acute respiratory distress syndrome in burn patients: a case series and literature update. Burns Trauma. 2019, 7:28.
4. Fouché TW, Vrouwe SQ, Gottlieb LJ, Song TH, Mehta S, Tung A, Estimé SR. Extracorporeal membrane oxygenation utilization in burn patients with severe acute respiratory distress syndrome. Burns. 2023, 49(1):244-246.
5. Hsu PS, Tsai YT, Lin CY, Chen SG, Dai NT, Chen CJ, et al. Benefit of extracorporeal membrane oxygenation in major burns after stun grenade explosion: Experience from a single military medical center. Burns. 2017, 43(3):674-680.
6. Huang YK, Liu KS, Lu MS, Wu MY, Tsai FC, Lin PJ. Extracorporeal life support in post-traumatic respiratory distress patients. Resuscitation. 2009, 80(5):535-539.
7. Marcus JE, Piper LC, Ainsworth CR, Sams VG, Batchinsky A, Okulicz JF, Barsoumian AE. Infections in patients with burn injuries receiving extracorporeal membrane oxygenation. Burns. 2019, 45(8):1880-1887.
8. Neubauer S, DelloStritto DJ, Capal N, Hotrum A, Henn L, Marchand T. Venovenous extracorporeal membrane oxygenation experience in a community level I trauma center. Perfusion. 2023, 38(3):484-490.
9. Soussi S, Gallais P, Kachatryan L, Benyamina M, Ferry A, Cupaciu A, et al. PRONOBURN Group. Extracorporeal membrane oxygenation in burn patients with refractory acute respiratory distress syndrome leads to 28 % 90-day survival. Intensive Care Med. 2016, 42(11):1826-1827.
10. Liu Ying, Yuan Xiang, Qu Yuran, Li Yajie, Li Xiaohui, Yu Haibin, et al. Clinical observation of extracorporeal membrane oxygenation in severe acute respiratory distress syndrome after severe trauma. Chinese Journal of Experimental Surgery. 2023, 40(8): 1504-1507. [In Chinese]. Available at: <https://rs.yiigle.com/cmaid/1473934>. (Accessed Dec 15, 2024).
11. Fei Danting, Li Wei, Chen Weiping, Shen Yunzhong, Xu Jun, Suo Yuan, et al. Clinical analysis and literature review of 8 cases with severe trauma treated by extracorporeal membrane oxygenation. Chinese Journal of Integrated Traditional and Western Medicine in Intensive and Critical Care. 2023, 30(4): 464-467. [In Chinese]. Available at: http://www.cccm-em120.com/ zhongxiyiguokan/30/464.pdf. (Accessed Dec 15, 2024).
12. Jiang Guoping, Jiang Guanyu, Ge Genxian, Xu Shiwei. Effect of emergency bedside extracorporeal membrane lung therapy on ARDS post severe trauma. 2005. Conference paper of the sixth national conference on critical illness. [In Chinese]. Available at: <https://www.doc88.com/p-9502334529043.html>. (Accessed Dec 15, 2024).
13. Li HS, Yuan ZQ, Song HP, et al. Clinical application of extracorporeal membrane oxygenation in the treatment of burn patients with acute respiratory distress syndrome: a retrospective analysis and systematic review. Chin J Burns, 2021, 37(10): 911-920.
14. Xie gang, Jiang Chonghui, Li Binfei, et a1. Extracorporeal membrane oxygenation in severe pulmonary contusion. Clinical Medicine of China. 2005, 21(09): 817-819. [In Chinese]. Available at: <https://rs.yiigle.com/CN2021/400096.htm>. (Accessed Dec 15, 2024).
15. Bosarge PL, Raff LA, McGwin G Jr, Carroll SL, Bellot SC, Diaz-Guzman E, Kerby JD. Early initiation of extracorporeal membrane oxygenation improves survival in adult trauma patients with severe adult respiratory distress syndrome. J Trauma Acute Care Surg. 2016, 81(2):236-43.
16. Chen TH, Shih JY, Shih JJ. Early Percutaneous Heparin-Free Veno-Venous Extra Corporeal Life Support (ECLS) is a Safe and Effective Means of Salvaging Hypoxemic Patients with Complicated Chest Trauma. Acta Cardiol Sin. 2016, 32(1):96-102.
17. Kim HS, Ha SO, Han SJ, Kim HS, Lee SH, Jung KS, Park S. Extracorporeal Membrane Oxygenation Support in Trauma Versus Nontrauma Patients with Noninfectious Acute Respiratory Failure. Artif Organs. 2017, 41(5):431-439.
18. Grant AA, Hart VJ, Lineen EB, Lai C, Ginzburg E, Houghton D, et al. The Impact of an Advanced ECMO Program on Traumatically Injured Patients. Artif Organs. 2018, 42(11):1043-1051.
19. Ainsworth CR, Dellavolpe J, Chung KK, Cancio LC, Mason P. Revisiting extracorporeal membrane oxygenation for ARDS in burns: A case series and review of the literature. Burns. 2018, 44(6):1433-1438.
20. Strumwasser A, Tobin JM, Henry R, Guidry C, Park C, Inaba K, Demetriades D. Extracorporeal membrane oxygenation in trauma: A single institution experience and review of the literature. Int J Artif Organs. 2018, 41(12): 845-853.
21. Szentgyorgyi L, Shepherd C, Dunn KW, Fawcett P, Barker JM, Exton P, et al. Extracorporeal membrane oxygenation in severe respiratory failure resulting from burns and smoke inhalation injury. Burns. 2018, 44(5):1091-1099.
22. Al-Thani H, Al-Hassani A, El-Menyar A, Asim M, Fawzy I. Outcome of post-traumatic acute respiratory distress syndrome in young patients requiring extracorporeal membrane oxygenation (ECMO). Sci Rep. 2022, 12(1):10609.
23. Brewer JM, Tran A, Yu J, Ali MI, Poulos CM, Gates J, Underhill D, Gluck J. Application and outcomes of extracorporeal life support in emergency general surgery and trauma. Perfusion. 2022, 37(6):575-581.
24. Eisenga J, Monday K, Blough B, Vandervest K, Lingle K, Espinoza O, Schwartz G. Extracorporeal membrane oxygenation support in the setting of penetrating traumatic injuries. J Card Surg. 2022, 37(12):4359-4361.
25. Lee GJ, Kim MJ, Lee JG, Lee SH. Use of venovenous extracorporeal membrane oxygenation in trauma patients with severe adult respiratory distress syndrome: A retrospective study. Int J Artif Organs. 2022, 45(10):833-840.
26. Trivedi JR, Alotaibi A, Sweeney JC, Fox MP, van Berkel V, Adkins K, et al. Use of Extracorporeal Membrane Oxygenation in Blunt Traumatic Injury Patients with Acute Respiratory Distress Syndrome. ASAIO J. 2022, 68(4): e60-e61.
27. Powell EK, Reynolds TS, Webb JK, Kundi R, Cantu J, Keville M, et al. Early veno-venous extracorporeal membrane oxygenation is an effective strategy for traumatically injured patients presenting with refractory respiratory failure. J Trauma Acute Care Surg. 2023, 95(2S Suppl 1): S50-S59.
28. Kim SH, Huh U, Song S, Kim MS, Wang IJ, Tak YJ. Outcomes in trauma patients undergoing veno-venous extracorporeal membrane oxygenation for acute respiratory distress syndrome. Perfusion. 2023, 38(5):1037-1044.
29. Henry R, Ghafil C, Piccinini A, Liasidis PK, Matsushima K, Golden A, et al. Extracorporeal support for trauma: A trauma quality improvement project (TQIP) analysis in patients with acute respiratory distress syndrome. Am J Emerg Med. 2021, 48:170-176.
30. Al-Thani H, Al-Hassani A, El-Menyar A, Asim M, Fawzy I. Outcome of post-traumatic acute respiratory distress syndrome in young patients requiring extracorporeal membrane oxygenation (ECMO). Sci Rep. 2022, 12(1):10609.
31. Austin SE, Galvagno SM, Podell JE, Teeter WA, Kundi R, Haase DJ, et al. Venovenous extracorporeal membrane oxygenation in patients with traumatic brain injuries and severe respiratory failure: A single-center retrospective analysis. J Trauma Acute Care Surg. 2024, 96(2): 332-339.
32. Tecos Maria, Buesing Keely, Waibel Brett, Kemp Kevin, Scriven Nicole, Evans Charity, et al. 1309: EXTRACORPOREAL MEMBRANE OXYGENATION FOR TRAUMA PATIENTS: A SINGLE-CENTER 5-YEAR RETROSPECTIVE REVIEW. Critical Care Medicine, 2023, 51(1): 654.
33. Chen Jianming, Zhong Jing, Song Zhiming, et al. Efficacy comparison of extracorporeal membrane oxygenation and ventilation therapy in the treatment of severe blast lung injury. Chinese Journal of Trauma. 2022, 38(11): 992-998. DOI: 10.3760/cma.j.cn501098-20220427-00323. [In Chinese]. Available at: <https://rs.yiigle.com/cmaid/1435374>. (Accessed Dec 15, 2024).
34. Bein T, Zonies D, Philipp A, Zimmermann M, Osborn EC, Allan PF, et al. Transportable extracorporeal lung support for rescue of severe respiratory failure in combat casualties. J Trauma Acute Care Surg. 2012, 73(6):1450-1456.
35. Bonacchi M, Spina R, Torracchi L, Harmelin G, Sani G, Peris A. Extracorporeal life support in patients with severe trauma: an advanced treatment strategy for refractory clinical settings. J Thorac Cardiovasc Surg. 2013, 145(6):1617-1626. 图片2012
36. Ried M, Bein T, Philipp A, Müller T, Graf B, Schmid C, Zonies D, Diez C, Hofmann HS. Extracorporeal lung support in trauma patients with severe chest injury and acute lung failure: a 10-year institutional experience. Crit Care. 2013, 17(3): R110.
37. Guirand DM, Okoye OT, Schmidt BS, Mansfield NJ, Aden JK, Martin RS, et al. Venovenous extracorporeal life support improves survival in adult trauma patients with acute hypoxemic respiratory failure: a multicenter retrospective cohort study. J Trauma Acute Care Surg. 2014, 76(5):1275-81.
38. Wu MY, Lin PJ, Tseng YH, Kao KC, Hsiao HL, Huang CC. Venovenous extracorporeal life support for posttraumatic respiratory distress syndrome in adults: the risk of major hemorrhages. Scand J Trauma Resusc Emerg Med. 2014, 22:56.
39. Amos T, Bannon-Murphy H, Yeung M, Gooi J, Marasco S, Udy A, Fitzgerald M. ECMO (extra corporeal membrane oxygenation) in major trauma: A 10-year single centre experience. Injury. 2021, 52(9):2515-2521.
40. Salas De Armas IA, Akkanti B, Doshi PB, Patel M, Kumar S, Akay MH, et al. Traumatic respiratory failure and veno-venous extracorporeal membrane oxygenation support. Perfusion. 2022, 37(5):477-483.
41. Trivedi JR, Alotaibi A, Sweeney JC, Fox MP, van Berkel V, Adkins K, et al. Use of Extracorporeal Membrane Oxygenation in Blunt Traumatic Injury Patients with Acute Respiratory Distress Syndrome. ASAIO J. 2022, 68(4): e60-e61.
42. Huang JE, Holland SR, Patrick J, Piper LC, Sams VG. Predictive survival factors of the traumatically injured on venovenous extracorporeal membrane oxygenation: A Bayesian model. J Trauma Acute Care Surg. 2020, 88(1):153-159.
43. Huh U, Song S, Chung SW, Kim SP, Lee CW, Ahn HY, et al. Is extracorporeal cardiopulmonary resuscitation practical in severe chest trauma? A systematic review in single center of developing country. J Trauma Acute Care Surg. 2017, 83(5):903-907.
44. Amos T, Bannon-Murphy H, Yeung M, Gooi J, Marasco S, Udy A, Fitzgerald M. ECMO (extra corporeal membrane oxygenation) in major trauma: A 10-year single centre experience. Injury. 2021, 52(9):2515-2521.
45. Lee A, Romano K, Tansley G, Al-Khaboori S, Thiara S, Garraway N, et al. Extracorporeal life support in trauma: Indications and techniques. J Trauma Acute Care Surg. 2024, 96(1):145-155.
46. Ull C, Schildhauer TA, Strauch JT, Swol J. Outcome measures of extracorporeal life support (ECLS) in trauma patients versus patients without trauma: a 7-year single-center retrospective cohort study. J Artif Organs. 2017, 20(2): 117-124.

**Domain 2. Patient Screening**

| **Question 2:** | **For adult trauma patients with acute cardiopulmonary failure refractory to conventional therapy, should the injury severity score be used as a screening tool for determining the ECMO candidates and treatment success?** |
| --- | --- |
| Population | Adult trauma patients with acute cardiopulmonary failure refractory to conventional therapy |
| Intervention | ISS screening tool |
| Comparator | Without screening |
| Main outcomes | Unimportant: Trauma-related mortality rate |
| Background | Although a variety of baseline variables were used to predict ECMO utilization and treatment success in severely injured patients, including age, sex, blood lactate, and oxygenation index, ISS might be independently associated with trauma ECMO use and treatment outcome. |
| Conflict of interests | None. |

**Assessment**

| **Problem**  Is the problem a priority? | | |
| --- | --- | --- |
| **Judgement** | **Research evidence** | **Additional considerations** |
| ○ No  ○ Probably no  ○ Probably yes  ● Yes  ○ Varies  ○ Don't know | More than 75% of the experts ranked the question and outcome measure as critical importance and agreed to be included in the consensus. |  |
| **Desirable Effects**  How substantial are the desirable anticipated effects? | | |
| **Judgement** | **Research evidence** | **Additional considerations** |
| ○ Trivial  ○ Small  ○ Moderate  ● Large  ○ Varies  ○ Don't know | ECMO-supported adult trauma patients had a higher ISS at admission than those unexposed to ECMO, with pooled ISS of 34.17 (95%CI 31.03 - 37.3; 35 studies, 1421 patients) and 26.77 (95%CI 24.14 - 29.41; 10 studies, 77,183 patients), respectively. Whereas The ECMO survivors had a relatively lower ISS than that of the non-survivors, the pooled ISSs were 30.36 (95% CI: 26.62–34.11) and 37.65 (95% CI: 31.87–43.43), with a SMD of 0.52 (95% CI: 0.19–0.84, *P* = 0.002; 14 studies, 989 patients).  Two retrospective studies reported that ISS was significantly related to ECMO utilization (*OR* 1.03, 95% CI: 1.01–1.04, *P* < 0.01), and mortality (*OR* 1.02, 95% CI: 1.01–1.03, *P* < 0.01). |  |
| **Undesirable Effects**  How substantial are the undesirable anticipated effects? | | |
| **Judgement** | **Research evidence** | **Additional considerations** |
| ● Trivial  ○ Small  ○ Moderate  ○ Large  ○ Varies  ○ Don't know | ISS scoring tool will not cause any harm to patients. |  |
| **Certainty of evidence**  **What is the overall certainty of the evidence of effects?** | | |
| **Judgement** | **Research evidence** | **Additional considerations** |
| ● Very low  ○ Low  ○ Moderate  ○ High  ○ No included studies | Very low, due to the non-randomized design, serious inconsistency, indirectness, imprecision, and heterogeneities across the studies (*I*^2^=60%–93%, τ^2^=0.18–0.22, all *P* < 0.01). The sample size is insufficient. The interventions used in the studies differed. |  |
| **Values and Preferences**  Is there important uncertainty about or variability in how much people value the main outcomes? | | |
| **Judgement** | **Research evidence** | **Additional considerations** |
| ○ Important uncertainty or variability  ○ Possibly important uncertainty or variability  ○ Probably no important uncertainty or variability  ● No important uncertainty or variability | The patient representative, stakeholders and experts are unanimously inclined to accept ECMO use for trauma patients with refractory cardiopulmonary failure. |  |
| **Balance of effects**  **Does the balance between desirable and undesirable effects favor the intervention or the comparison?** | | |
| **Judgement** | **Research evidence** | **Additional considerations** |
| ○ Favors the comparison  ○ Probably favors the comparison  ○ Does not favor either the intervention or the comparison  ○ Probably favors the intervention  ● Favors the intervention  ○ Varies  ○ Don't know | As a screening tool for ECMO candidate and treatment success, ISS is of non-invasion, simplicity, objectivity, and accessibility. No undesirable effects. |  |
| **Cost effectiveness**  Does the cost-effectiveness of the intervention favor the intervention or the comparison? | | |
| **Judgement** | **Research evidence** | **Additional considerations** |
| ○ Favors the comparison  ○ Probably favors the comparison  ○ Does not favor either the intervention or the comparison  ○ Probably favors the intervention  ● Favors the intervention  ○ Varies  ○ No included studies | Appropriate selection of patients who are most likely to benefit is conducive to reducing resource consumption and improving economic and social benefits. |  |
| **Equity**  What would be the impact on health equity? | | |
| **Judgement** | **Research evidence** | **Additional considerations** |
| ○ Reduced  ○ Probably reduced  ○ Probably no impact  ○ Probably increased  ● Increased  ○ Varies  ○ Don't know | Excluding patients with ineffective ECMO therapy may save medical resources and promote fair allocation of resources. |  |
| **Acceptability**  Is the intervention acceptable to key stakeholders? | | |
| **Judgement** | **Research evidence** | **Additional considerations** |
| ○ No  ○ Probably no  ○ Probably yes  ● Yes  ○ Varies  ○ Don't know | There is a lack of extensive patient survey and quantitative analysis. The patient representative, stakeholders and experts are unanimously inclined to accept ECMO use for trauma patients with refractory cardiopulmonary failure. |  |
| **Feasibility**  Is the intervention feasible to implement? | | |
| **Judgement** | **Research evidence** | **Additional considerations** |
| ○ No  ○ Probably no  ○ Probably yes  ● Yes  ○ Varies  ○ Don't know | SS scoring tool is of non-invasive, simple, objective, easy to use with training, easy to be accepted by patients, less resource consumption, and no additional equipment, which may promote medical equity and has high feasibility. |  |

**TYPE OF RECOMMENDATION**

| Strong recommendation against the intervention  ○ | Conditional recommendation against the intervention  ○ | Conditional recommendation for either the intervention or the comparison  ○ | Conditional recommendation for the intervention  ● | Strong recommendation for the intervention  ○ |
| --- | --- | --- | --- | --- |

**Conclusion**

| Recommendation |
| --- |
| **Recommendation 5.** We suggest using the injury severity score as an adjunctive screening tool for determining the ECMO candidate and treatment success in adult trauma patients with acute cardiopulmonary failure refractory to conventional therapy (weak recommendation, very low-quality evidence for both candidate and success). |
| Justification |
| Our meta-analyses demonstrated that ISS might be independently associated with trauma ECMO use and treatment success. A higher ISS of >30 points indicates that the patients have more serious and extensive injury and are more likely to develop refractory cardiopulmonary failure, whereas a very high ISS score denotes a poor chance of survival despite ECMO use. |
| Implementation considerations |
| In the case where the ISS exceeds an arbitrary threshold of 30 points, the risks of failure, cost-effectiveness, and the patients’ value and preference should be prudently considered when deciding to cannulate the patient or not. |

**References**

1. Mader MM, Lefering R, Westphal M, Maegele M, Czorlich P. Extracorporeal membrane oxygenation in traumatic brain injury - A retrospective, multicenter cohort study. Injury. 2023, 54(5):1271-1277.
2. Al-Thani H, Al-Hassani A, El-Menyar A, Asim M, Fawzy I. Outcome of post-traumatic acute respiratory distress syndrome in young patients requiring extracorporeal membrane oxygenation (ECMO). Sci Rep. 2022, 12(1):10609.
3. Henry R, Ghafil C, Piccinini A, Liasidis PK, Matsushima K, Golden A, et al. Extracorporeal support for trauma: A trauma quality improvement project (TQIP) analysis in patients with acute respiratory distress syndrome. Am J Emerg Med. 2021, 48:170-176.
4. Kim E, Song S, Kim SH, Lee NH, Lee S. Role of extracorporeal life support for traumatic hemopericardium: A single level I trauma center review. Injury. 2024, 55(1):111193.
5. Jiang Guoping, Jiang Guanyu, Ge Genxian, Xu Shiwei. Effect of emergency bedside extracorporeal membrane lung therapy on ARDS post severe trauma. 2005. Conference paper of the sixth national conference on critical illness. [In Chinese]. Available at: <https://www.doc88.com/p-9502334529043.html>. (Accessed Dec 15, 2024).
6. Guirand DM, Okoye OT, Schmidt BS, Mansfield NJ, Aden JK, Martin RS, et al. Venovenous extracorporeal life support improves survival in adult trauma patients with acute hypoxemic respiratory failure: a multicenter retrospective cohort study. J Trauma Acute Care Surg. 2014, 76(5):1275-81.
7. Lee GJ, Kim MJ, Lee JG, Lee SH. Use of venovenous extracorporeal membrane oxygenation in trauma patients with severe adult respiratory distress syndrome: A retrospective study. Int J Artif Organs. 2022, 45(10):833-840.
8. Bonacchi M, Spina R, Torracchi L, Harmelin G, Sani G, Peris A. Extracorporeal life support in patients with severe trauma: an advanced treatment strategy for refractory clinical settings. J Thorac Cardiovasc Surg. 2013, 145(6):1617-1626. 图片2012
9. Wu MY, Lin PJ, Tseng YH, Kao KC, Hsiao HL, Huang CC. Venovenous extracorporeal life support for posttraumatic respiratory distress syndrome in adults: the risk of major hemorrhages. Scand J Trauma Resusc Emerg Med. 2014, 22:56.
10. Ahmad SB, Menaker J, Kufera J, OʼConnor J, Scalea TM, Stein DM. Extracorporeal membrane oxygenation after traumatic injury. J Trauma Acute Care Surg. 2017, 82(3):587-591. doi: 10.1097/TA.0000000000001352.
11. Burke CR, Chan T, McMullan DM. Extracorporeal Life Support Use in Adult Burn Patients. J Burn Care Res. 2017, 38(3): 174-178. doi: 10.1097/BCR.0000000000000436.
12. Lin CY, Tsai FC, Lee HA, Tseng YH. Extracorporeal membrane oxygenation support in post-traumatic cardiopulmonary failure: A 10-year single institutional experience. Medicine (Baltimore). 2017, 96(6): e6067. doi: 10.1097/MD.00000000 00006067.
13. Menaker J, Tesoriero RB, Tabatabai A, Rabinowitz RP, Cornachione C, Lonergan T, et al. Veno-Venous Extracorporeal Membrane Oxygenation (VV ECMO) for Acute Respiratory Failure Following Injury: Outcomes in a High-Volume Adult Trauma Center with a Dedicated Unit for VV ECMO. World J Surg. 2018, 42(8):2398-2403. doi: 10.1007/s00268-018-4480-6.
14. Kruit N, Prusak M, Miller M, Barrett N, Richardson C, Vuylsteke A. Assessment of safety and bleeding risk in the use of extracorporeal membrane oxygenation for multitrauma patients: A multicenter review. J Trauma Acute Care Surg. 2019, 86(6):967-973.
15. Lang NW, Schwihla I, Weihs V, Kasparek M, Joestl J, Hajdu S, Sarahrudi K. Survival rate and Outcome of extracorporeal life support (ECLS) for treatment of acute cardiorespiratory failure in trauma patients. Sci Rep. 2019, 9(1):12902. doi: 10.1038/s41598-019-49346-z.
16. Weidemann F, Decker S, Epping J, Örgel M, Krettek C, Kühn C, et al. Analysis of extracorporeal membrane oxygenation in trauma patients with acute respiratory distress syndrome: A case series. Int J Artif Organs. 2022, 45(1):81-88. doi: 10.1177/0391398820980736.
17. Kim SH, Huh U, Song S, Kim MS, Wang IJ, Tak YJ. Outcomes in trauma patients undergoing veno-venous extracorporeal membrane oxygenation for acute respiratory distress syndrome. Perfusion. 2023, 38(5):1037-1044.
18. Powell EK, Reynolds TS, Webb JK, Kundi R, Cantu J, Keville M, et al. Early veno-venous extracorporeal membrane oxygenation is an effective strategy for traumatically injured patients presenting with refractory respiratory failure. J Trauma Acute Care Surg. 2023, 95(2S Suppl 1): S50-S59.
19. Ouwerkerk JJJ, Dorken-Gallastegi A, Renne BC, Lord S, He S, van Ee EPX, et al. Predictors of Mortality in Extracorporeal Membrane Oxygenation Support Patients Following Major Trauma. J Surg Res. 2023, 292: 14-21. doi: 10.1016/j.jss.2023.07. 022.
20. Austin SE, Galvagno SM, Podell JE, Teeter WA, Kundi R, Haase DJ, et al. Venovenous extracorporeal membrane oxygenation in patients with traumatic brain injuries and severe respiratory failure: A single-center retrospective analysis. J Trauma Acute Care Surg. 2024, 96(2): 332-339.
21. Baker SP, O'Neill B, Haddon W Jr, Long WB. The injury severity score: a method for describing patients with multiple injuries and evaluating emergency care. J Trauma. 1974, 14(3):187-196.
22. Hatfield J, Ohnuma T, Soto AL, Komisarow JM, Vavilala MS, Laskowitz DT, et al. Utilization and Outcomes of Extracorporeal Membrane Oxygenation Following Traumatic Brain Injury in the United States. J Intensive Care Med. 2023, 38(5):440-448. doi: 10.1177/08850666221139223.
23. Lammers D, Rokayak O, Uhlich R, Hu P, Baird E, Rakestraw S, et al. Early Use of Extracorporeal Membrane Oxygenation for Traumatically Injured Patients: A National Trauma Database Analysis. Am Surg. 2023, 89(8):3399-3405.
24. Bonacchi M, Spina R, Torracchi L, Harmelin G, Sani G, Peris A. Extracorporeal life support in patients with severe trauma: an advanced treatment strategy for refractory clinical settings. J Thorac Cardiovasc Surg. 2013, 145(6):1617-1626.

**Domain 3. Timing of initiation**

| **Question 3:** | **For adult trauma patients with acute cardiopulmonary failure refractory to conventional therapy, should ECMO be initiated earlier to improve survival?** |
| --- | --- |
| Population | Adult trauma patients with acute cardiopulmonary failure refractory to conventional therapy |
| Intervention | Early initiation |
| Comparator | Late initiation |
| Main outcomes | Critical: survival rate |
| Background | For adult trauma patients with worsening heart and lung injuries, the benefits of early initiation of ECMO as a rescue therapy following trauma versus delays in its initiation as a salvage treatment need to be clarified. Early and late initiation also need to be determined. |
| Conflict of interests | None. |

**Assessment**

| **Problem**  Is the problem a priority? | | |
| --- | --- | --- |
| **Judgement** | **Research evidence** | **Additional considerations** |
| ○ No  ○ Probably no  ○ Probably yes  ● Yes  ○ Varies  ○ Don't know | More than 75% of the experts ranked the question and outcome measure as critical important. |  |
| **Desirable Effects**  How substantial are the desirable anticipated effects? | | |
| **Judgement** | **Research evidence** | **Additional considerations** |
| ○ Trivial  ○ Small  ○ Moderate  ● Large  ○ Varies  ○ Don't know | In eight studies (122 patients), the time from injury to cannulation was within 5 days, and the pooled overall survival rate was 66% (95% CI: 58%–74%), whereas that of patients with over 5 days of time interval from injury to cannulation (17 patients, 3 studies) was 38% (95% CI: 13%–71%).  Nine studies (197 patients) reported a time interval of <5 days from admission to ECMO, with a pooled overall survival of 58% (95% CI: 39%–75%), whereas that of patients with a time interval of >5 days from admission to ECMO (12 patients, one study) was 42% (95% CI: 15%–72%).  In a single-center retrospective cohort study involving 36 TBI patients on VV-ECMO, despite a low admission Glasgow Coma Scale score, those with early cannulation had a higher survival rate and showed good neurological outcomes (OR 2.08, 95% CI: 1.34–2.35, P < 0.001) |  |
| **Undesirable Effects**  How substantial are the undesirable anticipated effects? | | |
| **Judgement** | **Research evidence** | **Additional considerations** |
| ○ Trivial  ● Small  ○ Moderate  ○ Large  ○ Varies  ○ Don't know | Either early or late initiation, ECMO may potentially cause damage to patients. |  |
| **Certainty of evidence**  **What is the overall certainty of the evidence of effects?** | | |
| **Judgement** | **Research evidence** | **Additional considerations** |
| ● Very low  ○ Low  ○ Moderate  ○ High  ○ No included studies | Very low, due to the serious risk of bias and imprecision. Substantial heterogeneity (*I*^2^=68%, τ^2^=0.77, *P* < 0.01). The sample size is insufficient. There are time differences in outcomes. |  |
| **Values and Preferences**  Is there important uncertainty about or variability in how much people value the main outcomes? | | |
| **Judgement** | **Research evidence** | **Additional considerations** |
| ○ Important uncertainty or variability  ○ Possibly important uncertainty or variability  ● Probably no important uncertainty or variability  ○ No important uncertainty or variability | Patient representative, policy-maker and consensus experts believe that early initiation once the criteria are met may benefit if cardiopulmonary failure worsens progressively. All incline to accept early intervention. |  |
| **Balance of effects**  **Does the balance between desirable and undesirable effects favor the intervention or the comparison?** | | |
| **Judgement** | **Research evidence** | **Additional considerations** |
| ○ Favors the comparison  ○ Probably favors the comparison  ○ Does not favor either the intervention or the comparison  ● Probably favors the intervention  ○ Favors the intervention  ○ Varies  ○ Don't know | Early initiation of ECMO as a rescue therapy within 5 days following trauma may confer survival benefits, whereas the delays in its initiation as a salvage treatment while attempting conventional damage control resuscitation worsen the outcomes. The stakeholders incline to accept early intervention. |  |
| **Cost effectiveness**  Does the cost-effectiveness of the intervention favor the intervention or the comparison? | | |
| **Judgement** | **Research evidence** | **Additional considerations** |
| ○ Favors the comparison  ○ Probably favors the comparison  ○ Does not favor either the intervention or the comparison  ○ Probably favors the intervention  ● Favors the intervention  ○ Varies  ○ No included studies | Expert opinion: early initiation may improve survival, shorten hospital stay and reduce expenses. |  |
| **Equity**  What would be the impact on health equity? | | |
| **Judgement** | **Research evidence** | **Additional considerations** |
| ○ Reduced  ○ Probably reduced  ○ Probably no impact  ● Probably increased  ○ Increased  ○ Varies  ○ Don't know | Early initiation may benefit patients' right to life and health. |  |
| **Acceptability**  Is the intervention acceptable to key stakeholders? | | |
| **Judgement** | **Research evidence** | **Additional considerations** |
| ○ No  ○ Probably no  ○ Probably yes  ● Yes  ○ Varies  ○ Don't know | Taking advantages and disadvantages into consideration, stakeholders prefer early intervention. |  |
| **Feasibility**  Is the intervention feasible to implement? | | |
| **Judgement** | **Research evidence** | **Additional considerations** |
| ○ No  ○ Probably no  ○ Probably yes  ● Yes  ○ Varies  ○ Don't know | Clinicians’ knowledge and experience are the main factors that affect the implementation. On the whole, it is feasible. |  |

**TYPE OF RECOMMENDATION**

| Strong recommendation against the intervention  ○ | Conditional recommendation against the intervention  ○ | Conditional recommendation for either the intervention or the comparison  ○ | Conditional recommendation for the intervention  ○ | Strong recommendation for the intervention  ● |
| --- | --- | --- | --- | --- |

**Conclusion**

| Recommendation |
| --- |
| Recommendation 6. For adult trauma patients with acute cardiopulmonary failure refractory to conventional therapy, we recommend that ECMO be initiated as early as possible provided that the initiation criteria are met (strong recommendation, very low-quality evidence). |
| Justification |
| Early initiation of ECMO as a rescue therapy within 5 days following trauma may confer survival benefits, whereas the delays in its initiation as a salvage treatment while attempting conventional management worsen the outcomes. |
| Implementation considerations |
| ECMO should be used as a rescue therapy as early as possible, provided that the ELSO criteria are met, especially in those with extensive injuries and worsening disease course. |

**References**

1. Bonacchi M, Spina R, Torracchi L, Harmelin G, Sani G, Peris A. Extracorporeal life support in patients with severe trauma: an advanced treatment strategy for refractory clinical settings. J Thorac Cardiovasc Surg. 2013, 145(6):1617-1626. 图片2012
2. Wu MY, Lin PJ, Tseng YH, Kao KC, Hsiao HL, Huang CC. Venovenous extracorporeal life support for posttraumatic respiratory distress syndrome in adults: the risk of major hemorrhages. Scand J Trauma Resusc Emerg Med. 2014, 22:56.
3. Amos T, Bannon-Murphy H, Yeung M, Gooi J, Marasco S, Udy A, Fitzgerald M. ECMO (extra corporeal membrane oxygenation) in major trauma: A 10-year single centre experience. Injury. 2021, 52(9):2515-2521.
4. Huang YK, Liu KS, Lu MS, Wu MY, Tsai FC, Lin PJ. Extracorporeal life support in post-traumatic respiratory distress patients. Resuscitation. 2009, 80(5):535-539.
5. Guirand DM, Okoye OT, Schmidt BS, Mansfield NJ, Aden JK, Martin RS, et al. Venovenous extracorporeal life support improves survival in adult trauma patients with acute hypoxemic respiratory failure: a multicenter retrospective cohort study. J Trauma Acute Care Surg. 2014, 76(5):1275-81.
6. Biderman P, Einav S, Fainblut M, Stein M, Singer P, Medalion B. Extracorporeal life support in patients with multiple injuries and severe respiratory failure: a single-center experience? J Trauma Acute Care Surg. 2013, 75(5):907-912.
7. Kim SH, Huh U, Song S, Kim MS, Wang IJ, Tak YJ. Outcomes in trauma patients undergoing veno-venous extracorporeal membrane oxygenation for acute respiratory distress syndrome. Perfusion. 2023, 38(5):1037-1044.
8. Ried M, Bein T, Philipp A, Müller T, Graf B, Schmid C, Zonies D, Diez C, Hofmann HS. Extracorporeal lung support in trauma patients with severe chest injury and acute lung failure: a 10-year institutional experience. Crit Care. 2013, 17(3): R110.
9. Powell EK, Reynolds TS, Webb JK, Kundi R, Cantu J, Keville M, et al. Early veno-venous extracorporeal membrane oxygenation is an effective strategy for traumatically injured patients presenting with refractory respiratory failure. J Trauma Acute Care Surg. 2023, 95(2S Suppl 1): S50-S59.
10. Hsu PS, Tsai YT, Lin CY, Chen SG, Dai NT, Chen CJ, et al. Benefit of extracorporeal membrane oxygenation in major burns after stun grenade explosion: Experience from a single military medical center. Burns. 2017, 43(3):674-680.
11. Eisenga J, Monday K, Blough B, Vandervest K, Lingle K, Espinoza O, Schwartz G. Extracorporeal membrane oxygenation support in the setting of penetrating traumatic injuries. J Card Surg. 2022, 37(12):4359-4361.
12. Kim HS, Ha SO, Han SJ, Kim HS, Lee SH, Jung KS, Park S. Extracorporeal Membrane Oxygenation Support in Trauma Versus Nontrauma Patients with Noninfectious Acute Respiratory Failure. Artif Organs. 2017, 41(5):431-439.
13. Strumwasser A, Tobin JM, Henry R, Guidry C, Park C, Inaba K, Demetriades D. Extracorporeal membrane oxygenation in trauma: A single institution experience and review of the literature. Int J Artif Organs. 2018, 41(12): 845-853.
14. Ull C, Schildhauer TA, Strauch JT, Swol J. Outcome measures of extracorporeal life support (ECLS) in trauma patients versus patients without trauma: a 7-year single-center retrospective cohort study. J Artif Organs. 2017, 20(2): 117-124.
15. Soussi S, Gallais P, Kachatryan L, Benyamina M, Ferry A, Cupaciu A, et al. PRONOBURN Group. Extracorporeal membrane oxygenation in burn patients with refractory acute respiratory distress syndrome leads to 28 % 90-day survival. Intensive Care Med. 2016, 42(11):1826-1827.
16. Huang JE, Holland SR, Patrick J, Piper LC, Sams VG. Predictive survival factors of the traumatically injured on venovenous extracorporeal membrane oxygenation: A Bayesian model. J Trauma Acute Care Surg. 2020, 88(1):153-159.
17. Austin SE, Galvagno SM, Podell JE, Teeter WA, Kundi R, Haase DJ, et al. Venovenous extracorporeal membrane oxygenation in patients with traumatic brain injuries and severe respiratory failure: A single-center retrospective analysis. J Trauma Acute Care Surg. 2024, 96(2): 332-339.
18. Tecos Maria, Buesing Keely, Waibel Brett, Kemp Kevin, Scriven Nicole, Evans Charity, et al. 1309: EXTRACORPOREAL MEMBRANE OXYGENATION FOR TRAUMA PATIENTS: A SINGLE-CENTER 5-YEAR RETROSPECTIVE REVIEW. Critical Care Medicine, 2023, 51(1): 654.

**Domain 4. Multidisciplinary approach**

| **Question 4:** | **In managing adult trauma patients requiring ECMO, should a multidisciplinary approach be used to improve the prognosis?** |
| --- | --- |
| Population | Adult trauma patients with acute cardiopulmonary failure refractory to conventional therapy |
| Intervention | Multidisciplinary approach |
| Comparator | Conventional therapy |
| Main outcomes | Critical: Survival rate  Important: Decision making to ECMO; time Rescue response time; Prehospital transport time. |
| Background | Interdisciplinary coordination among the emergency physicians, intensivists, trauma surgeons, and experienced staff is essential for ECMO success. |
| Conflict of interests | None. |

**Assessment**

| **Problem**  Is the problem a priority? | | |
| --- | --- | --- |
| **Judgement** | **Research evidence** | **Additional considerations** |
| ○ No  ○ Probably no  ○ Probably yes  ● Yes  ○ Varies  ○ Don't know | More than 75% of the experts ranked the question and outcome measures as critical important. |  |
| **Desirable Effects**  How substantial are the desirable anticipated effects? | | |
| **Judgement** | **Research evidence** | **Additional considerations** |
| ○ Trivial  ○ Small  ○ Moderate  ● Large  ○ Varies  ○ Don't know | Eighteen retrospective studies involving 357 ECMO-supported adult trauma patients reported the use of a multidisciplinary method in trauma ECMO management, with a pooled overall survival rate of 66% (95% CI: 60%–70%). In a multicenter retrospective analysis involving 522 ECMO-supported trauma adults, those admitted to trauma centers with ECMO capability were more likely to undergo multidisciplinary decisive surgeries and have a lower mortality rate (*OR* 0.96, 95% CI: 0.95–0.97, *P* < 0.001) | Resulting in fast reaction time for emergencies, suitable patient selection, rapid cannula insertion, debridement and definitive surgery, and safe patient transfer. |
| **Undesirable Effects**  How substantial are the undesirable anticipated effects? | | |
| **Judgement** | **Research evidence** | **Additional considerations** |
| ● Trivial  ○ Small  ○ Moderate  ○ Large  ○ Varies  ○ Don't know | It is necessary to build an efficient institutional structure responsible for the overall operation of the trauma ECMO center, standard procedures of trauma ECMO, staff training plans, and quality control standards.  Multidisciplinary management is unlikely to cause harm to patients. |  |
| **Certainty of evidence**  **What is the overall certainty of the evidence of effects?** | | |
| **Judgement** | **Research evidence** | **Additional considerations** |
| ● Very low  ○ Low  ○ Moderate  ○ High  ○ No included studies | Very low. The non-randomized study design, and the sample size is insufficient. Substantial heterogeneity (*I*^2^=68%, τ^2^=0.77, *P*=0.39). |  |
| **Values and Preferences**  Is there important uncertainty about or variability in how much people value the main outcomes? | | |
| **Judgement** | **Research evidence** | **Additional considerations** |
| ○ Important uncertainty or variability  ○ Possibly important uncertainty or variability  ○ Probably no important uncertainty or variability  ● No important uncertainty or variability | Stakeholders unanimously accept multidisciplinary ECMO management. |  |
| **Balance of effects**  **Does the balance between desirable and undesirable effects favor the intervention or the comparison?** | | |
| **Judgement** | **Research evidence** | **Additional considerations** |
| ○ Favors the comparison  ○ Probably favors the comparison  ○ Does not favor either the intervention or the comparison  ○ Probably favors the intervention  ● Favors the intervention  ○ Varies  ○ Don't know | Multidisciplinary methods may improve the survival rate up to 66%, with an OR of 0.96 (95% CI: 0.95-0.97, P < 0.001). There are no obvious undesirable consequences or adverse effects. |  |
| **Cost effectiveness**  Does the cost-effectiveness of the intervention favor the intervention or the comparison? | | |
| **Judgement** | **Research evidence** | **Additional considerations** |
| ○ Favors the comparison  ○ Probably favors the comparison  ○ Does not favor either the intervention or the comparison  ○ Probably favors the intervention  ● Favors the intervention  ○ Varies  ○ No included studies | Cost is mainly spent on establishing multidisciplinary institutions, formulating processes, training personnel and quality control. The cost is relatively low, it is worth spending, and the benefits increase. |  |
| **Equity**  What would be the impact on health equity? | | |
| **Judgement** | **Research evidence** | **Additional considerations** |
| ○ Reduced  ○ Probably reduced  ○ Probably no impact  ○ Probably increased  ● Increased  ○ Varies  ○ Don't know | Increasing equity, promoting the right to life and health of patients who are benefit. |  |
| **Acceptability**  Is the intervention acceptable to key stakeholders? | | |
| **Judgement** | **Research evidence** | **Additional considerations** |
| ○ No  ○ Probably no  ○ Probably yes  ● Yes  ○ Varies  ○ Don't know | A multidisciplinary method in trauma ECMO management has gained wide acceptance as it has been proved to improve the survival rate of patients. Theoretically, it can be used for the management of trauma ECMO. Stakeholders unanimously accept multidisciplinary approach. |  |
| **Feasibility**  Is the intervention feasible to implement? | | |
| **Judgement** | **Research evidence** | **Additional considerations** |
| ○ No  ○ Probably no  ○ Probably yes  ● Yes  ○ Varies  ○ Don't know | In low-level trauma centers or resource-deficient institutions, there may be obstacles to establishing multidisciplinary team, such as lack of personnel and experience. But it is feasible overall. |  |

**TYPE OF RECOMMENDATION**

| Strong recommendation against the intervention.  ○ | Conditional recommendation against the intervention  ○ | Conditional recommendation for either the intervention or the comparison  ○ | Conditional recommendation for the intervention  ○ | Strong recommendation for the intervention  ● |
| --- | --- | --- | --- | --- |

**Conclusion**

| Recommendation |
| --- |
| **Recommendation 7.** We recommend that a multidisciplinary team and quality improvement program be established in the management of adult trauma patients requiring ECMO (strong recommendation, very low-quality evidence). |
| Justification |
| Our meta-analyses showed a survival benefit to ECMO-supported adult trauma patients with an interdisciplinary intervention. |
| Implementation considerations |
| A regional trauma network around an ECMO-capable level I trauma center should be encouraged to maximize the access of patients to an ECMO expert center. |

**References**

1. Tonna JE, Abrams D, Brodie D, Greenwood JC, Rubio Mateo-Sidron JA, Usman A, Fan E. Management of Adult Patients Supported with Venovenous Extracorporeal Membrane Oxygenation (VV ECMO): Guideline from the Extracorporeal Life Support Organization (ELSO). ASAIO J. 2021, 67(6):601-610. doi: 10.1097/MAT.0000000 000001432.
2. Austin SE, Galvagno SM, Podell JE, Teeter WA, Kundi R, Haase DJ, et al. Venovenous extracorporeal membrane oxygenation in patients with traumatic brain injuries and severe respiratory failure: A single-center retrospective analysis. J Trauma Acute Care Surg. 2024, 96(2): 332-339.
3. Grant AA, Hart VJ, Lineen EB, Lai C, Ginzburg E, Houghton D, et al. The Impact of an Advanced ECMO Program on Traumatically Injured Patients. Artif Organs. 2018, 42(11):1043-1051.
4. Akhmerov A, Huang R, Carlson K, Dhillon NK, Ley EJ, Margulies DR, Ramzy D, Barmparas G. Access to extracorporeal life support as a quality metric: Lessons from trauma. J Card Surg. 2020, 35(4):826-830.
5. Powell EK, Reynolds TS, Webb JK, Kundi R, Cantu J, Keville M, et al. Early veno-venous extracorporeal membrane oxygenation is an effective strategy for traumatically injured patients presenting with refractory respiratory failure. J Trauma Acute Care Surg. 2023, 95(2S Suppl 1): S50-S59.
6. Chen TH, Shih JY, Shih JJ. Early Percutaneous Heparin-Free Veno-Venous Extra Corporeal Life Support (ECLS) is a Safe and Effective Means of Salvaging Hypoxemic Patients with Complicated Chest Trauma. Acta Cardiol Sin. 2016, 32(1):96-102.
7. Kim E, Song S, Kim SH, Lee NH, Lee S. Role of extracorporeal life support for traumatic hemopericardium: A single level I trauma center review. Injury. 2024, 55(1):111193.
8. ELSO Guidelines For ECMO Centers v1.8. Available at: <https://www.elso.org/ecmo-resources/elso-ecmo-guidelines.aspx>. (Accessed Dec 15, 2024).
9. Dadras M, Wagner JM, Wallner C, Huber J, Buchwald D, Strauch J, et al. Extracorporeal membrane oxygenation for acute respiratory distress syndrome in burn patients: a case series and literature update. Burns Trauma. 2019, 7:28.
10. Fouché TW, Vrouwe SQ, Gottlieb LJ, Song TH, Mehta S, Tung A, Estimé SR. Extracorporeal membrane oxygenation utilization in burn patients with severe acute respiratory distress syndrome. Burns. 2023, 49(1):244-246.
11. Kim E, Song S, Kim SH, Lee NH, Lee S. Role of extracorporeal life support for traumatic hemopericardium: A single level I trauma center review. Injury. 2024, 55(1):111193.
12. Lee A, Romano K, Tansley G, Al-Khaboori S, Thiara S, Garraway N, et al. Extracorporeal life support in trauma: Indications and techniques. J Trauma Acute Care Surg. 2024, 96(1):145-155.
13. Neubauer S, DelloStritto DJ, Capal N, Hotrum A, Henn L, Marchand T. Venovenous extracorporeal membrane oxygenation experience in a community level I trauma center. Perfusion. 2023, 38(3):484-490.
14. Bonacchi M, Spina R, Torracchi L, Harmelin G, Sani G, Peris A. Extracorporeal life support in patients with severe trauma: an advanced treatment strategy for refractory clinical settings. J Thorac Cardiovasc Surg. 2013, 145(6):1617-1626.
15. Ried M, Bein T, Philipp A, Müller T, Graf B, Schmid C, Zonies D, Diez C, Hofmann HS. Extracorporeal lung support in trauma patients with severe chest injury and acute lung failure: a 10-year institutional experience. Crit Care. 2013, 17(3): R110.
16. Tseng YH, Wu TI, Liu YC, Lin PJ, Wu MY. Venoarterial extracorporeal life support in post-traumatic shock and cardiac arrest: lessons learned. Scand J Trauma Resusc Emerg Med. 2014, 22:12.
17. Chen TH, Shih JY, Shih JJ. Early Percutaneous Heparin-Free Veno-Venous Extra Corporeal Life Support (ECLS) is a Safe and Effective Means of Salvaging Hypoxemic Patients with Complicated Chest Trauma. Acta Cardiol Sin. 2016, 32(1):96-102.
18. Huh U, Song S, Chung SW, Kim SP, Lee CW, Ahn HY, et al. Is extracorporeal cardiopulmonary resuscitation practical in severe chest trauma? A systematic review in single center of developing country. J Trauma Acute Care Surg. 2017, 83(5):903-907.
19. Grant AA, Hart VJ, Lineen EB, Lai C, Ginzburg E, Houghton D, et al. The Impact of an Advanced ECMO Program on Traumatically Injured Patients. Artif Organs. 2018, 42(11):1043-1051.
20. Ainsworth CR, Dellavolpe J, Chung KK, Cancio LC, Mason P. Revisiting extracorporeal membrane oxygenation for ARDS in burns: A case series and review of the literature. Burns. 2018, 44(6):1433-1438.
21. Amos T, Bannon-Murphy H, Yeung M, Gooi J, Marasco S, Udy A, Fitzgerald M. ECMO (extra corporeal membrane oxygenation) in major trauma: A 10-year single centre experience. Injury. 2021, 52(9):2515-2521.
22. Brewer JM, Tran A, Yu J, Ali MI, Poulos CM, Gates J, Underhill D, Gluck J. Application and outcomes of extracorporeal life support in emergency general surgery and trauma. Perfusion. 2022, 37(6):575-581.
23. Salas De Armas IA, Akkanti B, Doshi PB, Patel M, Kumar S, Akay MH, et al. Traumatic respiratory failure and veno-venous extracorporeal membrane oxygenation support. Perfusion. 2022, 37(5):477-483.
24. Powell EK, Reynolds TS, Webb JK, Kundi R, Cantu J, Keville M, et al. Early veno-venous extracorporeal membrane oxygenation is an effective strategy for traumatically injured patients presenting with refractory respiratory failure. J Trauma Acute Care Surg. 2023, 95(2S Suppl 1): S50-S59.
25. Kim SH, Huh U, Song S, Kim MS, Wang IJ, Tak YJ. Outcomes in trauma patients undergoing veno-venous extracorporeal membrane oxygenation for acute respiratory distress syndrome. Perfusion. 2023, 38(5):1037-1044.
26. Mader MM, Lefering R, Westphal M, Maegele M, Czorlich P. Extracorporeal membrane oxygenation in traumatic brain injury - A retrospective, multicenter cohort study. Injury. 2023, 54(5):1271-1277.
27. Huang JE, Holland SR, Patrick J, Piper LC, Sams VG. Predictive survival factors of the traumatically injured on venovenous extracorporeal membrane oxygenation: A Bayesian model. J Trauma Acute Care Surg. 2020, 88(1):153-159.

**Domain 5. Trauma ECMO management**

| **Question 5:** | **In managing adult trauma patients treated with ECMO, should the systemic anticoagulation goal be individualized to reduce anticoagulation-related complications?** |
| --- | --- |
| Population | Adult trauma patients with acute cardiopulmonary failure refractory to conventional therapy |
| Intervention | Individual anticoagulation |
| Comparator | Conventional therapy |
| Main outcomes | Critical: Thrombosis; Bleeding |
| Background | For trauma patients on ECMO, the anticoagulant dosage, target, and treatment course remain unclear. |
| Conflict of interests | None. |

**Assessment**

| **Problem**  Is the problem a priority? | | |
| --- | --- | --- |
| **Judgement** | **Research evidence** | **Additional considerations** |
| ○ No  ○ Probably no  ○ Probably yes  ● Yes  ○ Varies  ○ Don't know | The risks of bleeding and thrombosis in trauma patients with ECMO are seriously concerned by the intensivists and surgeons. More than 75% of the experts ranked the question and outcome measures as critical important, and agreed to include in the consensus. |  |
| **Desirable Effects**  How substantial are the desirable anticipated effects? | | |
| **Judgement** | **Research evidence** | **Additional considerations** |
| ○ Trivial  ○ Small  ● Moderate  ○ Large  ○ Varies  ○ Don't know | If bleeding is a serious concern, systemic heparinization could be withheld for 48 h to 5 days, or a pre-cannula loading dose of 50 IU/kg heparin could be administered without continuous infusion. Using a heparin-coated circuit with an ECMO blood flow of 3–5 L/min reduced the frequency of anticoagulant use and clotting time, and maintaining the platelet count above 80×10^9^/L as well as normal serum levels of clotting factors avoided the occurrence of bleeding. During trauma ECMO, the activated partial thromboplastin time and activated clotting time (ACT) were typically maintained at 45–60 and 150–200 seconds, respectively, and the antithrombin III activity, when available, was maintained at 80%–100% to ensure the efficacy of heparin and prevent bleeding. A thromboelastogram (TEG) reflects the entire profile of blood coagulation, and its early use as a monitoring tool has led to more targeted anticoagulation management. |  |
| **Undesirable Effects**  How substantial are the undesirable anticipated effects? | | |
| **Judgement** | **Research evidence** | **Additional considerations** |
| ○ Trivial  ○ Small  ○ Moderate  ○ Large  ● Varies  ○ Don't know | There is inefficient data showing that individual anticoagulation resulted in less anticoagulation-related complications in trauma patients undergoing ECMO. Two large retrospective studies showed that the incidence of complications in traumatic ARDS patients treated with VV-ECMO seemed to be higher than that in patients unexposed to ECMO, but no correlation was found between systemic heparinization and bleeding or death. |  |
| **Certainty of evidence**  **What is the overall certainty of the evidence of effects?** | | |
| **Judgement** | **Research evidence** | **Additional considerations** |
| ● Very low  ○ Low  ○ Moderate  ○ High  ○ No included studies | Based on clinical experience and expert opinions. |  |
| **Values and Preferences**  Is there important uncertainty about or variability in how much people value the main outcomes? | | |
| **Judgement** | **Research evidence** | **Additional considerations** |
| ○ Important uncertainty or variability  ○ Possibly important uncertainty or variability  ○ Probably no important uncertainty or variability  ● No important uncertainty or variability | Stakeholders tend to accept individualized anticoagulation. |  |
| **Balance of effects**  **Does the balance between desirable and undesirable effects favor the intervention or the comparison?** | | |
| **Judgement** | **Research evidence** | **Additional considerations** |
| ○ Favors the comparison  ○ Probably favors the comparison  ○ Does not favor either the intervention or the comparison  ○ Probably favors the intervention  ● Favors the intervention  ○ Varies  ○ Don't know | Most of the 49 included studies involving 990 ECMO-supported adult trauma patients reported individual anticoagulant strategies, and no significant increase in complications or deaths were found. |  |
| **Cost effectiveness**  Does the cost-effectiveness of the intervention favor the intervention or the comparison? | | |
| **Judgement** | **Research evidence** | **Additional considerations** |
| ○ Favors the comparison  ○ Probably favors the comparison  ○ Does not favor either the intervention or the comparison  ○ Probably favors the intervention  ● Favors the intervention  ○ Varies  ○ No included studies | Individualized anticoagulation strategy did not significantly increase the cost, but reduced the risk of bleeding. |  |
| **Equity**  What would be the impact on health equity? | | |
| **Judgement** | **Research evidence** | **Additional considerations** |
| ○ Reduced  ○ Probably reduced  ● Probably no impact  ○ Probably increased  ○ Increased  ○ Varies  ○ Don't know | Individualized anticoagulation strategy played no obvious roles on resource allocation, medical equality and the right to life and health. |  |
| **Acceptability**  Is the intervention acceptable to key stakeholders? | | |
| **Judgement** | **Research evidence** | **Additional considerations** |
| ○ No  ○ Probably no  ○ Probably yes  ● Yes  ○ Varies  ○ Don't know | Stakeholders tend to accept individualized anticoagulation. |  |
| **Feasibility**  Is the intervention feasible to implement? | | |
| **Judgement** | **Research evidence** | **Additional considerations** |
| ○ No  ○ Probably no  ○ Probably yes  ● Yes  ○ Varies  ○ Don't know | After weighing the benefits and damages, patients' values and preferences, and cost-effectiveness, the expert panel considered that individualized anticoagulation is feasible. |  |

**TYPE OF RECOMMENDATION**

| Strong recommendation against the intervention  ○ | Conditional recommendation against the intervention  ○ | Conditional recommendation for either the intervention or the comparison  ○ | Conditional recommendation for the intervention  ● | Strong recommendation for the intervention  ○ |
| --- | --- | --- | --- | --- |

**Conclusion**

| Recommendation |
| --- |
| **Recommendation 8.** For adult trauma patients treated with ECMO, we suggest minimal anticoagulation strategy be used to achieve individualized systemic anticoagulation goal (weak recommendation, very low-quality evidence). |
| Justification |
| In the past decades, the advances in anticoagulant methods and devices, including the centrifugal pump, methylpentene membrane oxygenator, and heparin-coated circuit, have resulted in more choices of anticoagulant strategies and less anticoagulation-related complications in trauma patients undergoing ECMO. |
| Implementation considerations |
| In deciding to implement trauma ECMO anticoagulation, the severity of injuries and risks of bleeding and thrombosis should be fully considered. In settings where point-of-care coagulation tests or TEG are not readily available, close monitoring of the patients’ vital signs, bleeding at the injured regions, limb ischemia, and circuit clotting is required. However, rapid damage control and surgical hemostasis are prerequisites before performing systemic heparinization. |

**References**

1. Austin SE, Galvagno SM, Podell JE, Teeter WA, Kundi R, Haase DJ, et al. Venovenous extracorporeal membrane oxygenation in patients with traumatic brain injuries and severe respiratory failure: A single-center retrospective analysis. J Trauma Acute Care Surg. 2024, 96(2): 332-339.
2. Dadras M, Wagner JM, Wallner C, Huber J, Buchwald D, Strauch J, et al. Extracorporeal membrane oxygenation for acute respiratory distress syndrome in burn patients: a case series and literature update. Burns Trauma. 2019, 7:28.
3. Huang YK, Liu KS, Lu MS, Wu MY, Tsai FC, Lin PJ. Extracorporeal life support in post-traumatic respiratory distress patients. Resuscitation. 2009, 80(5):535-539.
4. Kim E, Song S, Kim SH, Lee NH, Lee S. Role of extracorporeal life support for traumatic hemopericardium: A single level I trauma center review. Injury. 2024, 55(1):111193.
5. Lee A, Romano K, Tansley G, Al-Khaboori S, Thiara S, Garraway N, et al. Extracorporeal life support in trauma: Indications and techniques. J Trauma Acute Care Surg. 2024, 96(1):145-155.
6. Neubauer S, DelloStritto DJ, Capal N, Hotrum A, Henn L, Marchand T. Venovenous extracorporeal membrane oxygenation experience in a community level I trauma center. Perfusion. 2023, 38(3):484-490.
7. Perchinsky MJ, Long WB, Hill JG, Parsons JA, Bennett JB. Extracorporeal cardiopulmonary life support with heparin-bonded circuitry in the resuscitation of massively injured trauma patients. Am J Surg. 1995, 169(5):488-491. doi: 10.1016/S0002-9610(99)80201-3.
8. Chen Jianming, Zhong Jing, Song Zhiming, et al. Efficacy comparison of extracorporeal membrane oxygenation and ventilation therapy in the treatment of severe blast lung injury. Chinese Journal of Trauma. 2022, 38(11): 992-998. DOI: 10.3760/cma.j.cn501098-20220427-00323. [In Chinese]. Available at: <https://rs.yiigle.com/cmaid/1435374>. (Accessed Dec 15, 2024).
9. Liu Ying, Yuan Xiang, Qu Yuran, Li Yajie, Li Xiaohui, Yu Haibin, et al. Clinical observation of extracorporeal membrane oxygenation in severe acute respiratory distress syndrome after severe trauma. Chinese Journal of Experimental Surgery. 2023, 40(8): 1504-1507. [In Chinese]. Available at: <https://rs.yiigle.com/cmaid/1473934>. (Accessed Dec 15, 2024).
10. Fei Danting, Li Wei, Chen Weiping, Shen Yunzhong, Xu Jun, Suo Yuan, et al. Clinical analysis and literature review of 8 cases with severe trauma treated by extracorporeal membrane oxygenation. Chinese Journal of Integrated Traditional and Western Medicine in Intensive and Critical Care. 2023, 30(4): 464-467. [In Chinese]. Available at: http://www.cccm-em120.com/ zhongxiyiguokan/30/464.pdf. (Accessed Dec 15, 2024).
11. Jiang Guoping, Jiang Guanyu, Ge Genxian, Xu Shiwei. Effect of emergency bedside extracorporeal membrane lung therapy on ARDS post severe trauma. 2005. Conference paper of the sixth national conference on critical illness. [In Chinese]. Available at: <https://www.doc88.com/p-9502334529043.html>. (Accessed Dec 15, 2024).
12. Li HS, Yuan ZQ, Song HP, et al. Clinical application of extracorporeal membrane oxygenation in the treatment of burn patients with acute respiratory distress syndrome: a retrospective analysis and systematic review. Chin J Burns, 2021, 37(10): 911-920.
13. Xie gang, Jiang Chonghui, Li Binfei, et a1. Extracorporeal membrane oxygenation in severe pulmonary contusion. Clinical Medicine of China. 2005, 21(09): 817-819. [In Chinese]. Available at: <https://rs.yiigle.com/CN2021/400096.htm>. (Accessed Dec 15, 2024).
14. Anderson HL 3rd, Shapiro MB, Delius RE, Steimle CN, Chapman RA, Bartlett RH. Extracorporeal life support for respiratory failure after multiple trauma. J Trauma. 1994, 37(2): 266-72. doi: 10.1097/00005373-199408000-00020.
15. Senunas LE, Goulet JA, Greenfield ML, Bartlett RH. Extracorporeal life support for patients with significant orthopaedic trauma. Clin Orthop Relat Res. 1997, (339):32-40.
16. Cordell-Smith JA, Roberts N, Peek GJ, Firmin RK. Traumatic lung injury treated by extracorporeal membrane oxygenation (ECMO). Injury. 2006, 37(1): 29-32. doi: 10.1016/j.injury.2005.03.027.
17. Biderman P, Einav S, Fainblut M, Stein M, Singer P, Medalion B. Extracorporeal life support in patients with multiple injuries and severe respiratory failure: a single-center experience? J Trauma Acute Care Surg. 2013, 75(5):907-912.
18. Bonacchi M, Spina R, Torracchi L, Harmelin G, Sani G, Peris A. Extracorporeal life support in patients with severe trauma: an advanced treatment strategy for refractory clinical settings. J Thorac Cardiovasc Surg. 2013, 145(6):1617-1626.
19. Ried M, Bein T, Philipp A, Müller T, Graf B, Schmid C, Zonies D, Diez C, Hofmann HS. Extracorporeal lung support in trauma patients with severe chest injury and acute lung failure: a 10-year institutional experience. Crit Care. 2013, 17(3): R110.
20. Guirand DM, Okoye OT, Schmidt BS, Mansfield NJ, Aden JK, Martin RS, et al. Venovenous extracorporeal life support improves survival in adult trauma patients with acute hypoxemic respiratory failure: a multicenter retrospective cohort study. J Trauma Acute Care Surg. 2014, 76(5):1275-81.
21. Tseng YH, Wu TI, Liu YC, Lin PJ, Wu MY. Venoarterial extracorporeal life support in post-traumatic shock and cardiac arrest: lessons learned. Scand J Trauma Resusc Emerg Med. 2014, 22:12.
22. Wu MY, Lin PJ, Tseng YH, Kao KC, Hsiao HL, Huang CC. Venovenous extracorporeal life support for posttraumatic respiratory distress syndrome in adults: the risk of major hemorrhages. Scand J Trauma Resusc Emerg Med. 2014, 22:56.
23. Bosarge PL, Raff LA, McGwin G Jr, Carroll SL, Bellot SC, Diaz-Guzman E, Kerby JD. Early initiation of extracorporeal membrane oxygenation improves survival in adult trauma patients with severe adult respiratory distress syndrome. J Trauma Acute Care Surg. 2016, 81(2):236-43.
24. Wu SC, Chen WT, Lin HH, Fu CY, Wang YC, Lo HC, et al. Use of extracorporeal membrane oxygenation in severe traumatic lung injury with respiratory failure. Am J Emerg Med. 2015, 33(5):658-62. doi: 10.1016/j.ajem.2015.02.007.
25. Chen TH, Shih JY, Shih JJ. Early Percutaneous Heparin-Free Veno-Venous Extra Corporeal Life Support (ECLS) is a Safe and Effective Means of Salvaging Hypoxemic Patients with Complicated Chest Trauma. Acta Cardiol Sin. 2016, 32(1):96-102.
26. Kim HS, Ha SO, Han SJ, Kim HS, Lee SH, Jung KS, Park S. Extracorporeal Membrane Oxygenation Support in Trauma Versus Nontrauma Patients with Noninfectious Acute Respiratory Failure. Artif Organs. 2017, 41(5):431-439.
27. Ahmad SB, Menaker J, Kufera J, OʼConnor J, Scalea TM, Stein DM. Extracorporeal membrane oxygenation after traumatic injury. J Trauma Acute Care Surg. 2017, 82(3):587-591. doi: 10.1097/TA.0000000000001352.
28. Huh U, Song S, Chung SW, Kim SP, Lee CW, Ahn HY, et al. Is extracorporeal cardiopulmonary resuscitation practical in severe chest trauma? A systematic review in single center of developing country. J Trauma Acute Care Surg. 2017, 83(5):903-907.
29. Lin CY, Tsai FC, Lee HA, Tseng YH. Extracorporeal membrane oxygenation support in post-traumatic cardiopulmonary failure: A 10-year single institutional experience. Medicine (Baltimore). 2017, 96(6): e6067. doi: 10.1097/MD.00000000 00006067.
30. Grant AA, Hart VJ, Lineen EB, Lai C, Ginzburg E, Houghton D, et al. The Impact of an Advanced ECMO Program on Traumatically Injured Patients. Artif Organs. 2018, 42(11):1043-1051.
31. Ainsworth CR, Dellavolpe J, Chung KK, Cancio LC, Mason P. Revisiting extracorporeal membrane oxygenation for ARDS in burns: A case series and review of the literature. Burns. 2018, 44(6):1433-1438.
32. Strumwasser A, Tobin JM, Henry R, Guidry C, Park C, Inaba K, Demetriades D. Extracorporeal membrane oxygenation in trauma: A single institution experience and review of the literature. Int J Artif Organs. 2018, 41(12): 845-853.
33. Szentgyorgyi L, Shepherd C, Dunn KW, Fawcett P, Barker JM, Exton P, et al. Extracorporeal membrane oxygenation in severe respiratory failure resulting from burns and smoke inhalation injury. Burns. 2018, 44(5):1091-1099.
34. Kruit N, Prusak M, Miller M, Barrett N, Richardson C, Vuylsteke A. Assessment of safety and bleeding risk in the use of extracorporeal membrane oxygenation for multitrauma patients: A multicenter review. J Trauma Acute Care Surg. 2019, 86(6):967-973.
35. Lang NW, Schwihla I, Weihs V, Kasparek M, Joestl J, Hajdu S, Sarahrudi K. Survival rate and Outcome of extracorporeal life support (ECLS) for treatment of acute cardiorespiratory failure in trauma patients. Sci Rep. 2019, 9(1):12902. doi: 10.1038/s41598-019-49346-z.
36. Lee HK, Kim HS, Ha SO, Park S, Lee HS, Lee SK, Lee SH. Clinical outcomes of extracorporeal membrane oxygenation in acute traumatic lung injury: a retrospective study. Scand J Trauma Resusc Emerg Med. 2020, 28(1):41. doi: 10.1186/s13049-020-00733-w.
37. Amos T, Bannon-Murphy H, Yeung M, Gooi J, Marasco S, Udy A, Fitzgerald M. ECMO (extra corporeal membrane oxygenation) in major trauma: A 10-year single centre experience. Injury. 2021, 52(9):2515-2521.
38. Parker BM, Menaker J, Berry CD, Tesoreiero RB, O'Connor JV, et al. Single Center Experience With Veno-Venous Extracorporeal Membrane Oxygenation in Patients With Traumatic Brain Injury. Am Surg. 2021, 87(6): 949-953. doi: 10.1177/0003134820956360.
39. Al-Thani H, Al-Hassani A, El-Menyar A, Asim M, Fawzy I. Outcome of post-traumatic acute respiratory distress syndrome in young patients requiring extracorporeal membrane oxygenation (ECMO). Sci Rep. 2022, 12(1):10609.
40. Brewer JM, Tran A, Yu J, Ali MI, Poulos CM, Gates J, Underhill D, Gluck J. Application and outcomes of extracorporeal life support in emergency general surgery and trauma. Perfusion. 2022, 37(6):575-581.
41. Lee GJ, Kim MJ, Lee JG, Lee SH. Use of venovenous extracorporeal membrane oxygenation in trauma patients with severe adult respiratory distress syndrome: A retrospective study. Int J Artif Organs. 2022, 45(10):833-840.
42. Salas De Armas IA, Akkanti B, Doshi PB, Patel M, Kumar S, Akay MH, et al. Traumatic respiratory failure and veno-venous extracorporeal membrane oxygenation support. Perfusion. 2022, 37(5):477-483.
43. Trivedi JR, Alotaibi A, Sweeney JC, Fox MP, van Berkel V, Adkins K, et al. Use of Extracorporeal Membrane Oxygenation in Blunt Traumatic Injury Patients with Acute Respiratory Distress Syndrome. ASAIO J. 2022, 68(4): e60-e61.
44. Weidemann F, Decker S, Epping J, Örgel M, Krettek C, Kühn C, et al. Analysis of extracorporeal membrane oxygenation in trauma patients with acute respiratory distress syndrome: A case series. Int J Artif Organs. 2022, 45(1):81-88. doi: 10.1177/0391398820980736.
45. Powell EK, Reynolds TS, Webb JK, Kundi R, Cantu J, Keville M, et al. Early veno-venous extracorporeal membrane oxygenation is an effective strategy for traumatically injured patients presenting with refractory respiratory failure. J Trauma Acute Care Surg. 2023, 95(2S Suppl 1): S50-S59.
46. Kim SH, Huh U, Song S, Kim MS, Wang IJ, Tak YJ. Outcomes in trauma patients undergoing veno-venous extracorporeal membrane oxygenation for acute respiratory distress syndrome. Perfusion. 2023, 38(5):1037-1044.
47. Henry R, Ghafil C, Piccinini A, Liasidis PK, Matsushima K, Golden A, et al. Extracorporeal support for trauma: A trauma quality improvement project (TQIP) analysis in patients with acute respiratory distress syndrome. Am J Emerg Med. 2021, 48:170-176.
48. Menaker J, Tesoriero RB, Tabatabai A, Rabinowitz RP, Cornachione C, Lonergan T, et al. Veno-Venous Extracorporeal Membrane Oxygenation (VV ECMO) for Acute Respiratory Failure Following Injury: Outcomes in a High-Volume Adult Trauma Center with a Dedicated Unit for VV ECMO. World J Surg. 2018, 42(8):2398-2403. doi: 10.1007/s00268-018-4480-6.
49. Bein T, Zonies D, Philipp A, Zimmermann M, Osborn EC, Allan PF, et al. Transportable extracorporeal lung support for rescue of severe respiratory failure in combat casualties. J Trauma Acute Care Surg. 2012, 73(6):1450-1456.

| **Question 6:** | **In managing severely injured adult trauma patients on ECMO, should coagulopathy, hypothermia, and acidosis be closely managed to improve the prognosis?** |
| --- | --- |
| Population | Adult trauma patients with acute cardiopulmonary failure refractory to conventional therapy |
| Intervention | Closely managing coagulopathy, hypothermia, and acidosis |
| Comparator | Conventional therapy |
| Main outcomes | Critical: survival rate |
| Background | Massive bleeding or hemorrhagic shock is among the primary causes of early death in trauma patients, which may create the traumatic lethal triad in terms of coagulopathy, hypothermia, and acidosis, significantly increasing the risk of mortality of patients with massive but potentially survivable injuries. |
| Conflict of interests | None. |

**Assessment**

| **Problem**  Is the problem a priority? | | |
| --- | --- | --- |
| **Judgement** | **Research evidence** | **Additional considerations** |
| ○ No  ○ Probably no  ○ Probably yes  ● Yes  ○ Varies  ○ Don't know | Trauma-induced coagulopathy increased the mortality risk of patients by almost five times, and by four or seven times in those with a body core temperature of <35°C (OR 3.95, 95% CI: 2.90–5.4) or pH < 7.16 (OR 7.3, 95% CI: 1.39–38.11).  More than 75% of the experts ranked the question and outcome measures as critical important. |  |
| **Desirable Effects**  How substantial are the desirable anticipated effects? | | |
| **Judgement** | **Research evidence** | **Additional considerations** |
| ○ Trivial  ○ Small  ○ Moderate  ● Large  ○ Varies  ○ Don't know | ECMO support provides additional advantages, including extended damage control for severe injuries, circulatory support, and rewarming in those with hypothermia, thereby interrupting the vicious cycle of the traumatic lethal triad and conferring potential survival benefits without significant complications. |  |
| **Undesirable Effects**  How substantial are the undesirable anticipated effects? | | |
| **Judgement** | **Research evidence** | **Additional considerations** |
| ● Trivial  ○ Small  ○ Moderate  ○ Large  ○ Varies  ○ Don't know | Without significant harms and complications. |  |
| **Certainty of evidence**  **What is the overall certainty of the evidence of effects?** | | |
| **Judgement** | **Research evidence** | **Additional considerations** |
| ● Very low  ○ Low  ○ Moderate  ○ High  ○ No included studies | Based on clinical experience and expert opinions. The panel have moderate confidence in the estimate of the effects. Insufficient data and heterogeneity across the studies hindered further analyses. |  |
| **Values and Preferences**  Is there important uncertainty about or variability in how much people value the main outcomes? | | |
| **Judgement** | **Research evidence** | **Additional considerations** |
| ○ Important uncertainty or variability  ○ Possibly important uncertainty or variability  ○ Probably no important uncertainty or variability  ● No important uncertainty or variability | Stakeholders tend to accept the intervention. |  |
| **Balance of effects**  **Does the balance between desirable and undesirable effects favor the intervention or the comparison?** | | |
| **Judgement** | **Research evidence** | **Additional considerations** |
| ○ Favors the comparison  ○ Probably favors the comparison  ○ Does not favor either the intervention or the comparison  ○ Probably favors the intervention  ● Favors the intervention  ○ Varies  ○ Don't know | The damage control resuscitation (DCR) strategy, focusing on rapid bleeding control, maintaining core temperatures between 36°C and 37°C, and correcting acidosis, has become a milestone in trauma care and has significantly decreased the incidence of traumatic lethal triad and subsequent death.  Without significant complications. |  |
| **Cost effectiveness**  Does the cost-effectiveness of the intervention favor the intervention or the comparison? | | |
| **Judgement** | **Research evidence** | **Additional considerations** |
| ○ Favors the comparison  ○ Probably favors the comparison  ○ Does not favor either the intervention or the comparison  ○ Probably favors the intervention  ● Favors the intervention  ○ Varies  ○ No included studies | The cost of manpower and lab test may increase, and the thromboelastographic test requires special equipment. However, the benefit of survival rate overweighs the adverse effects of increased costs |  |
| **Equity**  What would be the impact on health equity? | | |
| **Judgement** | **Research evidence** | **Additional considerations** |
| ○ Reduced  ○ Probably reduced  ○ Probably no impact  ○ Probably increased  ● Increased  ○ Varies  ○ Don't know | No significant resource occupation or inequal distribution, the patient's right to life and health increased. |  |
| **Acceptability**  Is the intervention acceptable to key stakeholders? | | |
| **Judgement** | **Research evidence** | **Additional considerations** |
| ○ No  ○ Probably no  ○ Probably yes  ● Yes  ○ Varies  ○ Don't know | Stakeholders tend to accept the intervention. |  |
| **Feasibility**  Is the intervention feasible to implement? | | |
| **Judgement** | **Research evidence** | **Additional considerations** |
| ○ No  ○ Probably no  ○ Probably yes  ● Yes  ○ Varies  ○ Don't know | After weighing the benefits and harms, patients' values and preferences, and cost-effectiveness, the expert panel considered that the intervention is feasible. |  |

**TYPE OF RECOMMENDATION**

| Strong recommendation against the intervention  ○ | Conditional recommendation against the intervention  ○ | Conditional recommendation for either the intervention or the comparison  ○ | Conditional recommendation for the intervention  ● | Strong recommendation for the intervention  ○ |
| --- | --- | --- | --- | --- |

**Conclusion**

| Recommendation |
| --- |
| **Recommendation 9.** For adult trauma patients with massive bleeding or hemorrhagic shock, we suggest that coagulopathy, hypothermia, and acidosis be closely managed during ECMO treatment (best practice statement). |
| Justification |
| Although insufficient data and inherent defects, such as the retrospective nature, high risk of bias, and heterogeneity across the studies, hindered further analyses, considering the high mortality risk of patients with the traumatic lethal triad and the serious concern about the risk of bleeding by physicians and surgeons who are responsible for ECMO management and decisive surgery, the panel issued a best practice statement on the close monitoring and management of cases with the traumatic lethal triad during ECMO therapy. |
| Implementation considerations |
| In the context of ECMO therapy, the diagnostic indices for coagulopathy should be treated with caution, and the clinical observation, severity of injury, and physician’s judgment are still of paramount importance in the early detection of the traumatic lethal triad. |

**References**

1. Powell EK, Reynolds TS, Webb JK, Kundi R, Cantu J, Keville M, et al. Early veno-venous extracorporeal membrane oxygenation is an effective strategy for traumatically injured patients presenting with refractory respiratory failure. J Trauma Acute Care Surg. 2023, 95(2S Suppl 1): S50-S59.
2. Lee A, Romano K, Tansley G, Al-Khaboori S, Thiara S, Garraway N, et al. Extracorporeal life support in trauma: Indications and techniques. J Trauma Acute Care Surg. 2024, 96(1):145-155.
3. Brohi K, Singh J, Heron M, Coats T. Acute traumatic coagulopathy. J Trauma. 2003, 54(6): 1127-1130.
4. Burke CR, Chan T, McMullan DM. Extracorporeal Life Support Use in Adult Burn Patients. J Burn Care Res. 2017, 38(3): 174-178.
5. Balmer JC, Hieb N, Daley BJ, Many HR, Heidel E, Rowe S, McKnight CL. Continued Relevance of Initial Temperature Measurement in Trauma Patients. Am Surg. 2022, 88(3):424-428.
6. Cole E, Weaver A, Gall L, West A, Nevin D, Tallach R, et al. A decade of damage control resuscitation: new transfusion practice, new survivors, new directions. Ann Surg. 2021;273(6):1215-1220.
7. Song JC, Yang LK, Zhao W, Zhu F, Wang G, Chen YP, Li WQ. Chinese People’s Liberation Army Professional Committee of Critical Care Medicine and Chinese Society of Thrombosis, Hemostasis and Critical Care, Chinese Medicine Education Association. Chinese expert consensus on diagnosis and treatment of trauma-induced hypercoagulopathy. Mil Med Res. 2021, 8(1): 25.
8. Rossaint R, Afshari A, Bouillon B, Cerny V, Cimpoesu D, Curry N, et al. The European guideline on management of major bleeding and coagulopathy following trauma: sixth edition. Crit Care. 2023 Mar 1;27(1):80.
9. Beyersdorf C, Bieler D, Lefering R, Imach S, Hackenberg L, Schiffner E, Thelen S, Lakomek F, Windolf J, Jaekel C, TraumaRegister Dgu. Early Point-of-Care Thromboelastometry Reduces Mortality in Patients with Severe Trauma and Risk of Transfusion: An Analysis Based on the TraumaRegister DGU®. J Clin Med. 2024, 13(14):4059.

| **Question 7:** | **In managing adult trauma patients requiring ECMO, should bedside ultrasound be used to optimize fluid management?** |
| --- | --- |
| Population | Adult trauma patients with acute cardiopulmonary failure refractory to conventional therapy |
| Intervention | Bedside ultrasound |
| Comparator | Conventional monitoring |
| Main outcomes | Critical: survival rate  Important: fluid balance |
| Background | Over the last decade, bedside ultrasound has gained wide acceptance within the critical care society. Nonetheless, few studies have been conducted on ultrasound-guided fluid management for trauma patients with or without ECMO. |
| Conflict of interests | None. |

**Assessment**

| **Problem**  Is the problem a priority? | | |
| --- | --- | --- |
| **Judgement** | **Research evidence** | **Additional considerations** |
| ○ No  ○ Probably no  ○ Probably yes  ● Yes  ○ Varies  ○ Don't know | More than 75% of the experts ranked the question and outcome measures as critical important. |  |
| **Desirable Effects**  How substantial are the desirable anticipated effects? | | |
| **Judgement** | **Research evidence** | **Additional considerations** |
| ○ Trivial  ○ Small  ○ Moderate  ● Large  ○ Varies  ○ Don't know | Bedside ultrasound is a preferred visual imaging method for evaluating volume status and fluid responsiveness. Aggressive fluid resuscitation following ECMO initiation may be needed in trauma patients with hemorrhagic shock for hemodynamic stability and adequate oxygen delivery. In this situation, the principle of early fluid resuscitation guided by hemodynamics and late fluid removal once the hemodynamics has stabilized should be followed to improve the patient’s prognosis. | A large Cochrane review found that the pooled sensitivity and specificity of the bedside ultrasound in detecting hemopneumothorax, hemopericardium, or free abdominal fluid were 0.78 (95% CI: 0.69–0.84) and 0.97 (95% CI: 0.96–0.99) in an adult or mixed population. |
| **Undesirable Effects**  How substantial are the undesirable anticipated effects? | | |
| **Judgement** | **Research evidence** | **Additional considerations** |
| ● Trivial  ○ Small  ○ Moderate  ○ Large  ○ Varies  ○ Don't know | Without significant harms and complications. |  |
| **Certainty of evidence**  **What is the overall certainty of the evidence of effects?** | | |
| **Judgement** | **Research evidence** | **Additional considerations** |
| ● Very low  ○ Low  ○ Moderate  ○ High  ○ No included studies | Based on clinical experience and expert opinions. Insufficient data hindered further analyses. |  |
| **Values and Preferences**  Is there important uncertainty about or variability in how much people value the main outcomes? | | |
| **Judgement** | **Research evidence** | **Additional considerations** |
| ○ Important uncertainty or variability  ○ Possibly important uncertainty or variability  ○ Probably no important uncertainty or variability  ● No important uncertainty or variability | During ECMO, traditional hemodynamic monitoring, e.g., the transpulmonary thermodilution method, may be ineffective, whereas bedside ultrasound is a preferred visual imaging method for evaluating volume status and fluid responsiveness. Stakeholders tend to accept the intervention. |  |
| **Balance of effects**  **Does the balance between desirable and undesirable effects favor the intervention or the comparison?** | | |
| **Judgement** | **Research evidence** | **Additional considerations** |
| ○ Favors the comparison  ○ Probably favors the comparison  ○ Does not favor either the intervention or the comparison  ○ Probably favors the intervention  ● Favors the intervention  ○ Varies  ○ Don't know | Bedside ultrasound has gained wide acceptance within the critical care society due to its non-invasiveness, repeatability, and feasibility. No anticipated harms to the patients. |  |
| **Cost effectiveness**  Does the cost-effectiveness of the intervention favor the intervention or the comparison? | | |
| **Judgement** | **Research evidence** | **Additional considerations** |
| ○ Favors the comparison  ○ Probably favors the comparison  ○ Does not favor either the intervention or the comparison  ○ Probably favors the intervention  ● Favors the intervention  ○ Varies  ○ No included studies | Comparing with invasive hemodynamic monitoring, e.g., the transpulmonary thermodilution method, non-invasive bedside ultrasound is a preferred visual imaging method for evaluating volume status and fluid responsiveness. Equipment and staff training may increase the costs. However, the benefits of improving volume status and patient prognosis outweigh the costs. |  |
| **Equity**  What would be the impact on health equity? | | |
| **Judgement** | **Research evidence** | **Additional considerations** |
| ○ Reduced  ○ Probably reduced  ● Probably no impact  ○ Probably increased  ○ Increased  ○ Varies  ○ Don't know | The equipment could be used for all types of patients, and there is no resource occupation, no medical inequality, and no harm to patients' right to health. |  |
| **Acceptability**  Is the intervention acceptable to key stakeholders? | | |
| **Judgement** | **Research evidence** | **Additional considerations** |
| ○ No  ○ Probably no  ○ Probably yes  ● Yes  ○ Varies  ○ Don't know | Bedside ultrasound has gained wide acceptance within the critical care society due to its non-invasiveness, repeatability, and feasibility. Expensive equipment and operator-dependency may be the main obstacles. Both patient representative and consensus experts are inclined to accept the intervention. |  |
| **Feasibility**  Is the intervention feasible to implement? | | |
| **Judgement** | **Research evidence** | **Additional considerations** |
| ○ No  ○ Probably no  ○ Probably yes  ● Yes  ○ Varies  ○ Don't know | Bedside ultrasound has non-invasiveness, repeatability, and feasibility. The expert panel considered that the intervention is feasible. |  |

**TYPE OF RECOMMENDATION**

| Strong recommendation against the intervention  ○ | Conditional recommendation against the intervention  ○ | Conditional recommendation for either the intervention or the comparison  ○ | Conditional recommendation for the intervention  ● | Strong recommendation for the intervention  ○ |
| --- | --- | --- | --- | --- |

**Conclusion**

| Recommendation |
| --- |
| **Recommendation 10.** We suggest that bedside ultrasound be used to optimize fluid management in adult trauma patients requiring ECMO (weak recommendation, very low-quality evidence). |
| Justification |
| Given the indispensable roles of bedside ultrasound in ECMO management pre-, during, and post-cannula, particularly in ECMO blood flow management and etiology identification of hypovolemia, as well as the patients’ value and preference, the panel strongly suggested bedside ultrasound be used for fluid management in adult trauma patients requiring ECMO. |
| Implementation considerations |
| Due to the known limitations of ultrasound examination, such as considerable operator-dependency and the discrepancy between fluid responsiveness and intolerance, clinicians should not use the bedside ultrasound alone for decision-making instead of clinical evaluation. |

**References**

1. Rossaint R, Afshari A, Bouillon B, Cerny V, Cimpoesu D, Curry N, et al. The European guideline on management of major bleeding and coagulopathy following trauma: sixth edition. Crit Care. 2023 Mar 1;27(1):80. doi: 10.1186/s13054-023-04327-7.
2. Schmidt M, Bailey M, Kelly J, Hodgson C, Cooper DJ, Scheinkestel C, et al. Impact of fluid balance on outcome of adult patients treated with extracorporeal membrane oxygenation. Intensive Care Med. 2014, 40(9):1256-66. doi: 10.1007/s00134-014-3360-2.
3. Marik PE, Cavallazzi R. Does the central venous pressure predict fluid responsiveness? An updated meta-analysis and a plea for some common sense[J]. Crit Care Med, 2013, 41(7): 1774-1781. DOI: 10.1097/CCM.0b013 e31828a25fd.
4. Reuter DA, Huang C, Edrich T, et al. Cardiac output monitoring using indicator-dilution techniques: basics, limits, and perspectives. Anesth Analg. 2010, 110(3): 799-811. DOI:1 0.121 3/ANE.0b013e3181 cc885a.
5. Boehm D, Menke H. A History of Fluid Management-From "One Size Fits All" to an Individualized Fluid Therapy in Burn Resuscitation. Medicina (Kaunas). 2021 Feb 23;57(2):187. doi: 10.3390/medicina57020187.
6. Martin-Villen L, Martin-Bermudez R, Perez-Chomon H, Fuset Cabanes MP. Role of ultrasound in the critical ill patient with ECMO. Med Intensiva (Engl Ed). 2024, 48(1):46-55. doi: 10.1016/j.medine.2023.07.002.
7. Thiessen MEW, Riscinti M. Application of Focused Assessment with Sonography for Trauma in the Intensive Care Unit. Clin Chest Med. 2022, 43(3):385-392. doi: 10.1016/j.ccm.2022.05.004.
8. Stengel D, Leisterer J, Ferrada P, Ekkernkamp A, Mutze S, Hoenning A. Point-of-care ultrasonography for diagnosing thoracoabdominal injuries in patients with blunt trauma. Cochrane Database Syst Rev. 2018, 12(12): CD012669. doi: 10.1002/14651858.CD012669.pub2.

**Domain 6. Complications**

| **Question 8:** | **In adult trauma patients with acute cardiopulmonary failure requiring ECMO, should measures be taken to prevent ECMO-related complications?** |
| --- | --- |
| Population | Adult trauma patients with acute cardiopulmonary failure refractory to conventional therapy |
| Intervention | Preventive measures |
| Comparator | None |
| Main outcomes | Critical: Bleeding, Thrombosis  Important: Limb ischemia, Hemolysis, Coagulation disorders |
| Background | ECMO and anticoagulation-related complications are of great concern to clinicians in managing trauma patients receiving ECMO. |
| Conflict of interests | None. |

**Assessment**

| **Problem**  Is the problem a priority? | | |
| --- | --- | --- |
| **Judgement** | **Research evidence** | **Additional considerations** |
| ○ No  ○ Probably no  ○ Probably yes  ● Yes  ○ Varies  ○ Don't know | ECMO-relate complications and its influence on patient’s long-term outcomes played an important role on patient's preference for treatment. More than 75% of the experts ranked the question and outcome measures as critical important and important. |  |
| **Desirable Effects**  How substantial are the desirable anticipated effects? | | |
| **Judgement** | **Research evidence** | **Additional considerations** |
| ○ Trivial  ○ Small  ● Moderate  ○ Large  ○ Varies  ○ Don't know | Three retrospective cohort studies compared the incidence of complications between 144 adult trauma patients on ECMO and 1313 unexposed to ECMO. Acute renal injury was the most frequent complication, accounting for 42.1% and 19.6% for the two cohorts, followed by sepsis of 26.3% and 15.6%, thrombosis of 22.3% and 14.2%, and bleeding of 3.6% and 0.29%, respectively.  Thiry-four retrospective studies involving 675 ECMO-supported adult trauma patients reported anticoagulation-related complications. Hemorrhage occurred in 19.7% and thrombosis in 18.5% of the patients, of which 4% had hemorrhagic/ischemic stroke.  In eight retrospective studies on ECMO-supported adult trauma patients involving 32 TBI and 46 non-TBI patients, thrombosis occurred in 25.0% and 10.9% of the two cohorts, followed by hemorrhage of 15.6% and 23.9%, and stroke of 9.4% and 6.5%, respectively. |  |
| **Undesirable Effects**  How substantial are the undesirable anticipated effects? | | |
| **Judgement** | **Research evidence** | **Additional considerations** |
| ○ Trivial  ● Small  ○ Moderate  ○ Large  ○ Varies  ○ Don't know | Although the prevalence of complications seems to be higher in ECMO-supported trauma patients, no significant differences between the survivors and non-survivors. The rates of clinically important adverse events, e.g., massive intracranial hemorrhage and neurological disability, are comparable to those of trauma patients without ECMO. |  |
| **Certainty of evidence**  **What is the overall certainty of the evidence of effects?** | | |
| **Judgement** | **Research evidence** | **Additional considerations** |
| ● Very low  ○ Low  ○ Moderate  ○ High  ○ No included studies | The evidence is of indirectness, and insufficient data hindered further analyses. |  |
| **Values and Preferences**  Is there important uncertainty about or variability in how much people value the main outcomes? | | |
| **Judgement** | **Research evidence** | **Additional considerations** |
| ○ Important uncertainty or variability  ○ Possibly important uncertainty or variability  ● Probably no important uncertainty or variability  ○ No important uncertainty or variability | Both patient representative and consensus experts are inclined to accept the intervention. |  |
| **Balance of effects**  **Does the balance between desirable and undesirable effects favor the intervention or the comparison?** | | |
| **Judgement** | **Research evidence** | **Additional considerations** |
| ○ Favors the comparison  ○ Probably favors the comparison  ○ Does not favor either the intervention or the comparison  ○ Probably favors the intervention  ● Favors the intervention  ○ Varies  ○ Don't know | Although the prevalence of complications seems to be higher in ECMO-supported trauma patients, which are comparable to those of trauma patients without ECMO, and the survival benefits of ECMO might come at the expense of a potential increase in complications. |  |
| **Cost effectiveness**  Does the cost-effectiveness of the intervention favor the intervention or the comparison? | | |
| **Judgement** | **Research evidence** | **Additional considerations** |
| ○ Favors the comparison  ○ Probably favors the comparison  ○ Does not favor either the intervention or the comparison  ○ Probably favors the intervention  ● Favors the intervention  ○ Varies  ○ No included studies | Active preventive measures include immediate damage control, close anticoagulation monitoring and implementing ECMO in experienced trauma centers, which may increase the cost. However, studies showed that referring to a large-capacity ECMO center improved the survival of patients. |  |
| **Equity**  What would be the impact on health equity? | | |
| **Judgement** | **Research evidence** | **Additional considerations** |
| ○ Reduced  ● Probably reduced  ○ Probably no impact  ○ Probably increased  ○ Increased  ○ Varies  ○ Don't know | Transferring patients to a large-capacity ECMO center may increase inequality in treatment options and resource allocation, but it may increase the right to life and health of trauma patients. |  |
| **Acceptability**  Is the intervention acceptable to key stakeholders? | | |
| **Judgement** | **Research evidence** | **Additional considerations** |
| ○ No  ○ Probably no  ○ Probably yes  ● Yes  ○ Varies  ○ Don't know | Both patient representative and consensus experts are inclined to accept the intervention. |  |
| **Feasibility**  Is the intervention feasible to implement? | | |
| **Judgement** | **Research evidence** | **Additional considerations** |
| ○ No  ○ Probably no  ○ Probably yes  ● Yes  ○ Varies  ○ Don't know | After weighing the benefits and harms, patients' values and preferences, and cost-effectiveness, the expert panel considered that the intervention is feasible. |  |

**TYPE OF RECOMMENDATION**

| Strong recommendation against the intervention  ○ | Conditional recommendation against the intervention  ○ | Conditional recommendation for either the intervention or the comparison  ○ | Conditional recommendation for the intervention  ● | Strong recommendation for the intervention  ○ |
| --- | --- | --- | --- | --- |

**Conclusion**

| Recommendation |
| --- |
| **Recommendation 11.** We suggest that effective measures be taken to reduce ECMO-related complications in adult trauma patients requiring ECMO (weak recommendation, very low-quality evidence). |
| Justification |
| Although the prevalence seems to be higher in ECMO-supported trauma patients, the rates of clinically important adverse events, e.g., massive intracranial hemorrhage and neurological disability, are comparable to those of trauma patients without ECMO. Given that the survival benefits of ECMO might come at the expense of a potential increase in complications, the panel conditionally suggested that effective measures be taken to prevent ECMO-related complications. |
| Implementation considerations |
| Immediate damage control, close monitoring of anticoagulation, and implementing ECMO in large-volume and experienced trauma centers are expected to further reduce the occurrence of complications. |

**References**

1. Austin SE, Galvagno SM, Podell JE, Teeter WA, Kundi R, Haase DJ, et al. Venovenous extracorporeal membrane oxygenation in patients with traumatic brain injuries and severe respiratory failure: A single-center retrospective analysis. J Trauma Acute Care Surg. 2024, 96(2): 332-339.
2. Dadras M, Wagner JM, Wallner C, Huber J, Buchwald D, Strauch J, et al. Extracorporeal membrane oxygenation for acute respiratory distress syndrome in burn patients: a case series and literature update. Burns Trauma. 2019, 7:28.
3. Lee A, Romano K, Tansley G, Al-Khaboori S, Thiara S, Garraway N, et al. Extracorporeal life support in trauma: Indications and techniques. J Trauma Acute Care Surg. 2024, 96(1):145-155.
4. Neubauer S, DelloStritto DJ, Capal N, Hotrum A, Henn L, Marchand T. Venovenous extracorporeal membrane oxygenation experience in a community level I trauma center. Perfusion. 2023, 38(3):484-490.
5. Perchinsky MJ, Long WB, Hill JG, Parsons JA, Bennett JB. Extracorporeal cardiopulmonary life support with heparin-bonded circuitry in the resuscitation of massively injured trauma patients. Am J Surg. 1995, 169(5):488-491. doi: 10.1016/S0002-9610(99)80201-3.
6. Liu Ying, Yuan Xiang, Qu Yuran, Li Yajie, Li Xiaohui, Yu Haibin, et al. Clinical observation of extracorporeal membrane oxygenation in severe acute respiratory distress syndrome after severe trauma. Chinese Journal of Experimental Surgery. 2023, 40(8): 1504-1507. [In Chinese]. Available at: <https://rs.yiigle.com/cmaid/1473934>. (Accessed Dec 15, 2024).
7. Fei Danting, Li Wei, Chen Weiping, Shen Yunzhong, Xu Jun, Suo Yuan, et al. Clinical analysis and literature review of 8 cases with severe trauma treated by extracorporeal membrane oxygenation. Chinese Journal of Integrated Traditional and Western Medicine in Intensive and Critical Care. 2023, 30(4): 464-467. [In Chinese]. Available at: http://www.cccm-em120.com/ zhongxiyiguokan/30/464.pdf. (Accessed Dec 15, 2024).
8. Jiang Guoping, Jiang Guanyu, Ge Genxian, Xu Shiwei. Effect of emergency bedside extracorporeal membrane lung therapy on ARDS post severe trauma. 2005. Conference paper of the sixth national conference on critical illness. [In Chinese]. Available at: <https://www.doc88.com/p-9502334529043.html>. (Accessed Dec 15, 2024).
9. Xie gang, Jiang Chonghui, Li Binfei, et a1. Extracorporeal membrane oxygenation in severe pulmonary contusion. Clinical Medicine of China. 2005, 21(09): 817-819. [In Chinese]. Available at: <https://rs.yiigle.com/CN2021/400096.htm>. (Accessed Dec 15, 2024).
10. Anderson HL 3rd, Shapiro MB, Delius RE, Steimle CN, Chapman RA, Bartlett RH. Extracorporeal life support for respiratory failure after multiple trauma. J Trauma. 1994, 37(2): 266-72. doi: 10.1097/00005373-199408000-00020.
11. Senunas LE, Goulet JA, Greenfield ML, Bartlett RH. Extracorporeal life support for patients with significant orthopaedic trauma. Clin Orthop Relat Res. 1997, (339):32-40.
12. Bein T, Zonies D, Philipp A, Zimmermann M, Osborn EC, Allan PF, et al. Transportable extracorporeal lung support for rescue of severe respiratory failure in combat casualties. J Trauma Acute Care Surg. 2012, 73(6):1450-1456.
13. Bonacchi M, Spina R, Torracchi L, Harmelin G, Sani G, Peris A. Extracorporeal life support in patients with severe trauma: an advanced treatment strategy for refractory clinical settings. J Thorac Cardiovasc Surg. 2013, 145(6):1617-1626.
14. Ried M, Bein T, Philipp A, Müller T, Graf B, Schmid C, Zonies D, Diez C, Hofmann HS. Extracorporeal lung support in trauma patients with severe chest injury and acute lung failure: a 10-year institutional experience. Crit Care. 2013, 17(3): R110.
15. Guirand DM, Okoye OT, Schmidt BS, Mansfield NJ, Aden JK, Martin RS, et al. Venovenous extracorporeal life support improves survival in adult trauma patients with acute hypoxemic respiratory failure: a multicenter retrospective cohort study. J Trauma Acute Care Surg. 2014, 76(5):1275-81.
16. Tseng YH, Wu TI, Liu YC, Lin PJ, Wu MY. Venoarterial extracorporeal life support in post-traumatic shock and cardiac arrest: lessons learned. Scand J Trauma Resusc Emerg Med. 2014, 22:12.
17. Wu MY, Lin PJ, Tseng YH, Kao KC, Hsiao HL, Huang CC. Venovenous extracorporeal life support for posttraumatic respiratory distress syndrome in adults: the risk of major hemorrhages. Scand J Trauma Resusc Emerg Med. 2014, 22:56.
18. Bosarge PL, Raff LA, McGwin G Jr, Carroll SL, Bellot SC, Diaz-Guzman E, Kerby JD. Early initiation of extracorporeal membrane oxygenation improves survival in adult trauma patients with severe adult respiratory distress syndrome. J Trauma Acute Care Surg. 2016, 81(2):236-43.
19. Lin CY, Tsai FC, Lee HA, Tseng YH. Extracorporeal membrane oxygenation support in post-traumatic cardiopulmonary failure: A 10-year single institutional experience. Medicine (Baltimore). 2017, 96(6): e6067. doi: 10.1097/MD.00000000 00006067.
20. Grant AA, Hart VJ, Lineen EB, Lai C, Ginzburg E, Houghton D, et al. The Impact of an Advanced ECMO Program on Traumatically Injured Patients. Artif Organs. 2018, 42(11):1043-1051.
21. Strumwasser A, Tobin JM, Henry R, Guidry C, Park C, Inaba K, Demetriades D. Extracorporeal membrane oxygenation in trauma: A single institution experience and review of the literature. Int J Artif Organs. 2018, 41(12): 845-853.
22. Szentgyorgyi L, Shepherd C, Dunn KW, Fawcett P, Barker JM, Exton P, et al. Extracorporeal membrane oxygenation in severe respiratory failure resulting from burns and smoke inhalation injury. Burns. 2018, 44(5):1091-1099.
23. Kruit N, Prusak M, Miller M, Barrett N, Richardson C, Vuylsteke A. Assessment of safety and bleeding risk in the use of extracorporeal membrane oxygenation for multitrauma patients: A multicenter review. J Trauma Acute Care Surg. 2019, 86(6):967-973.
24. Lang NW, Schwihla I, Weihs V, Kasparek M, Joestl J, Hajdu S, Sarahrudi K. Survival rate and Outcome of extracorporeal life support (ECLS) for treatment of acute cardiorespiratory failure in trauma patients. Sci Rep. 2019, 9(1):12902. doi: 10.1038/s41598-019-49346-z.
25. Lee HK, Kim HS, Ha SO, Park S, Lee HS, Lee SK, Lee SH. Clinical outcomes of extracorporeal membrane oxygenation in acute traumatic lung injury: a retrospective study. Scand J Trauma Resusc Emerg Med. 2020, 28(1):41. doi: 10.1186/s13049-020-00733-w.
26. Amos T, Bannon-Murphy H, Yeung M, Gooi J, Marasco S, Udy A, Fitzgerald M. ECMO (extra corporeal membrane oxygenation) in major trauma: A 10-year single centre experience. Injury. 2021, 52(9):2515-2521.
27. Parker BM, Menaker J, Berry CD, Tesoreiero RB, O'Connor JV, et al. Single Center Experience With Veno-Venous Extracorporeal Membrane Oxygenation in Patients With Traumatic Brain Injury. Am Surg. 2021, 87(6): 949-953. doi: 10.1177/0003134820956360.
28. Lee GJ, Kim MJ, Lee JG, Lee SH. Use of venovenous extracorporeal membrane oxygenation in trauma patients with severe adult respiratory distress syndrome: A retrospective study. Int J Artif Organs. 2022, 45(10):833-840.
29. Salas De Armas IA, Akkanti B, Doshi PB, Patel M, Kumar S, Akay MH, et al. Traumatic respiratory failure and veno-venous extracorporeal membrane oxygenation support. Perfusion. 2022, 37(5):477-483.
30. Trivedi JR, Alotaibi A, Sweeney JC, Fox MP, van Berkel V, Adkins K, et al. Use of Extracorporeal Membrane Oxygenation in Blunt Traumatic Injury Patients with Acute Respiratory Distress Syndrome. ASAIO J. 2022, 68(4): e60-e61.
31. Weidemann F, Decker S, Epping J, Örgel M, Krettek C, Kühn C, et al. Analysis of extracorporeal membrane oxygenation in trauma patients with acute respiratory distress syndrome: A case series. Int J Artif Organs. 2022, 45(1):81-88. doi: 10.1177/0391398820980736.
32. Kim SH, Huh U, Song S, Kim MS, Wang IJ, Tak YJ. Outcomes in trauma patients undergoing veno-venous extracorporeal membrane oxygenation for acute respiratory distress syndrome. Perfusion. 2023, 38(5):1037-1044.
33. Henry R, Ghafil C, Piccinini A, Liasidis PK, Matsushima K, Golden A, et al. Extracorporeal support for trauma: A trauma quality improvement project (TQIP) analysis in patients with acute respiratory distress syndrome. Am J Emerg Med. 2021, 48:170-176.
34. Menaker J, Tesoriero RB, Tabatabai A, Rabinowitz RP, Cornachione C, Lonergan T, et al. Veno-Venous Extracorporeal Membrane Oxygenation (VV ECMO) for Acute Respiratory Failure Following Injury: Outcomes in a High-Volume Adult Trauma Center with a Dedicated Unit for VV ECMO. World J Surg. 2018, 42(8):2398-2403. doi: 10.1007/s00268-018-4480-6.
35. Nosanov LB, McLawhorn MM, Vigiola Cruz M, Chen JH, Shupp JW. A National Perspective on ECMO Utilization Use in Patients with Burn Injury. J Burn Care Res. 2017, 39(1):10-14.
36. Bein T, Zonies D, Philipp A, Zimmermann M, Osborn EC, Allan PF, et al. Transportable extracorporeal lung support for rescue of severe respiratory failure in combat casualties. J Trauma Acute Care Surg. 2012, 73(6):1450-1456.
37. Tseng YH, Wu TI, Liu YC, Lin PJ, Wu MY. Venoarterial extracorporeal life support in post-traumatic shock and cardiac arrest: lessons learned. Scand J Trauma Resusc Emerg Med. 2014, 22:12.
38. Huh U, Song S, Chung SW, Kim SP, Lee CW, Ahn HY, et al. Is extracorporeal cardiopulmonary resuscitation practical in severe chest trauma? A systematic review in single center of developing country. J Trauma Acute Care Surg. 2017, 83(5):903-907.
